# Assessment of the Safety of Glucocorticoid Regimens in Combination With Abiraterone Acetate for Metastatic Castration-Resistant Prostate Cancer: A Randomized, Open-label Phase 2 Study

**DOI:** 10.1001/jamaoncol.2019.1011

**Published:** 2019-06-27

**Authors:** Gerhardt Attard, Axel S Merseburger, Wiebke Arlt, Cora N Sternberg, Susan Feyerabend, Alfredo Berruti, Steven Joniau, Lajos Géczi, Florence Lefresne, Marjolein Lahaye, Florence Nave Shelby, Geneviève Pissart, Sue Chua, Robert J Jones, Bertrand Tombal

**Affiliations:** 1 University College London Cancer Institute, London, United Kingdom; 2 Department of Urology, University Hospital Schleswig-Holstein, Lübeck, Germany; 3 Institute of Metabolism and Systems Research (IMSR), Centre for Endocrinology, Diabetes and Metabolism, University of Birmingham, Birmingham Health Partners, NIHR Birmingham Biomedical Research Centre, University Hospitals Birmingham NHS Foundation Trust, Birmingham, United Kingdom; 4 Department of Medical Oncology, San Camillo and Forlanini Hospitals, Rome, Italy; 5 Studienpraxis Urologie, Nürtingen, Germany; 6 Department of Medical and Surgical Specialties, Radiological Sciences and Public Health, University of Brescia, Brescia, Italy; 7 Department of Urology, University Hospitals Leuven, Leuven, Belgium; 8 National Institute of Oncology, Budapest, Hungary; 9 Janssen Research and Development, Beerse, Belgium; 10 Department of Nuclear Medicine and PET/CT, Royal Marsden NHS Foundation Trust, London, United Kingdom; 11 Institute of Cancer Sciences, University of Glasgow, Glasgow, Scotland; 12 Institut de Recherche Clinique, Université Catholique de Louvain, Brussels, Belgium

## Abstract

**Importance:**

Abiraterone acetate is combined with prednisone, 5 mg, twice daily for metastatic castration-resistant prostate cancer (mCRPC) and with prednisone, 5 mg, once daily for newly diagnosed, high-risk, metastatic castration-sensitive prostate cancer. Understanding the physiological effects of these and other regimens is important.

**Objective:**

To evaluate the safety of abiraterone acetate with 4 glucocorticoid regimens.

**Design, Setting, and Participants:**

Open-label, randomized clinical trial (1:1:1:1) of 164 men with mCRPC from 22 hospitals in 5 countries who were randomly assigned to 1 of 4 intervention groups between June 2013 and October 2014. Analyses were conducted from August 2017 to June 2018.

**Interventions:**

Abiraterone acetate, 1000 mg, once daily with prednisone, 5 mg, twice daily (n = 41), 5 mg once daily (n = 41), 2.5 mg twice daily (n = 40), or dexamethasone, 0.5 mg, once daily (n = 42).

**Main Outcomes and Measures:**

Primary end point was no mineralocorticoid excess (grade ≥1 hypokalemia or grade ≥2 hypertension) through 24 weeks (6 cycles) from treatment.

**Results:**

Of 164 men (median [range] age, 70 [50-90] years) randomized to receive abiraterone acetate, 1000 mg, daily with prednisone, 5 mg, twice daily, once daily, or 2.5 mg twice daily, or dexamethasone, 0.5 mg, once daily, 24 (70.6%) of 34 patients (95% CI, 53.8%-83.2%), 14 (36.8%) of 38 patients (95% CI, 23.4%-52.7%), 21 (60.0%) of 35 patients (95% CI, 43.6%-74.4%), and 26 (70.3%) of 37 patients (95% CI, 54.2%-82.5%), respectively, had no mineralocorticoid excess. Plasma adrenocorticotrophic hormone and urinary mineralocorticoid metabolites after 8 weeks were higher with prednisone, 2.5 mg, twice daily and 5 mg once daily than with 5 mg twice daily or dexamethasone, 0.5 mg, once daily. The level of urinary glucocorticoid metabolites appeared higher in patients who did not meet the primary end point, regardless of glucocorticoid regimen. Total lean body mass decreased in the prednisone groups and total body fat increased in the prednisone, 5 mg, twice daily and dexamethasone groups. In the dexamethasone group, there was an increase in serum insulin and homeostatic model assessment of insulin resistance, while total bone mineral density decreased. In the prednisone, 5 mg, twice daily, 5 mg once daily, 2.5 mg twice daily, and dexamethasone groups, median radiographic progression-free survival was 18.5, 15.3, 12.8, and 26.6 months, respectively.

**Conclusions and Relevance:**

Abiraterone acetate with prednisone, 5 mg, twice daily or dexamethasone, 0.5 mg, once daily met the prespecified threshold for the primary end point (95% CI excluded 50% mineralocorticoid excess); abiraterone acetate with prednisone, 5 mg, once daily or 2.5 mg twice daily did not meet the threshold. Abiraterone acetate in combination with dexamethasone appeared to be particularly active but may be associated with adverse metabolic consequences.

**Trial Registration:**

ClinicalTrials.gov identifier: NCT01867710

## Key Points

**Question:**

What are the physiological effects associated with abiraterone acetate plus various glucocorticoid regimens to treat metastatic castration-resistant prostate cancer?

**Findings:**

In this open-label, phase 2 randomized clinical trial, the 164 men with metastatic castration-resistant prostate cancer treated with abiraterone acetate plus prednisone, 5 mg, twice or once daily, 2.5 mg twice daily, or dexamethasone, 0.5 mg, once daily showed no mineralocorticoid excess toxic effects (grade ≥1 hypokalemia or grade ≥2 hypertension) through cycle 6. Insulin resistance and loss of total body bone mineral density at the end of study were only significant with dexamethasone.

**Meaning:**

Lowering glucocorticoid dose combined with abiraterone acetate requires careful monitoring for toxic effects related to mineralocorticoid excess.

## Teaser

This open-label, phase 2 randomized clinical trial assesses the safety of abiraterone acetate combined with 4 glucocorticoid regimens for treatment of metastatic castration-resistant prostate cancer.

## Introduction

Abiraterone acetate, 1000 mg, once daily is approved for use in combination with either prednisone, 5 mg, twice daily or prednisone, 5 mg, once daily based on phase 3 trials reporting improvements in overall survival for the former in patients with metastatic castration-resistant prostate cancer (mCRPC)^1,2^ and for the latter in newly-diagnosed, high-risk patients with metastatic castration-sensitive prostate cancer.^3^ Prednisone, 5 mg, once daily was also used in phase 3 trials investigating the benefit of abiraterone acetate in hormone-naive advanced prostate cancer (STAMPEDE [NCT00268476]^4^ and PEACE-1 [NCT01957436]). When administered without glucocorticoids, abiraterone acetate induces a syndrome of secondary mineralocorticoid excess due to CYP17A1 17α-hydroxylase inhibition, consequently decreased glucocorticoid production, and a compensatory increase in adrenocorticotrophic hormone (ACTH), resulting in a rise in steroids with mineralocorticoid properties upstream of CYP17A1.^5,6^ In addition to maximizing efficacy for treating prostate cancer, glucocorticoids are combined with abiraterone acetate to prevent this syndrome; however, glucocorticoid exposure may exceed physiological requirements. Different glucocorticoid combination regimens have not been formally tested with abiraterone acetate. Because prolonged exposure to abiraterone acetate plus glucocorticoid is expected from earlier use, there is an urgent need to improve understanding of the physiological effects of these treatments to improve individualized treatment.

We aimed to evaluate administration of abiraterone acetate with prednisone, 5 mg, twice daily, the lower dose of 5 mg once daily used in metastatic castration-sensitive prostate cancer trials,^3,4,7^ and prednisone, 2.5 mg, twice daily. In men with progressive mCRPC, early studies of abiraterone acetate showed tumor response when combined with dexamethasone, 0.5 mg, once daily,^5,8^ and subsequent reports described tumor responses to abiraterone acetate after a switch from prednisone to dexamethasone.^9^ Therefore, our fourth treatment group received dexamethasone, 0.5 mg, once daily. The primary end point was the absence of mineralocorticoid excess in the first 24 weeks of treatment. Additionally, we assessed toxic effects related to glucocorticoid excess or androgen suppression, effects on steroid biosynthesis, and antitumor activity.

## Methods

### Patients and Study Design

In this open-label, parallel-arm, multicenter, phase 2 study, 164 asymptomatic or minimally symptomatic men with mCRPC received abiraterone acetate 1000 mg once daily and were randomly assigned in a 1:1:1:1 ratio, using an interactive web response system and a computer-generated randomization schedule, to 1 of 4 glucocorticoid regimens: prednisone, 5 mg, twice daily, prednisone, 5 mg, once daily, prednisone, 2.5 mg, twice daily, or dexamethasone, 0.5 mg, once daily. The trial protocol is available in Supplement 1. Study treatment was administered in 28-day cycles without interruption. Participants were recruited at 22 centers in 5 countries (eTable 1 in Supplement 2). They were required to have histologically or cytologically confirmed prostate adenocarcinoma with metastatic disease documented by positive bone scan, computed tomography scan, or magnetic resonance imaging and prostate cancer progression documented by prostate-specific antigen (PSA) according to the Prostate Cancer Working Group 2^10^ or radiographically according to the modified Response Evaluation Criteria In Solid Tumors (RECIST) version 1.1.^11^ Men were surgically or medically castrated, with testosterone levels of less than 50 ng/dL. Men with visceral disease were initially excluded until a protocol amendment in November 2013 (after 61 patients had been accrued), when they were permitted to enroll. Patients with prior cytotoxic chemotherapy, biologic therapy, or inhibition of the androgen receptor with abiraterone acetate plus prednisone for the treatment of mCRPC were excluded.

The planned maximum duration of the main study was 39 cycles (156 weeks) after the first participating patient started study treatment. Patients continued the main study treatment until disease progression, initiation of another prostate cancer treatment, or discontinuation of study treatment for either safety reasons (eg, sustained toxic effects that did not return to grade ≤1 with appropriate medical management) or patient request. At the end of the main study, men could continue treatment in a separate extension protocol. Patients with grade 3 or 4 hypokalemia and those who required diuretic treatment, a change in glucocorticoid dose, or palliative radiotherapy discontinued the main study treatment but could enroll in the extension protocol and restart the main study treatment after improvement to grade ≤1 without disease progression. The main study was completed in August 2016, and analyses were conducted from August 2017 to June 2018.

Study visits occurred on days 1 and 15 of cycle 1, day 1 of cycles 2 to 6, day 1 of every 3 cycles thereafter, and 4 weeks after completing the main study. Patients reported any adverse events since the prior visit, and the investigator graded each event using the National Cancer Institute Common Terminology Criteria for Adverse Events (NCI CTCAE) version 4.0. Patients with low potassium or prior history of hypokalemia could undergo weekly or more frequent laboratory electrolyte evaluation. Additional procedures are described in the eMethods in Supplement 2.

The study protocol and amendments were approved by the institutional review board at each participating site, and the study was conducted according to the provisions of the Declaration of Helsinki and the International Conference on Harmonization of Good Clinical Practice Guidelines. All patients provided written informed consent before participating.

### Outcomes

The primary end point of no mineralocorticoid excess was defined as experiencing neither of 2 mineralocorticoid excess toxic effects, grade ≥1 hypokalemia or grade ≥2 hypertension, during the first 24 weeks of treatment (ie, 6 cycles). This end point was derived from treatment emergent adverse event data, defined using the Medical Dictionary for Regulatory Activities (MedDRA)^12^ and graded according to NCI CTCAE v4.0. Secondary end points were global safety profile by NCI CTCAE v4.0; changes in plasma ACTH and urinary metabolites (eMethods and eTable 2 in Supplement 2); incidence of exogenous glucocorticoid adverse effects, namely changes in fasting serum insulin, homeostatic model assessment of insulin resistance (HOMA-IR; calculated as insulin × glucose ÷ 22.5), total lean body mass, total body fat, and bone mineral density; and clinical benefit, including antitumor activity from serum PSA values, radiographic progression-free survival, and influence on quality of life (based on the EuroQol 5-dimension questionnaire and Functional Assessment of Cancer Therapy-Prostate Cancer questionnaire). Total lean body mass and total body fat were assessed using bone density scan data.^13^ To avoid artifacts in interpretation due to bone metastases, we only report body fat changes involving the arms and total body. An exploratory secondary objective to evaluate associations with outcome and plasma androgen receptor aberrations^14^ will be reported separately.

### Trial Size Calculation and Statistical Analyses

The assumption was that for a glucocorticoid regimen to be considered of interest, 75% of patients in a treatment group would need to meet the primary end point, based on previously reported rates in a regulatory phase 3 trial.^2^ In each treatment group, an exact binomial test with a 5% 1-sided significance level had 89% power to detect the difference between 50% and 75% with a sample size of at least 30 patients; a treatment group would be rejected if 50% of patients or fewer met the primary end point. Analyses were conducted separately within each group, and the study was neither designed nor powered to compare results across groups.

Changes in steroid metabolites (after 2 cycles) and exogenous glucocorticoid adverse effects (at cycle 12 and at the end of the main study) were examined with the Wilcoxon signed rank test. Values for urinary metabolites after 2 cycles were compared among patients who met the primary end point and those who did not at 24 weeks using the Wilcoxon 2-sample test. Statistical significance was defined as *P* < .05, with no correction for multiple testing. Missing values for the end-of-main-study visit were imputed using the last-observation-carried-forward method; for other time points, only patients with data were included. Patient-reported quality of life was summarized descriptively at baseline and at cycles 6 and 18. Radiographic progression-free survival was analyzed with the Kaplan-Meier product-limit method. The trial was not designed to compare antitumor activity between the 4 treatment groups.

## Results

### Patient Characteristics

**Figure 1.**
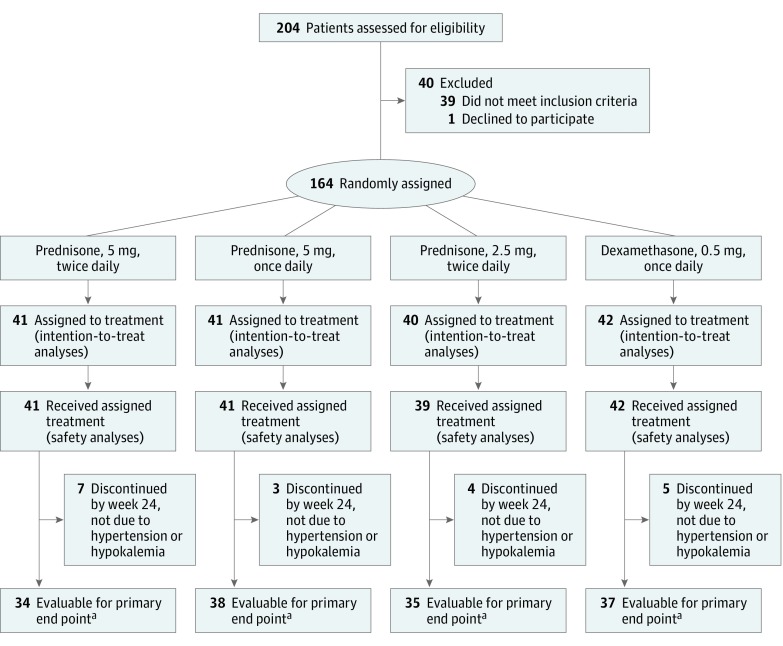
Trial Consort Diagram ^a^Evaluable patients completed the 24 weeks of treatment or discontinued treatment early and experienced either hypertension (National Cancer Institute Common Terminology Criteria for Adverse Events grade ≥2) or hypokalemia. Reasons for treatment discontinuation are provided in the eFigure in the Supplement.

Of 204 men assessed for study eligibility, 164 (median [range] age, 70 [50-90] years) were randomly assigned between June 2013 and October 2014 to 1 of 4 glucocorticoid regimens (intention-to-treat population) and 163 received at least 1 dose (safety population) (Figure 1). Patient disposition details are provided in eFigure 1 in Supplement 2. Most baseline characteristics were balanced across treatment groups, although incidences of elevated blood pressure at baseline were 46.3% for prednisone, 5 mg, once daily and ranged from 26.8% to 33.4% in the other groups. The mean time from diagnosis to randomization was 101.4 months in the dexamethasone, 0.5 mg, once daily group and ranged from 59.6 to 69.2 months in the other groups (eTable 3 in Supplement 2). Between 39.0% and 56.1% of patients (16 to 23 patients) in each treatment group were taking any antihypertensive medication at baseline. Median treatment exposure was 12.9 months overall (12.9, 10.6, 8.4, and 18.4 months in the prednisone, 5 mg, twice daily, once daily, and 2.5 mg twice daily, and dexamethasone groups, respectively), with a maximum follow-up time on treatment of 35.6 months.

### Primary End Point

Of 163 patients who received any treatment, 19 discontinued before week 24 without an adverse event of either grade 2 or higher hypertension or grade 1 or higher hypokalemia; therefore, 144 patients were evaluable for the primary end point (Figure 1). Of these patients, 85 (59.0%) experienced neither grade 2 or higher hypertension nor grade 1 or higher hypokalemia during the first 24 weeks of treatment and met the primary end point. The breakdown by treatment group was 24 of 34 patients (71%; 95% CI, 54%-83%) treated with prednisone, 5 mg, twice daily, 14 of 38 patients (37%; 95% CI, 23%-53%) treated with prednisone, 5 mg, once daily, 21 of 35 patients (60%; 95% CI, 44%-74%) treated with prednisone, 2.5 mg, twice daily, and 26 of 37 patients (70%; 95% CI, 54%-83%) treated with dexamethasone (Table). The lower boundary of the 95% CI was higher than 50% (the protocol-defined criterion) with prednisone, 5 mg, twice daily and dexamethasone but less than 50% with prednisone, 5 mg, once daily and 2.5 mg twice daily. Overall, grade 2 or higher hypertension occurred in the first 24 weeks in 49 (34.0%) of 144 evaluable patients, and grade 1 or higher hypokalemia occurred in 18 (12.5%) of 144 patients (Table), including 2 patients with grade 3 hypokalemia in the prednisone, 5 mg, once daily group.

**Table.** Primary End Point: Mineralocorticoid Excess Adverse Events During the First 24 Weeks

Variable	Treatment Group^a^			
	Prednisone, 5 mg, Twice Daily (n = 34)^b^	Prednisone, 5 mg, Once Daily (n = 38)^b^	Prednisone, 2.5 mg, Twice Daily (n = 35)^b^	Dexamethasone, 0.5 mg, Once Daily (n = 37)^b^
Met the primary end point^c^				
No. (%)	24 (71)	14 (37)	21 (60)	26 (70)
95% CI	(54-83)	(23-53)	(44-74)	(54-83)
Investigator reported an adverse event				
Grade ≥2 hypertension and grade ≥1 hypokalemia, No. (%)	2 (6)	1 (3)	3 (9)	2 (5)
Grade ≥2 hypertension alone, No. (%)	7 (21)	18 (47)	10 (29)	6 (16)
Grade ≥1 hypokalemia alone, No. (%)	1 (3)	5 (13)	1 (3)	3 (8)

^a^ Patients in all treatment groups received abiraterone acetate.

^b^ Patients completed the 24 weeks of treatment (6 cycles) or discontinued treatment early and experienced either hypertension (National Cancer Institute Common Terminology Criteria for Adverse Events grade ≥2) or hypokalemia.

^c^ Investigator reported neither hypertension (National Cancer Institute Common Terminology Criteria for Adverse Events grade ≥2) nor hypokalemia during the first 24 weeks (6 cycles).

### Treatment Effect on Steroid Biosynthesis

**Figure 2.**
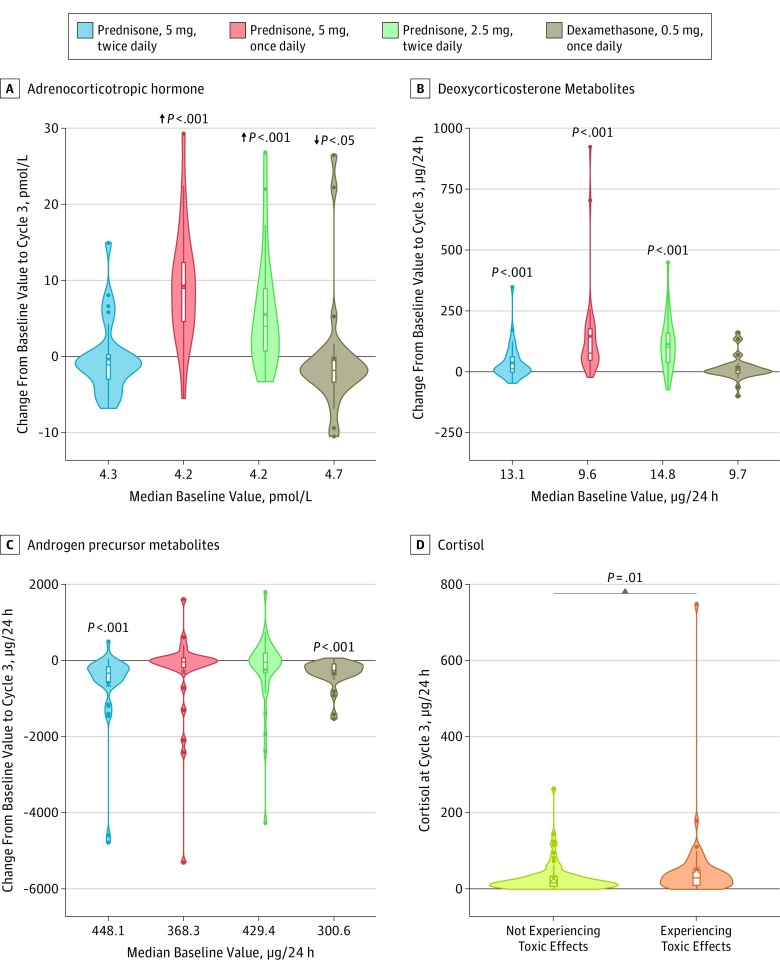
Changes From Baseline to Cycle 3 in the 4 Treatment Arms and the Association With Toxic Effects of Sum of Metabolites of Cortisol at Cycle 3 Violin plots show the changes in plasma adrenocorticotropic hormone (A), urinary deoxycorticosterone metabolites (B), and urinary sum of androgen precursor metabolites (C). Boxes represent the median (horizontal bar), mean (X), and quartiles 1 (Q1) and 3 (Q3) (lower and upper end of boxes). The whiskers indicate quartiles ±1.5 × (Q3-Q1). Minimum and maximum values are the lowest and highest dots. Significant changes from baseline, as determined by the Wilcoxon signed rank test, are noted in panels A to C. A, Arrows represent a significant decrease in the adrenocorticotropic hormone level in the dexamethasone group vs significant increases in the groups treated with prednisone, 5 mg, once daily or prednisone, 2.5 mg, twice daily. D, Significant differences in the level of metabolites of cortisol between patients experiencing or not experiencing clinical mineralocorticoid toxic effects (eg, grade ≥2 hypertension, grade ≥ 1 hypokalemia) in the first 24 weeks of treatment, as determined by the Wilcoxon 2-sample test.

In keeping with the prednisone, 2.5 mg, twice daily and 5 mg once daily groups not meeting the primary end point for absence of mineralocorticoid excess toxic effects, plasma ACTH increased significantly during treatment from baseline to cycle 3 with abiraterone plus prednisone, 5 mg, once daily (median, 8.95 pmol/L; interquartile range [IQR], 4.39-12.63) (*P* < .001) and abiraterone plus prednisone, 2.5 mg, twice daily (median, 3.97 pmol/L; IQR, 0.42-9.24) (*P* < .001). Plasma ACTH did not change significantly with prednisone, 5 mg, twice daily (median, −1.07 pmol/L; IQR, −3.08 to 0.30) (*P* = .16) and decreased significantly with dexamethasone (median, −1.82 pmol/L; IQR, −3.39 to −0.10) (*P* = .02) (Figure 2A and eTable 4 in Supplement 2).

To further evaluate upregulation of steroids upstream of CYP17A1 inhibition, urinary steroid metabolites were evaluated. From baseline to cycle 3, deoxycorticosterone metabolites significantly increased (all *P* < .001) with prednisone, 5 mg, twice daily (absolute median, 11.4 μg/24 h; IQR, −1.9 to 60.9 and median percentage change, 149%; IQR, −15% to 324%); 5 mg once daily (absolute median, 75.9 μg/24 h; IQR, 46.7 to 176.2 and median percentage change, 862%; IQR, 351%-1491%); and 2.5 mg twice daily (absolute median, 100.5 μg/24 h; IQR, 36.9 to 158.6 and median percentage change, 716%; IQR, 402%-1391%) but not with dexamethasone (Figure 2B and eTable 4 in Supplement 2). Corticosterone metabolites significantly increased (each *P* < .001) with prednisone, 5 mg, once daily (absolute median, 4452.6 μg/24 h; IQR, 2610.2-6573.0 and median percentage change, 984% IQR, 622%-1851%) and prednisone, 2.5 mg, twice daily (absolute median, 4913.5 μg/24 h; IQR, 1650.6 to 7787.9 and median percentage change, 1016%; IQR, 471%-1846%) (eFigure 2A and eTable 4 in Supplement 2). At first assessment, at cycle 3, median suppression of urinary excretion of major androgen metabolites androsterone (eFigure 2B in Supplement 2) and dehydroepiandrosterone (eFigure 2C in Supplement 2) was greater than 90% (eTable 4 in Supplement 2). Androgen precursor metabolites were also significantly suppressed (both *P* < .001) with prednisone, 5 mg, twice daily (absolute median, −330.8 μg/24 h; IQR, −555.5 to −165.8 and median percentage change, −81%; IQR, −89% to −63%) and dexamethasone (absolute median, −262.1 μg/24 h; IQR, −369.0 to −89.5 and median percentage change, −88%; IQR, −93% to −56%). However, androgen precursor metabolites were not significantly suppressed with prednisone, 5 mg, once daily or 2.5 mg twice daily (Figure 2C and eTable 4 in Supplement 2).

In exploratory analyses, 24-hour urinary excretion of corticosterone metabolites (eFigure 3A in Supplement 2), cortisol (Figure 2D), and cortisone (eFigure 3B in Supplement 2) were significantly higher in patients in any treatment group who experienced grade 2 or higher hypertension or grade 1 or higher hypokalemia in the first 24 weeks of the study compared with patients without either adverse event (eTable 5 in Supplement 2).

### Adverse Events

Over the main study, grade 3 adverse events of hypertension were reported in 7% (n = 3), 22% (n = 9), 13% (n = 5), and 7% (n = 3) of patients in the prednisone, 5 mg, twice daily, prednisone, 5 mg, once daily, prednisone, 2.5 mg, twice daily, and dexamethasone groups, respectively. Grade 3 hypokalemia was reported in 7.3% (n = 3) of patients in the prednisone, 5 mg, once daily group and in no other patients; all other events of hypokalemia were grade 1 or grade 2 (eTable 6 in Supplement 2). Neither grade 4 hypokalemia nor grade 4 hypertension were reported, and no patient discontinued study treatment for persistent hypertension. One patient in the prednisone, 5 mg, once daily group with an unrelated gastrointestinal illness had a prolonged hospitalization for a serious adverse event of hypokalemia. No patient had a serious adverse event of hypertension. Cumulative incidences of hypertension and hypokalemia over time are shown in eFigure 4 in Supplement 2. Other adverse events of interest such as peripheral edema, cardiac effects, and liver function are summarized in eTable 7 in Supplement 2.

### Long-term Metabolic and Musculoskeletal Effects

**Figure 3.**
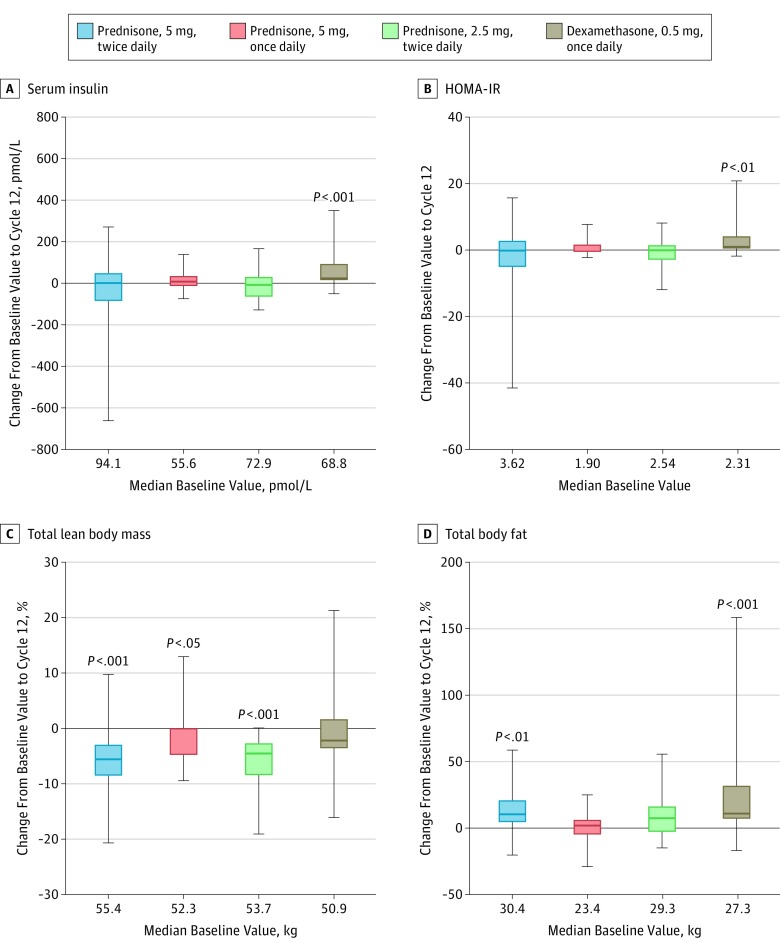
Changes From Baseline to Cycle 12 by Treatment Group Boxes represent the median (horizontal bar) value with 95% CIs. The whiskers indicate minimum and maximum values. Significant changes from baseline, by Wilcoxon signed rank test, are noted in each panel. HOMA-IR indicates homeostatic model assessment of insulin resistance.

Insulin resistance was evaluated by recording changes in fasting serum insulin or HOMA-IR. The mean change from baseline for fasting serum insulin in all patients was 9.2 pmol/L (95% CI, −14.5 to 33.0) at cycle 12 and 17.7 pmol/L (95% CI, 1.0-34.3) at the end of the main study. Significant increases in fasting serum insulin and HOMA-IR were observed in the dexamethasone group only, both at cycle 12 (fasting serum insulin, 53.7 pmol/L; 95% CI, 16.6-90.8; *P* < .001 and HOMA-IR, 2.15; 95% CI, 0.24-4.07; *P* = .004) (Figure 3A,B) and at the end of the main study (fasting serum insulin, 47.5 pmol/L; 95% CI, 18.8-76.2; *P* = .002 and HOMA-IR, 1.99; 95% CI, 0.59-3.38; *P* = .01) (eFigure 5 in Supplement 2). No patient required insulin. Two patients started medications for non–insulin-dependent diabetes (both were in the dexamethasone group).

Body composition showed a significant decrease in total lean body mass from baseline to cycle 12 in the prednisone, 5 mg, twice daily (−5.8%; 95% CI, −8.6% to −3.0%) (*P* < .001), prednisone, 5 mg, once daily (−2.5%; 95% CI, −4.9% to -0.0%) (*P* = .02), and prednisone, 2.5 mg, twice daily groups (−5.6%; 95% CI, −8.5% to −2.6%; *P* < .001) (Figure 3C) and in all groups at the end of the main study (eFigure 5 in Supplement 2). In keeping with higher glucocorticoid exposure, a significant increase in total body fat in the prednisone, 5 mg, twice daily and dexamethasone groups was observed when comparing baseline to cycle 12 (12.3%; *P* = .001 and 19.2%; *P* < .001, respectively) (Figure 3D) or to the end of the main study (eFigure 5 in Supplement 2) but was not observed with the lower glucocorticoid regimens. A small but significant decrease in total body bone mineral density from baseline to the end of the main study was seen in the dexamethasone group (−2.0%; 95% CI, −3.4% to −0.6%); no other change in the bone mineral density of the total body or the arms was observed at cycle 12 or at the end of the main study (eTable 8 in Supplement 2).

### Clinical Benefit

**Figure 4.**
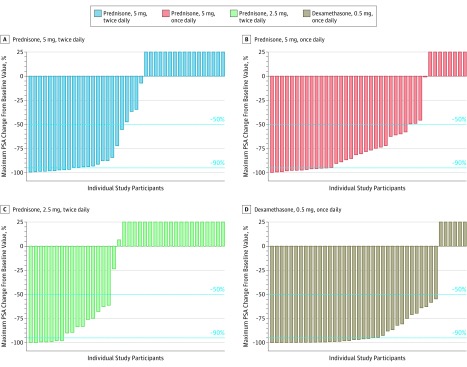
Prostate-Specific Antigen Declines by Treatment Group Maximum percent change in prostate specific antigen (PSA) level from baseline during the main study for abiraterone acetate with prednisone, 5 mg, twice daily (n = 41), prednisone, 5 mg, once daily (n = 40), prednisone, 2.5 mg, twice daily (n = 38), or dexamethasone, 0.5 mg, once daily (n = 41). Dotted lines represent the PSA responders (50% reduction and 90% reduction in PSA). Prostate-specific antigen plots for prednisone, 5 mg, once daily, prednisone, 2.5 mg, twice daily, and dexamethasone, 0.5 mg, once daily exclude patients who were missing either baseline or postbaseline PSA values. Increases greater than 25% were cut off at 25%.

Among the 164 randomized patients evaluated, a PSA decrease of greater than or equal to 50% was observed in 26 patients (63.4%; 95% CI, 48.1%-76.4%) treated with prednisone, 5 mg, twice daily, 32 (78.0%; 95% CI, 63.3%-88.0%) treated with prednisone, 5 mg, once daily, 24 (60.0%; 95% CI, 44.6%-73.7%) treated with prednisone, 2.5 mg, twice daily, and 37 (88.1%; 95% CI, 75.0%-94.8%) treated with dexamethasone (Figure 4 and eTable 9 in Supplement 2). Median radiographic progression-free survival was 18.5 months (95% CI, 10.0-26.7) with prednisone, 5 mg, twice daily, 15.3 months (95% CI, 8.4-29.5) with prednisone, 5 mg, once daily, 12.8 months (95% CI, 7.4-20.9) with prednisone, 2.5 mg, twice daily, and 26.6 months (95% CI, 20.9-not evaluable) with dexamethasone (eFigure 6A in Supplement 2). Patient-reported quality of life in this population, which was asymptomatic or minimally symptomatic pretreatment, remained stable in all groups, supporting no detectable reduction secondary to treatment (eFigure 6B in Supplement 2).

## Discussion

Abiraterone acetate plus prednisone is being used earlier in the treatment paradigm for prostate cancer and because patients benefit for a longer period, treatment exposure is increased. We therefore designed a randomized clinical trial to support physician decision making in selecting a glucocorticoid regimen that balances controlling manifestations of endogenous mineralocorticoid excess with the adverse consequences of nonphysiological exposure to exogenous glucocorticoids. Manifestations of endogenous mineralocorticoid excess often present within 24 weeks of treatment, whereas the adverse consequences associated with exogenous glucocorticoids are usually associated with cumulative exposure. Prednisone, 5 mg, twice daily and dexamethasone, 0.5 mg, once daily met the prespecified threshold for the primary end point (the 95% CI excluded 50% mineralocorticoid excess), but prednisone, 5 mg, once daily and 2.5 mg twice daily did not meet the threshold.

We selected hypertension and hypokalemia as indicators of clinically relevant mineralocorticoid excess because other clinical features, including lower limb edema and general fluid overload, are less feasible to standardize in a multicenter trial. To support our clinical end point, we confirmed a significant rise in plasma ACTH and a subsequent rise in mineralocorticoid production with the lower prednisone doses. We hypothesize that monitoring of urinary glucocorticoid metabolite levels could identify patients at risk of mineralocorticoid excess and allow tailoring of the glucocorticoid dose.

A strength of the trial was monitoring for long-term adverse effects of glucocorticoid excess. Total lean body mass decreased significantly in all 3 prednisone groups and total body fat increased significantly in the prednisone, 5 mg, twice daily and dexamethasone groups. Significant increases in insulin resistance and decreases in total bone mineral density were observed only with dexamethasone. Androgen precursors were significantly suppressed with dexamethasone, 0.5 mg, once daily and prednisone, 5 mg, twice daily. The antitumor activity associated with abiraterone acetate plus glucocorticoids in the present study was similar to previous reports^1,2^ in this disease setting; dexamethasone appeared particularly active but may be associated with adverse metabolic consequences.

### Limitations

A limitation of the trial is that the size calculation was based on the primary end point related to the safety of individual regimens; therefore, the number of patients accrued does not allow for direct comparisons among the 4 treatment groups. Second, although patients were randomly assigned to treatment, differences at baseline could contribute to differences in outcomes. Third, testing for physiological effects and their association with clinical manifestation of mineralocorticoid excess were exploratory analyses, and we did not correct for multiple testing. Overall, the observations of our phase 2 trial require further validation.

## Conclusions

This trial provides results consistent with the approved use of abiraterone acetate with prednisone, 5 mg, twice daily for the treatment of mCRPC. Long-term adverse metabolic and musculoskeletal changes are small and do not appear to have a detrimental effect on patient-reported quality of life. Different glucocorticoid regimens make distinct compromises on control of mineralocorticoid excess, changes in body composition, and development of insulin resistance. With careful monitoring, the risk of hypokalemia with lower glucocorticoid doses can be mitigated, as demonstrated in the LATITUDE and STAMPEDE trials where no major safety concerns were raised.^3,4^ A lower dose of prednisone appears to reduce long-term risks of insulin resistance, increased body fat, and loss of bone mineral density.

## Notes

Supplement 1. Trial Protocol

Supplement 2. eMethods eReferences eFigure 1. Patient Disposition eFigure 2. Changes from baseline to cycle 3 by treatment group for urinary corticosterone metabolites (A), the major androgen metabolite androsterone (B), and DHEA (C); and values at cycle 3 for the urinary metabolites of corticosterone (D) and cortisone (E) among patients experiencing or not experiencing clinical mineralocorticoid excess toxicity (grade ≥2 hypertension or grade ≥1 hypokalemia) in the first 24 weeks eFigure 3. Values at cycle 3 for the urinary metabolites of corticosterone (A) and cortisone (B) among patients experiencing or not experiencing clinical mineralocorticoid excess toxicity (grade ≥2 hypertension or grade ≥1 hypokalemia) in the first 24 weeks eFigure 4. Cumulative incidences for adverse events of hypokalemia or hypertension eFigure 5. Change from baseline in fasting serum insulin (A) and HOMA-IR (B), and percentage change from baseline in total lean body mass (C) and total body fat (D) at end of main study (up to 39 cycles) eFigure 6. Clinical benefit: Kaplan-Meier plot of radiographic progression-free survival during the main study for intention-to-treat population, divided by treatment group (A); and patient-reported quality of life as measured by EQ-5D-5L and FACT-P (B) eTable 1. Study investigators and centers eTable 2. Full list of urinary steroid metabolites analyzed by gas chromatography-mass spectrometry eTable 3. Baseline characteristics eTable 4. Changes from baseline to cycle 3 by treatment group for plasma ACTH and key urinary steroid metabolites eTable 5. Urinary steroid metabolites with P<0.1 for the comparison of values at cycle 3 day 1 between patients with or without clinical mineralocorticoid excess toxicity in the first 24 weeks eTable 6. Adverse events of hypokalemia by treatment group and severity eTable 7. Adverse events of special interest during the main study (up to 39 cycles) eTable 8. Changes from baseline in bone mineral density - total body and arms eTable 9. Number of patients with any PSA response and confirmed PSA response during the main study (up to 39 cycles)

Supplement 3. Data Sharing Statement
