# Supplementary material for: Assessment of the Safety of Glucocorticoid Regimens in Combination With Abiraterone Acetate for Metastatic Castration-Resistant Prostate Cancer: A Randomized, Open-label Phase 2 Study
Source: JAMA Oncol. 2019 Jun 27;5(8):1159–67. doi: 10.1001/jamaoncol.2019.1011 (PMC6604092; doi:10.1001/jamaoncol.2019.1011)
Supplement: Supplement 1. — Trial Protocol [file jamaoncol-5-1159-s001.pdf]

Janssen EMEA*

Clinical Protocol

A Randomized Phase 2 Study Evaluating Abiraterone Acetate With Different Steroid Regimens for Preventing Symptoms Associated With Mineralocorticoid Excess in Asymptomatic, Chemotherapy-Naïve and Metastatic Castration-Resistant Prostate Cancer (mCRPC) Patients

Protocol 212082-PCR-2023; Phase 2
AMENDMENT INT-5

JNJ-212082 (abiraterone acetate)

*Janssen EMEA is an organization in Europe, the Middle East, and Africa that operates through different legal entities in various countries. The legal entity acting as the sponsor for Janssen EMEA studies may vary, such as, but not limited to Janssen Pharmaceutica NV. The term "sponsor" is used throughout the protocol to represent these various legal entities; the sponsor is identified on the Contact Information page that accompanies the protocol.

EudraCT NUMBER: 2012-004331-23

**Status:**	Approved
**Date:**	28 January 2016
**Prepared by:**	Janssen Pharmaceutica NV
**Document No.:**	EDMS-ERI-50635007; 6.0

GCP Compliance: This study will be conducted in compliance with Good Clinical Practice and applicable regulatory requirements.

Confidentiality Statement
The information in this document contains trade secrets and commercial information that are privileged or confidential and may not be disclosed unless such disclosure is required by applicable law or regulations. In any event, persons to whom the information is disclosed must be informed that the information is privileged or confidential and may not be further disclosed by them. These restrictions on disclosure will apply equally to all future information supplied to you that is indicated as privileged or confidential.

INVESTIGATOR AGREEMENT

I have read this protocol and agree that it contains all necessary details for carrying out this study. I will conduct the study as outlined herein and will complete the study within the time designated.

I will provide copies of the protocol and all pertinent information to all individuals responsible to me who assist in the conduct of this study. I will discuss this material with them to ensure that they are fully informed regarding the study drug, the conduct of the study, and the obligations of confidentiality.

Coordinating Investigator (where required):

Name (typed or printed): Dr Gerhardt Attard

Institution and Address: The Royal Marsden NHS Foundation Trust
Downs Road, Sutton
Surrey SM2 5PT
United Kingdom

Signature: ____________________ Date: ____________
(Day Month Year)

Principal (Site) Investigator:

Name (typed or printed): ____________________

Institution and Address: ____________________

Telephone Number: ____________________

Signature: ____________________ Date: ____________
(Day Month Year)

Sponsor's Responsible Medical Officer:

Name (typed or printed): LEFRESNE

Institution: JANSSEN

Signature: Lefresne Date: 09/02/2016
(Day Month Year)

Note: If the address or telephone number of the investigator changes during the course of the study, written notification will be provided by the investigator to the sponsor, and a protocol amendment will not be required.

TABLE OF CONTENTS

INVESTIGATOR AGREEMENT 2

TABLE OF CONTENTS 3

LIST OF ATTACHMENTS 5

LIST OF IN-TEXT TABLES AND FIGURES 5

PROTOCOL AMENDMENTS 6

SYNOPSIS 20

TIME AND EVENTS SCHEDULE 27

TIME AND EVENTS SCHEDULE – LABORATORY TEST SCHEDULE 30

ABBREVIATIONS 32

1. INTRODUCTION 34
1.1. Concurrent Glucocorticoids 38
1.2. Overall Rationale for the Study 39

2. OBJECTIVES AND HYPOTHESIS 40
2.1. Objectives 40
2.2. Hypothesis 40

3. STUDY DESIGN AND RATIONALE 41
3.1. Overview of Study Design 41
3.1.1. Assessments 43
3.1.2. Extension and Follow-up Phases 44
3.1.3. Study Duration 45
3.2. Study Design Rationale 47

4. SUBJECT SELECTION 50
4.1. Inclusion Criteria 51
4.2. Exclusion Criteria 52
4.3. Prohibitions and Restrictions 54

5. TREATMENT ALLOCATION AND BLINDING 55

6. DOSAGE AND ADMINISTRATION 55

7. TREATMENT COMPLIANCE 56

8. CONCOMITANT THERAPY 57

9. STUDY EVALUATIONS 59
9.1. Study Procedures 59
9.1.1. Overview 59
9.1.2. Screening Phase 60
9.1.3. Open-Label Main Study Treatment Period 61
9.1.4. Open-Label Extension Phase 62
9.1.5. Follow-Up Phase 63
9.2. Safety 63
9.2.1. Evaluations 63
9.2.2. Endpoints 71
9.3. Efficacy 71

9.3.1. Evaluations 71
9.3.2. Endpoints 73
9.4. Biomarkers 74
9.5. Medical Resource Utilization 75
9.6. Sample Collection and Handling 75

10. SUBJECT COMPLETION/WITHDRAWAL 76
10.1. Completion 76
10.2. Discontinuation of Treatment 76
10.3. Withdrawal From the Study 78

11. STATISTICAL METHODS 79
11.1. Subject Information 79
11.2. Sample Size Determination 79
11.3. Safety Analyses 80
11.4. Efficacy Analyses 81
11.5. Extension Phase and Follow-up Phase Data 81
11.6. Biomarker Analyses 82
11.7. Medical Resource Utilization Analyses 82
11.8. Scientific Advisory Committee 82

12. ADVERSE EVENT REPORTING 82
12.1. Definitions 82
12.1.1. Adverse Event Definitions and Classifications 82
12.1.2. Attribution Definitions 84
12.1.3. Severity Criteria 84
12.2. Special Reporting Situations 85
12.3. Procedures 85
12.3.1. All Adverse Events 85
12.3.2. Serious Adverse Events 86
12.3.3. Pregnancy 87
12.4. Contacting Sponsor Regarding Safety 87

13. PRODUCT QUALITY COMPLAINT HANDLING 87
13.1. Procedures 87
13.2. Contacting Sponsor Regarding Product Quality 87

14. STUDY DRUG INFORMATION 88
14.1. Physical Description of Study Drugs 88
14.2. Packaging 88
14.3. Labeling 88
14.4. Preparation, Handling, and Storage 88
14.5. Drug Accountability 88

15. STUDY-SPECIFIC MATERIALS 89

16. ETHICAL ASPECTS 89
16.1. Study-Specific Design Considerations 89
16.2. Regulatory Ethics Compliance 91
16.2.1. Investigator Responsibilities 91
16.2.2. Independent Ethics Committee or Institutional Review Board 91
16.2.3. Informed Consent 92
16.2.4. Privacy of Personal Data 93
16.2.5. Long-Term Retention of Samples for Biomarker Research 94
16.2.6. Country Selection 94

17. ADMINISTRATIVE REQUIREMENTS 94
17.1. Protocol Amendments 94
17.2. Regulatory Documentation 95

17.2.1. Regulatory Approval/Notification ... 95
17.2.2. Required Prestudy Documentation ... 95
17.3. Subject Identification, Enrollment, and Screening Logs ... 96
17.4. Source Documentation ... 96
17.5. Case Report Form Completion ... 97
17.6. Data Quality Assurance/Quality Control ... 97
17.7. Record Retention ... 98
17.8. Monitoring ... 98
17.9. Study Completion/Termination ... 99
17.9.1. Study Completion ... 99
17.9.2. Study Termination ... 99
17.10. On-Site Audits/Inspections ... 99
17.11. Use of Information and Publication ... 99

REFERENCES ... 102

LAST PAGE ... 121

LIST OF ATTACHMENTS

Attachment 1: Summary of Response Evaluation Criteria in Solid Tumors (RECIST, Version 1.1) ... 106
Attachment 2: BPI-SF ... 112
Attachment 3: ECOG Performance Status ... 114
Attachment 4: EQ-5D-5L (Sample UK English Version) ... 115
Attachment 5: FACT-P (Sample UK English Version) ... 117
Attachment 6: Protocol Specific PCWG2 Criteria ... 120
Attachment 7: NCI CTCAE v4.0 ... 121

LIST OF IN-TEXT TABLES AND FIGURES

TABLES

Table 1: Definition of Grade ≥1 Hypokalemia (NCI CTCAE v4.0) ... 65
Table 2: Definition of Grade ≥2 Hypertension (NCI CTCAE v4.0) ... 67

FIGURES

Figure 1: The Suppression of Estradiol by Abiraterone Acetate's Inhibition of CYP17 in Men With CRPC ... 36
Figure 2: Diagram of the Study Design ... 42
Figure 3: Summary of the Main Study, Extension Phase and Follow-up Phase ... 46

PROTOCOL AMENDMENTS

Protocol Version	Issue Date
Original Protocol	28 Nov 2012
Amendment INT-1	25 Feb 2013
Amendment INT-2	13 Nov 2013
Amendment INT-3	22 Apr 2014
Amendment INT-4	27 Nov 2014
Amendment INT-5	28 Jan 2016

Amendments are listed beginning with the most recent amendment.

Amendment INT-5 (28 January 2016)

This amendment is considered to be nonsubstantial based on the criteria set forth in Article 10(a) of Directive 2001/20/EC of the European Parliament and the Council of the European Union, in that it does not significantly impact the safety or physical/mental integrity of subjects, nor the scientific value of the study.

The overall reason for the amendment: To enable initiation of osteoprotective therapy during the study and to clarify data to be collected.

Applicable Section(s)	Description of Change(s)
**Rationale:** To enable initiation of bisphosphonates or other approved osteoprotective agents during the study.	
Synopsis, Dosage and Administration; Time and Events Schedule (footnote u); 3.1. Overview of Study Design; 6. Dosage and Administration; 8. Concomitant Therapy	Changed text to indicate that initiation of bisphosphonates or other approved osteoprotective agents during the study will be allowed after appropriate documentation of the bone loss, and according to local clinical practice and the current product label.
**Rationale:** To clarify that palliative radiotherapy during the extension phase must be documented.	
8. Concomitant Therapy; 9.1.4. Open-label Extension Phase	Clarified that information on palliative radiotherapy during the extension phase must be recorded in the eCRF.
Time and Events Schedule (footnote u)	Added a footnote to clarify that all therapies must be documented in the eCRF and that palliative radiotherapy is allowed during the extension phase.
**Rationale:** To provide additional information on prohibited therapies.	
8. Concomitant Therapy; 10.2. Discontinuation of Treatment	Clarified that concurrent anticancer therapies prohibited during the main study treatment period and the extension phase include the bone-targeted alpha emitter (Radium 223).

Applicable Section(s)	Description of Change(s)
**Rationale:** To clarify the dates of the main study treatment period cut-off and end of the follow-up phase, and the timing of EOMT assessments.	
Synopsis, Overview of Study Design; Time and Events Schedule (footnote c); Time and Events Schedule – Laboratory Test Schedule (footnote c); 3.1. Overview of Study Design; 9.1.1. Overview; 9.1.3. Open-Label Main Study Treatment Period	Since the first subject started study treatment in July 2013, text was added to clarify that the end of main study treatment period cut-off date will be in July 2016.
Synopsis, Overview of Study Design; Time and Events Schedule (footnotes e, v); 3.1.2. Extension and Follow-up Phases; 3.1.3. Study duration; 9.1.1. Overview; 9.1.5. Follow-Up Phase; 10.1. Completion; 10.2. Discontinuation of Treatment	Clarified that the follow-up phase will end in July 2018.
Time and Events Schedule (footnote c); Time and Events Schedule – Laboratory Test Schedule (footnote c); 3.1. Overview of Study Design; 9.1.1. Overview; 9.1.3. Open-Label Main Study Treatment Period	Clarified that, for subjects who have completed the main study treatment period, EOMT assessments will be performed by 16 July 2016±15 days.

Applicable Section(s)	Description of Change(s)
Synopsis, Overview of Study Design; Time and Events Schedule (footnote c); Time and Events Schedule – Laboratory Test Schedule (footnote c); 3.1. Overview of Study Design; 9.1.1. Overview; 10.2. Discontinuation of Treatment	Clarified that EOMT assessments must be performed before initiation of any new anticancer treatment including radiotherapy.
**Rationale:** To provide updated information on administration with medicinal products known to prolong QT interval.	
8. Concomitant therapy	Text from the most recent SmPC was added to provide information on administration with medicinal products known to prolong QT interval.
References	Referenced the most recent versions of the Investigator's Brochure and SmPC.
**Rationale:** Minor errors were noted	
Throughout the protocol	Minor editorial changes and clarifications were made, including grammatical, formatting, or spelling changes.

Amendment INT-4 (27 November 2014)

This amendment is considered to be substantial based on the criteria set forth in Article 10(a) of Directive 2001/20/EC of the European Parliament and the Council of the European Union.

The overall reason for the amendment: To reference additional information on potential drug-drug interactions in the Investigator's Brochure and SmPC, and to clarify data analysis steps and other study procedures.

Applicable Section(s)	Description of Change(s)
**Rationale:** To reference additional information on potential drug-drug interactions in the Investigator's Brochure and SmPC.	
8. Concomitant Therapy; References	The Investigator's Brochure and SmPC were identified as sources of additional information on potential drug-drug interactions, including those related to CYP1A2, CYP2D6 and CYP2C8. The most recent edition of the Investigator's Brochure was referenced.
**Rationale:** To clarify the scope and timing of data analyses.	
11. Statistical Methods	Text was added to clarify that data will also be analyzed when EOMT assessments have been performed for all subjects (main analysis). Text was expanded to provide more information on data to be included in the analyses, and wording regarding the timing of the primary analysis was corrected and clarified.
**Rationale:** To describe dosing compliance procedures.	
7. Treatment Compliance	Text was added to describe dosing compliance procedures. The level of non-compliance to be defined as a major protocol violation was specified.
**Rationale:** To clarify the timing of EOMT assessments in subjects discontinuing the main study treatment period.	
Synopsis; Time and Events Schedule footnote c; Time and Events Schedule – Laboratory Test Schedule footnote c; 3.1. Overview of Study Design; 9.1.1. Overview (Study Procedures); 9.1.3. Open-Label Main Study Treatment Period; 10.2. Discontinuation of Treatment.	Text was added to clarify that, for subjects discontinuing the main study treatment period, EOMT assessments will be performed as close as possible to the time of discontinuation and before initiation of any new anticancer treatment.
**Rationale:** To clarify text on DXA procedures.	
9.2.1. Evaluations (Safety)	Text regarding DXA procedures was simplified.
**Rationale:** Minor errors were noted.	
Throughout the protocol	Minor editorial changes and clarifications were made.

Amendment INT-3 (22 April 2014)

This amendment is considered to be nonsubstantial based on the criteria set forth in Article 10(a) of Directive 2001/20/EC of the European Parliament and the Council of the European Union in that it does not significantly impact the safety or physical/mental integrity of subjects, nor the scientific value of the study.

The overall reason for the amendment: The overall reason for the amendment is to describe more comprehensively the data to be documented, to clarify the scheduling of assessments and to avoid repetition of assessments.

Applicable Section(s)	Description of Change(s)
**Rationale:** To avoid repetition of assessments previously performed within the timeframe specified in the protocol and to reduce the procedural/ blood sampling burden for subjects.	
Synopsis; Time and Events Schedule; Time and Events Schedule – Laboratory Test Schedule; 3.1.1. Assessments; 4. Subject Selection; 9.1.2. Screening Phase; 9.2.1. Evaluations (Safety); 9.3.1. Evaluations (Efficacy)	Stated that laboratory tests, ECG, MUGA/echocardiography, DXA, CT, MRI, and bone scan assessments performed according to standard of care prior to signature of the ICF can be used as screening assessments, and do not need to be repeated, provided these have been performed within the time interval, and according to the methods, specified in the protocol.
Time and Events Schedule (footnote r); 9.3.1. Evaluations (Efficacy)	For the EOMT assessment, stated that a complete tumor assessment does not need to be repeated if this has been performed within 8 weeks of the EOMT assessment, unless it is clinically indicated.
Time and Events Schedule	For clarity, added a separate row for bone scan assessments.
Time and Events Schedule – Laboratory Test Schedule (footnote l); 9.2.1. Evaluations (Safety)	Indicated that, unless clinically indicated, a laboratory test (local and/or central testing) does not need to be repeated at the EOMT assessments if testing was performed within 2 weeks of the EOMT assessment.
Time and Events Schedule – Laboratory Test Schedule (footnote n); 9.3.1. Evaluations (Efficacy)	Modified the PSA sampling schedule to every 12 weeks after Cycle 6, which is adequate for efficacy evaluation.
**Rationale:** To specify that any data on soft tissue progression as defined in the modified RECIST will be documented.	
Synopsis; 3.1.1. Assessments; 9.3.1. Evaluations; 9.3.2. Endpoints	Indicated that any evidence of soft tissue progression as defined in the modified RECIST, including assessments performed according to local clinical practice, will be documented in the eCRF during the EOMT assessment.

Applicable Section(s)	Description of Change(s)
**Rationale:** To correct the sampling schedule for serum androgen testing.	
Time and Events Schedule – Laboratory Test Schedule	Indicated that serum androgen testing will be performed at Cycles 24, 30 and 36.
**Rationale:** To state specifically that data on vitamin D use will be documented	
8. Concomitant Therapy	Stated that details of vitamin D use must be recorded in the eCRF.
**Rationale:** To clarify the definition of 2 antihypertensives if combination therapies are used.	
4.1. Inclusion Criteria, inclusion criterion 9.1	Added the clarification that any antihypertensive treatment containing 2 agents in combination is considered to be 2 antihypertensives.
**Rationale:** To distinguish more clearly monitoring as part of subjects' routine clinical care, and assessments repeated as clinically indicated, from protocol-specific assessments defined in the Time & Events Schedule.	
9.2.1. Evaluations (Safety)	Added more information on routine monitoring of serum transaminases and serum potassium per SmPC. Added information on routine monitoring of blood pressure per SmPC.
Time and Events Schedule – Laboratory Test Schedule	Removed details on routine clinical monitoring from Time & Events footnotes, for clarity.
9.2.1. Evaluations (Safety, Clinical Laboratory Tests)	Clarified that the results of any laboratory testing performed as part of subjects' routine monitoring per SmPC, which are not study-related assessments, will not be recorded routinely in the eCRF, unless clinically significant abnormalities that will be reported as AEs.
9.2.1. Evaluations (Safety)	Added statements to reiterate that: safety assessments can be performed more frequently than specified in the protocol if clinically indicated; clinically significant abnormalities, including laboratory test abnormalities, will be reported as AEs in the eCRF; all incidences of hypokalemia and of hypertension will be reported as AEs; unscheduled visits may be planned to assess, confirm and follow up clinically significant abnormalities.
Time and Events Schedule; Time and Events Schedule – Laboratory Test Schedule	Removed text and footnotes indicating that ECG, vital signs and serum K^+, Na^+, urea and creatinine assessments can be performed more frequently if clinically indicated, for clarity.
**Rationale:** To remove text indicating a fixed recruitment period.	
9.1.1. Overview	Deleted text indicating that the recruitment period will be exactly 1 year, since this is an approximate duration and will be flexible depending on subject recruitment rate.
**Rationale:** Minor errors were noted.	
Throughout the protocol	Minor editorial changes and clarifications were made, including grammatical, formatting, or spelling changes.

Amendment INT-2 (13 November 2013)

This amendment is considered to be substantial based on the criteria set forth in Article 10(a) of Directive 2001/20/EC of the European Parliament and the Council of the European Union.

The overall reason for the amendment: The overall reason for the amendment is to adjust inclusion/exclusion criteria and to clarify procedural aspects of the study.

Applicable Section(s)	Description of Change(s)
**Rationale:** To modify the inclusion/exclusion criteria, enabling patients with liver or lung visceral metastasis, and patients treated with a maximum of 2 antihypertensives, to participate in the study.	
Synopsis, Subject Selection; 3.1. Overview of Study Design; 4.1. Inclusion Criteria, inclusion criterion 3; 4.2. Exclusion Criteria, exclusion criterion 5	Modified text to indicate that subjects with liver or lung visceral metastasis are eligible for participation in the study.
4.1. Inclusion Criteria, inclusion criterion 9	Modified criterion to indicate that subjects treated with a maximum of 2 antihypertensives are eligible for study.
4.2. Exclusion Criteria, exclusion criterion 3	Modified criterion to exclude subjects who have received prior corticosteroid treatment for prostate cancer.
4.2. Exclusion Criteria, exclusion criterion 11	Modified criterion to enable participation of subjects who have previously received palliative radiotherapy for metastatic CRPC.
4.1. Inclusion Criteria, inclusion criterion 15; 4.3. Prohibitions and Restrictions	Amended text to state that a condom and another effective form of birth control must be used until 1 week after the last dose of study drug and that a condom must be used for sex with a woman who is pregnant.
**Rationale:** To clarify definitions of study completion and procedures associated with discontinuation.	
Synopsis, Overview of Study Design; 3.1. Overview of Study Design; Figure 3; 10.1. Completion	The definition of completion of the main study treatment period was clarified.

Applicable Section(s)	Description of Change(s)
Synopsis, Overview of Study Design; Time and Events Schedule; 3.1. Overview of Study Design; 9.1.1. Overview; 9.1.3. Open-Label Main Study Treatment Period	Defined the main study treatment period cut-off date and clarified the timing of EOMT assessments
Time and Events Schedule	Defined Day 1 of Cycle 39 assessments separately from EOMT assessments in the Time and Events Schedule.
9.1.4 Open-Label Extension Phase	Clarified that extension phase treatment dispensing will start at the time of EOMT assessments (at the end of the last cycle of main study treatment).
Synopsis, Overview of Study Design; Time and Events Schedule; 3.1. Overview of Study Design; Figure 3; 9.1.1. Overview; 9.1.3. Open-Label Main Study Treatment Period	Added text to clarify that if a progression-free subject does not enter the extension phase that the reason must be documented and an EOMS visit will be performed.
10.2. Discontinuation of Treatment	Added text to clarify that permanent changes to study treatment are a reason for discontinuation. “Stress doses” of glucocorticoid are allowed during the extension phase only.
3.1. Overview of Study Design	Added subsections and cross-references for clarity.
**Rationale:** To clarify concomitant therapy during the main study treatment period and extension phase	
8. Concomitant Therapy	Amended text to indicate that during the extension phase concomitant therapy should be according to the SmPC for abiraterone acetate.
8. Concomitant Therapy; 10.2. Discontinuation of Treatment	Added text stating that during the extension phase palliative radiation will be allowed, after the consultation of a multidisciplinary team and according to local guidelines.
6. Dosage and Administration; 8. Concomitant Therapy	Added text regarding “stress doses” of glucocorticoid.
10.2. Discontinuation of Treatment	Added the need for palliative radiation as a reason for discontinuation from the main study treatment period.
8. Concomitant Therapy; References	Added information on potential drug-drug interactions with CYP3A4 inducers and referenced the most recent edition of the Investigator’s Brochure.

Applicable Section(s)	Description of Change(s)
**Rationale:** To correct and clarify timing of study procedures.	
Synopsis, Overview of Study Design; Time and Events Schedule; 3.1. Overview of Study Design; Figure 2; 9.1.1. Overview; 9.1.2. Screening Phase; 9.3.1. Evaluations	Amended the duration of the screening period to 4 weeks (28 days) prior to randomization.
Time and Events Schedule; 9.1.1. Overview	Amended visit time windows after Cycle 6 to ±6 days.
Time and Events Schedule; 9.1.1. Overview; 9.1.2. Screening Phase; 9.2.1. Evaluations	Specified time windows for laboratory tests. Clarified timing of tests needed prior to randomization.
Time and Events Schedule	Created a separate schedule table for laboratory testing, for clarity.
Time and Events Schedule; 3.1. Overview of Study Design; 9.3.1. Evaluations	Added and clarified the timing of evaluation of response and best response.
Time and Events Schedule	Aligned dosing compliance assessments with study treatment dispenses.
Time and Events Schedule	Itemized measurement of height separately for clarity.
**Rationale:** To correct and clarify procedural information	
3.1. Overview of Study Design; 9.1.4. Open-Label Extension Phase	Clarified that medical monitoring during the extension phase will be performed according to the approved SmPCs.
Synopsis, Safety Evaluations; Time and Events Schedule; 3.1. Overview of Study Design; 9.1.2. Screening Phase; 9.2.1. Evaluations	Added "axillary temperature" to vital signs assessments.
Time and Events Schedule; 9.2.1. Evaluations	Amended text such that for positive dipstick on urinalysis, sediment will be evaluated according to local practice. Added that, for centers where dipstick results are not available, sediment will be evaluated according to local practice.

Applicable Section(s)	Description of Change(s)
9.2.1. Evaluations; 9.6. Sample Collection and Handling	Reiterated that the serum androgen/androgen precursor profile, urine full steroid excretion profile and biomarkers will be analyzed separately by central laboratory.
11.3. Safety Analyses	Added text to clarify that data analysis for serum/urinary androgens and their precursors will be performed externally.
11.3. Safety Analyses	Removed text on statistical analysis of ECG data, which is not applicable.
7. Treatment Compliance	Removed redundant text relating to ≤75% compliance.
5. Treatment Allocation and Blinding	Removed text on IVRS, which is not applicable.
9.4. Biomarkers	Corrected blood volumes collected for biomarker analysis.
14.4. Preparation, Handling and Storage	Amended storage instructions for the study medication to be as instructed by the manufacturer.
16.2.5. Long-Term Retention of Samples for Biomarker Research	Corrected text relating to storage of samples for biomarker research.
**Rationale:** To provide reference information	
9.2.1. Evaluations	Provided HOMA-IR formula.
Time and Events Schedule; 3.2. Study Design Rationale; 8. Concomitant Therapy; 9.1.1. Overview; 9.1.4. Open-Label Extension Phase; 9.2.1. Evaluations; References	Added references to prescribing information for abiraterone acetate, prednisone and dexamethasone.
1. Introduction; 1.1. Concurrent Glucocorticoids	Updated information on the number of subjects treated with abiraterone acetate in clinical studies, and the number of countries in which abiraterone acetate is approved, based on the current version of the Investigator's Brochure.
**Rationale:** Minor errors were noted	
Throughout the protocol	Minor editorial changes and clarifications were made, including grammatical, formatting, or spelling changes.

Amendment INT-1 (25 February 2013)

This amendment is considered to be nonsubstantial based on the criteria set forth in Article 10(a) of Directive 2001/20/EC of the European Parliament and the Council of the European Union, in that it does not significantly impact the safety or physical/mental integrity of subjects, or the scientific value of the study.

The overall reason for the amendment: To clarify the timing of events and assessments in the Time and Events Schedule, to capture in a more comprehensive way the schedule of assessments/events. To indicate that the biomarker research component of the study is optional and subject to separate informed consent. To further clarify study procedures where appropriate, including the procedure for dose increases of glucorticoids during the study.

Applicable Section(s)	Description of Change(s)
**Rationale:** To clarify the timing of assessments.	
Time and Events Schedule	For clarity, visit descriptors in the Time and Events Schedule were revised to refer only to Cycles and Days. Headings were amended to specify that assessments will be performed on Day 1 of cycles as applicable.<br>A dedicated Day 1, Cycle 2 column was created, removing a redundant Week 4, Cycle 1 column.<br>Footnote text on timing of biomarker samples, no longer needed, was removed.<br>A footnote was added to clarify that Day 1, Cycle 1 assessments will be performed before initiation of study treatment.<br>The timing of dispensing of study treatment was corrected and clarified using a footnote.<br>Footnotes were amended to clarify the visit windows for CT, MRI, and bone scans.<br>A dedicated row was added to clarify the timing of BPI-SF assessments, which will be performed at screening but not on Day 1, Cycle 1.<br>Medical resource utilization data collection at the Day 15, Cycle 1 visit was removed.
Synopsis; Time and Events Schedule; 3.1. Overview of Study Design; Figure 2; 4. Subject Selection; 6. Dosage and Administration; 7. Treatment Compliance; 8. Concomitant Therapy; 9 Study Evaluations	One or more of the following have been changed in the applicable sections:<br>The 'baseline' or 'Day 1' visit descriptor was replaced with the term 'Day 1, Cycle 1'. The timing of randomization relative to Day 1 was clarified. Text was amended to clarify that: written informed consent must be obtained within 3 weeks prior to Day 1, Cycle 1, before any study-related procedure; initiation of study treatment will occur on Day 1, Cycle 1 and that this must occur within 72 hours after randomization. 'Randomization' was removed from the Day 1 label of Figure 2.<br>Clarified activities scheduled to occur in the main study treatment period only or in the main study treatment period and the extension phase.
Time and Events Schedule; 4. Subject Selection; 9.1.3. Open-Label Main Study Treatment Period; 9.2.1 Evaluations	Footnotes or text were amended to clarify that hematology and urinalysis assessments and LVEF measurement will be performed at screening.
9.1.1. Overview; 9.1.2. Screening Phase	Visit windows were amended to refer to cycle number. Visit windows for scanning visits were specified.

Applicable Section(s)	Description of Change(s)
9.3.1. Evaluations	The timing of screening scans was clarified, consistent with the Time and Events Schedule footnotes.
9.4. Biomarkers	The text describing the timing of biomarker samples was revised to refer only to Cycles and Days, for clarity.<br>Added clarification on timing of processing and freezing of samples.
Synopsis; Time and Events Schedule; 3.1. Overview of Study Design; Figure 2; Figure 3; 9.1.1. Overview; 9.1.3. Open-Label Main Study Treatment Period; 10.1. Completion	For consistency, clarifications regarding 'Cycle 39' or '39 cycles' were added.
**Rationale:** To add detail on the timing of steroid doses in accordance with the Summary of Product Characteristics (SmPC).	
Synopsis; 6. Dosage and Administration	Text was amended to indicate that prednisone or dexamethasone tablets should be taken with or immediately after a meal.
**Rationale:** To indicate that the biomarker research component of the study is optional and subject to separate informed consent.	
Synopsis; Time and Events Schedule; 2.1. Objectives; 3. Study Design and Rationale; 4.1. Inclusion Criteria; 9.1.3. Open-Label Main Study Treatment Period; 9.4. Biomarkers; 10.3. Withdrawal From the Study; 16.2 Regulatory Ethics Compliance	Indicated that subject participation in the biomarker research phase is optional and subject to signing a separate ICF before Day 1, Cycle 1.<br>Provided guidance on options for the use/destruction of samples for the biomarker research phase for subjects who withdraw from this phase and/or from the main study.
**Rationale:** To clarify the storage of optional blood and plasma bioresearch samples collected during the study.	
9.4. Biomarkers 16.2.5. Long-Term Retention of Samples for Biomarker Research	Clarified that blood and plasma samples collected during the study for biomarker research will be stored only until biomarker analysis has been completed.

Applicable Section(s)	Description of Change(s)
**Rationale:** To clarify the procedure for dose increases of prednisone or dexamethasone.	
6. Dosage and Administration; 8. Concomitant Therapy; 9.2.1. Safety Evaluations; 10.2. Discontinuations of Treatment; 16.1. Study-Specific Design Considerations.	Clarified that subjects who receive a “stress dose” of glucocorticoid will be discontinued from the main study, but will have the option of continuing study treatment in the extension phase.<br>Removed additional systemic glucocorticoid administration from the list of prohibited therapies
**Rationale:** To include information on the (safety) monitoring of subjects during the extension phase.	
Synopsis; Time and Events Schedule; 9. Study Evaluations	Indicated that subjects will be monitored according to the approved SmPC during the extension phase.
**Rationale:** To clarify text on discontinuation.	
10.1. Completion; 10.2. Discontinuation of treatment; 10.3. Withdrawal From the Study	Subheadings and text were amended to emphasize where text is applicable to both the main study and extension phase or applicable to the main study only. An unnecessary paragraph was removed for clarity.
**Rationale:** To update the text on product quality complaint (PQC) reporting to align with company policy.	
13.1. Procedures	The text specifying reporting time for initial PQCs was changed from "as soon as possible" to "within 24 hours".
**Rationale**: To specify that women who are pregnant or may be pregnant should not handle abiraterone acetate without protection, in accordance with the SmPC.	
14.4. Preparation, Handling, and Storage	Text was added to state that women who are pregnant or may be pregnant should not handle abiraterone acetate without protection, eg, gloves.
**Rationale:** To clarify the information that will be provided to investigators.	
15. Study-specific materials; Attachment 1	Removed ‘pharmacy manual’ (site investigational product manual only to be provided) and ‘IVRS manual’. Clarified that NCI CTCAE and RECIST are provided as attachments to protocol. Amended information and provided reference for RECIST criteria in Attachment 1.
**Rationale:** To update the text on use of information and publication to align with company policy.	
17.11. Use of Information and Publication	Minor amendments were made to the text on use of information and publication to align with company policy.
**Rationale:** To update the text regarding approval of the Type II variation.	
Synopsis; 1. Introduction; 1.1. Concurrent Glucocorticoids	The text was updated to state the date of approval of the Type II variation.

Applicable Section(s)	Description of Change(s)
**Rationale:** Minor errors were noted.	
Throughout the protocol	Minor grammatical, formatting, or spelling changes were made.

SYNOPSIS

PROTOCOL NUMBER: 212082-PCR-2023

EUDRACT NUMBER: 2012-004331-23

TITLE

A Randomized Phase 2 Study Evaluating Abiraterone Acetate With Different Steroid Regimens for Preventing Symptoms Associated With Mineralocorticoid Excess in Asymptomatic, Chemotherapy-Naïve and Metastatic Castration-Resistant Prostate Cancer (mCRPC) Patients

COMPOUND

Abiraterone acetate is a prodrug of abiraterone. Abiraterone is a selective irreversible inhibitor of 17α hydroxylase/$C_{17,20}$-lyase (cytochrome P450C17 [CYP17]). Inhibition of CYP17 inhibits the conversion of pregnenolone or progesterone into dehydroepiandrosterone (DHEA) or androstenedione, respectively, which are 2 precursors of testosterone in the adrenal glands and testes. CYP17 inhibition also results in increased mineralocorticoid production by the adrenal glands. Abiraterone acetate is indicated with prednisone or prednisolone for the treatment of metastatic castration-resistant prostate cancer (mCRPC) in adult men whose disease has progressed on or after a docetaxel-based chemotherapy regimen. A Type II variation, to extend the indication to include the treatment of metastatic castration resistant prostate cancer in adult men who are asymptomatic or mildly symptomatic after failure of androgen deprivation therapy in whom chemotherapy is not yet clinically indicated, was approved on 18 December 2012.

RATIONALE

The aim of this Phase 2 study is to evaluate alternative steroid treatment strategies for preventing symptoms associated with mineralocorticoid excess and minimizing toxicity from long-term glucocorticoid use in subjects treated with abiraterone acetate for asymptomatic and chemotherapy-naïve mCRPC.

OBJECTIVES AND HYPOTHESIS

Primary Objective

The primary objective is to evaluate the safety of abiraterone acetate with 4 alternative steroid treatment strategies related to symptoms associated with mineralocorticoid excess toxicities (ie, hypokalemia and/or hypertension) during the first 24 weeks of treatment in asymptomatic, chemotherapy-naïve, mCRPC subjects.

Secondary Objectives

The following secondary objectives will be assessed <u>during the entire study</u> in asymptomatic, chemotherapy-naïve and mCRPC subjects treated with abiraterone acetate and 4 alternative steroid treatment strategies:

- To further characterize the global safety profile (including the incidence of mineralocorticoid excess toxicities [eg, hypokalemia and hypertension]).
- To characterize mid-term and long-term exogenous glucocorticoid side effects.
- To characterize the clinical benefit.

- To evaluate the impact on pain and quality of life (QoL) as measured by the EQ-5D-5L, Brief Pain Inventory - short form (BPI-SF) and Functional Assessment of Cancer Therapy - Prostate Cancer (FACT-P) tools.
- To collect medical resource utilization (MRU) data that may be used in future economic modeling (the construction and reporting of the economic model will be conducted separately from this study).
- To evaluate overall survival.
- To collect data on subsequent therapies for prostate cancer (time to next therapy for prostate cancer, time to initiation of chemotherapy, treatment duration, best response) following cessation of study treatment.

Additionally, the study includes optional biomarker analyses of circulating genetic material as a secondary exploratory objective to:

- Identify genomic aberrations in circulating plasma DNA, and evaluate expression of circulating plasma and whole blood mRNA and microRNA (miRNA) occurring pre- and on-treatment.
- Analyze circulating biomarkers as predictors of response or sensitivity to abiraterone acetate or for identifying mechanisms of resistance.

Hypothesis

It will be determined whether the 4 treatment arms warrant further study based on the "no mineralocorticoid excess" rate. “No mineralocorticoid excess” is defined as experiencing (yes or no) neither of the 2 mineralocorticoid excess toxicities, ie, neither hypokalemia nor hypertension, during the first 24 weeks of treatment. The highest percentage of subjects experiencing neither hypokalemia nor hypertension during the first 24 weeks at which it is required to reject the treatment is 50% and the treatment would be worth developing further if this rate is 75%.

OVERVIEW OF STUDY DESIGN

This is a randomized, open-label, parallel-arm, multicenter, Phase 2 study of treatment with abiraterone acetate and 4 alternative steroid treatment strategies in asymptomatic, chemotherapy-naïve, mCRPC subjects.

A target of 144 subjects will be enrolled in this study with 36 subjects planned per treatment arm. Subjects will be randomized in a 1:1:1:1 ratio to receive abiraterone acetate with either prednisone 5 mg twice daily (Arm 1), prednisone 5 mg once daily (Arm 2), prednisone 2.5 mg twice daily (Arm 3) or dexamethasone 0.5 mg once daily (Arm 4). Abiraterone acetate will be taken as oral tablets at a once-daily dose of 1,000 mg. Abiraterone acetate, prednisone and dexamethasone will be considered as study drugs.

The main study will consist of a screening phase of 4 weeks followed by an open-label treatment period of a maximum of 39 cycles (156 weeks). The main study treatment period cut-off date will be in July 2016 (156 weeks after the start of study treatment for the first subject participating in the study). Subjects will participate in the main study treatment period until the cut-off date, and will receive study treatment until radiographic disease progression and/or unequivocal clinical progression and/or other specific reasons for discontinuation of treatment as per protocol. End-of-main-study-treatment (EOMT) assessments will be performed for all subjects randomized into the main study treatment period, regardless of whether they complete study treatment up to the cut-off date or discontinue from the main study treatment period before the cut-off date. For subjects discontinuing from the main study treatment period, EOMT assessments will be performed as close as possible to the time of discontinuation and before initiation of any new anticancer treatment, including radiotherapy. Additionally, for subjects discontinuing study treatment before the cut-off date, an end-of-main-study (EOMS) visit will be performed 4 weeks after the cessation of study medication.

All subjects must sign a written informed consent form (ICF) before any study-specific procedure is performed. Each subject consenting to participate in the optional biomarker research phase must also sign a separate ICF before Day 1, Cycle 1, to indicate that he agrees to provide optional blood samples for biomarker research (where local regulations permit). However, laboratory tests, Multiple Gated Acquisition (MUGA)/echocardiography, dual-energy X-ray absorptiometry (DXA), computed tomography (CT), magnetic resonance imaging (MRI), and bone scan assessments performed according to standard of care prior to signature of the ICF can be used as screening assessments, and do not need to be repeated, provided these have been performed within the time interval, and according to the methods, specified in the protocol. Subjects eligible for the study at screening will be randomized to a treatment arm and study medication will be initiated within 72 hours (3 calendar days) of randomization.

The primary parameter is defined as experiencing (yes or no) neither of the 2 mineralocorticoid excess toxicities during the first 24 weeks of treatment, ie, neither hypokalemia nor hypertension. Secondary safety parameters will assess the global safety profile (including the incidence of mineralocorticoid excess toxicities [eg, hypokalemia and hypertension], and the incidence of mid-term and long-term glucocorticoid side effects), according to the National Cancer Institute Common Terminology Criteria for Adverse Events (NCI CTCAE) v4.0, during the entire study. All events will be collected from signing of the ICF onwards.

Safety and tolerability, including monitoring of adverse events (AEs) and serious adverse events (SAEs), clinical laboratory tests, vital signs, DXA scans, and physical examination will be assessed at scheduled visits.

A 12-lead electrocardiogram (ECG) will be performed at screening and can be repeated as clinically indicated. An ECG performed up to 28 days prior to Day 1, Cycle 1 but before the subject has signed the ICF can be used as the screening assessment.

Efficacy assessments will include progression-free survival (PFS) (based on radiographic PFS [rPFS], clinical progression or death) and survival. The assessment of rPFS will utilize sequential imaging studies as defined by Prostate Cancer Working Group 2 (PCWG2) and modified response evaluation criteria in solid tumors (modified RECIST, v1.1). Prostate specific antigen (PSA) values will be collected throughout the study to assess PSA response rate and time to PSA progression. Any evidence of soft tissue progression as defined in the modified RECIST will be documented. Time to opiate use for cancer-related pain will be prospectively assessed. Eastern Cooperative Oncology Group (ECOG) performance status will be evaluated to assess time to first deterioration.

Pain and QoL will be monitored by administration of EQ-5D-5L, BPI-SF and FACT-P at scheduled visits or if disease progression is suspected.

Optional blood samples will be collected at predefined time points for biomarker analyses in whole blood and plasma. Medical resource utilization data associated with medical encounters will also be collected.

Subjects who discontinue study treatment due to disease progression, or initiation of another prostate cancer treatment, or due to safety reasons, or at the subject's request, at any time during the study will enter a follow-up phase. These subjects will be followed up for survival and for subsequent prostate cancer therapies until July 2018 (5 years after the start of study treatment of the first subject participating in the study).

Subjects who require diuretic treatment or a change in glucocorticoid dose, or who experience grade 3 or grade 4 hypokalemia will be discontinued from the main study in the absence of disease progression. However, if toxicities related to the conditions described above have recovered to ≤grade 1, these subjects will have the option of entering an extension phase, allowing continuation of their study treatment until radiographic disease progression and/or unequivocal clinical progression and/or other specific reasons for discontinuation as per protocol, or death, if earlier. Similarly, subjects who are progression-free at the cut-off date will be able to enter the extension phase of the study and continue study treatment until

radiographic disease progression and/or unequivocal clinical progression and/or other specific reasons for discontinuation. If a progression-free subject does not enter the extension phase of the study, the reason for this must be documented during the EOMT assessment and an EOMS visit will be performed 4 weeks after the last study treatment.

During the extension phase, as part of routine clinical care, subjects should be monitored according to the approved Summaries of Product Characteristics (SmPCs); (S)AEs and survival information will be collected. Following disease progression or discontinuation of extension study treatment for any other reason, an end-of-extension-treatment (EOE) visit will be performed. Survival and subsequent prostate cancer therapies will be monitored during the follow-up phase until July 2018 (5 years after the start of study treatment of the first subject participating in the study).

The main study treatment period will be considered complete at the cut-off date in July 2016, ie, 156 weeks (equivalent to a maximum of 39 study treatment cycles) after the start of study treatment for the first subject participating in the study. Therefore, each subject will participate in the main study treatment period for a maximum of 156 weeks, depending on the time of enrollment. With the extension and follow-up phases, the entire study will be complete in July 2018 (5 years after the start of study treatment of the first subject participating in the study).

A Scientific Advisory Committee will be commissioned to ensure scientific validity of this study, to identify any scientifically relevant trends, and to provide recommendations to the sponsor.

SUBJECT SELECTION

The subject population will comprise medically or surgically castrated male patients of 18 years of age or older with mCRPC who have shown tumor progression, are chemotherapy-naïve, and are asymptomatic by prospectively defined criteria. Eligible subjects should have metastatic disease documented by positive bone scan or by CT or MRI.

DOSAGE AND ADMINISTRATION

Abiraterone acetate 1,000 mg (four 250-mg tablets) will be taken orally once daily continuously at least 2 hours after eating and no food should be eaten for at least 1 hour after taking the tablets. Oral prednisone will be given twice daily in treatment arm 1 (one 5-mg tablet per dose) and treatment arm 3 (one 2.5-mg tablet per dose): the first dose in the morning with or immediately after a meal and the second dose after a minimum interval of 8 hours in the late afternoon or early evening, with or immediately after a meal. In treatment arm 2, oral prednisone (one 5-mg tablet) will be given once daily in the morning with or immediately after a meal. In treatment arm 4, dexamethasone (one 0.5-mg tablet) will be taken as a single dose in the morning, with or immediately after breakfast. Tablets should be swallowed whole with water.

Subjects already treated with osteoprotective agents prior to the study can continue their treatment. According to the original protocol, if treatment with an osteoprotective agent was started, it must have been started prior to Day 1 of Cycle 1, with the choice of the osteoprotective agent at the investigator's discretion according to local clinical practice and the current product label. Per protocol amendment INT-5, initiation of bisphosphonates or other approved osteoprotective agents during the study will be allowed after appropriate documentation of the bone loss, and according to local clinical practice and the current product label.

SAFETY EVALUATIONS

Safety and tolerability will be evaluated throughout the study (see the Time and Events Schedule for details). Safety evaluations include monitoring of (S)AEs, clinical laboratory tests (hematology, chemistry, serum androgen/androgen precursors profile, urine full steroid excretion profile, homeostatic model assessment to quantify insulin resistance (HOMA-IR), adrenocorticotrophic hormone [ACTH]), vital signs (including seated blood pressure, heart rate, respiratory rate, and oral, or axillary or tympanic

body temperature), physical examinations, body weight and DXA scans (to assess water retention, bone mineral density, lean body mass and body fat distribution).

Mineralocorticoid excess toxicities (eg, hypokalemia, hypertension, fluid retention) will be assessed by blood pressure measurements, laboratory assessments (including serum sodium, potassium, urea and creatinine), and physical examination. In addition, DXA scans will be used to measure the level of water retention in the body; whole-body DXA scans can measure small differences between treatments in the amount of fluid going into the extravascular system. The impact of study treatment on adrenal function will be assessed by measurement of plasma ACTH and plasma and urinary androgens and their precursors (dehydroepiandrosterone [DHEA], dehydroepiandrosterone-sulphate [DHEA-S], androstenedione, testosterone and 5α-dihydrotestosterone).

Endocrine and metabolic glucocorticoid side effects (eg, impaired carbohydrate tolerance, cushingoid facies and weight gain) will be assessed by laboratory assessments (including fasting glucose, insulin, hemoglobin A1c (HbA1c), C-reactive protein, serum lipids), and body weight recording. Fasting glucose, insulin (from which HOMA-IR will be calculated) and HbA1c measurements will demonstrate the impact of treatment on insulin resistance. Additionally, DXA scans will be evaluated for redistribution of visceral or subcutaneous fat and sarcopenia (decrease in skeletal muscle or lean body mass) as a side effect of steroid excess.

DXA scans (or other radiological methods as per local guidelines) will also be used at predefined time points to assess musculoskeletal adverse effects including osteoporosis, tendon rupture, and vertebral and long bone fractures. Symptoms concerning fluid and electrolyte disturbance (hypertension, hypokalemia, sodium and water retention) following mid-term and long-term steroid use will be assessed by blood pressure measurements, laboratory assessments and physical examination. Infections, gastrointestinal symptoms (eg, nausea, increased appetite, peptic ulceration) and neuropsychiatric symptoms (eg, irritability, sleep disturbances) associated with the use of steroids will be assessed by (S)AE reporting.

Safety Endpoints

Primary: "No mineralocorticoid excess" is defined as experiencing (yes or no) neither of the 2 mineralocorticoid excess toxicities, ie, neither hypokalemia nor hypertension, during the first 24 weeks of treatment.

Secondary: Global safety profile according to NCI CTCAE, Version 4.0, including the incidence of mineralocorticoid excess toxicities (eg, hypokalemia and hypertension), and incidence of mid-term and long-term glucocorticoid side effects during the entire study.

EFFICACY EVALUATIONS

Efficacy evaluations will be performed as specified in the Time and Events Schedule and will include the ECOG Performance Status, PSA tests and radiographic assessments.

Patient reported outcomes (PROs) will be measured utilizing the BPI-SF, EQ-5D-5L and FACT-P and data will be collected throughout the study at scheduled visits.

Efficacy Endpoints

- PFS (based on radiographic progression, clinical progression or death)
- PSA response rate (by PCWG2 criteria)
- Time to PSA progression (by PCWG2 criteria)
- Objective response rate in subjects with measurable disease (modified RECIST, v1.1)
- Time to opiate use for cancer pain

- Time to deterioration in ECOG performance score by 1 point
- Change in prostate cancer-related PRO measures: EQ-5D-5L, BPI-SF, FACT-P
- Overall survival
- Subsequent anticancer therapies (time to next therapy for prostate cancer, time to initiation of chemotherapy, treatment duration and best response) until death or study close

BIOMARKER EVALUATIONS

Circulating plasma DNA and RNA and whole blood RNA will be evaluated for subjects participating in the optional biomarker research prior to starting, during, and upon discontinuation of study treatment. Tumor genomic aberrations and microRNA (miRNA) and mRNA profiles will be investigated. The association of these molecular studies with response or resistance to abiraterone acetate will be explored.

MEDICAL RESOURCE UTILIZATION

Medical resource utilization data associated with medical encounters will be collected in the electronic case report form (eCRF) by the investigator and staff for all subjects throughout the study.

STATISTICAL METHODS

All efficacy and safety analyses will be performed on the intent-to-treat (ITT) population, which will include all randomized subjects regardless of whether they received any study treatment. Additionally, where appropriate, the safety analysis will be performed including only randomized subjects who receive at least 1 dose of abiraterone acetate plus prednisone or dexamethasone and have at least 1 safety assessment after Day 1, Cycle 1 (modified intent-to-treat [mITT] analysis set).

Sample Size Determination

A sample size of approximately 144 subjects is planned.

The COU-AA-302 study indicated that 81% of subjects did not experience hypokalemia and/or hypertension within 6 months of the study start. For the present study, the assumption is that 75% of the subjects within each treatment arm will not experience either of the events defined in the primary parameter, ie, hypokalemia, and/or hypertension, during 24 weeks of treatment. Fifty percent is defined as the highest percentage to reject the treatment. An exact binomial test with a 5% one-sided significance level will have 89% power to detect the difference between 50% and 75% when the sample size is 30. Assuming 15% of non-evaluable subjects, 36 subjects per treatment arm will be included.

Safety Analyses

The number and percentage of subjects with neither of the 2 mineralocorticoid excess toxicities (ie, hypokalemia and/or hypertension) during the first 24 weeks of treatment will be summarized descriptively per treatment group and corresponding 95% confidence intervals (CIs) will be displayed.

For each (S)AE, the percentage of subjects who experience at least one occurrence of the given event during the study will be summarized by treatment arm.

Laboratory data will be summarized by type of laboratory test. Descriptive statistics will be calculated for each laboratory analyte at baseline and at each scheduled time point. Changes from baseline results will be presented in pre- versus post-treatment cross-tabulations. The laboratory data for subjects with any post-baseline result outside the reference range will be summarized, when appropriate, by use of the NCI CTCAE Grade, Version 4.0.

The effects on cardiovascular variables will be evaluated by means of descriptive statistics and frequency tabulations. These tables will include observed values and changes from baseline values to allow detection of clinically relevant changes in individuals.

Descriptive statistics of pulse and blood pressure (systolic and diastolic) values and changes from baseline will be summarized at each scheduled time point. The percentage of subjects with values beyond clinically important limits will be summarized.

For the physical examination analyses, descriptive statistics of changes from baseline will be summarized at each scheduled time point.

Efficacy Analyses

Changes from baseline and observed values for continuous/ordinal efficacy data will be summarized descriptively at each assessment time point during the study and at the subject's last efficacy evaluation (endpoint). Summary tabulations will be presented that will display the number of observations, mean, standard deviation, median, minimum and maximum and 95% CIs by treatment arm.

Time-to-event type of analyses (eg, PFS, time to PSA progression) will be performed using standard survival analysis methods including Kaplan-Meier product-limit survival curve estimates. Results will be summarized by 25th, 50th (median) and 75th percentiles with associated 2-sided 95% CIs, as well as percent of censored observations by treatment arm.

For categorical variables (eg, PSA response rate, objective response in subjects with measurable disease), the number and percent per category will be summarized by treatment.

No between-group comparisons will be performed.

Extension Phase and Follow-up Phase Analyses

Data from the extension phase and follow-up phase will be analyzed descriptively. Overall survival, time to next therapy for prostate cancer and time to initiation of chemotherapy will be analyzed using standard survival analysis methods including Kaplan-Meier product-limit survival curve estimates. The duration of the next anticancer therapy and the corresponding best response will be summarized descriptively.

Biomarker Analyses

Analyses of biomarker data will be conducted externally.

Medical Resource Utilization Analyses

For the MRU and health economics data, descriptive statistics for changes from baseline to each assessment will be presented by treatment arm.

TIME AND EVENTS SCHEDULE

	Screen[a]	Main Study Treatment Period (Until Cut-Off Date)												EOMS or EOE[d]	Extension[e]	Follow-up[e]
**Cycle (28 days)**		1		2	3	4, 5	6	9,15	12	18	21, 27, 33, 39	24, 30, 36	EOMT[c]			
**Cycle day (±2 days up to Cycle 6 inc.; ±6 days after Cycle 6)**	-28 to 0	1[b]	15	1	1	1	1	1	1	1	1	1				
**Study Procedure**																
**Screening/Administrative**																
Informed consent (ICF)[f]	X															
Informed consent for optional biomarker research phase[f]	X															
Inclusion/exclusion criteria	X															
Medical history/demographics[g]	X															
Height	X															
Prestudy anticancer therapy	X															
Prostate cancer characteristics	X															
MUGA or echocardiography[h]	X															
Randomization[i]		X														
**Laboratory Tests**																
	See laboratory test schedule															
**Study Drug Administration**																
Dispense study drug[j]		X[i]			X		X	X	X	X	X	X			X[k]	
Dosing compliance					X		X	X	X	X	X	X	X		X	
**Safety Assessments**																
Adverse events[l]	———	———	———	———	———	———	———	———	———	———	———	———	———	———	——→	
DXA scan	X[m]				X		X		X				X			
Vital signs[n]	X	X	X	X	X	X	X	X	X	X	X	X	X	X		
12-lead ECG[o]	X															
Physical examination[p]/body weight/fluid retention/edema	X	X	X	X	X	X	X	X	X	X	X	X	X	X		
**Efficacy Assessments**																
Clinical examination and performance status (ECOG)	X	X	X	X	X	X	X	X	X	X	X	X	X	X		
CT, MRI[q]	X[g]				X		X	X	X	X	X	X	X[r]			
Bone scan[q]	X[g]				X		X	X	X	X	X	X	X[r]			
Evaluation of response					X		X		X	X	X	X	X			
Best response[s]														X		X

	Screen[a]	Main Study Treatment Period (Until Cut-Off Date)												EOMS or EOE[d]	Extension[e]	Follow-up[e]
**Cycle (28 days)**		1		2	3	4, 5	6	9,15	12	18	21, 27, 33, 39	24, 30, 36				
**Cycle day (±2 days up to Cycle 6 inc.; ±6 days after Cycle 6)**	-28 to 0	1[b]	15	1	1	1	1	1	1	1	1	1	EOMT[c]			
**Study Procedure**																
EQ-5D-5L, FACT-P[t]		X					X			X			X			
BPI-SF[t]	X						X			X			X			
**Biomarkers (optional[f])**																
Blood sampling	See laboratory test schedule															
**Ongoing Subject Review**																
Concomitant therapy[l, u]	→	→	→	→	→	→	→	→	→	→	→	→	→	→	→	
Survival status[v]	→	→	→	→	→	→	→	→	→	→	→	→	→	→	→	X
Next prostate cancer therapies																X
**Other Assessments**																
Medical resource utilization	X	X		X	X	X	X	X	X	X	X	X	X	X		

a Screening period within 28 days prior to randomization.

b Day 1, Cycle 1 assessments performed prior to initiation of study treatment.

c End-of-main-study-treatment (EOMT) assessments will be performed for all subjects randomized into the main study treatment period, regardless of whether they complete study treatment up to the cut-off date (July 2016; 156 weeks after the start of treatment of the first subject participating in the study) or discontinue from the main study treatment period before the cut-off date. For subjects discontinuing from the main study treatment period, EOMT assessments will be performed as close as possible to the time of discontinuation and before initiation of any new anticancer treatment, including radiotherapy. For subjects who have completed the main study treatment period, EOMT assessments will be performed 4 weeks after Day 1 of the current cycle (ie, at the end of the cycle), and at the latest by 16 July 2016±15 days. For all subjects entering the extension phase, the day of EOMT assessments will be Day 1 of extension Cycle 1.

d An end-of-main-study (EOMS) or end-of-extension-treatment (EOE) safety assessment should be performed for each subject 4 weeks after the cessation of study medication. For subjects stopping treatment during the extension phase (EOE), [S]AEs, best response to abiraterone acetate, and survival will be assessed.

e Extension: Extension phase enabling continuation of study treatment in progression-free subjects who have discontinued or completed main study treatment, until radiographic disease progression and/or unequivocal clinical progression and/or other specific reasons for discontinuation as detailed in Section 10; visits every 12 weeks for assessment of [S]AEs and survival. During the extension phase, as part of routine clinical care, subjects should be monitored according to the approved SmPCs for abiraterone acetate[72], prednisone[48] and dexamethasone[16]. If a progression-free subject does not enter the extension phase of the study, the reason for this must be documented during the EOMT assessment and an EOMS visit will be performed 4 weeks after the last study treatment.
Follow-up: Follow-up after discontinuation of study treatment for any reason including disease progression (other than withdrawal of consent), at any time during the study, all subjects will be followed up for survival status and subsequent prostate cancer therapy (by chart review and 6-monthly telephone contact) until July 2018 (5 years after the start of study treatment of the first subject participating in the study).

f Written informed consent for the main study to be obtained within 28 days prior to Day 1, Cycle 1, before any study-related procedure; written informed consent for the optional biomarker research phase in the main study to be obtained within 28 days prior to Day 1, Cycle 1, before the first blood sample for biomarker analyses.

g Including a tumor assessment (with CT or MRI of the chest, abdomen and pelvis and a bone scan) performed up to 28 days prior to Day 1, Cycle 1. Assessments performed according to standard of care, and according to the methods specified in the protocol, up to 28 days prior to Day 1, Cycle 1 but before the subject has signed the ICF can be used as screening assessments.

[h] Left ventricular ejection fraction (LVEF) must be ≥50% for study eligibility. LVEF data will be obtained by Multiple Gated Acquisition scan (MUGA) or echocardiography if MUGA is not possible, or if echocardiography is the local standard of care. Assessments performed according to standard of care up to 28 days prior to Day 1, Cycle 1 but before the subject has signed the ICF can be used as screening assessments.

[i] Initiation of study treatment on Day 1, Cycle 1 must occur within 72 hours (3 calendar days) after randomization.

[j] Administration of 1,000 mg abiraterone acetate once daily, continuously + steroid regimen (prednisone/dexamethasone) according to randomized arm.

[k] Extension phase treatment will be dispensed at the time of the EOMT assessment (at the end of main study treatment) and thereafter on Day 1 of a cycle every 3 extension cycles.

[l] Will be monitored continuously from signing of the ICF onwards until the EOMS or EOE visit.

[m] DXA scans performed according to standard of care, and according to the methods specified in the protocol, up to 28 days prior to Day 1, Cycle 1, but before the subject has signed the ICF can be used as screening assessments.

[n] Including seated blood pressure, heart rate, respiratory rate, and oral, or axillary, or tympanic body temperature.

[o] Subjects should rest in a supine position for at least 5 minutes before electrocardiogram (ECG) collection. An ECG performed up to 28 days prior to Day 1, Cycle 1 but before the subject has signed the ICF can be used as the screening assessment.

[p] All examinations will include assessment of signs of fluid retention/edema and measurement of body weight.

[q] Radiographic disease to be measured using the same modality (CT or MRI, bone scan). Imaging visits may occur up to 8 days before due Cycle number visit. Subjects should return for other procedures and dispensing of study treatment on due Cycle number visits.

[r] For the EOMT assessment, a complete tumor assessment does not need to be repeated if this has been performed within 8 weeks of the EOMT assessment, unless it is clinically indicated.

[s] Best response to study treatment recorded at the EOE visit. Best response to each subsequent therapy recorded during follow-up.

[t] PROs (EQ-5D-5L, FACT-P and BPI-SF) to be done before any other visit procedure, at the scheduled time points or if disease progression is suspected.

[u] All therapies will be documented in the eCRF. Initiation of bisphosphonates or other approved osteoprotective agents during the study will be allowed after appropriate documentation of the bone loss, and according to local clinical practice and the current product label. During the extension phase, palliative radiotherapy will be allowed after consultation of a multidisciplinary team and according to local guidelines.

[v] Survival data will be collected throughout the main study treatment period and extension phase. During the follow-up phase, all subjects will be followed up for survival status (by chart review and 6-monthly telephone contact) until July 2018 (5 years after the start of study treatment of the first subject participating in the study).

TIME AND EVENTS SCHEDULE – LABORATORY TEST SCHEDULE

	Screen[a]	Main Study Treatment Period (Until Cut-Off Date)												EOMS or EOE[d]	Extension[e]	Follow-up
**Cycle (28 days)**	Day -14 to 0	1		2	3	4, 5	6	9,15	12	18	21, 27, 33, 39	24, 30, 36	EOMT[c,l]			
**Cycle day (±2 days up to Cycle 6 inc.; ±6 days after Cycle 6)**		1[b]	15	1	1	1	1	1	1	1	1	1				
**Local Testing – test results available on the day of the visit[f]**																
Hematology[g]	X[k]															
Urinalysis[h]	X[k]															
Serum testosterone[i]	X[k]															
AST, ALT, ALP, total bilirubin calcium, LDH, albumin, total protein and CRP[j]	X[k]			X	X		X		X	X			X[l]			
Serum K+, Na+, urea, creatinine	X[k]		X	X	X	X	X	X	X	X	X	X	X[l]	X		
Fasting plasma glucose, fasting serum insulin, and hemoglobin A1c (HbA1c)	X[k]			X	X		X		X	X			X[l]			
Fasting serum lipids[m]	X[k]			X	X		X		X	X			X[l]			
PSA[n]	X[k]			X	X	X	X	X	X	X	X	X	X[l]			
Plasma ACTH	X[k]				X											
**Central Testing – sampling at the time of the visit**																
**Serum and Urine testing**																
Serum androgens[o]		X		X	X		X		X	X		X	X[l]			
Urinary steroid excretion profile[p]		X			X		X		X	X		X	X[l]			
**Biomarkers (optional)**																
Blood sampling[q]		X		X									X			

[a] Screening laboratory tests within 14 days prior to randomization. Laboratory assessments performed according to standard of care within 14 days prior to randomization but before the subject has signed the ICF can be used as screening assessments.

[b] Day 1, Cycle 1 assessments performed prior to initiation of study treatment.

[c] End-of-main-study-treatment (EOMT) assessments will be performed for all subjects randomized into the main study treatment period, regardless of whether they complete study treatment up to the cut-off date (July 2016; 156 weeks after the start of treatment of the first subject participating in the study) or discontinue from the main study treatment period before the cut-off date. For subjects discontinuing from the main study treatment period, EOMT assessments will be performed as close as possible to the time of discontinuation and before initiation of any new anticancer treatment, including radiotherapy. For subjects who have completed the main study treatment period, EOMT assessments will be performed 4 weeks after Day 1 of the current cycle (ie, at the end of the cycle) and at the latest by 16 July 2016±15 days. For all subjects entering the extension phase, the day of EOMT assessments will be Day 1 of extension Cycle 1.

[d] An end-of-main-study (EOMS) or end-of-extension-treatment (EOE) safety assessment should be performed for each subject 4 weeks after the cessation of study medication.
[e] During the extension phase, as part of routine clinical care, subjects should be monitored according to the approved SmPCs for abiraterone acetate[72], prednisone[48] and dexamethasone.[16]
[f] During the main study treatment period, blood samples for laboratory testing must be taken ≤4 days prior to each visit.
[g] Including coagulation factors, prothrombin time, PTT and INR
[h] Dipstick for blood, protein and glucose. If the urinalysis dipstick result is abnormal, or not available, sediment will be evaluated according to local practice.
[i] Confirmation of testosterone levels <50 ng/dL (<2.0 nM).
[j] C-reactive protein: CRP.
[k] The results of screening laboratory tests must be available prior to randomization.
[l] For the EOMT assessments, a laboratory test (local and/or central testing) does not need to be repeated, unless clinically indicated, if testing was performed within 2 weeks of the EOMT assessment.
[m] Fasting serum lipids include cholesterol, triglycerides, high-density lipoprotein and low-density lipoprotein.
[n] Blood samples for PSA testing will be obtained every 4 weeks up to and including Cycle 6 and every 12 weeks thereafter during the main study treatment period.
[o] DHEA, dihydrotestosterone, testosterone, DHEA-S and androstenedione. Frozen aliquots will be sent in batches to a central laboratory.
[p] 24-hour urine assessments of mineralocorticoids and their precursor steroids. Note: total urine volume to be recorded prior to taking aliquots. Frozen aliquots will be sent in batches to a central laboratory.
[q] Optional blood samples collected for biomarker analyses in whole blood and plasma.

ABBREVIATIONS

ACTH	adrenocorticotrophic hormone
AE	adverse event
AIPC	androgen-independent prostate cancer
ALP	alkaline phosphatase
ALT	alanine aminotransferase
AR	androgen receptor
AST	aspartate aminotransferase
AUC	area under curve
BP	blood pressure
BPI-SF	Brief Pain Inventory - short form
CHMP	Committee for Medicinal Products for Human Use
CI	confidence interval
C_{max}	maximum observed concentration
CPT	cryopreservation tube
CT	computed tomography
CRP	C-reactive protein
CRPC	castration-resistant prostate cancer
CTCAE	Common Terminology Criteria for Adverse Events
CYP1A2	cytochrome P450 1A2
CYP17	cytochrome P450 C17
CYP2C8	cytochrome P450 2C8
CYP2D6	cytochrome P450 2D6
CYP3A4	cytochrome P450 3A4
DHEA	dehydroepiandrosterone
DHEA-S	dehydroepiandrosterone-sulphate
DHT	dihydrotestosterone
DNA	deoxyribonucleic acid
DRE	digital rectal exam
DXA	dual-energy X-ray absorptiometry
ECG	electrocardiogram
ECOG	Eastern Cooperative Oncology Group
eCRF	electronic case report form
eDC	electronic data capture
EOE	end of extension treatment
EOMS	end of main study
EOMT	end of main study treatment
ESMO	European Society for Medical Oncology
EU	European Union
FACT-P	Functional Assessment of Cancer Therapy - Prostate Cancer
GCP	Good Clinical Practice
GC-MS	gas chromatography-mass spectrometry
GEP	gene expression profile
HbA1c	hemoglobin A1c
HOMA-IR	homeostatic model assessment to quantify insulin resistance
HR	hazard ratio
HRPC	hormone refractory prostate cancer
ICF	informed consent form
ICH	International Conference on Harmonisation
IEC	Independent Ethics Committee
INR	international normalized ratio
IRB	Institutional Review Board
ITT	intent-to-treat
IVF	intravenous fluid
IWRS	interactive web response system
LC-MS	liquid chromatography-mass spectrometry
LDH	lactic acid dehydrogenase

LDL	low-density lipoprotein
LHRH	luteinizing hormone releasing hormone
LLN	lower limit of normal
LVEF	left ventricular ejection fraction
mCRPC	metastatic castration-resistant prostate cancer
MedDRA	Medical Dictionary for Regulatory Activities
miRNA	micro ribonucleic acid
mITT	modified intent-to-treat
MRI	magnetic resonance imaging
mRNA	messenger ribonucleic acid
MRU	medical resource utilization
MUGA	multiple gated acquisition scan
NCI	National Cancer Institute
NF	National Formulary
PCWG2	Prostate Cancer Working Group 2
PFS	progression-free survival
PQC	product quality complaint
PRO	patient reported outcome
PSA	prostate specific antigen
PT	preferred term
PTT	partial thromboplastin time
QoL	quality of life
RBC	red blood cell
RECIST	response evaluation criteria in solid tumors
RNA	ribonucleic acid
rPFS	radiographic progression-free survival
SAC	Scientific Advisory Committee
SAE	serious adverse event
SmPC	Summary of Product Characteristics
SOC	system organ class
TPN	total parenteral nutrition
ULN	upper limit of normal
USP/NF/EP	United States Pharmacopeia/the National Formulary/European Pharmacopoeia
WBC	white blood cell
WNL	within normal limits

1. INTRODUCTION

Abiraterone acetate is a prodrug of abiraterone. Abiraterone is a selective irreversible inhibitor of 17α hydroxylase/$C_{17,20}$-lyase (cytochrome P450C17 [CYP17]). Inhibition of CYP17 inhibits the conversion of pregnenolone or progesterone into dehydroepiandrosterone (DHEA) or androstenedione, respectively, which are 2 precursors of testosterone in the adrenal glands and testes. CYP17 inhibition also results in increased mineralocorticoid production by the adrenal glands. Abiraterone acetate is indicated with prednisone or prednisolone for the treatment of metastatic castration-resistant prostate cancer in adult men whose disease has progressed on or after a docetaxel-based chemotherapy regimen. A Type II variation, to extend the indication to include the treatment of metastatic castration resistant prostate cancer in adult men who are asymptomatic or mildly symptomatic after failure of androgen deprivation therapy in whom chemotherapy is not yet clinically indicated, was approved on 18 December 2012.

For the most comprehensive nonclinical and clinical information regarding the efficacy and safety of abiraterone acetate, refer to the latest version of the Investigator's Brochure[31] for abiraterone acetate.

The term "sponsor" used throughout this document refers to the entities listed in the Contact Information page(s), which will be provided as a separate document.

Disease-specific background information

Prostate cancer has become an increasingly important health issue globally. With 679,060 men diagnosed each year, prostate cancers are the fifth most common tumor type worldwide[33].

Prostate cancer is hormone sensitive at the time of initial diagnosis. Although most patients with advanced metastatic disease initially respond to conventional androgen deprivation with medical[14] or surgical[18] castration, the median duration of disease control has been 13 to 22 months and overall survival 28 to 36 months[56]. The clinical status of patients after failure of castration was commonly referred to as hormone-refractory prostate cancer (HRPC), or androgen-independent prostate cancer (AIPC). However, recent investigations have established that tumor progression often remains androgen-dependent albeit at much reduced systemic androgen levels after castration. Although widely used in clinical settings, the terms HRPC and AIPC do not reflect the biology of advanced prostate cancer where the androgen receptor (AR) and its ligand remain pivotal in tumor growth. Prostate cancer progression after conventional medical or surgical castration should therefore be considered castration-resistant prostate cancer (CRPC)[3].

Mechanism of action

Testosterone production is a key element in the control of prostate cancer growth. In the testes and adrenal glands, $C_{17,20}$-lyase converts the C_{21} precursors to the corresponding C_{19} androgens. Testosterone, a C_{19} androgen, is further converted to the more potent androgen dihydrotestosterone (DHT) by 5α-reductase in the prostate[9,54]. Both testosterone and DHT stimulate prostatic growth, although DHT plays a much more important role than testosterone in the organogenesis and homeostasis of the prostate[45,71]. Inhibitors of the enzyme

17α hydroxylase/$C_{17,20}$-lyase, such as abiraterone acetate and abiraterone, block testosterone production both in the testis and adrenal gland.

In castrated men, as much as 10% of baseline circulating testosterone remains because of conversion of adrenal androgens to testosterone[30]. In addition, more than 10% of baseline concentrations of the androgens testosterone, DHT, dehydroepiandrosterone-sulphate (DHEA-S), and androstenedione remain in recurrent prostate cancer tissue after castration[39]. Autocrine synthesis may contribute to these high prostate tumor androgen concentrations since high concentrations of CYP17 messenger ribonucleic acid (mRNA) are found in high Gleason score prostate tumors of subjects who experience metastasis[60].

Furthermore, the AR becomes amplified in prostate tumor cells, and the amplified AR is activated by extremely low androgen concentrations[12]. The sensitivity of the AR is also increased by the overexpression of 2 nuclear coactivators, which enhances activation of the AR at lower testosterone concentrations[26,27]. Together, the increased sensitivity of the AR with persistent androgens after castration results in tumor progression in many men with CRPC.

Therefore, even though the progression of prostate cancer may be slowed in the absence of testicular androgens, the tumor remains responsive to stimulation from extratesticular or autocrine androgens. Because of this responsiveness, anti-androgen therapy with abiraterone acetate may still be effective after medical or surgical castration.

Current second-line hormone therapies include anti-androgens such as flutamide, bicalutamide, and nilutamide, estrogens including diethylstilbestrol, or adrenal androgen synthesis inhibitors such as hydrocortisone alone, ketoconazole, and aminoglutethimide[66]. The adverse events (AEs) observed in subjects receiving ketoconazole or aminoglutethimide were thought to be related to the nonspecific nature of enzyme inhibition. Since abiraterone acetate, and its chief metabolite abiraterone, are selective inhibitors of 17α hydroxylase/$C_{17,20}$-lyase, administration is expected to reduce the risk of nonspecific enzyme inhibition affecting the synthesis of glucocorticoids and mineralocorticoids, improving efficacy and minimizing AEs. Figure 1 depicts the suppression of estradiol by abiraterone acetate[6,5].

Data from early studies have contributed to the current understanding of the mechanism of abiraterone action. These suggest that a state of mineralocorticoid excess can occur after pharmacologic inhibition of CYP17, with the resulting reduced cortisol levels leading to a compensatory adrenocorticotrophic hormone (ACTH) surge and accompanying hypertension, hypokalemia, and fluid retention[7]. These side effects were readily managed with potassium supplementation, eplerenone (selective mineralocorticoid antagonist), antihypertensive agents, and low dose corticosteroids. Grade 1-2 fatigue was observed in some patients and was associated with discontinuation of corticosteroids as required per Phase 2 protocol entry criteria and extended duration of treatment with abiraterone acetate. Although there was no evidence of an abiraterone dose-response relationship, administration of low dose corticosteroids as specified in the study improved symptoms of fatigue and tolerability of abiraterone acetate, including symptoms of mineralocorticosteroid excess. The improved tolerability of abiraterone acetate

after concomitant administration of low-dose corticosteroids was associated with suppression of ACTH and upstream adrenal steroids.

Figure 1: The Suppression of Estradiol by Abiraterone Acetate's Inhibition of CYP17 in Men With CRPC

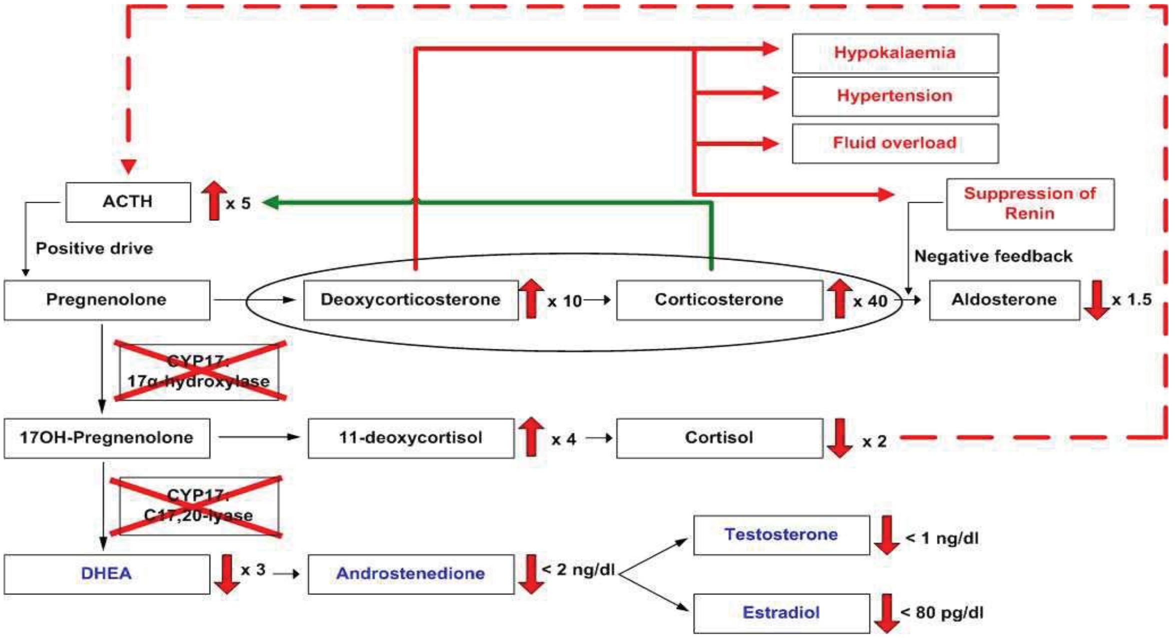

Clinical Studies

Up to 27 April 2013, 3,012 subjects had received abiraterone acetate in clinical studies since the start of the clinical development program. Of these, 668 subjects were exposed to abiraterone acetate in the Phase 1 program, 162 subjects were exposed to abiraterone acetate in the Phase 1/2 program, 849 subjects were exposed to abiraterone acetate in the Phase 2 program, and 1,333 subjects were exposed to abiraterone acetate in the Phase 3 program. In an expanded access program, 2,236 subjects from 15 countries were treated with abiraterone acetate.

Human Pharmacokinetics

In subjects with metastatic castration-resistant prostate cancer (mCRPC), the mean maximum observed concentration ($C_{max)}$) and area under curve (AUC) of abiraterone after a single dose of 1,000 mg abiraterone acetate under modified fasting conditions were approximately 182 ng/mL and 675 ng*h/mL, respectively. In these subjects, after 28 days of continuous daily dosing, mean C_{max} and AUC were increased approximately 2.0- and 2.2-fold to 226 ng/mL and 993 ng*h/mL, respectively. The estimated accumulation ratio (2.0 for C_{max}, 2.2 for AUC) is compatible with an effective half-life in a multiple-dose setting of 24 to 28 hours, higher than that estimated from single-dose studies under fasting conditions in healthy subjects. Overall, the exposure to abiraterone in subjects with mCRPC was higher than in healthy male subjects.

Administration of abiraterone acetate with food significantly increased the absorption of abiraterone acetate. Administration of 1,000 mg doses of abiraterone acetate tablets in fed conditions increased systemic exposure to abiraterone compared with the fasted state. Abiraterone mean C_{max} and AUC values increased approximately 7- and 5-fold, respectively,

when administered immediately after a low-fat meal. Abiraterone mean C_{max} and AUC values increased approximately 17- and 10-fold, respectively, when administered immediately after a high-fat meal.

Efficacy/Safety Studies

The efficacy of abiraterone acetate has been demonstrated based on 2 randomized, placebo-controlled, multicenter, Phase 3 clinical studies: a study of subjects with mCRPC who had previously received docetaxel chemotherapy (Study COU-AA-301[15]), and a study of subjects with chemotherapy-naïve mCRPC who had shown tumor progression and were asymptomatic or mildly symptomatic (Study COU-AA-302[52]). Studies COU-AA-301[15] and COU-AA-302[52] form the basis of the currently approved indication.

Study COU-AA-301

For Study COU-AA-301, the primary endpoint was overall survival. Treatment with abiraterone acetate plus prednisone prolonged overall survival in this patient population and reduced the risk of death by 26% compared with placebo (hazard ratio [HR]=0.740; 95% confidence interval [CI]: 0.638, 0.859; p<0.0001). There was a 41% improvement in median survival (482.0 days [15.8 months] in the abiraterone acetate group and 341.0 days [11.2 months] in the placebo group). These benefits were achieved without the toxic effects characteristic of cytotoxic chemotherapy.

In addition to the observed improvement in overall survival for Study COU-AA-301, all secondary study endpoints favored abiraterone acetate and differences versus placebo were statistically significant. Abiraterone acetate demonstrated an improvement in subject reported pain outcomes and time to first skeletal-reported event.

Study COU-AA-302

Study COU-AA-302 had co-primary endpoints of radiographic progression-free survival (rPFS) and overall survival. At the time of the primary rPFS analysis (20 December 2010):

- Treatment with abiraterone acetate plus prednisone resulted in a 58% decrease in the risk of rPFS (independent review) compared with placebo and prednisone (HR=0.425; 95% CI: 0.347, 0.522; p<0.0001). The median rPFS was not reached in the abiraterone acetate group and was 8.3 months in the placebo group. The results were similar and highly consistent between the independent and the investigator assessments of rPFS.
- The treatment effect for rPFS was statistically significant and consistently in favor of the abiraterone acetate group across all subgroups examined.

At the time of the second interim analysis for overall survival (20 December 2011):

- Treatment with abiraterone acetate plus prednisone resulted in a 25% decrease in the risk of death compared with placebo plus prednisone (HR=0.752; 95% CI: 0.606, 0.934; p=0.0097). The study did not reach the prespecified significance level for overall survival of p=0.0008. After a median follow-up of 22.2 months, the median overall survival was not reached in the abiraterone acetate group and was 27.2 months for the placebo group.

- The treatment effect for overall survival was consistently in favor of the abiraterone acetate group across all the subgroups examined.

Results for all the secondary endpoints (time to opiate use for cancer pain, time to initiation of cytotoxic chemotherapy, time to Eastern Cooperative Oncology Group [ECOG] performance status deterioration, and time to prostate specific antigen [PSA] progression) favored abiraterone acetate plus prednisone treatment.

The data indicate a highly significant clinical benefit and advantage for patients in the experimental arm. The risk of death was reduced by 25% for the group treated with abiraterone acetate. Although the nominal p-value (0.0097) did not meet the protocol prespecified criteria for statistical significance of 0.0008, the independent data monitoring committee unanimously recommended stopping of the study due to the magnitude of difference observed on the co-primary endpoints, including the large difference in the treatment effect observed in the secondary endpoints.

The safety profile of abiraterone acetate across studies in mCRPC was distinct from the safety profile typically associated with myelosuppressive cytotoxic agents. For the combined treatment groups, the most frequently reported AEs (≥20% of subjects in either combined treatment group) were fatigue, back pain, arthralgia, constipation, bone pain, peripheral edema, hot flush, and diarrhea, AEs that are consistent with mCRPC disease.

1.1. Concurrent Glucocorticoids

Glucocorticoids appear to possess both hormonal and direct anti-tumor effects in prostate cancer. Patients with CRPC may still have hormone-sensitive disease that is stimulated by weak androgens of adrenal origin, and these androgens are suppressed by prednisone through its negative feedback on secretion of ACTH. In a study with 37 men with symptomatic bone metastases from prostate cancer who had progressed following earlier treatment with estrogens and/or orchidectomy, Tannock et al[64] demonstrated that low-dose prednisone treatment (7.5 to 10 mg daily) led to a decrease in the concentration of serum testosterone in 7 of 9 subjects where it was not initially suppressed below 2.0 nmol/mL. This also resulted in a decrease in serum levels of androstenedione and dehydroepiandrosterone sulfate in more than 50% of subjects. These changes were associated with symptomatic and clinical improvement. Glucocorticoids may also have direct inhibitory effects on prostate cancer cells through enhanced growth-inhibitory transforming growth factor-beta 1 (TGF-ß1) signaling and suppression of the transcriptional activities of nuclear factor-kappa B (NF-κB)[42,37].

Two prospective Phase 3 studies have documented the AE profile and palliative benefit of prednisone. Prednisone 7.5 to 10 mg daily was examined among 81 subjects in one arm of a Phase 3 study, with 22% of subjects achieving a 50% PSA decline and a median time to progression of 4.0 months[65]. Likewise, in a randomized study control arm where 201 subjects were treated with prednisone 5 mg twice daily, PSA decline of ≥50% was observed in 21% of subjects[24]. Significant improvements in pain, quality of life and fatigue were also reported.

Importantly, the concomitant administration of a low dose of glucocorticoid can also prevent symptoms associated with mineralocorticoid excess during abiraterone treatment.

As of 27 April 2013, abiraterone acetate with prednisone or prednisolone is approved in 76 countries for the treatment of men with mCRPC.

1.2. Overall Rationale for the Study

Based upon experience from Phase 1 and ongoing Phase 2 studies, abiraterone acetate is generally well tolerated. The most common AEs related to abiraterone acetate monotherapy include: fatigue due to reduced cortisol level as a result of CYP17 inhibition; and hypertension, fluid retention, and hypokalemia due to mineralocorticoid excess caused by compensatory ACTH drive. The current indication includes a recommended daily dose of 5 mg twice daily of prednisone. In this study, the concomitant administration of prednisone at a lower dose or equivalent (5 mg once daily or 2.5 mg twice daily) or dexamethasone (0.5 mg once daily) is expected to control the mineralocorticoid excess toxicities and decrease the risk of mid-term and long-term steroid side effects.

It has been documented that following prolonged therapy with corticosteroids, patients may develop Cushing's syndrome characterized by central adiposity, thin skin, easy bruising, and proximal myopathy. Withdrawal of the corticosteroid may result in symptoms that include fever, myalgia, fatigue, arthralgia, and malaise. This may occur even without evidence of adrenal insufficiency.

Subjects that will be enrolled in this study are chemotherapy-naïve and are expected to receive longer treatment duration with abiraterone acetate and glucocorticoids in comparison to patients who receive abiraterone with prednisone according to the current label. They have more chance to be exposed to steroid side effects (mid-term and long-term). Consequently, there is a need to define alternative steroid treatment strategies such as a reduced dose of prednisone (5 mg once daily or 2.5 mg twice daily) or the use of dexamethasone (0.5 mg once daily).

Dexamethasone 0.5 mg may have greater activity, in terms of PSA response, and a different safety profile (lacking water and salt-retaining properties) in comparison to the other corticosteroids in CRPC and has been tested with abiraterone acetate in the prechemotherapy (COU-AA-001) and the post-docetaxel (COU-AA-003) clinical studies[3,31]. Additionally, in a retrospective study[69] of 102 subjects with progressive CRPC, treatment with dexamethasone 0.5 mg daily demonstrated a confirmed PSA response in 49% of the subjects. In responders, the median duration of PSA response was 11.6 months.

The aim of this 4-arm, randomized, Phase 2 study is to evaluate alternative steroid treatment strategies for preventing symptoms associated with mineralocorticoid excess and minimizing toxicity from long-term glucocorticoid use in subjects treated with abiraterone acetate for asymptomatic and chemotherapy-naïve mCRPC.

2. OBJECTIVES AND HYPOTHESIS

2.1. Objectives

Primary Objective

The primary objective is to evaluate the safety of abiraterone acetate with 4 alternative steroid treatment strategies related to symptoms associated with mineralocorticoid excess toxicities (ie, hypokalemia and/or hypertension) during the first 24 weeks of treatment in asymptomatic, chemotherapy-naïve, mCRPC subjects.

Secondary Objectives

The following secondary objectives will be assessed during the entire study in asymptomatic, chemotherapy-naïve and mCRPC subjects treated with abiraterone acetate and 4 alternative steroid treatment strategies:

- To further characterize the global safety profile (including the incidence of mineralocorticoid excess toxicities [eg, hypokalemia and hypertension]).
- To characterize mid-term and long-term exogenous glucocorticoid side effects.
- To characterize the clinical benefit.
- To evaluate the impact on pain and quality of life (QoL) as measured by the EQ-5D-5L, Brief Pain Inventory - short form (BPI-SF) and Functional Assessment of Cancer Therapy - Prostate Cancer (FACT-P) tools.
- To collect medical resource utilization (MRU) data that may be used in future economic modeling (the construction and reporting of the economic model will be conducted separately from this study).
- To evaluate overall survival.
- To collect data on subsequent therapies for prostate cancer (time to next therapy for prostate cancer, time to initiation of chemotherapy, treatment duration, best response) following cessation of study treatment.

Additionally, the study includes optional biomarker analyses of circulating genetic material as a secondary exploratory objective to:

- Identify genomic aberrations in circulating plasma deoxyribonucleic acid (DNA), and evaluate expression of circulating plasma and whole blood mRNA and microRNA (miRNA) occurring pre- and on-treatment.
- Analyze circulating biomarkers as predictors of response or sensitivity to abiraterone acetate or for identifying mechanisms of resistance.

2.2. Hypothesis

It will be determined whether the 4 treatment arms warrant further study based on the "no mineralocorticoid excess" rate. “No mineralocorticoid excess” is defined as experiencing (yes or no) neither of the 2 mineralocorticoid excess toxicities, ie, neither hypokalemia nor hypertension, during the first 24 weeks of treatment. The highest percentage of subjects experiencing neither

hypokalemia nor hypertension during the first 24 weeks at which it is required to reject the treatment is 50% and the treatment would be worth developing further if this rate is 75%.

3. STUDY DESIGN AND RATIONALE

3.1. Overview of Study Design

This is a randomized, open-label, parallel-arm, multicenter, Phase 2 study of treatment with abiraterone acetate and 4 alternative steroid treatment strategies in asymptomatic, chemotherapy-naïve, mCRPC subjects.

The subject population will comprise medically or surgically castrated men aged 18 years or older with mCRPC who have shown tumor progression and are asymptomatic by prospectively defined criteria (Section 4.1).

A target of 144 subjects will be enrolled in this study, with 36 subjects planned per treatment arm. Eligible subjects should have mCRPC documented by positive bone scan or by computed tomography (CT) or magnetic resonance imaging (MRI).

The study will consist of a screening phase of 4 weeks followed by an open-label main study treatment period of a maximum of 39 cycles (156 weeks), with an extension phase for progression-free subjects (if applicable, see Section 3.1.2), followed by a follow-up phase. The main study treatment period cut-off date will be in July 2016, 156 weeks after the start of study treatment for the first subject participating in the study. Subjects will participate in the main study treatment period until the cut-off date; study treatment will be administered until radiographic disease progression, and/or unequivocal clinical progression, and/or other reasons for discontinuation detailed in Section 10.

End-of-main-study-treatment (EOMT) assessments will be performed for all subjects randomized into the main study treatment period, regardless of whether they complete study treatment up to the cut-off date in July 2016 or discontinue from the main study treatment period before the cut-off date. For subjects who have completed the main study treatment period up to the cut-off date, EOMT assessments will be performed 4 weeks after Day 1 of the current cycle (ie, at the end of the cycle) and at the latest by 16 July 2016±15 days. For subjects discontinuing from the main study treatment period, EOMT assessments will be performed as close as possible to the time of discontinuation and before initiation of any new anticancer treatment, including radiotherapy. Additionally, for subjects discontinuing study treatment before the cut-off date, an end-of-main-study (EOMS) visit will be performed 4 weeks after the cessation of study medication.

A Scientific Advisory Committee (SAC) will be commissioned for this study. See Section 11.8 for details.

A diagram of the study design is provided in Figure 2.

Figure 2: Diagram of the Study Design

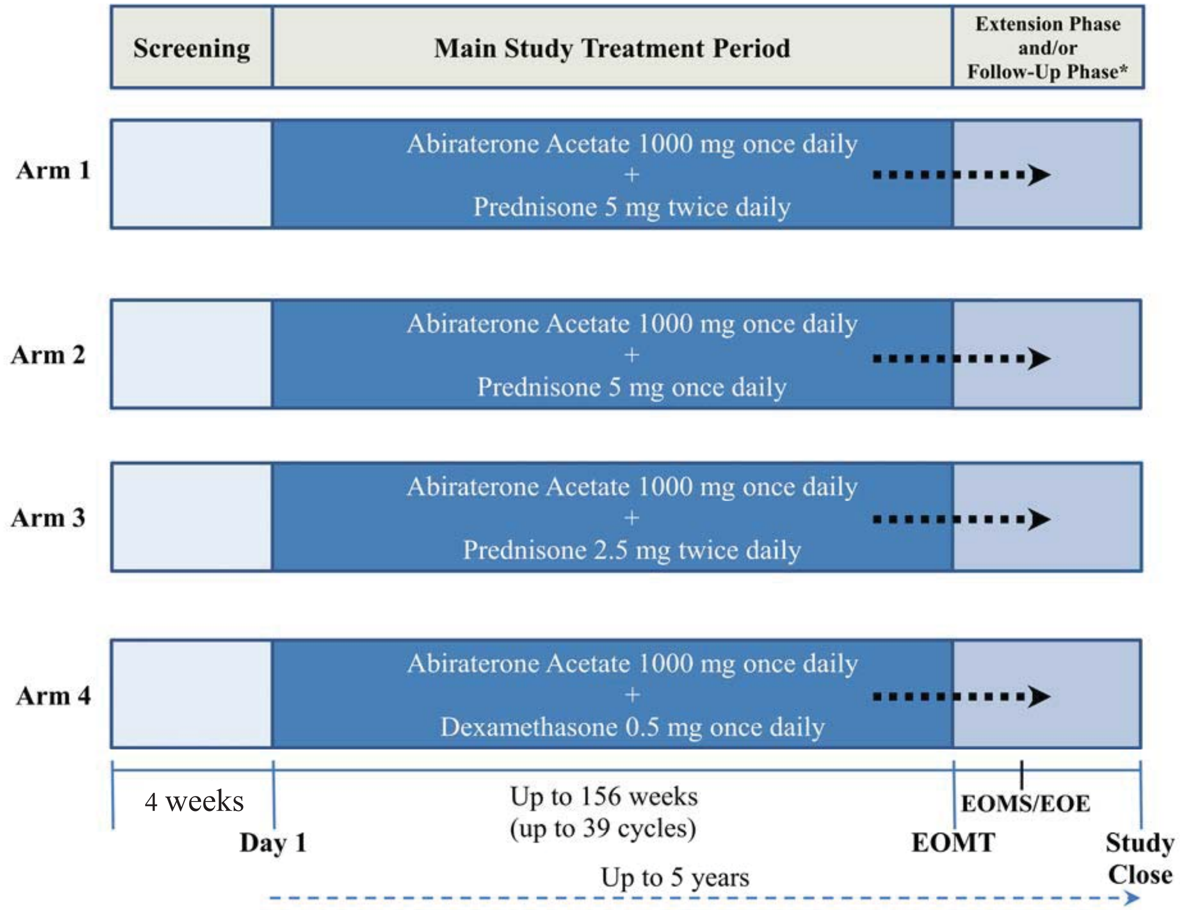

* Extension phase enabling continuation of study treatment in progression-free subjects (if applicable, see Section 3.1.2).
Follow-up phase: follow up for survival and subsequent prostate cancer therapy after study treatment discontinuation (see Figure 3)

EOMT = End of main study treatment

EOMS/EOE = End-of-main-study visit or end-of-extension-treatment visit, 4 weeks after last study treatment

Study Close = 5 years after the start of study treatment of the first subject participating in the study

Subjects will be randomized in a 1:1:1:1 ratio to receive abiraterone acetate with either prednisone 5 mg twice daily (Arm 1), prednisone 5 mg once daily (Arm 2), prednisone 2.5 mg twice daily (Arm 3) or dexamethasone 0.5 mg once daily (Arm 4). Abiraterone acetate will be taken as oral tablets at a once daily dose of 1,000 mg. Oral prednisone will be taken either as a once-daily dose (5 mg) or twice daily (2.5 mg or 5 mg) with a minimum interval of 8 hours. Dexamethasone (0.5 mg) will be taken as a single dose. Abiraterone acetate, prednisone and dexamethasone will be considered as study drugs. Refer to Section 6 for further details on dosage and administration. Permitted or prohibited concomitant therapies are discussed in Section 8. Osteoprotective therapy may be administered according to local clinical practice and the product label. According to the original protocol, any such osteoprotective therapy must have been started before Day 1, Cycle 1. Per protocol amendment INT-5, initiation of bisphosphonates or other approved osteoprotective agents during the study will be allowed after appropriate

documentation of the bone loss, and according to local clinical practice and the current product label.

3.1.1. Assessments

All subjects must sign a written informed consent form (ICF) before any study-specific procedure is performed. Each subject consenting to participate in the optional biomarker research phase must also sign a separate ICF before Day 1, Cycle 1, to indicate that he agrees to provide optional blood samples for biomarker research (where local regulations permit). Screening procedures to evaluate subject eligibility for the study will be conducted within 4 weeks prior to randomization. Laboratory tests, ECG, Multiple Gated Acquisition scan (MUGA)/echocardiography, dual-energy X-ray absorptiometry (DXA), CT, MRI, and bone scan assessments performed according to standard of care before the subject has signed the ICF can be used as screening assessments and do not need to be repeated, provided these have been performed within the time interval, and according to the methods, specified in the protocol. When the results of all screening assessments are known and show that the subject is eligible for inclusion, he will be randomized to a treatment arm. Study medication will be initiated within 72 hours (3 calendar days) of randomization.

The primary parameter is defined as experiencing (yes or no) neither of the 2 mineralocorticoid excess toxicities during the first 24 weeks of treatment, ie, neither hypokalemia nor hypertension. This will be derived from treatment-emergent AE data, which will be defined using the Medical Dictionary for Regulatory Activities (MedDRA) and will be graded according to the National Cancer Institute Common Terminology Criteria for Adverse Events (NCI CTCAE v4.0). Serum potassium and blood pressure (BP) will be monitored throughout the study and incidences of hypokalemia of any grade ≥1 or of hypertension grade ≥2 (NCI CTCAE v4.0) will be reported as AEs.

Secondary safety parameters will assess the global safety profile (including the incidence of mineralocorticoid excess toxicities [eg, hypokalemia and hypertension], and the incidence of mid-term and long-term glucocorticoid side effects during the entire study and according to NCI CTCAE v4.0. All events will be collected from signing of the informed consent onwards.

Safety and tolerability (including monitoring of AEs and serious adverse events [SAEs]; clinical laboratory tests including hematology, chemistry, serum androgen/androgen precursors profile, urine full steroid excretion profile, homeostatic model assessment to quantify insulin resistance [HOMA-IR], ACTH; vital signs including seated BP, heart rate, respiratory rate, and oral, or axillary, or tympanic body temperature; DXA scans; physical examinations, and body weight) will be assessed at scheduled visits.

Efficacy assessments will include PFS (based on rPFS, clinical progression or death) and survival. The assessment of rPFS will utilize sequential imaging studies as defined by Prostate Cancer Working Group 2 (PCWG2) criteria and modified response evaluation criteria in solid tumors (modified RECIST, v1.1). Scheduling of assessments will be defined according to locally-defined guidelines. PSA values will be collected throughout the study to assess PSA response rate and time to PSA progression. Evaluations of response will be recorded. Any

evidence of soft tissue progression as defined in the modified RECIST,[19] including assessments performed according to local clinical practice, will be documented. Time to opiate use for cancer-related pain will be prospectively assessed. ECOG performance status will be evaluated throughout the main study treatment period to assess time to first deterioration.

Pain and QoL will be monitored by administration of EQ-5D-5L, BPI-SF and FACT-P at scheduled visits or if disease progression is suspected. EQ-5D-5L, BPI-SF and FACT-P are patient reported outcome (PRO) assessments, ie, completed by the subject (or, if a subject cannot read or complete it independently, by a designated member of the staff as verbally stated by the subject).

For subjects consenting to participate in the biomarker research phase, blood samples will be collected at predefined time points for biomarker analyses in whole blood and plasma.

Medical resource utilization data, associated with medical encounters, will be collected in the electronic case report form (eCRF) by the investigator and staff for all subjects throughout the main study treatment period.

3.1.2. Extension and Follow-up Phases

Subjects who discontinue study treatment due to disease progression, or initiation of another prostate cancer treatment, or due to safety reasons, or at the subject's request, at any time during the study, will enter a follow-up phase to the study. These subjects will be followed-up for survival and for subsequent prostate cancer therapies until July 2018 (5 years after the start of study treatment of the first subject participating in the study).

Subjects who require diuretic treatment or a change in glucocorticoid dose, or who experience grade 3 or grade 4 hypokalemia will also be discontinued from the main study in the absence of disease progression. However, these subjects will have the option of entering the extension phase, allowing continuation of their study treatment until disease progression or death if earlier, after normalization of the subject's condition (grade ≤1) if, in the opinion of the investigator, the subject will benefit from continuation of study treatment. Similarly, subjects who are progression-free at the cut-off date will be able to enter the extension phase of the study and continue study treatment until disease progression, or death if earlier. If a progression-free subject does not enter the extension phase of the study, the reason for this must be documented during the EOMT assessment and an EOMS visit will be performed 4 weeks after the last study treatment.

During the extension phase, as part of routine clinical care, subjects should be monitored according to the approved Summaries of Product Characteristics (SmPCs)[16,47,48,49,72]; (S)AEs and survival data will be collected. Following disease progression or discontinuation of extension study treatment for any other reason, there will be an end-of-extension-treatment (EOE) visit 4 weeks after cessation of study medication. Assessments at the EOE visit will include best response to abiraterone acetate. Thereafter, survival and subsequent prostate cancer therapies (including best response) will be monitored during the follow-up phase until July 2018 (5 years after the start of study treatment of the first subject participating in the study).

3.1.3. Study Duration

The main study treatment period will be considered complete at the cut-off date, ie, 156 weeks (equivalent to a maximum of 39 study treatment cycles) after the start of study treatment for the first subject participating in the study. Therefore, each subject will participate in the main study treatment period for a maximum of 156 weeks, depending on the time of enrollment. With the extension and follow-up phases, the entire study will be complete in July 2018 (5 years after the start of study treatment of the first subject participating in the study).

A diagram summarizing subjects' progress through the main study treatment period, extension phase and follow-up phase is shown in Figure 3.

Figure 3: Summary of the Main Study, Extension Phase and Follow-up Phase

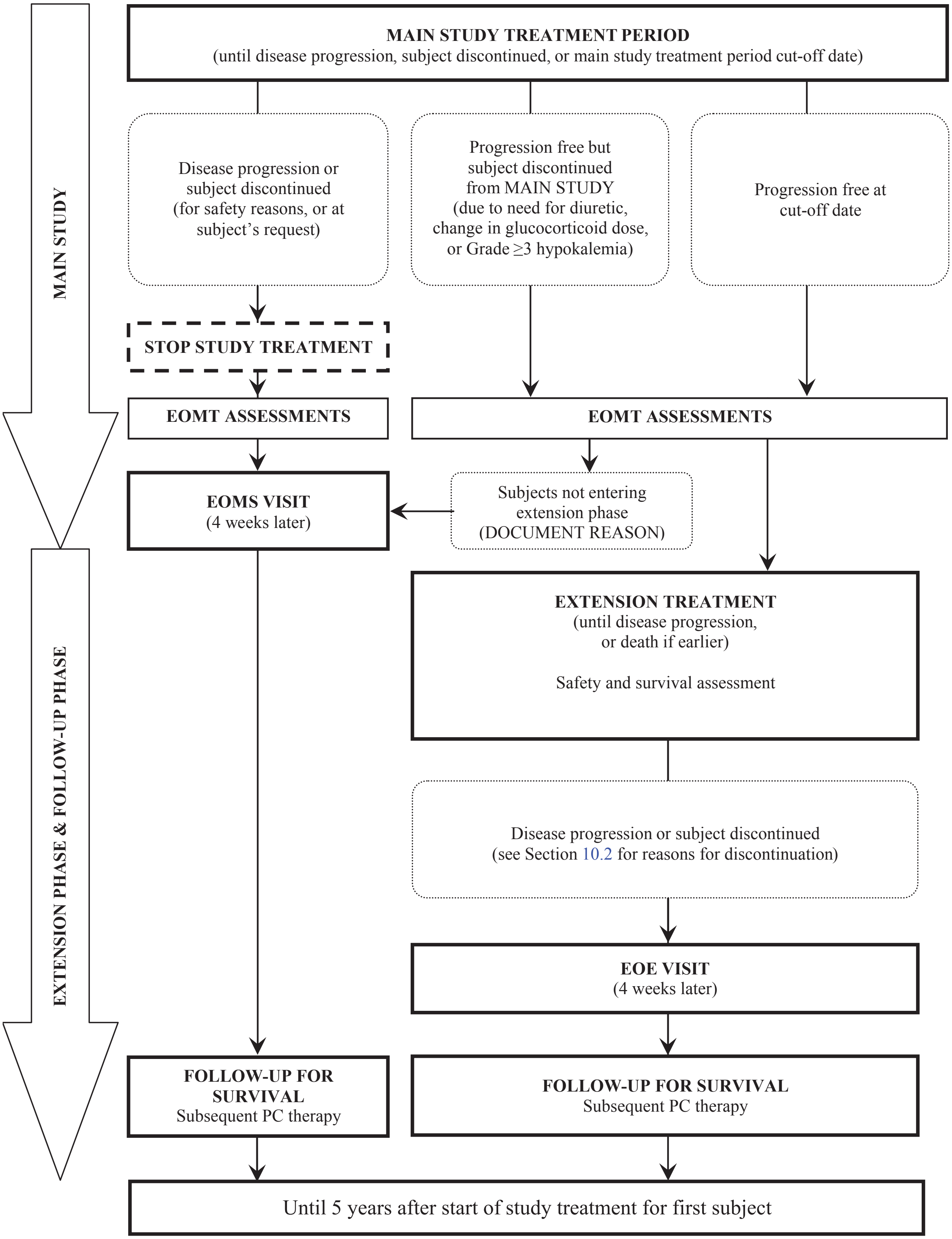

PC: Prostate Cancer

3.2. Study Design Rationale

Blinding, Control, Study Phase, Treatment Arms

Randomization will be used to minimize bias in the assignment of subjects to treatment arms, to increase the likelihood that known and unknown subject attributes (eg, demographic and baseline characteristics) are evenly balanced across treatment arms.

The present study will be performed in subjects with asymptomatic, chemotherapy-naïve mCRPC. This patient population was studied previously in COU-AA-302[52], a Phase 3, randomized, double-blind, placebo-controlled study of abiraterone acetate plus prednisone in asymptomatic or mildly symptomatic subjects with mCRPC. Interim analysis showed that abiraterone acetate plus prednisone produced a statistically significant improvement in rPFS and a strong trend for increased overall survival. Abiraterone acetate resulted in clinically and statistically significant effects on all secondary endpoints. The results confirmed the acceptable safety/tolerability profile of abiraterone acetate in asymptomatic or mildly symptomatic subjects. This was the first randomized trial to demonstrate both overall survival and rPFS benefits in chemotherapy-naïve mCRPC.

Thirty-six out of the 42 CRPC patients treated with single-agent abiraterone acetate experienced clinical evidence of a syndrome of mineralocorticoid excess that was treated with eplerenone initiated after a median of 28 days[4]. The time point for analyzing the primary endpoint in this study will be after 24 weeks of treatment, which will provide an accurate incidence of the mineralocorticoid excess rate with a minimum drop-out. Furthermore, full analyses of the other secondary parameters at this time point will provide information on the mid-term steroid side effects. Analyses on long-term steroid side effects require a sufficient length of treatment with steroids in order to experience these side effects. In the previously mentioned Phase 3 study in asymptomatic or mildly symptomatic subjects, COU-AA-302[52], the mean treatment duration was 13.8 months (range 0.3-29.9 months). In the present study, the maximum main study treatment period duration for a subject, depending on the time of enrollment, will be 3 years (ie, 36 months; 39 cycles; 156 weeks), which will permit adequate analyses on long-term steroid side effects. Moreover, as the present study will be limited to asymptomatic subjects, the mean treatment duration will be expected to increase relative to the COU-AA-302 study.

Inclusion of an extension phase will enable progression-free subjects to continue study treatment at the end of the main study treatment period or following discontinuation from the main study, when appropriate (see Section 16.1). Data on all subsequent therapies for prostate cancer (treatment duration, best response) will be collected during a follow-up phase to provide information on the efficacy of subsequent therapies after abiraterone acetate. Collection of survival data throughout the study may also enable circulating biomarker analyses to be related to survival.

The dose of abiraterone acetate in this study is 1,000 mg daily, based on results of previous studies including 2 Phase 1 dose-finding studies, COU-AA-001[3] and COU-AA-002[51], and the Phase 3 efficacy studies COU-AA-301[15] and COU-AA-302[52].

Glucocorticoids such as prednisone, dexamethasone, and hydrocortisone have been frequently administered as standard of care in advanced prostate cancer because of their modest antitumor activity and palliative effects on disease. Furthermore, concomitant administration of low doses of a glucocorticoid such as prednisone reduces symptoms associated with mineralocorticoid excess during treatment with abiraterone acetate.

According to the SmPC of abiraterone acetate, the recommended dose of prednisone or prednisolone is 10 mg daily[72]. Prednisone 5 mg twice daily is commonly used as standard of care[29] in combination with approved chemotherapy agents or as a monotherapy for palliation of symptoms. This dose frequency has previously been studied in 2 major efficacy studies (COU-AA-301[15] and COU-AA-302[52]). The present study will also investigate the side effect profile associated with a lower dose of prednisone (2.5 mg twice daily or 5 mg once daily) or with an equivalent dose of dexamethasone.

Once-daily dosing of prednisone may be more convenient for patients than twice-daily dosing. It is also possible that once-daily dosing in the morning will result in fewer sleep disturbances than twice-daily dosing of prednisone. Additionally, 2 recent neoadjuvant studies (Taplin et al[67] and Efstathiou et al[16]) concluded that no new safety signals were seen with abiraterone acetate when used with prednisone 5 mg once daily. However, the adrenal pathway is continuously stimulated during abiraterone acetate treatment. In the absence of 24-hour steroid control, mineralocorticoids may accumulate and cause side effects. Due to the short plasma elimination half-life of prednisone/prednisolone (approximately 3 hours[48]), twice-daily dosing may be important to maintain constant exposure over 24 hours. Therefore, this study will evaluate both 5 mg once-daily and 2.5 mg twice-daily dose regimens of prednisone.

Dexamethasone has antitumor activity in prostate cancer[41,53,61] and has been tested with abiraterone acetate in a Phase 1 study[3]. In a large, retrospective study of patients with progressive CRPC, dexamethasone 0.5 mg daily showed a 49% rate of PSA decline and an 11.6-month median duration of PSA response[69]. Additionally, dexamethasone 0.5 mg may have greater activity, in terms of PSA response, and an improved safety profile (lacking water and salt-retaining properties) in comparison to other corticosteroids in CRPC[69]. The toxicity profile of dexamethasone 0.5 mg daily makes it an attractive candidate for combined therapy. Clinical experience of glucocorticoid replacement therapy, and studies comparing relative anti-inflammatory properties, indicates that dexamethasone 0.5 mg is equipotent to prednisolone 4 mg and to prednisone 5 mg[2]. Moreover, the long biological half-life (36 to 54 hours) of dexamethasone enables once daily dosing[16].

Safety Parameters

Mineralocorticoid excess toxicities (eg, hypokalemia, hypertension and fluid retention) will be assessed by BP measurements, laboratory assessments (including serum sodium, potassium, urea and creatinine), DXA scanning and physical examination. Since serum potassium and BP are well defined objective assessments, which can be reliably measured, the primary parameter will be based on the incidence of hypokalemia and hypertension as indicators of mineralocorticoid excess.

The global safety profile, including fluid retention/edema, will be assessed as a secondary objective. Fluid retention will be assessed clinically and by DXA scanning. DXA scans will be used to measure the level of water retention in the body; whole-body DXA scans can measure small differences between treatments in the amount of fluid going into the extravascular system, although the definition of pathological water retention observed by DXA requires characterization.

Moreover, since DXA scanning measures exact body composition, it is an excellent tool to determine sarcopenia (decrease in skeletal muscle or lean body mass), as previously observed in a recent study during androgen-deprivation therapy for prostate cancer[59], and also to determine increased central fat mass (central obesity). Both sarcopenia and central obesity are possible side effects of systemic glucocorticoid excess due to the exogenous glucocorticoid treatment in this study. In addition, the impact of the combined treatment of abiraterone acetate with glucocorticoids on adrenal function will be assessed by measurement of plasma ACTH and androgens and their precursors (DHEA, DHEA-S, androstenedione, testosterone and DHT).

Endocrine and metabolic steroid side effects (eg, impaired carbohydrate tolerance, cushingoid facies and weight gain) will be assessed by laboratory assessments (including fasting glucose, insulin, hemoglobin A1c [HbA1c], C-reactive protein [CRP], serum lipids), body weight recording, and physical examination. Fasting glucose, insulin (from which HOMA-IR will be calculated) and HbA1c measurements will demonstrate the impact of treatment on insulin resistance. Additionally, DXA scans will be evaluated for redistribution of visceral or subcutaneous fat and sarcopenia (decrease in skeletal muscle or lean body mass) as a side effect of steroid excess.

At predefined time points, DXA scans (or other radiological methods as per local guidelines) will also be used to assess musculoskeletal adverse effects including osteoporosis, tendon rupture, and vertebral and long bone fractures. Symptoms concerning fluid and electrolyte disturbance (hypertension, hypokalemia, sodium and water retention) following mid-term and long-term steroid use will be assessed by BP measurements, laboratory assessments (serum chemistry/electrolytes) and physical examination. Infections, gastrointestinal symptoms (eg, nausea, increased appetite, peptic ulceration) and neuropsychiatric symptoms (eg, irritability, sleep disturbances) symptoms associated with the use of steroids will be assessed by (S)AE reporting.

Efficacy Parameters

The efficacy parameters include rPFS. Radiographic progression requires visceral or soft-tissue progression on CT or MRI scans using modified RECIST[19] (summarized in Attachment 1), or, more commonly, appearance of new bone metastases on radionuclide scans.[55]

Other efficacy parameters will also allow a direct estimation of the clinical benefit of delay in radiographic progression. Data on time to opiate administration for cancer pain, and time to deterioration in ECOG performance status will be collected prospectively using clear definitions of events. Standard secondary endpoints of efficacy in CRPC (time to PSA progression and PSA response rate based on PCWG2 criteria) will also be measured.

Health-Related Patient Reported Outcome (PRO) Questionnaires

The 2 health-related QoL questionnaires, EQ-5D-5L[20] and FACT-P[50], are widely used and appropriate for this patient population.

The FACT-P tool has been selected for use in this study based on several factors: 1) its content and psychometric properties have been well validated[22]; and 2) clinically meaningful changes in the FACT-P have been determined by validation comparisons with clinical measures of disease burden[11].

The BPI-SF is a validated and widely recognized tool for the assessment of pain.[13,58] The BPI uses simple numeric rating scales from 0 to10 that are easy to understand and easy to translate into other languages.

Survival and Subsequent Prostate Cancer Therapies

In addition, data on overall survival and subsequent prostate cancer therapies will be collected (see Section 9.3.1). This should allow a comprehensible clinical context for improvements in time to radiographic progression.

Medical Resource Utilization Data Collection

There is a lack of MRU data for patients presenting with mCRPC. MRU data obtained during this study, including information on number and duration of medical care encounters and hospitalizations, would support the construction of meaningful health economic models.

Biomarker Sample Collection

For subjects consenting for the optional biomarker research phase, blood samples will be collected for biomarkers analyses in whole blood and plasma, to evaluate factors associated with resistance or sensitivity to treatment with abiraterone acetate. This may help to explain inter-individual variability in clinical outcomes or to identify population subgroups that respond differently to abiraterone acetate with alternative steroid treatment strategies. The resulting data may aid evaluation of the drug-clinical response relationship (see Section 9.4).

4. SUBJECT SELECTION

The inclusion and exclusion criteria for enrolling subjects in this study are described in the following 2 subsections. If there is a question about the inclusion or exclusion criteria below, the investigator should consult with the appropriate sponsor representative before enrolling a subject in the study.

Laboratory tests, ECG, MUGA/echocardiography, DXA scan, CT, MRI, and bone scan assessments performed according to standard of care prior to signature of the ICF can be used as screening assessments, and do not need to be repeated, provided these have been performed within the time interval, and according to the methods, specified in the protocol (see TIME AND EVENTS SCHEDULE and TIME AND EVENTS SCHEDULE – laboratory test schedule).

4.1. Inclusion Criteria

Each potential subject must satisfy all of the following criteria to be enrolled in the study. Each subject must:

1. Be a man of 18 years of age or above.

2. Have a histologically or cytologically confirmed adenocarcinoma of the prostate.

3. Modified per amendment.

    3.1 Have metastatic disease documented by positive bone scan or by CT or MRI.

4. Have prostate cancer progression documented by PSA according to PCWG2 or radiographic progression according to modified RECIST (v1.1) criteria.

5. Be asymptomatic from prostate cancer. A score of 0-1 on BPI-SF Question #3 (worst pain in last 24 hours) will be considered asymptomatic.

6. Be surgically or medically castrated, with testosterone levels of <50 ng/dL (<2.0 nmol/L). If the subject is being treated with luteinizing hormone releasing hormone (LHRH) agonists or antagonists (subjects who have not undergone orchiectomy), this therapy must have been initiated at least 4 weeks prior to Day 1, Cycle 1 and must be continued throughout the study.

7. Have had previous anti-androgen therapy and progression after withdrawal. Subjects who received combined androgen blockade with an anti-androgen must have shown PSA progression after discontinuing the anti-androgen prior to enrollment (≥4 weeks since last flutamide, 6 weeks since last bicalutamide or nilutamide).

8. Have an ECOG Performance Status of 0 or 1.

9. Modified per amendment.

    9.1. Modified per amendment

    9.2. Be normotensive (with systolic BP ≤160 mmHg or diastolic BP ≤95 mmHg), or be hypertensive (with systolic BP ≥160 mmHg or diastolic BP ≥95 mmHg) with a well-controlled BP and receive a maximum of 2 antihypertensives (excluding diuretics) which must have been initiated at least 3 months prior to Day 1, Cycle 1. Any antihypertensive treatment containing 2 agents in combination is considered to be 2 antihypertensives.

10. Have a normal creatinine value <upper limit of normal (ULN).

11. Have a serum potassium level ≥3.5 mmol/L.

12. Have the following liver function tests:

    • Serum bilirubin <1.5 x ULN (except for subjects with documented Gilbert's disease)

    • AST or ALT <2.5 x ULN

13. Be able to swallow the study drug whole as a tablet.

14. Have a life expectancy of at least 6 months.

15. Modified per amendment.

    15.1. If the subject is heterosexually active with a woman of childbearing potential, he must agree to use a condom and another effective method of birth control during the study and for 1 week after receiving the last dose of study drug. He must agree to use a condom if having sex with a woman who is pregnant and not to donate sperm during the study.

16. Be willing and able to adhere to the prohibitions and restrictions specified in this protocol.

17. Sign an informed consent document indicating that they understand the purpose of and procedures required for the study and are willing to participate in the study.

18. Sign a separate informed consent form indicating that he agrees to provide optional blood samples for biomarker research (where local regulations permit). Refusal to give consent for the optional biomarker research sampling does not exclude a subject from participation in the study.

4.2. Exclusion Criteria

Any potential subject who meets any of the following criteria will be excluded from participating in the study. The subject will be excluded if he:

1. Has a history of pituitary or adrenal dysfunction.

2. Has an active infection or other medical condition that would contraindicate corticosteroid use.

3. Modified per amendment.

    3.1. Either:

    - Has any chronic medical condition requiring corticosteroid treatment.
    - Or has received prior corticosteroid treatment for prostate cancer.

4. Has a pathological finding consistent with small cell carcinoma of the prostate.

5. Criterion deleted per amendment.

6. Has a known brain metastasis.

7. Uses diuretics or has done so within 4 weeks of Day 1, Cycle 1.

8. Currently uses opiate analgesics for cancer-related pain, including codeine and dextropropoxyphene, or has done so anytime within 4 weeks of Day 1, Cycle 1.

9. Has received prior cytotoxic chemotherapy or biologic therapy for the treatment of CRPC.

10. Has received radiation therapy for treatment of the primary tumor within 6 weeks of Day 1, Cycle 1.

11. Modified per amendment.

    11.1. Has received radionuclide therapy for treatment of metastatic CRPC.

12. Has been previously treated with ketoconazole for prostate cancer for longer than 7 days.

13. Has received prior systemic treatment with an azole drug (eg, fluconazole, itraconazole) within 4 weeks of Day 1, Cycle 1.

14. Has received prior flutamide (Eulexin) treatment within 4 weeks of Day 1, Cycle 1 (subjects whose PSA did not decline for 3 or more months in response to antiandrogen given as a second line or later intervention will require only a 2-week washout prior to Day 1, Cycle 1).

15. Has received bicalutamide (Casodex), nilutamide (Nilandron) within 6 weeks of Day 1, Cycle 1 (subjects whose PSA did not decline for 3 or more months in response to antiandrogen given as a second line or later intervention will require only a 2-week washout prior to Day 1, Cycle 1).

16. Has active or symptomatic viral hepatitis or chronic liver disease.

17. Modified per amendment.

    17.1. Has a clinically significant heart disease as evidenced by myocardial infarction, or arterial thrombotic events in the past 6 months, severe or unstable angina, or New York Heart Association Class II-IV heart disease or cardiac ejection fraction measurement of <50%, measured within 28 days prior to Day 1, Cycle 1.

18. Has atrial fibrillation, or other cardiac arrhythmia requiring therapy.

19. Has another malignancy, except non-melanoma skin cancer, with a 30% probability of recurrence within 24 months.

20. Has diabetes mellitus.

21. Has fluid retention/edema of any grade assessed clinically.

22. Has received prior treatment with abiraterone acetate.

23. Has received an investigational drug (including investigational vaccines) or used an invasive investigational medical device within 30 days before the planned first dose of study drug or is currently enrolled in an investigational study.

24. Has known allergies, hypersensitivity, or intolerance to abiraterone acetate or its excipients (refer to Investigator's Brochure[31]).

25. Has contraindications to the use of prednisone or dexamethasone per local prescribing information.

26. Has any condition that, in the opinion of the investigator, would make participation not be in the best interest (eg, compromise the well-being) of the subject or that could prevent, limit, or confound the protocol-specified assessments.

NOTE: Investigators should ensure that all study enrollment criteria have been met at screening. If a subject's status changes (including laboratory results) after screening but before first dose of study drug is given such that they now meet an exclusion criterion, they should be excluded from participation in the study.

4.3. Prohibitions and Restrictions

Potential subjects must be willing and able to adhere to the following prohibitions and restrictions during the course of the study to be eligible for participation:

1. If heterosexually active with a woman of childbearing potential (eg, premenarchal, non-postmenopausal), he must use a condom and another effective method of birth control during the study and for 1 week after receiving the last dose of study drug. He must use a condom if having sex with a woman who is pregnant and not donate sperm during the study (see Section 4.1).

See Section 8 for prohibited concomitant therapy and permitted supportive care medications during the treatment period of the study.

5. TREATMENT ALLOCATION AND BLINDING

Treatment Allocation

Subjects will be randomized after the investigator has verified that all eligibility criteria have been met. Subjects will be randomized in a 1:1:1:1 ratio to receive abiraterone acetate with either prednisone 5 mg twice daily (Arm 1), prednisone 5 mg once daily (Arm 2), prednisone 2.5 mg twice daily (Arm 3) or dexamethasone 0.5 mg once daily (Arm 4).

Procedures for Randomization

Central randomization will be implemented in this study. Subjects will be randomly assigned to 1 of 4 treatment arms based on a computer-generated randomization schedule prepared before the study by or under the supervision of the sponsor. The interactive web response system (IWRS) will assign a unique treatment code, which will dictate the treatment assignment and matching study drug kit for the subject. The requestor must use his or her own user identification and personal identification number when contacting the IWRS, and will then give the relevant subject details to uniquely identify the subject.

Blinding

This is an open-label study.

6. DOSAGE AND ADMINISTRATION

Study medication will be administered as described in the TIME AND EVENTS SCHEDULE. During the entire treatment period, subjects will be instructed to take 1,000 mg (four 250-mg tablets) of abiraterone acetate orally, once daily, continuously, at least 2 hours after eating, and no food should be eaten for at least 1 hour after taking the tablets. Oral prednisone will be given twice daily in treatment arm 1 (one 5-mg tablet per dose) and treatment arm 3 (one 2.5-mg tablet per dose): the first dose in the morning with or immediately after a meal and the second dose after a minimum interval of 8 hours in the late afternoon or early evening, with or immediately after a meal. In treatment arm 2, oral prednisone (one 5-mg tablet) will be given once daily in the morning with or immediately after a meal. In treatment arm 4, dexamethasone (one 0.5-mg tablet) will be taken as a single dose in the morning, with or immediately after breakfast. Tablets should be swallowed whole with water.

Subjects will take study treatment until radiographic disease progression and/or unequivocal clinical progression, and/or other specific reasons for discontinuation as detailed in Section 10.2. If the subject has radiographic progression but no unequivocal clinical progression, and alternative treatment is not initiated, the subject may continue on study treatment at the Investigator's discretion (see Section 10.2). It is not required for the prednisone to be taken at the same time as abiraterone acetate. The dose of prednisone or dexamethasone cannot be increased and will remain unchanged in the event that the abiraterone acetate dose is changed. However, any subject who, during the main treatment period, requires a "stress dose" glucocorticoid when clinically indicated for a life-threatening condition will have the option of entering the extension phase (see Section 10.2). Additional systemic glucocorticoid administration such as "stress dose" glucocorticoid treatment is allowed during the extension phase if clinically indicated.

If a prednisone or dexamethasone dose is missed, it should be omitted and will not be made up. In the event of a missed daily dose of abiraterone acetate, treatment should be resumed the following day with the usual daily dose.

Study staff will instruct subjects on how to store medication for at-home use as indicated for this protocol.

Any dose/dosage adjustment should be overseen by medically qualified site personnel (preferably the principal or assigned sub-investigator) unless there is an immediate safety risk. Dose adjustments for the management of toxicities are described in Section 9.2.1.

Subjects may also take bisphosphonates or other osteoprotective agents, according to local clinical practice and the current product label. Subjects already treated with osteoprotective agents prior to the study can continue their treatment. According to the original protocol, if treatment with an osteoprotective agent was started, it must have been started prior to Day 1, Cycle 1, with the choice of osteoprotective agent at the investigator's discretion. Per protocol amendment INT-5, initiation of bisphosphonates or other approved osteoprotective agents during the study will be allowed after appropriate documentation of the bone loss, and according to local clinical practice and the current product label.

For guidance on discontinuation or dose reduction procedures for (S)AE management, refer to Section 9.2.1.

7. TREATMENT COMPLIANCE

Accurate records of all drug shipments as well as tablets dispensed and returned will be maintained. This inventory must be available for inspection by designated sponsor or regulatory authority representatives at any time. Drug supplies are to be used only in accordance with this protocol and under the supervision of the investigator. Study drug administration and dosing compliance will be assessed as specified in the TIME AND EVENTS SCHEDULE. A count of all study drug provided by the sponsor will be conducted during the treatment period.

A current and accurate account of the number of study treatment tablets the investigator received from the sponsor, dispensed to the subjects, the number of units returned to the investigator by the subject, and the number of units returned to the sponsor or its representative or destroyed on site during and at the completion of the main study treatment period and extension phase must be maintained. A detailed inventory must be completed for the study treatment.

If a subject's medication intake is not according to the protocol, the investigator will take the necessary measures to ensure future adherence to the protocol. If dosing compliance is not 100% in the absence of toxicity, subjects should be re-instructed regarding proper dosing procedures and may continue with the study. Subsequent dosing compliance procedures will be conducted at each dosing compliance assessment visit (as specified in the TIME AND EVENTS SCHEDULE). If the number of study drug doses taken by the subject is ≤75% in the absence of toxicity or disease progression, then subjects should be re-instructed regarding proper dosing

procedures. Subjects who have study drug dosing compliance of ≤75% at each dosing compliance assessment will be assessed as major protocol violations.

8. CONCOMITANT THERAPY

All therapies (prescription or over-the-counter medications, including vaccines, vitamins, herbal supplements) different from the study drug must be recorded in the eCRF for the main study treatment period and extension phase. In addition, tobacco and alcohol use will be collected.

Bisphosphonates (or other osteoprotective therapy) may be used according to local clinical practice and the current product label. Subjects already treated with osteoprotective agents prior to the study can continue their treatment. According to the original protocol, if treatment with an osteoprotective agent was started, it had to be started prior to Day 1, Cycle 1, with the choice of the osteoprotective agent at the investigator's discretion. Per protocol amendment INT-5, initiation of bisphosphonates or other approved osteoprotective agents during the study will be allowed after appropriate documentation of the bone loss, and according to local clinical practice and the current product label.

For subjects who did not undergo orchiectomy, concurrent treatment with an LHRH analogue is mandatory and must be recorded.

The following supportive care medications are considered **permissible** during the study:

- LHRH agonists or antagonists to maintain testosterone <50 ng/dL (<2.0 nM).
- Venlafaxine, selective serotonin re-uptake inhibitors, or sage extract for the management of hot flushes (a side effect of LHRH therapy).
- Conventional multivitamins, selenium and soy supplements.
- Transfusions and hematopoietic growth factors per institutional practice guidelines.

If the permissibility of a specific drug/treatment is in question, please contact the study sponsor.

Details of vitamin D use must be recorded in the eCRF.

The concurrent administration of other anticancer therapy, including cytotoxic, hormonal (except LHRH agonists or antagonists), bone-targeted alpha emitter (Radium-223), or immunotherapy is prohibited during the main study treatment period and the extension phase. Use of other investigational drug therapy for any reason is prohibited.

Concomitant therapy with any of the following listed is **prohibited** during the main study treatment period:

- 5 α-reductase inhibitor
- Chemotherapy
- Immunotherapy
- Bicalutamide, nilutamide, flutamide

- Systemic ketoconazole (or other azole drugs such as fluconazole and itraconazole)
- Diethylstilbestrol, PC-SPES, and other preparations such as saw palmetto thought to have endocrine effects on prostate cancer
- Radiopharmaceuticals such as strontium (89Sr) or samarium (153Sm)
- Spironolactone and all other diuretics
- Digoxin, digitoxin, and other digitalis drugs
- Cyproterone acetate in combination with estrogens
- Fludrocortisone acetate

The sponsor must be notified in advance (or as soon as possible thereafter) of any instances in which prohibited therapies are administered.

Additional systemic glucocorticoid administration such as "stress dose" glucocorticoid is permitted when clinically indicated for a life-threatening medical condition. If this occurs during the main study treatment period, the subject will discontinue from the main study treatment period but will be able to enter the extension phase, allowing continuation of their study treatment. "Stress dose" glucocorticoid treatment is allowed during the extension phase.

In a clinical pharmacokinetic interaction study of healthy subjects pretreated with a strong CYP3A4 inducer (rifampicin, 600 mg daily for 6 days) followed by a single dose of abiraterone acetate 1,000 mg, the mean plasma AUC_∞ of abiraterone was decreased by 55%.[31] Strong inducers of CYP3A4 (eg, phenytoin, carbamazepine, rifampicin, rifabutin, rifapentine, phenobarbital, St. John's wort [Hypericum perforatum]) during treatment are to be avoided, unless there is no therapeutic alternative. For additional information on drug-drug interactions, including the potential of abiraterone acetate to affect exposures to other medicinal products by inhibiting hepatic drug-metabolizing enzymes CYP1A2, CYP2D6 and CYP2C8, please refer to the SmPC for abiraterone acetate or the Investigator's Brochure as appropriate.[31,72]

Since androgen deprivation treatment may prolong the QT interval, caution is advised when administering abiraterone acetate with medicinal products known to prolong the QT interval or medicinal products able to induce Torsade de pointes such as class IA (e.g. quinidine, disopyramide) or class III (e.g. amiodarone, sotalol, dofetilide, ibutilide) antiarrhythmic medicinal products, methadone, moxifloxacin, or antipsychotics.[72]

During the extension phase of the study, concomitant therapy should be according to the SmPC for abiraterone acetate[72].

During the extension phase, palliative radiation will be allowed after the consultation of a multidisciplinary team; the subject will continue on study treatment at the investigator's decision and according to local guidelines. Information on palliative radiotherapy during the extension phase must be recorded in the eCRF.

9. STUDY EVALUATIONS

9.1. Study Procedures

9.1.1. Overview

The TIME AND EVENTS SCHEDULE summarizes the frequency and timing of efficacy, safety, and other measurements applicable to this study.

Screening procedures to evaluate subject eligibility for the study will be conducted within the 28 days prior to randomization. When the results of all screening assessments are known and show that the subject is eligible for inclusion, he will be randomized to a treatment arm. Study medication will be initiated within 72 hours (3 calendar days) of randomization.

Subjects in all treatment arms will be seen at specific time points during the study, and 4 weeks after termination of study medication at the EOMS visit.

The following time windows are acceptable:

- For visits up to and including Cycle 6: ± 2 days
- For visits after Cycle 6: ± 6 days
- Imaging assessments may occur up to 8 days before due Cycle number visit. Subjects should return for other procedures and dispensing of study treatment on due Cycle number visits.
- Laboratory assessments may occur up to 4 days before the cycle visit.

Visits should be based on the start date of abiraterone acetate administration. As indicated above, some flexibility in the planning of the visits is allowed; however, during the main study total duration of treatment with abiraterone acetate should be a maximum of 39 cycles (ie, 156 weeks).

In addition to the visits and assessments as detailed in the TIME AND EVENTS SCHEDULE, unscheduled visits may be planned to assess, confirm, and follow up on clinically relevant AEs or laboratory/imaging abnormalities. If an imaging procedure is performed at an unscheduled visit and progression is observed by bone scan, a confirmatory bone scan should be obtained at least 6 weeks later.

All visit-specific PRO assessments should preferably be conducted before any tests, procedures, or other consultations for that visit to prevent influencing subject perceptions.

Medical resource utilization data will be collected. Refer to Section 9.5 for details.

The maximum amount of blood drawn from each subject in this study will not exceed 420 mL. Repeat or unscheduled samples may be taken for safety reasons.

The occurrence of (S)AEs and information on concomitant therapies will be collected from signing of the ICF onwards until the EOMS or EOE visit (see Section 12.3.1 and Section 8). Survival data will be collected throughout the study including the follow-up phase.

The main study treatment period cut-off date will be in July 2016 (156 weeks after the start of treatment of the first subject participating in the study). End-of-main-study-treatment (EOMT) assessments will be performed for all subjects randomized into the main study treatment period, regardless of whether they complete study treatment up to the cut-off date or discontinue from the main study treatment period before the cut-off date. For subjects discontinuing from the main study treatment period, EOMT assessments will be performed as close as possible to the time of discontinuation and before initiation of any new anticancer treatment, including radiotherapy. For subjects who have completed the main study treatment period up to the cut-off date, EOMT assessments will be performed 4 weeks after Day 1 of the current cycle (ie, at the end of the cycle) and at the latest by 16 July 2016±15 days.

Subjects who discontinue from the main study in the absence of disease progression and subjects who are progression-free at the EOMT assessment will have the option of entering an extension phase. For subjects entering the extension phase, the day of EOMT assessments will be Day 1 of Cycle 1 of extension phase treatment. If a progression-free subject does not enter the extension phase of the study, the reason for this must be documented during the EOMT assessment and an EOMS visit will be performed 4 weeks after the last study treatment.

During the extension phase, subjects will continue their study treatment and should be monitored according to the approved SmPCs, as part of routine clinical care.[16,47,48,49,72] Data collection during the extension phase will include (S)AEs and survival information. Subjects will attend visits every 12 weeks, until radiographic disease progression and/or unequivocal clinical progression and/or other specific reasons for discontinuation as detailed in Section 10.2. An EOE visit will be performed 4 weeks after discontinuing extension treatment.

Subjects who discontinue study treatment during the main study or extension phase for any reason other than withdrawal of consent, will be followed up for survival and subsequent prostate cancer therapies, by 6-monthly telephone contact and/or chart review, until July 2018 (5 years after the start of study treatment of the first subject participating in the study).

9.1.2. Screening Phase

At screening, after signing the ICF, the subject's medical history, such as previous treatments, procedures, and conditions will be collected.

Prior prostate cancer therapies (previous hormonal, cytotoxic, and experimental treatments with start and stop dates), clinical stage and prostate cancer characteristics will be recorded. The overall eligibility of the subject to participate in the study will be assessed, including measurement of serum testosterone levels. Compliance with all individual eligibility criteria must be recorded in the source documentation.

During the screening phase, a BPI-SF (Attachment 2) will be completed and demographic data will be recorded. Body weight will be recorded and a physical examination (including height) with baseline signs and symptoms will be conducted. Also the ECOG performance status will be assessed by the investigator (Attachment 3). A complete and thorough listing will be obtained of all prescription and nonprescription (over the counter) concomitant medications currently taken

including pain medications. This will also include any nutritional supplements and/or herbal preparations.

Additionally, vital signs including seated BP, heart rate, respiratory rate, and oral, or axillary, or tympanic body temperature will be recorded and a DXA scan will give information on water retention, bone mineral density, lean body mass, and body fat distribution. A DXA scan performed according to the methods stated in the protocol, and up to 28 days prior to Day 1, Cycle 1, but before signature of the ICF, can be used as a screening assessment.

A 12-lead electrocardiogram (ECG) will be performed at screening and can be repeated as clinically indicated. ECGs performed up to 28 days prior to Day 1, Cycle 1, including assessments before signature of the ICF, can be used as screening assessments.

Blood samples for biochemistry (including K^+, Na^+, urea and creatinine) and additional laboratory tests (PSA, HOMA-IR, fasting serum lipids, and CRP) will be taken. If the subject undergoes a digital rectal exam (DRE), the PSA must be sampled prior to the DRE. Blood samples for laboratory testing must be taken ≤14 days prior to randomization (ie, ≤17 days prior to the start of study treatment). Laboratory tests performed according to standard of care ≤14 days prior to randomization but before signature of the ICF can be used as screening assessments. Refer to the TIME AND EVENTS SCHEDULE – laboratory test schedule for the timing of laboratory assessments for study eligibility.

Left ventricular ejection fraction (LVEF) must be ≥50% for study eligibility. LVEF data will be obtained by MUGA or echocardiography if MUGA is not possible, or if echocardiography is the local standard of care. Assessments performed up to 28 days prior to Day 1, Cycle 1, including assessments before signature of the ICF, can be used for screening.

Also at screening, a baseline tumor assessment will take place including a CT or MRI of the chest, abdomen and pelvis and a bone scan. Tumor burden must be evaluated by physical examination and image-based evaluation (modified RECIST, summarized in Attachment 1). Ultrasound should not be used to measure lesions that are not clinically accessible, such as liver lesions. Chest x-ray is not recommended. Scans performed according to the methods stated in the protocol, up to 28 days prior to Day 1, Cycle 1, including assessments before signature of the ICF, can be used for baseline assessments.

9.1.3. Open-Label Main Study Treatment Period

The treatment period will begin on Day 1 of Cycle 1 and will continue until all study medication is discontinued. Subjects will be randomly assigned to treatment, and must start taking study drug within 72 hours of randomization.

During the treatment period, investigators will review all clinical, laboratory and imaging data, and will use this information to make decisions about dose modification and study medication discontinuation.

PRO assessments (EQ-5D-5L, BPI-SF and FACT-P) will be performed at scheduled visits prior to all other study-related procedures planned during these visits (see TIME AND EVENTS SCHEDULE).

Safety and tolerability, including monitoring (S)AEs, clinical laboratory tests (including K^+, Na^+, urea, creatinine, plasma ACTH, serum androgen/androgen precursors profile and urine full steroid excretion profile), a urine sample for urinalysis, vital signs, DXA scans, physical examination, with clinical assessment of signs of fluid retention, and body weight, will be assessed at predefined time points as specified in the TIME AND EVENTS SCHEDULE, as well as efficacy parameters by means of ECOG, PSA test, radiologic response, survival and PRO assessments. Hematology tests including coagulation factors prothrombin time, PTT and international normalized ratio (INR), and dipstick/sediment evaluation urinalysis will only be performed at screening. Unscheduled visits during the treatment period may be performed for safety/tolerability reasons.

Optional blood samples for biomarker analyses in whole blood and plasma will be taken at the time points specified in the TIME AND EVENTS SCHEDULE – laboratory test schedule.

EOMT assessments will be performed for all subjects discontinuing from the main study treatment period before the cut-off date; such assessments will be performed as close as possible to the time of discontinuation and before initiation of any new anticancer treatment. Progression-free subjects who have completed the main study treatment period up to the cut-off date in July 2016 will also have EOMT assessments at the end of the ongoing cycle (28 days after Day 1 of the cycle), at the latest by 16 July 2016±15 days, before entering the extension phase, if applicable.

An EOMS visit will occur 4 weeks after discontinuation of study medication for any reason other than death of the subject. Safety, tolerability and efficacy data will be collected as specified in the TIME AND EVENTS SCHEDULE.

If a progression-free subject does not enter the extension phase of the study, the reason for this must be documented during the EOMT assessments and an EOMS visit will be performed 4 weeks after the last study treatment.

9.1.4. Open-Label Extension Phase

Extension phase treatment dispensing will start at the time of EOMT assessments (at the end of the last cycle of main study treatment).

During the extension phase, study visits for data collection will occur every 12 weeks. While receiving extension study treatment, subjects will be monitored for (S)AEs and for survival. However, subjects should also be medically monitored, with safety assessed, and toxicities managed according to the approved SmPCs.[16,47,48,49,72] For safety reporting during this period, refer to Section 12. Information on palliative radiotherapy during the extension phase must be recorded in the eCRF.

An EOE visit will be performed 4 weeks after the last dose of extension study treatment, during which (S)AEs, best response to abiraterone acetate, and survival will be recorded.

9.1.5. Follow-Up Phase

Following discontinuation of study treatment at any time during the study, for any reason other than withdrawal of consent, survival and subsequent prostate cancer therapies will be monitored during the follow-up phase until July 2018 (5 years after the start of study treatment of the first subject participating in the study). This information will be obtained by 6-monthly telephone contact and/or chart review, with a source data verification visit scheduled after the death of the subject or at the end of the study.

9.2. Safety

9.2.1. Evaluations

Any clinically significant abnormalities persisting at the end of the study/early withdrawal will be followed by the investigator until resolution or until a clinically stable endpoint is reached.

Safety assessments can be performed more frequently than specified in the protocol if clinically indicated; any abnormalities that are clinically significant in the opinion of the investigator will be reported as AEs in the eCRF (see Section 12).

The study will include the following evaluations of safety and tolerability according to the time points provided in the TIME AND EVENTS SCHEDULE:

Adverse Events

Any clinically significant abnormality must be reported as an AE in the eCRF. This includes any occurrence that is new in onset or aggravated in severity or frequency from the baseline condition, or abnormal results of diagnostic procedures, including laboratory test abnormalities designated as clinically significant in the opinion of the investigator. All incidences of hypokalemia (NCI CTCAE grade ≥1) and of hypertension (NCI CTCAE grade ≥2) will be reported as AEs. Unscheduled visits may be planned to assess, confirm and follow up clinically significant abnormalities.

Adverse events will be reported by the subject for the duration of the study. Adverse events will be followed by the investigator as specified in Section 12, Adverse Event Reporting. Adverse events including laboratory AEs will be graded and summarized according to the NCI CTCAE, Version 4.0.

Clinical Laboratory Tests

Blood samples for serum chemistry and hematology and a urine sample for urinalysis will be collected during the main study treatment period only. The timing of sample collection is described in the TIME AND EVENTS SCHEDULE – laboratory test schedule.

The results of screening laboratory tests must be available prior to randomization. Blood samples for screening laboratory assessments must be taken ≤14 days prior to randomization (ie,

≤17 days prior to the start of study treatment). Laboratory tests performed according to standard of care ≤14 days prior to randomization but before signature of the ICF can be used as screening assessments.

During the main study treatment period, locally-analyzed laboratory results must be available on the day of each study visit. These blood samples must be taken ≤4 days prior to each visit.

For the EOMT assessments, a laboratory test (local and/or central testing) does not need to be repeated, unless clinically indicated, if testing was performed within 2 weeks of the EOMT assessment.

The investigator must review laboratory reports throughout the study, document this review, and record any clinically relevant changes occurring during the study in the Adverse Event section of the eCRF. The following tests will be performed by the local laboratory, unless specified otherwise:

- Hematology Panel*

  - hemoglobin
  - hematocrit
  - platelet count with differential
  - red blood cell (RBC) count
  - white blood cell (WBC) count with differential
  - Prothrombin time
  - Partial thromboplastin time (PTT)
  - International normalized ratio (INR)

  **Only at screening*

- Serum Chemistry Panel

  - sodium
  - potassium
  - creatinine
  - C-reactive protein (CRP)
  - aspartate aminotransferase (AST/SGOT)
  - alanine aminotransferase (ALT/SGPT)
  - total protein
  - alkaline phosphatase (ALP)
  - total bilirubin
  - lactic acid dehydrogenase (LDH)
  - calcium
  - urea
  - albumin

Serum potassium, sodium, urea and creatinine testing can be performed more frequently than specified in the TIME AND EVENTS SCHEDULE – laboratory test schedule if clinically indicated. Regarding serum potassium levels, all incidences of hypokalemia of any grade ≥1 (NCI CTCAE v4.0) must be reported as AEs. The NCI CTCAE v4.0 grades of hypokalemia are summarized in Table 1:

Table 1: Definition of Grade ≥1 Hypokalemia (NCI CTCAE v4.0)

Grade 1	Grade 2	Grade 3	Grade 4	Grade 5
<LLN - 3.0 mmol/L	<LLN - 3.0 mmol/L; symptomatic; intervention indicated	<3.0 - 2.5 mmol/L; hospitalization indicated	<2.5 mmol/L; life-threatening consequences	Death

LLN: lower limit of normal

In addition to the testing described for this study, serum transaminases (AST, ALT) should be monitored every 2 weeks for the first 3 months of abiraterone acetate treatment and every month thereafter, and serum potassium should be monitored monthly, as part of subjects' routine clinical care, according to the approved SmPC[72]. The results of laboratory tests performed as part of subjects' routine clinical care per SmPC, which are not specified as study-related assessments in the TIME AND EVENTS SCHEDULE – laboratory test schedule, will not be recorded routinely in the eCRF. However, any resulting laboratory test abnormalities that are clinically significant in the opinion of the investigator will be captured and reported as AEs in the eCRF (see Section 12). Unscheduled visits may be planned to assess, confirm and follow up clinically significant abnormalities.

- Plasma ACTH
- Homeostatic model assessment to quantify insulin resistance (HOMA-IR: fasting plasma glucose, fasting serum insulin) and HbA1c.

  HOMA-IR is calculated using the following formula:

  fasting plasma glucose (mmol/L) times fasting serum insulin (mU/L) divided by 22.5.
- Fasting serum lipids (cholesterol, triglycerides, high density lipoprotein and low density lipoprotein)
- Urinalysis*

**Dipstick***	Sediment (if dipstick result is abnormal)
- glucose	- RBC
- protein	- WBC
- blood	- epithelial cells
	- crystals
	- casts
	- bacteria

If the dipstick result is abnormal, or for study centers where dipstick results are not available, sediment will be evaluated according to local practice.

* *Only at screening*

All laboratory tests, except for the serum androgen/androgen precursors profile and urine full steroid excretion profile, and biomarker analysis, should be performed at the laboratory facilities associated with the investigational site, unless the facility does not fulfill the protocol requirement for the laboratory tests. Laboratory certificates or accreditation and normal ranges of the laboratory facility at the site must be submitted before enrollment of any subject at the site. If the subject has their laboratory assessments conducted at a laboratory facility other than the

facility associated with the investigational site, then the investigator must submit laboratory certificates or accreditation and normal ranges for that facility as well.

The following analyses will be performed at central laboratories:

- Serum DHEA, DHT, testosterone, DHEA-S and androstenedione. Serum androgens will be measured by liquid chromatography-mass spectrometry (LC-MS). Frozen aliquots will be sent to a centralized laboratory in batches.

- 24 hour urine

  - DHEA
  - DHT
  - testosterone
  - DHEA-S
  - androstenedione

  Full steroid excretion profile will be measured by gas chromatography-mass spectrometry (GC-MS). Frozen aliquots will be sent to a centralized laboratory in batches. 24h urine volume needs to be recorded prior to taking the aliquots.

Electrocardiogram (ECG)

Electrocardiogram recording will be performed for screening purposes only. Repeat measurement will be at the discretion of the investigator, if clinically indicated. ECGs performed up to 28 days prior to Day 1, Cycle 1, including assessments before signature of the ICF, can be used as screening assessments.

During the collection of ECGs, subjects should be in a quiet setting without distractions (eg, television, cell phones). Subjects should rest in a supine position for at least 5 minutes before ECG collection and should refrain from talking or moving arms or legs. If blood sampling or vital signs measurement is scheduled for the same time point as ECG recording, the procedures should be performed in the following order: ECG(s), vital signs, blood draw.

Vital Signs (oral, or axillary or tympanic temperature, pulse/heart rate, respiratory rate, blood pressure)

Blood pressure ([BP] systolic and diastolic) and heart rate measurements will be assessed seated as 3 consecutive measurements using a completely automated device. Manual techniques will be used only if an automated device is not available. Each BP and heart rate measurement should be preceded by at least 5 minutes of rest in a quiet setting without distractions (eg, television, cell phones). The lowest of the 3 consecutive BP readings will be reported.

In addition to the time points in the TIME AND EVENTS SCHEDULE, vital signs measurements can be performed more frequently if clinically indicated. In addition to the testing described for this study, blood pressure should be monitored monthly, as part of subjects' routine clinical care, according to the approved SmPC.[72]

Hypertension grade ≥2 (NCI CTCAE v4.0), as summarized in Table 2, will be reported as an AE.

Table 2: Definition of Grade ≥2 Hypertension (NCI CTCAE v4.0)

Grade 2	Grade 3	Grade 4	Grade 5
Stage 1 hypertension (systolic BP 140-159 mm Hg or diastolic BP 90-99 mm Hg); medical intervention indicated; recurrent or persistent (≥24 hrs); symptomatic increase by >20 mm Hg (diastolic) or to >140/90 mm Hg if previously WNL; monotherapy indicated	Stage 2 hypertension (systolic BP ≥160 mm Hg or diastolic BP ≥100 mm Hg); medical intervention indicated; more than one drug or more intensive therapy than previously used indicated.	Life-threatening consequences (eg, malignant hypertension, transient or permanent neurologic deficit, hypertensive crisis); urgent intervention indicated	Death

WNL: within normal limits

Physical Examination

Evaluations should be performed by the same evaluator throughout the main study period whenever possible. If it is not possible to use the same evaluator to follow the subject, then evaluations should overlap (ie, examine the subject together and discuss findings) for at least one visit.

Physical examination includes head, eyes, ears, nose, and throat, chest, cardiac, abdominal, extremities, neurologic, and lymph node examinations. Height will be recorded at screening only.

Fluid Retention/Edema

Subjects will be assessed for signs of fluid retention/edema during physical examination at every main study visit. Fluid retention will also be evaluated during DXA scans (see below).

Body Weight

Weight will be recorded at every main study visit using a consistent methodology throughout the study for each subject.

Dual-Energy X-Ray Absorptiometry (DXA) Scan

The DXA scans will give information on water retention, bone mineral density (osteopenia), sarcopenia, and body fat distribution.

Guidelines on usage of DXA scan machines and procedures will be provided in a separate protocol-specific manual.

A DXA scan performed according to the methods stated in the protocol, and up to 28 days prior to Day 1, Cycle 1, but before signature of the ICF, can be used as a screening assessment.

Management of Specific Toxicities with Abiraterone Acetate

The safety monitoring and toxicity management plan described below takes into account AEs based on the clinical safety data of abiraterone acetate.

Hypokalemia

Hypokalemia of any grade should be reported as an AE. At the initial observation of grade 1 hypokalemia (serum potassium <3.5 mM or below lower limit of normal range, but ≥3.0 mM), oral potassium supplement will be initiated. The dose of potassium supplement must be carefully titrated to maintain serum potassium at ≥3.5 mM but ≤5.0 mM. Any subject with low potassium while on study or a history of hypokalemia from a pre-existing or concurrent medical condition will undergo weekly or more frequent laboratory electrolyte evaluation. The investigator should consider maintaining the subject’s potassium level at ≥4.0 mM in these subjects.

If any subject experiences grade 3 hypokalemia (serum potassium levels <3.0 mM–2.5 mM, NCI CTCAE v4.0) or life-threatening hypokalemia with potassium levels <2.5 mM (NCI CTCAE v4.0 hypokalemia grade 4), study treatment will be withheld and the subject will be hospitalized for intravenous potassium replacement and cardiac monitoring. Subjects who experience grade 3 or grade 4 hypokalemia will be discontinued from the main study treatment period. However, these subjects will have the option of entering an extension phase, allowing continuation of their study treatment until disease progression or death if earlier, after normalization of the subject's condition (grade ≤1) if, in the opinion of the investigator, the subject will benefit from continuation of study treatment.

Hypertension

Hypertension grade ≥2 should be reported as an AE.

- Grade 1-2: Management per investigator. No study treatment dose reduction.
- Grade 3-4: Hold study treatment. Adjust or add medications to mitigate the toxicity. When hypertension resolves to ≤grade 1, resume study treatment at full dose.
- If toxicity recurs, hold study treatment, and adjust or add medications to mitigate the toxicity. When resolved to ≤grade 1, resume study treatment with the first dose level reduction (3 tablets, 750 mg of study treatment).
- If toxicity recurs, hold study treatment, and adjust or add medications to mitigate the toxicity. When resolved to ≤grade 1, resume study treatment with the second dose level reduction (2 tablets, 500 mg of study treatment).
- If toxicity recurs despite optimal medical management and 2 dose level reductions, discontinue study treatment.

Fluid retention/edema

- If diuretics are required, participation in the main study has to be discontinued. Study treatment may be continued during the extension phase, after normalization of the subject's condition (grade ≤1) if, in the opinion of the investigator, the subject will benefit from continuation of study treatment.

- Pedal edema: supportive management per investigator. No study treatment dose reduction.
- Anasarca and/or pulmonary edema requiring supplemental oxygen: hold study treatment. Adjust or add medications to mitigate the toxicity. When toxicity resolves to ≤grade 1, resume study treatment at full dose.
- If toxicity recurs, hold study treatment, and adjust or add medications to mitigate the toxicity. When resolved to ≤grade 1, resume study treatment with the first dose level reduction (3 tablets, 750 mg of study treatment).
- If toxicity recurs again, hold study treatment, and adjust or add medications to mitigate the toxicity. When resolved to ≤grade 1, resume study treatment with the second dose level reduction (2 tablets, 500 mg of study treatment).
- If toxicity recurs despite optimal medical management and 2 dose level reductions, discontinue study treatment.

Abnormal liver function tests

- If grade 1 increases in AST, ALT or bilirubin occur (eg, increase in AST or ALT from ULN to 2.5 x ULN; increase in total bilirubin from ULN to 1.5 x ULN): the frequency of liver function test monitoring should be increased, if the investigator judges that the laboratory abnormalities are potentially related to study medication. No study treatment dose reduction is required.
- If grade 2 increases in AST, ALT or bilirubin occur (eg, increase in AST or ALT to >2.5-5 x ULN; increase in total bilirubin from >1.5-3 x ULN): the frequency of liver function test monitoring should be increased to ≥ once a week, if the investigator judges that the laboratory abnormalities are potentially related to study medication. No study treatment dose reduction is required.
- If grade 3 or higher increases in AST, ALT, or bilirubin occur (eg, increase in AST or ALT to >5 x ULN; increase in total bilirubin to >3 x ULN), hold study medication and all other concomitant medications that are potentially hepatotoxic. Frequent laboratory evaluations (at least once weekly) should be conducted until the liver function tests return to baseline value or grade 1. Liver enzyme measurements should be made immediately, regardless of when the next study visit or monitoring interval is scheduled.
  - If study treatment resumption is considered for subjects who have experienced grade 3 increases in AST, ALT, or bilirubin, and the Medical Monitor agrees, resume study treatment with the first dose level reduction (3 tablets, 750 mg of study treatment) when grade 3 toxicities resolve to grade 1 or baseline.
  - If grade 3 or higher increases in AST, ALT, or bilirubin recur after the first dose reduction hold study medication and all other concomitant medications that are potentially hepatotoxic. Frequent laboratory evaluations should be conducted (at minimum weekly) until the liver function tests return to baseline value or grade 1. Liver enzyme measurements should be made immediately, regardless of when the next study visit or monitoring interval is scheduled.
  - If study treatment resumption is considered for subjects who have experienced grade 3 increases in AST, ALT, or bilirubin with the first dose reduction, and the Medical Monitor agrees, resume study treatment with the second dose level reduction (2 tablets,

500 mg of study treatment) when AST, ALT, or bilirubin returns to baseline value or grade 1.

- If grade 4 increases in AST, ALT, or bilirubin occur (eg, increase in AST or ALT to >20 x ULN; increase in total bilirubin to >10 x ULN), subjects must discontinue study treatment immediately and will not be re-challenged. They should be followed until resolution of abnormal liver function tests.

Non-mineralocorticoid-based side effects

- If grade 1 to 2 toxicities occur, give supportive care per institutional guidelines. There should be no study treatment dose reduction.
- If grade 3 or higher toxicities including headache (interferes with activities of daily living), nausea (total parenteral nutrition [TPN], intravenous fluid [IVF]), vomiting (>6 episodes/24 hours, TPN or IVF), diarrhea (IVF, hospitalization, hemodynamic collapse), or any other toxicity judged to be related to study treatment is observed where the subject's safety is jeopardized, hold study treatment.
- When toxicity resolves to ≤grade 1, resume study treatment at full dose.
- If toxicity recurs, hold study treatment, and adjust or add medications to mitigate the toxicity. When resolved to ≤grade 1, resume study treatment with the first dose level reduction (3 tablets, 750 mg of study treatment).
- If toxicity recurs, hold study treatment, and adjust or add medications to mitigate the toxicity. When resolved to ≤grade 1, resume study treatment with the second dose level reduction (2 tablets, 500 mg of study treatment).
- If toxicity recurs despite aggressive medical management and two dose level reductions, discontinue study treatment.

Dose-reduction procedure for (S)AE management

In the event where dose-reduction is used for (S)AE management, 2 dose reductions are allowed. At each dose reduction, one tablet of abiraterone acetate will be removed, eg, 4→3 tablets, and 3→2 tablets.

Management of Specific Toxicities with Prednisone and Dexamethasone

Management of (S)AEs associated with the use of prednisone or dexamethasone will be at the discretion of the investigator, taking into account generally accepted medical standards for patient monitoring and management, and the investigator's clinical judgment and clinical practice. The manufacturer's prescribing information for prednisone and dexamethasone should be consulted.[16,47,48,49] However, if a subject requires an increased dose of prednisone or dexamethasone as a "stress dose" glucocorticoid, this subject will be discontinued from participation in the main study. In such cases, it will be possible to continue study treatment during the extension phase. These subjects will have the option of entering an extension phase, allowing continuation of their study treatment until disease progression or death if earlier, after normalization of the subject's condition (grade ≤1) if, in the opinion of the investigator, the subject will benefit from continuation of study treatment. After normalization of the subject's condition, the subject should, at the discretion of the investigator, continue in the extension phase on the same dose of prednisone or dexamethasone as in the main study treatment period.

9.2.2. Endpoints

Primary Endpoint

The primary endpoint "No mineralocorticoid excess" is defined as experiencing (yes or no) neither of the 2 mineralocorticoid excess toxicities, ie, neither hypokalemia nor hypertension, during the first 24 weeks of treatment. This will be derived from treatment-emergent AE data, which will be defined using MedDRA and graded according to NCI CTCAE v4.0.

Secondary Endpoints

Global safety profile according to the NCI CTCAE v4.0, including the incidence of mineralocorticoid excess toxicities (eg, hypokalemia and hypertension), and incidence of mid-term and long-term exogenous glucocorticoid side effects during the entire study.

9.3. Efficacy

9.3.1. Evaluations

The following efficacy evaluations will be performed as indicated in the TIME AND EVENTS SCHEDULE:

ECOG

The ECOG Performance Status is a grade scale to measure QoL. Scores run from 0 to 5, with 0 denoting perfect health and 5 denoting death. Any changes in ECOG Performance Status will be confirmed at the subject's next visit.

PSA test

Blood samples will be drawn to assess the PSA level. This will be done at the times shown in the TIME AND EVENTS SCHEDULE – laboratory test schedule, every 4 weeks up to and including Cycle 6 and every 12 weeks thereafter during the main study treatment period.

Radiologic response

Radiographic disease will be measured using the same modality (CT scan, MRI or bone scan) during the study. CT scans, MRI or bone scans performed up to 28 days prior to Day 1, Cycle 1, including assessments before signature of the ICF, can be used for baseline assessments, provided these have been performed according to the methods specified in the protocol. During the study, radiologic assessments will be performed every 12 weeks, or as clinically indicated if earlier.

For the EOMT assessment, a complete tumor assessment does not need to be repeated if this has been performed within 8 weeks of the EOMT assessment, unless it is clinically indicated.

Any evidence of soft tissue progression as defined in the modified RECIST,[19] including assessments performed according to local clinical practice, will be documented in the eCRF during the EOMT assessment.

Best Response

Best response to study treatment will be recorded at the EOE visit. Best response to each subsequent therapy will be recorded, if applicable.

EQ-5D-5L

The EQ-5D-5L (Attachment 4) is a frequently used generic instrument used to measure health-related QoL such as mobility, self-care, usual activities, pain, discomfort, and anxiety/depression.

BPI-SF

The BPI-SF is a validated, widely used, self-administered questionnaire developed to assess the severity of pain and the impact of pain on daily functions (Attachment 2).

The BPI-SF includes 4 items measuring the intensity of pain which make up the pain intensity subscales, 7 items that assess how much pain has interfered with 7 daily activities which make up the pain interference subscale, and an additional item on the extent of pain relief. The intensity of pain is assessed with 4 items using 11-point numerical rating scales from “0” = no pain to “10” = pain as bad as you can imagine. As pain may vary during the day, the intensity is rated at the time of completing the questionnaire (right now) and at its worst, least, and on average over the past day. Scores for the intensity items can be reported individually or averaged for a pain intensity subscale score. The pain interference items are scored by the respondent on a scale of 0, “Does not interfere” to 10, “completely interferes.” This subscale is typically scored as the mean of the 7 interference items. Scores for both the pain intensity and pain interference subscales range from 0 to 10, where higher scores indicate higher severity of pain.

For study eligibility, a score of 0-1 on BPI-SF Question #3 (worst pain in last 24 hours) will be considered asymptomatic.

FACT-P

The FACT-P questionnaire Version 4.0 (Attachment 5) will be utilized to assess functional status. The FACT-P is a multidimensional, self-reported QoL instrument specifically designed for use with prostate cancer patients. It consists of 27 core items that assess patient function in 4 domains: Physical, Social/Family, Emotional, and Functional well-being, which is further supplemented by 12 site-specific items to assess for prostate-related symptoms. Each item is rated on a 0 to 4 Likert type scale, and then combined to produce subscale scores for each domain, as well as a global QoL score. Higher scores represent better QoL.

Survival and Subsequent Prostate Cancer Therapies

Survival data will be collected throughout the main study treatment period and extension phase.

During the follow-up phase, there will be follow-up for survival and for subsequent prostate cancer therapies for all subjects discontinuing study treatment before the end of the study. Best response to each subsequent therapy will be recorded during follow-up. Follow-up information will be obtained by 6-monthly telephone contact and/or chart review, with a source data

verification visit scheduled after the death of the subject or at the end of the study. If the follow-up information is obtained via telephone contact, written documentation of the communication must be available for review in the source documents.

9.3.2. Endpoints

- Progression-free survival (PFS) is defined as the time from randomization to the occurrence of one of the following: radiographic progression, clinical progression or death.
  - *Radiographic progression* is based on PCWG2 criteria and modified RECIST as the time from randomization to the occurrence of one of the following:
    1. A patient is considered to have progressed by bone scan if:
       a. The first bone scan with ≥2 new lesions compared to baseline is observed <12 weeks from randomization and is confirmed by a second bone scan taken ≥6 weeks later showing ≥2 additional new lesions (a total of ≥4 new lesions compared to baseline).
       b. The first bone scan with ≥2 new lesions compared to baseline is observed ≥12 weeks from randomization and the new lesions are verified on the next bone scan ≥6 weeks later (a total of ≥2 new lesions compared to baseline).
    2. Progression of soft tissue lesions measured by CT or MRI as defined in modified RECIST[19].

       Any other evidence of progression of soft tissue lesions as defined in the modified RECIST, including assessments performed according to local clinical practice, will also be documented in the eCRF during the EOMT assessment.
  - *Clinical progression* will be characterized as:
    1. Cancer pain requiring initiation of chronic administration of opiate analgesia (oral opiate use for ≥3 weeks; parenteral opiate use for ≥7 days).

       Subjects with cancer pain requiring opiate analgesia for relief should also be assessed by the investigator for the need for initiating systemic chemotherapy.

       Or
    2. Immediate need to initiate cytotoxic chemotherapy or the immediate need to have either radiation therapy or surgical intervention for complications due to tumor progression, even in the absence of radiographic evidence of disease progression.

       Or
    3. Deterioration in ECOG performance status to grade 3 or higher.
  - *Death from any cause*
- PSA response rate (PCWG2 criteria, Attachment 6). A PSA response is defined as a ≥50% decline from baseline according to the adapted PCWG2 criteria. For a PSA response to be confirmed, an additional PSA measurement obtained 4 or more weeks later has to show ≥50% decline from baseline.
- Time to PSA progression (by PCWG2 criteria)

- Objective response rate in subjects with measurable disease (modified RECIST, v1.1)
- Time to opiate use for cancer pain
- Time to deterioration in ECOG performance score by 1 point
- Change in prostate cancer-related PRO measures: EQ-5D-5L, BPI-SF, FACT-P
- Overall survival
- Subsequent anticancer therapies (time to next therapy for prostate cancer, time to initiation of chemotherapy, treatment duration and best response) until death or study close

9.4. Biomarkers

For subjects consenting to participate in the optional biomarker research phase, 16 mL of peripheral venous blood will be collected in two 8-mL cryopreservation tubes (CPT) and 2.5 mL in a 5-mL PAXgene™ tube at Cycle 1 Day 1, Cycle 2 Day 1 and at EOMT. Both CPTs will be processed for plasma collection within 2 hours of being drawn and plasma immediately frozen at -80ºC. One tube will be used for extraction of circulating DNA and the other for circulating RNA. Once drawn, the PAXgene™ tubes will kept at 18 to 23 º C (ambient) for not less than 2 hours and not more than 24 hours and then frozen at -80 ºC. Whole blood RNA will be isolated and purified using the PAXgene™ Blood RNA Kit according to the manufacturer's instructions. Detailed procedures for handling and storage of the samples are described in the Laboratory Manual. Samples for biomarker research will only be stored until analysis has been completed (see Section 16.2.5).

Genomic aberrations increase in prevalence with the development of drug resistance and could underlie resistance to hormone therapies.[25] Sequential tumor biopsies from large cohorts of patients treated with abiraterone are challenging to obtain. Furthermore, a biopsy of a single lesion does not take into account inter-metastases heterogeneity. Circulating plasma DNA and RNA and whole blood RNA will therefore be evaluated. Several groups have now confirmed the feasibility of next-generation sequencing on circulating plasma DNA and RNA[23,57]. These studies will aim to identify genomic aberrations (point mutations, copy number aberrations) not present in germ-line samples that occur pre-treatment (predict response or primary resistance to abiraterone acetate) or appear on treatment (and associate with secondary resistance to abiraterone acetate). Olmos et al have shown that whole-blood RNA profiling could identify more aggressive cancers that could require the early initiation of treatment[43].

MicroRNA (miRNAs) have been widely studied in prostate cancer and are small noncoding RNAs that regulate the expression of protein-coding genes by modulating both mRNA stability and translation[28,38]. Alteration of miRNA expression levels can alter cell function and induce cellular transformation leading to cancer[34]. A number of dysregulated miRNAs have been associated with different prostate cancer stages and some miRNAs are consistently dysregulated at early and advanced stages of disease or associated with more aggressive disease. Since some miRNAs are androgen-controlled,[1,36,46,62,63,68,70] it is hypothesised that dysregulated miRNAs patterns in baseline samples may predict response to drugs such as abiraterone acetate. Changes

in miRNA expression levels from baseline may allow elucidation of mechanisms leading to resistance.

Dysregulation of steroid synthesis has been previously reported in xenograft models treated with abiraterone acetate and evaluation of steroid profiles may complement miRNA studies[40]. An analysis of dysregulated steroid transcript levels by gene expression profiling will investigate the predictive profiles found in these previously reported studies. Data collected from this study will be compared to data obtained from prior studies in asymptomatic metastatic castration-resistant prostate cancer to identify miRNA and gene expression profiles (GEPs) that correlate with response (or primary resistance) to abiraterone acetate. The biomarker results from this study will then be used to inform future studies of anti-androgen therapies, possibly leading to product differentiation by selection of responsive subjects.

MicroRNA (miRNAs) that correlate with high risk prostate cancer[1,68,70,44] have also been identified. Since it is difficult to histologically distinguish high risk from benign disease and because PSA velocity and doubling time may be an unreliable measure of disease aggression, the aim is to investigate whether miRNA profiles may better define high risk prostate cancer in the early metastatic disease setting. These data may then be utilized in selection of high risk patients in future studies if these previously reported miRNA profiles are confirmed and found to be more sensitive than conventional clinical estimates of high risk disease.

9.5. Medical Resource Utilization

Medical resource utilization data associated with medical encounters will be collected in the eCRF by the investigator and staff for all subjects throughout the main study treatment period, as shown in the TIME AND EVENTS SCHEDULE. Protocol-mandated procedures, tests, and encounters are excluded. The data collected may be used to conduct exploratory economic analyses and will include:

- Number and duration of medical care encounters, including surgeries, and other selected procedures (inpatient and outpatient)
- Duration of hospitalization (total days length of stay, including duration by wards; eg, intensive care unit)
- Number and character of diagnostic and therapeutic tests and procedures
- Outpatient medical encounters and treatments (including physician or emergency room visits, tests and procedures, and medications)

9.6. Sample Collection and Handling

The actual dates and times of sample collection must be recorded in the eCRF or laboratory requisition form. Refer to the TIME AND EVENTS SCHEDULE – laboratory test schedule for the timing and frequency of all sample collections.

The serum androgen/androgen precursor profile and urine full steroid excretion profile, and biomarkers will be analyzed separately by central laboratories.

Instructions for the collection, handling, and shipment of samples are found in the laboratory manual that will be provided for sample collection and handling. Samples will be analyzed at a facility meeting regulatory requirements and/or using methods documented in a methods validation report. All shipping requirements and storage conditions will be provided in a protocol-specific laboratory manual.

10. SUBJECT COMPLETION/WITHDRAWAL

10.1. Completion

A subject will be considered to have completed the main study treatment period if he has completed assessments up until the end-of-main-study-treatment cut-off date (156 weeks [3 years] after the start of study treatment for the first subject participating in the study), or until progression of disease (unequivocal clinical progression or radiographic progression), or has experienced a clinical endpoint that precludes further study participation. Subjects who prematurely discontinue main study treatment for any other reason will not be considered to have completed the main study treatment period.

A subject will be considered to have completed the entire study, including extension and follow-up phases, if he has completed the last study assessment up until July 2018 (5 years after the start of study treatment of the first subject), or until death if earlier.

10.2. Discontinuation of Treatment

If a subject's study treatment must be discontinued before the end of the treatment regimen, this will not result in automatic withdrawal from the study.

During the main study and extension phase, a subject's study treatment should be discontinued if:

- The investigator believes that for safety reasons (eg, AE) it is in the best interest of the subject to stop treatment.
- Sustained side effects: subjects who have sustained toxicities that do not return to NCI CTCAE (v4.0) grade 1 or less with appropriate medical management, should be discontinued from the study treatment.
- The investigator permanently changes the study treatment regimen, ie dose or schedule or type of a glucocorticoid (during the extension phase only, a “stress dose” of glucocorticoid is allowed when clinically indicated for a life-threatening condition).
- Initiation of new anticancer treatment (see Section 8): subjects will be discontinued from the study treatment when the investigator determines new treatment for prostate cancer is warranted. For subjects who did not undergo orchiectomy, concurrent treatment with LHRH analogue is mandatory and must be recorded. The concurrent administration of other anticancer therapy, including cytotoxic, hormonal (except LHRH agonists or antagonists), bone-targeted alpha emitter (Radium-223), or immunotherapy is prohibited during the treatment period. Use of other investigational drug therapy for any reason is prohibited.

- Unequivocal clinical progression without radiographic progression:

  Confirmed radiographic progression may be a reliable indicator of clinical benefit in patients with CRPC[55]. Subjects should ordinarily be maintained on study treatment until confirmed radiographic progression. If the subject has radiographic progression but no unequivocal clinical progression and alternate treatment is not initiated, the subject may continue on study treatment, at the investigator's discretion.

  During the extension phase, palliative radiation will be allowed after the consultation of a multidisciplinary team; the subject will continue on study treatment at the investigator's decision and according to local guidelines.

  However, if subjects have unequivocal clinical progression without radiographic progression, these subjects are indicated for the current standard of care. Study treatment should be stopped and subjects advised regarding available treatment options.

  For this study, unequivocal clinical progression will be characterized as previously detailed in Section 9.3.2.

- Radiographic progression, as previously detailed in Section 9.3.2.

Study treatment will be continued for subjects who have increasing PSA values in the absence of radiographic or unequivocal clinical progression. Although serial PSA's will be measured during this study, progression or change in PSA values is not considered a reliable measure of disease progression, and should not be used as an indication to discontinue study treatment.

The following specific criteria have been defined for discontinuation from the main study treatment period only:

- Grade 3 hypokalemia (serum potassium levels <3.0 mM – 2.5 mM, NCI CTCAE v4.0) or life-threatening hypokalemia with potassium levels <2.5 mM (NCI CTCAE v4.0 hypokalemia grade 4).
- If treatment with diuretics is required.
- If an increased dose of glucocorticoids is required as a "stress dose" (when clinically indicated for a life-threatening condition).
- If palliative radiation is required.

However, these subjects will have the option of entering the extension phase, allowing continuation of their study treatment until disease progression or death if earlier, after normalization of the subject's condition (grade ≤1) if, in the opinion of the investigator, the subject will benefit from continuation of study treatment.

Assessments After Study Treatment Discontinuation

Subjects discontinuing study treatment during the main study treatment period should have the end-of-main-study-treatment (EOMT) assessments shown in the TIME AND EVENTS SCHEDULE, incorporating assessments of radiographic progression, including a confirmatory bone scan, as appropriate. EOMT assessments will be performed as close as possible to the time of discontinuation and before initiation of any new anticancer treatment, including radiotherapy. These subjects will also have end-of-main-study (EOMS) assessments 4 weeks after finishing study treatment.

Subjects discontinuing study treatment during the extension phase of the study will have EOE assessments to monitor [S]AEs, record best response to abiraterone acetate, and record survival 4 weeks after finishing extension study treatment.

All subjects discontinuing study treatment at any time during the study will be followed up for survival and for information on subsequent prostate cancer therapy until July 2018 (5 years after the start of study treatment of the first subject participating in the study).

10.3. Withdrawal From the Study

A subject will be withdrawn from any phase of the study for any of the following reasons:

- Lost to follow-up
- Withdrawal of consent
- Death

If a subject is lost to follow-up, every reasonable effort must be made by the study site personnel to contact the subject and determine the reason for discontinuation/withdrawal. The measures taken to follow up must be documented.

When a subject withdraws before completing the study, the reason for withdrawal is to be documented in the eCRF and in the source document. Study drug assigned to the withdrawn subject may not be assigned to another subject. Subjects who withdraw will not be replaced.

A subject who withdraws from the study will have the following options regarding the blood samples for the optional biomarker research phase:

- The collected samples will be retained and used in accordance with the subject's original separate informed consent for optional research samples.
- The subject may withdraw consent for optional research samples, in which case the samples will be destroyed and no further testing will take place. To initiate the sample destruction process, the investigator must notify the sponsor study site contact of withdrawal of consent for the optional research samples and to request sample destruction. The sponsor study site contact will, in turn, contact the biomarker representative to execute sample destruction. If requested, the investigator will receive written confirmation from the sponsor that the samples have been destroyed.

Withdrawal From the Optional Biomarker Research Phase While Remaining in the Main Study

The subject may withdraw consent for optional biomarker research blood samples while remaining in the study. In such a case, the optional research samples will be destroyed. The sample destruction process will proceed as described above.

11. STATISTICAL METHODS

Statistical analysis will be done by the sponsor or under the authority of the sponsor. A general description of the statistical methods to be used to analyze the efficacy and safety data is outlined below. Specific details will be provided in the Statistical Analysis Plan.

Baseline for all analyses will be the day of randomization unless otherwise specified.

The primary analysis will be done when all subjects have completed the first 24 weeks of the main study treatment period and will be based on the data of those first 24 weeks only. It will focus on the primary endpoint. In addition, other efficacy and safety data, which will be specified in the Statistical Analysis Plan, will be analyzed. The main analysis will be performed upon completion of the main study treatment period, when EOMT assessments have been completed for all subjects. This main analysis will be based on all data for the main study treatment period and will analyze all parameters assessed during that period. The final analysis will be performed when all subjects have completed the study and will include data for the extension phase and follow-up phase.

All continuous variables will be summarized using descriptive statistics, which will include the number of patients, mean, standard deviation, median, minimum, maximum, and 95% confidence interval (CI). All categorical variables will be summarized using frequencies and percentages.

11.1. Subject Information

For all subjects who sign an ICF and are not randomly assigned to study drug descriptive demographic statistics will be provided.

The intent-to-treat (ITT) population is defined as all randomized subjects regardless of whether they received any study treatment. All efficacy and safety analyses will be performed on the ITT population. Additionally, where appropriate, the safety analysis will be performed including only randomized subjects who receive at least 1 dose of abiraterone acetate plus prednisone or dexamethasone and have at least 1 post-baseline safety assessment (modified intent-to-treat [mITT] analysis set).

If there are a substantial number of protocol violators (eg, more than 10%), an additional per-protocol analysis may be performed.

11.2. Sample Size Determination

The primary parameter is defined as experiencing (yes or no) neither of the 2 mineralocorticoid excess toxicities during the first 24 weeks of treatment, ie, neither hypokalemia nor hypertension (see Section 11.3). The COU-AA-302[52] study indicated that 81% of the subjects did not experience hypokalemia and/or hypertension within 6 months of the study start. However, for the present study, there is greater focus on hypokalemia and hypertension, which may result in higher reporting of these events. Therefore, for this study the assumption is that 75% of the subjects within each treatment arm will not experience any of the events defined in the primary

parameter, ie, hypokalemia and/or hypertension, during 24 weeks of treatment. Fifty percent is defined as the highest percentage to reject the treatment. An exact binomial test with a 5% one-sided significance level will have 89% power to detect the difference between 50% and 75% when the sample size is 30. Assuming 15% of non-evaluable subjects, 36 subjects per treatment arm will be included.

11.3. Safety Analyses

All safety analyses will be based on the ITT population.

Adverse Events

The verbatim terms used in the eCRF by investigators to identify AEs will be coded using the MedDRA. All reported AEs with onset during the treatment period (ie, treatment-emergent AEs, and AEs that have worsened since baseline), which will include pre-existing medical conditions that have worsened since baseline, will be included in the analysis.

Criteria

Primary Endpoint

The primary parameter is defined as experiencing (yes or no) neither of the 2 mineralocorticoid excess toxicities during the first 24 weeks of treatment, ie, neither hypokalemia nor hypertension. This will be derived from treatment-emergent AE data, which will be defined using MedDRA and graded according to the NCI CTCAE v4.0. The number and percentage of subjects experiencing neither of the 2 mineralocorticoid excess toxicities during the first 24 weeks of treatment will be summarized descriptively per treatment arm and the corresponding 95% CI will be displayed.

Secondary Endpoints

The verbatim terms used in the eCRF by investigators to identify AEs will be coded using MedDRA and AEs will be graded according to the NCI CTCAE v4.0. Incidence of AEs, experienced during the first 24 weeks of treatment as well as during the entire study, will be summarized by system organ class (SOC) and preferred term (PT), and will be presented by treatment arm and overall. Adverse events will be summarized by grade, according to the worst grade experienced. In addition, most frequently observed AEs will be summarized by treatment arm. In the summary of AEs, an AE occurring more than once within a SOC and PT will be counted only once using the worst grade experienced.

Summaries, listings, datasets, or subject narratives may be provided, as appropriate, for those subjects who die, who discontinue treatment due to an AE, or who experience a severe or an SAE.

Clinical Laboratory Tests

Laboratory data will be summarized by type of laboratory test. Reference ranges and markedly abnormal results (specified in the Statistical Analysis Plan) will be used in the summary of laboratory data. Descriptive statistics will be calculated for each laboratory analyte at baseline

and at each scheduled time point. Changes from baseline results will be presented in pre- versus post-treatment cross-tabulations (with classes for below, within, and above normal ranges). The laboratory data for subjects with any post-baseline result outside the reference range will be summarized, when appropriate, by use of the NCI CTCAE Grade, v4.0. A listing of subjects with any laboratory results outside the reference ranges will be provided. A listing of subjects with any markedly abnormal laboratory results will also be provided.

The impact of study treatment on adrenal function will be assessed by measurement of serum and urinary androgens and their precursors (DHEA, DHEA-S, androstenedione, testosterone and DHT). These data will be analyzed externally.

Vital Signs

Descriptive statistics of pulse and BP (systolic and diastolic) values and changes from baseline will be summarized at each scheduled time point. The percentage of subjects with values beyond clinically important limits will be summarized.

Physical Examination

Descriptive statistics of changes from baseline will be summarized at each scheduled time point.

11.4. Efficacy Analyses

Criteria

Secondary Endpoints

All efficacy analyses will be based on the ITT population. Changes from baseline and observed values for continuous/ordinal efficacy data (eg, ECOG score) will be summarized descriptively at each assessment time point during the study and at the subject's last efficacy evaluation (endpoint). Summary tabulations will be presented that will display the number of observations, mean, standard deviation, median, minimum and maximum and 95% CIs by treatment arm.

Time-to-event type of analyses (eg, PFS, time to PSA progression) will be performed using standard survival analysis methods including Kaplan-Meier product-limit survival curve estimates. Results will be summarized by 25th, 50th (median) and 75th percentiles with associated 2-sided 95% CIs, as well as percent of censored observations by treatment arm.

For categorical variables (eg, PSA response rate, objective response in subjects with measurable disease), the number and percent per category will be summarized by treatment.

No between-group comparisons will be performed.

11.5. Extension Phase and Follow-up Phase Data

The data from the extension phase and follow-up phase will be analyzed descriptively. Frequency overviews will be provided to tabulate the number of subjects with the corresponding reason for entering the extension phase per treatment arm (eg, study treatment continued unchanged, study treatment continued with a change in corticosteroid dose) or for entering the

follow-up phase. For those subjects entering the extension phase, the duration of study treatment will be summarized descriptively. Safety analysis will be based on reporting of (S)AEs started during the extension phase for all subjects and, if numbers allow, per dose of corticosteroids. Overall survival, time to next therapy for prostate cancer and time to initiation of chemotherapy will be analyzed using standard survival analysis methods including Kaplan-Meier product-limit survival curve estimates. Results will be summarized by 25th, 50th (median) and 75th percentiles with associated 2-sided 95% CIs, as well as percent of censored for all subjects in the follow-up phase. If numbers allow, overall survival will be analyzed separately for those subjects who continued treatment in the extension phase. The next anticancer therapy(ies) and the corresponding best response will be summarized descriptively.

11.6. Biomarker Analyses

Analyses of biomarker data will be done externally. Biomarker data will be reported separately from the clinical study report.

11.7. Medical Resource Utilization Analyses

For the MRU and health economics data, descriptive statistics for changes from baseline to each assessment will be presented by treatment arm.

11.8. Scientific Advisory Committee

The primary purpose of the SAC is to provide consulting oversight during the course of the study to ensure the scientific validity of the clinical study, to identify any scientifically relevant trends, and to provide recommendations to the sponsor. The SAC serves as an expert advisory group and is responsible for determining its operational procedures and acting in accordance with its approved SAC charter. The SAC will review data as specified in this charter. Throughout its tenure, the SAC will undertake these data reviews while maintaining the scientific integrity of the study. Any final strategic decision will belong to the sponsor.

12. ADVERSE EVENT REPORTING

Timely, accurate, and complete reporting and analysis of safety information from clinical studies are crucial for the protection of subjects, investigators, and the sponsor, and are mandated by regulatory agencies worldwide. The sponsor has established Standard Operating Procedures in conformity with regulatory requirements worldwide to ensure appropriate reporting of safety information; all clinical studies conducted by the sponsor or its affiliates will be conducted in accordance with those procedures.

12.1. Definitions

12.1.1. Adverse Event Definitions and Classifications

Adverse Event

An AE is any untoward medical occurrence in a clinical study subject administered a medicinal (investigational or non-investigational) product. An AE does not necessarily have a causal relationship with the treatment. An AE can therefore be any unfavorable and unintended sign

(including an abnormal finding), symptom, or disease temporally associated with the use of a medicinal (investigational or non-investigational) product, whether or not related to that medicinal (investigational or non-investigational) product. (Definition per International Conference on Harmonisation [ICH])

This includes any occurrence that is new in onset or aggravated in severity or frequency from the baseline condition, or abnormal results of diagnostic procedures, including laboratory test abnormalities designated as clinically significant in the opinion of the investigator. All incidences of hypokalemia (NCI CTCAE grade ≥1) and of hypertension (NCI CTCAE grade ≥2) will be reported as AEs.

Note: The sponsor will collect AEs starting from the signing of the ICF (refer to Section 12.3.1, All Adverse Events, for time of last AE recording).

Serious Adverse Event

An SAE based on ICH is any untoward medical occurrence that at any dose:

- Results in death
- Is life-threatening
  (The subject was at risk of death at the time of the event. It does not refer to an event that hypothetically might have caused death if it were more severe.)
- Requires inpatient hospitalization or prolongation of existing hospitalization
- Results in persistent or significant disability/incapacity
- Is a congenital anomaly/birth defect
- Is a suspected transmission of any infectious agent via a medicinal product
- Is Medically Important*

*Medical and scientific judgment should be exercised in deciding whether expedited reporting is also appropriate in other situations, such as important medical events that may not be immediately life threatening or result in death or hospitalization but may jeopardize the subject or may require intervention to prevent one of the other outcomes listed in the definition above. These should usually be considered serious.

Unlisted (Unexpected) Adverse Event/Reference Safety Information

An AE is considered unlisted if the nature or severity is not consistent with the applicable product reference safety information. For an investigational product, the expectedness of an AE will be determined by whether or not it is listed in the Investigator's Brochure. For a non-sponsor investigational medicinal product (eg, a comparator product) with a marketing authorization, the expectedness of an AE will be determined by whether or not it is listed in the package insert/SmPC.

Adverse Event Associated With the Use of the Drug

An AE is considered associated with the use of the drug if the attribution is possible, probable, or very likely by the definitions listed in Section 12.1.2.

12.1.2. Attribution Definitions

Not Related

An AE that is not related to the use of the drug.

Doubtful

An AE for which an alternative explanation is more likely, eg, concomitant drug(s), concomitant disease(s), or the relationship in time suggests that a causal relationship is unlikely.

Possible

An AE that might be due to the use of the drug. An alternative explanation, eg, concomitant drug(s), concomitant disease(s), is inconclusive. The relationship in time is reasonable; therefore, the causal relationship cannot be excluded.

Probable

An AE that might be due to the use of the drug. The relationship in time is suggestive (eg, confirmed by dechallenge). An alternative explanation is less likely, eg, concomitant drug(s), concomitant disease(s).

Very Likely

An AE that is listed as a possible adverse reaction and cannot be reasonably explained by an alternative explanation, eg, concomitant drug(s), concomitant disease(s). The relationship in time is very suggestive (eg, it is confirmed by dechallenge and rechallenge).

12.1.3. Severity Criteria

Adverse event severity is a clinical determination of the intensity of an AE and SAEs. The severity assessment for an AE/SAE should be completed using the NCI CTCAE, v4.0, (Attachment 7).

Any AE/SAE not listed in the CTCAE version 4.0 will be graded using the following general categorical descriptors:

Grade 1, Mild: Awareness of symptoms that are easily tolerated, causing minimal discomfort and not interfering with everyday activities.

Grade 2, Moderate: Sufficient discomfort is present to cause interference with normal activity.

Grade 3, Severe: Extreme distress, causing significant impairment of functioning or incapacitation. Prevents normal everyday activities.

Grade 4, Life-threatening: Urgent intervention indicated.

Grade 5, Death: Death.

The investigator should use clinical judgment in assessing the severity of events not directly experienced by the subject (eg, laboratory abnormalities).

12.2. Special Reporting Situations

Safety events of interest on a sponsor medicinal product that may require expedited reporting and/or safety evaluation include, but are not limited to:

- Overdose of a sponsor medicinal product
- Suspected abuse/misuse of a sponsor medicinal product
- Inadvertent or accidental exposure to a sponsor medicinal product
- Medication error involving a sponsor product (with or without subject/patient exposure to the sponsor medicinal product, eg, name confusion)

Special reporting situations should be recorded in the eCRF. Any special reporting situation that meets the criteria of an SAE should be recorded on the Serious Adverse Event page of the eCRF.

12.3. Procedures

12.3.1. All Adverse Events

All AEs and special reporting situations, whether serious or non-serious, will be reported from the time a signed and dated ICF is obtained until completion of the subject's last study-related procedure (which may include contact for follow-up of safety). For this study, the last study-related procedure will be the EOMS or EOE visit 4 weeks after the cessation of study medication. SAEs, including those spontaneously reported to the investigator within 30 days after the last dose of study drug, must be reported using the Serious Adverse Event Form. The sponsor will evaluate any safety information that is spontaneously reported by an investigator beyond the time frame specified in the protocol.

All AEs, regardless of seriousness, severity, or presumed relationship to study therapy, must be recorded using medical terminology in the source document and the eCRF. Whenever possible, diagnoses should be given when signs and symptoms are due to a common etiology (eg, cough, runny nose, sneezing, sore throat, and head congestion should be reported as "upper respiratory infection"). Investigators must record in the eCRF their opinion concerning the relationship of the AE to study therapy. All measures required for (S)AE management must be recorded in the source document and reported according to sponsor instructions.

The sponsor assumes responsibility for appropriate reporting of AEs to the regulatory authorities. The sponsor will also report to the investigator all SAEs that are unlisted (unexpected) and associated with the use of the drug. The investigator (or sponsor where required) must report these events to the appropriate IEC/IRB that approved the protocol unless otherwise required and documented by the IEC/IRB.

Subjects (or their designees, if appropriate) must be provided with a "study card" indicating the following:

- Subject's name
- Subject number
- Subject's date of birth
- Study site number
- Investigator's name and 24-hour contact information
- Local sponsor's name and 24-hour safety contact information
- Statement that the subject is participating in a clinical trial.

12.3.2. Serious Adverse Events

All SAEs occurring during clinical studies must be reported to the appropriate sponsor contact person by investigational staff within 24 hours of their knowledge of the event.

Information regarding SAEs will be transmitted to the sponsor using the Serious Adverse Event Form, which must be completed and signed by a member of the investigational staff, and transmitted to the sponsor within 24 hours. The initial and follow-up reports of an SAE should be made by facsimile (fax).

All SAEs that have not resolved by the end of the study, or that have not resolved upon discontinuation of the subject's participation in the study, must be followed until any of the following occurs:

- The event resolves
- The event stabilizes
- The event returns to baseline, if a baseline value/status is available
- The event can be attributed to agents other than the study drug or to factors unrelated to study conduct
- It becomes unlikely that any additional information can be obtained (subject or health care practitioner refusal to provide additional information, lost to follow-up after demonstration of due diligence with follow-up efforts)

Suspected transmission of an infectious agent by a medicinal product will be reported as an SAE. Any event requiring hospitalization (or prolongation of hospitalization) that occurs during the course of a subject's participation in a clinical study must be reported as an SAE, except hospitalizations for the following:

- Social reasons in the absence of an AE
- Surgery or procedure planned before entry into the study (must be documented in the eCRF)

Disease progression should not be recorded as an AE or SAE term; instead, signs and symptoms of clinical sequelae resulting from disease progression/lack of efficacy will be reported if they fulfill the SAE definition (see Section 12.1.1, Adverse Event Definitions and Classifications).

12.3.3. Pregnancy

Because the effect of the study drug on human sperm is unknown, pregnancies in partners of male subjects included in the study will be reported by the investigational staff within 24 hours of their knowledge of the event using the appropriate pregnancy notification form.

Follow-up information regarding the outcome of the pregnancy and any postnatal sequelae in the infant will be required.

12.4. Contacting Sponsor Regarding Safety

The names (and corresponding telephone numbers) of the individuals who should be contacted regarding safety issues or questions regarding the study are listed on the Contact Information page(s), which will be provided as a separate document.

13. PRODUCT QUALITY COMPLAINT HANDLING

A product quality complaint (PQC) is defined as any suspicion of a product defect related to manufacturing, labeling, or packaging, ie, any dissatisfaction relative to the identity, quality, durability, or reliability of a product, including its labeling or package integrity. PQCs may have an impact on the safety and efficacy of the product. Timely, accurate, and complete reporting and analysis of PQC information from clinical studies are crucial for the protection of subjects, investigators, and the sponsor, and are mandated by regulatory agencies worldwide. The sponsor has established procedures in conformity with regulatory requirements worldwide to ensure appropriate reporting of PQC information; all clinical studies conducted by the sponsor or its affiliates will be conducted in accordance with those procedures.

13.1. Procedures

All initial PQCs must be reported to the sponsor by the investigational staff within 24 hours after being made aware of the event.

If the defect is combined with an SAE, the investigational staff must report the PQC to the sponsor according to the SAE reporting timelines (refer to Section 12.3.2, Serious Adverse Events). A sample of the suspected product should be maintained for further investigation if requested by the sponsor.

13.2. Contacting Sponsor Regarding Product Quality

The names (and corresponding telephone numbers) of the individuals who should be contacted regarding product quality issues are listed on the Contact Information page(s), which will be provided as a separate document.

14. STUDY DRUG INFORMATION

14.1. Physical Description of Study Drugs

The abiraterone acetate 250-mg tablets supplied for this study are oval, white to off-white and contain abiraterone acetate and compendial (USP/NF/EP) grade lactose monohydrate, microcrystalline cellulose, croscarmellose sodium, povidone, sodium lauryl sulfate, magnesium stearate, colloidal silicon dioxide, and purified water, in descending order of concentration (the water is removed during tabletting).

Prednisone will be provided as 2.5-mg or 5-mg tablets.

Dexamethasone will be provided as 0.5-mg tablets.

14.2. Packaging

Study medication is provided to each site in bulk form. The study site pharmacist will dispense study medication to each subject in accordance with this protocol under the guidelines of the site's dispensation standard operating procedure.

14.3. Labeling

Study drug labels will contain information to meet the applicable regulatory requirements.

14.4. Preparation, Handling, and Storage

Study drugs must be stored in a secure area and administered only to study subjects in accordance with conditions specified in this protocol. Abiraterone acetate, dexamethasone and prednisone should be stored at monitored room temperature, as instructed by the manufacturer, in the original container. Subjects should be instructed to keep medications out of reach and sight of children. Additional information is provided in the abiraterone acetate Investigator's Brochure[31].

Women who are pregnant or may be pregnant should not handle abiraterone acetate without protection, eg, gloves. Refer to the site investigational product manual for additional guidance on study drug preparation and handling.

14.5. Drug Accountability

The investigator is responsible for ensuring that all study drug received at the site is inventoried and accounted for throughout the study. The dispensing of study drug to the subject, and the return of study drug from the subject (if applicable), must be documented on the drug accountability form. Subjects must be instructed to return all original containers, whether empty or containing study drug. All study drug will be stored and disposed of according to the sponsor's instructions. Site staff must not combine contents of the study drug containers. These requirements also apply to prednisone and dexamethasone tablets supplied by the Sponsor.

Study drug must be handled in strict accordance with the protocol and the container label, and must be stored at the study site in a limited-access area or in a locked cabinet under appropriate

environmental conditions. Unused study drug, and study drug returned by the subject, must be available for verification by the sponsor's site monitor during on-site monitoring visits. The return to the sponsor of unused study drug, or used returned study drug for destruction, will be documented on the drug return form. When the site is an authorized destruction unit and study drug supplies are destroyed on site, this must also be documented on the drug return form.

Study drug should be dispensed under the supervision of the investigator or a qualified member of the investigational staff, or by a hospital/clinic pharmacist. Study drug will be supplied only to subjects participating in the study. Returned study drug must not be dispensed again, even to the same subject. Whenever a subject brings his study drug to the site for pill count, this is not seen as a return of supplies. Study drug may not be relabeled or reassigned for use by other subjects. The investigator agrees neither to dispense the study drug from, nor store it at, any site other than the study sites agreed upon with the sponsor.

15. STUDY-SPECIFIC MATERIALS

The investigator will be provided with the following supplies:

- Investigator's brochure for abiraterone acetate
- SmPC for prednisone and dexamethasone
- Site investigational product manual
- Laboratory manual
- ECOG Performance Status, BP-SF, EQ-5D-5L and FACT-P questionnaires and user instructions
- IWRS Manual
- Electronic data capture (eDC) Manual
- Manual for DXA scans
- RECIST guidelines, (modified RECIST, Version 1.1; see Attachment 1)
- NCI CTCAE Version 4.0 (see Attachment 7)

16. ETHICAL ASPECTS

16.1. Study-Specific Design Considerations

The optimal management of chemotherapy-naïve, asymptomatic patients with prostate cancer refractory to medical or surgical castration who are showing tumor progression is uncertain. There are no approved second-line hormonal therapies for this population, and cytotoxic chemotherapy (docetaxel or mitoxantrone) is ordinarily reserved for patients with symptomatic or rapidly progressive cancer[10]. The American Society of Clinical Oncology/Cancer Care of Ontario and European Association of Urology (EAU guidelines for the cytotoxic treatment of CRPC[8,29]) emphasize the need to individualize the timing of nonhormonal therapy for prostate cancer and consider routine docetaxel questionable in men who have metastatic disease but lack symptoms. Similarly, the current European Society for Medical Oncology (ESMO) guidelines[21]

for metastatic prostate cancer recommend that patients with castrate-resistant disease should receive second and possibly third line hormonal therapies, while chemotherapy with docetaxel given every 3 weeks should be considered for patients with CRPC who are symptomatic. Thus the non-cytotoxic treatment of patients with mCRPC who are asymptomatic remains a significant unmet medical need.

Recently, the interim analysis results for study COU-AA-302[52] became available. The results of this interim analysis showed that abiraterone acetate plus prednisone produced a statistically significant improvement in rPFS and a strong trend for increased overall survival. Abiraterone acetate resulted in clinically and statistically significant effects on all secondary endpoints. The results confirmed the acceptable tolerability/safety profile of abiraterone acetate.

In clinical studies in subjects with mCRPC, the most common AEs related to abiraterone acetate monotherapy include hypertension, fluid retention/edema, and hypokalemia due to mineralocorticoid excess caused by compensatory ACTH drive. In this study, concurrent administration of prednisone or dexamethasone is expected to mitigate these effects through cortisol supplementation and abrogation of the ACTH drive.

Diuretic treatments or other changes, including an increased glucocorticoid dose as a “stress dose” during the study, have the potential to confound the safety analysis. Therefore, during the main study treatment period, subjects who require diuretic treatment or a change in glucocorticoid dose, or who experience grade 3 or grade 4 hypokalemia, will be discontinued from the main study. However, in the absence of disease progression, these patients could still benefit from study treatment. For this reason, an extension phase has been included in the study to enable continuation of study treatment if, in the investigator's opinion, the subject would benefit from this.

The use of osteoprotective agents such as bisphosphonates (eg, zoledronic acid) or denosumab, where they have received marketing authorization, is possible during this study, in accordance with local clinical practice and the current product label. Indeed, within the EU, bisphosphonates are indicated for prevention of skeletal-related events (eg, pathological fractures and spinal cord compressions) in patients with advanced malignancies involving bone or for the treatment of osteoporosis associated with long-term systemic glucocorticoid therapy in men at risk of fractures. Denosumab is approved in the EU for the treatment of bone loss associated with hormone ablation in men with prostate cancer at risk of fractures.

Potential subjects will be fully informed of the risks and requirements of the study and, during the study, subjects will be given any new information that may affect their decision to continue participation. They will be told that their consent to participate in the study is voluntary and may be withdrawn at any time with no reason given and without penalty or loss of benefits to which they would otherwise be entitled. Only subjects who are fully able to understand the risks, benefits, and potential AEs of the study, and provide their consent voluntarily will be enrolled.

The total blood volume to be collected is considered to be within the normal range for this subject population.

16.2. Regulatory Ethics Compliance

16.2.1. Investigator Responsibilities

The investigator is responsible for ensuring that the clinical study is performed in accordance with the protocol, current ICH guidelines on Good Clinical Practice (GCP), and applicable regulatory and country-specific requirements.

Good Clinical Practice is an international ethical and scientific quality standard for designing, conducting, recording, and reporting studies that involve the participation of human subjects. Compliance with this standard provides public assurance that the rights, safety, and well-being of study subjects are protected, consistent with the principles that originated in the Declaration of Helsinki, and that the clinical study data are credible.

16.2.2. Independent Ethics Committee or Institutional Review Board

Before the start of the study, the investigator (or sponsor where required) will provide the IEC/IRB with current and complete copies of the following documents:

- Final protocol and, if applicable, amendments
- Sponsor-approved ICF (and any other written materials to be provided to the subjects)
- IB (or equivalent information) and amendments/addenda
- Sponsor-approved subject recruiting materials
- Information on compensation for study-related injuries or payment to subjects for participation in the study, if applicable
- Investigator's curriculum vitae or equivalent information (unless not required, as documented by the IEC/IRB)
- Information regarding funding, name of the sponsor, institutional affiliations, other potential conflicts of interest, and incentives for subjects
- Any other documents that the IEC/IRB requests to fulfill its obligation

This study will be undertaken only after the IEC/IRB has given full approval of the final protocol, amendments (if any), the ICF, applicable recruiting materials, and subject compensation programs, and the sponsor has received a copy of this approval. This approval letter must be dated and must clearly identify the IEC/IRB and the documents being approved.

Approval for the collection of optional blood samples for biomarker research and for the corresponding ICF must also be obtained from the IEC/IRB. Approval for the protocol can be obtained independent of this optional research component.

During the study the investigator (or sponsor where required) will send the following documents and updates to the IEC/IRB for their review and approval, where appropriate:

- Protocol amendments
- Revision(s) to ICF and any other written materials to be provided to subjects

- If applicable, new or revised subject recruiting materials approved by the sponsor
- Revisions to compensation for study-related injuries or payment to subjects for participation in the study, if applicable
- New edition(s) of the IB and amendments/addenda
- Summaries of the status of the study at intervals stipulated in guidelines of the IEC/IRB (at least annually)
- Reports of AEs that are serious, unlisted/unexpected, and associated with the investigational drug
- New information that may adversely affect the safety of the subjects or the conduct of the study
- Deviations from or changes to the protocol to eliminate immediate hazards to the subjects
- Report of deaths of subjects under the investigator's care
- Notification if a new investigator is responsible for the study at the site
- Development Safety Update Report and Line Listings, where applicable
- Any other requirements of the IEC/IRB

For all protocol amendments (excluding the ones that are purely administrative, with no consequences for subjects, data or trial conduct), the amendment and applicable ICF revisions must be submitted promptly to the IEC/IRB for review and approval before implementation of the change(s).

At least once a year, the IEC/IRB will be asked to review and reapprove this clinical study. The reapproval should be documented in writing (excluding the ones that are purely administrative, with no consequences for subjects, data, or study conduct).

At the end of the study, the investigator (or sponsor where required) will notify the IEC/IRB about the study completion.

16.2.3. Informed Consent

Each subject must give written consent according to local requirements after the nature of the study has been fully explained. The consent form must be signed before performance of any study-related activity. The consent form that is used must be approved by both the sponsor and by the reviewing IEC/IRB and be in a language that the subject can read and understand. The informed consent should be in accordance with principles that originated in the Declaration of Helsinki, current ICH and GCP guidelines, applicable regulatory requirements, and sponsor policy.

Before enrollment in the study, the investigator or an authorized member of the investigational staff must explain to potential subjects the aims, methods, reasonably anticipated benefits, and potential hazards of the study, and any discomfort participation in the study may entail. Subjects will be informed that their participation is voluntary and that they may withdraw consent to participate at any time. They will be informed that choosing not to participate will not affect the

care the subject will receive for the treatment of his disease. Subjects will be told that alternative treatments are available if they refuse to take part and that such refusal will not prejudice future treatment. Finally, they will be told that the investigator will maintain a subject identification register for the purposes of long-term follow-up if needed and that their records may be accessed by health authorities and authorized sponsor staff without violating the confidentiality of the subject, to the extent permitted by the applicable law(s) or regulations. By signing the ICF, the subject is authorizing such access, and agrees to allow his study physician to recontact the subject for the purpose of obtaining consent for additional safety evaluations, if needed, and subsequent disease-related treatments, or to obtain information about his survival status.

The subject will be given sufficient time to read the ICF and the opportunity to ask questions. After this explanation and before entry into the study, consent should be appropriately recorded by means of the subject's personally dated signature. After having obtained the consent, a copy of the ICF must be given to the subject.

If the subject is unable to read or write, an impartial witness should be present for the entire informed consent process (which includes reading and explaining all written information) and should personally date and sign the ICF after the oral consent of the subject is obtained.

16.2.4. Privacy of Personal Data

The collection and processing of personal data from subjects enrolled in this study will be limited to those data that are necessary to fulfill the objectives of the study.

These data must be collected and processed with adequate precautions to ensure confidentiality and compliance with applicable data privacy protection laws and regulations. Appropriate technical and organizational measures to protect the personal data against unauthorized disclosures or access, accidental or unlawful destruction, or accidental loss or alteration must be put in place. Sponsor personnel whose responsibilities require access to personal data agree to keep the identity of study subjects confidential.

The informed consent obtained from the subject includes explicit consent for the processing of personal data and for the investigator to allow direct access to his original medical records for study-related monitoring, audit, IEC/IRB review, and regulatory inspection. This consent also addresses the transfer of the data to other entities and to other countries.

The subject has the right to request through the investigator access to his personal data and the right to request rectification of any data that are not correct or complete. Reasonable steps will be taken to respond to such a request, taking into consideration the nature of the request, the conditions of the study, and the applicable laws and regulations.

Exploratory biomarker research is not conducted under standards appropriate for the return of data to subjects. In addition, the sponsor cannot make decisions as to the significance of any findings resulting from exploratory research. Therefore, exploratory research data will not be returned to subjects or investigators, unless required by law. Privacy and confidentiality of data

generated in the future on stored samples will be protected by the same standards applicable to all other clinical data.

16.2.5. Long-Term Retention of Samples for Biomarker Research

Biomarker research will only be used to understand abiraterone acetate with different steroid regimens, understand mCRPC, understand differential drug responders, and/or to develop tests/assays related to abiraterone acetate and/or mCRPC. Blood and plasma samples for biomarker research will be stored only until biomarker analysis has been completed.

Samples will be coded throughout the sample storage and analysis process and will not be labeled with personal identifiers. Subjects may withdraw their consent for their optional biomarker samples to be stored for research purposes (refer to Section 10.3).

16.2.6. Country Selection

This study will only be conducted in those countries where the intent is to launch or otherwise help ensure access to the developed product, unless explicitly addressed as a specific ethical consideration in Section 16.1, Study-Specific Design Considerations.

17. ADMINISTRATIVE REQUIREMENTS

17.1. Protocol Amendments

Neither the investigator nor the sponsor will modify this protocol without a formal amendment by the sponsor. All protocol amendments must be issued by the sponsor, and signed and dated by the investigator. Protocol amendments must not be implemented without prior IEC/IRB approval, or when the relevant competent authority has raised any grounds for non-acceptance, except when necessary to eliminate immediate hazards to the subjects, in which case the amendment must be promptly submitted to the IEC/IRB and relevant competent authority. Documentation of amendment approval by the investigator and IEC/IRB must be provided to the sponsor or its designee. When the change(s) involves only logistic or administrative aspects of the study, the IRB (and IEC where required) only needs to be notified.

During the course of the study, in situations where a departure from the protocol is unavoidable, the investigator or other physician in attendance will contact the appropriate sponsor representative (see Contact Information page(s) provided separately). Except in emergency situations, this contact should be made before implementing any departure from the protocol. In all cases, contact with the sponsor must be made as soon as possible to discuss the situation and agree on an appropriate course of action. The data recorded in the eCRF and source documents will reflect any departure from the protocol, and the source documents will describe this departure and the circumstances requiring it.

17.2. Regulatory Documentation

17.2.1. Regulatory Approval/Notification

This protocol and any amendment(s) must be submitted to the appropriate regulatory authorities in each respective country, if applicable. A study may not be initiated until all local regulatory requirements are met.

17.2.2. Required Prestudy Documentation

The following documents must be provided to the sponsor before shipment of study drug to the investigational site:

- Protocol and amendment(s), if any, signed and dated by the principal investigator
- A copy of the dated and signed, written IEC/IRB approval of the protocol, amendments, ICF, any recruiting materials, and if applicable, subject compensation programs. This approval must clearly identify the specific protocol by title and number and must be signed by the chairman or authorized designee.
- Name and address of the IEC/IRB, including a current list of the IEC/IRB members and their function, with a statement that it is organized and operates according to GCP and the applicable laws and regulations. If accompanied by a letter of explanation, or equivalent, from the IEC/IRB, a general statement may be substituted for this list. If an investigator or a member of the investigational staff is a member of the IEC/IRB, documentation must be obtained to state that this person did not participate in the deliberations or in the vote/opinion of the study.
- Regulatory authority approval or notification, if applicable
- Signed and dated statement of investigator (eg, Form FDA 1572), if applicable
- Documentation of investigator qualifications (eg, curriculum vitae)
- Completed investigator financial disclosure form from the principal investigator, where required
- Signed and dated clinical trial agreement, which includes the financial agreement
- Any other documentation required by local regulations

The following documents must be provided to the sponsor before enrollment of the first subject:

- Completed investigator financial disclosure forms from all clinical subinvestigators
- Documentation of subinvestigator qualifications (eg, curriculum vitae)
- Name and address of any local laboratory conducting tests for the study, and a dated copy of current laboratory normal ranges for these tests, if applicable
- Local laboratory documentation demonstrating competence and test reliability (eg, accreditation/license), if applicable

17.3. Subject Identification, Enrollment, and Screening Logs

The investigator agrees to complete a subject identification and enrollment log to permit easy identification of each subject during and after the study. This document will be reviewed by the sponsor study site contact for completeness.

The subject identification and enrollment log will be treated as confidential and will be filed by the investigator in the study file. To ensure subject confidentiality, no copy will be made. All reports and communications relating to the study will identify subjects by subject ID and date of birth. In cases where the subject is not randomized into the study, the date seen and date of birth will be used.

The investigator must also complete a subject screening log, which reports on all subjects who were seen to determine eligibility for inclusion in the study.

17.4. Source Documentation

At a minimum, source documentation must be available for the following to confirm data collected in the eCRF: subject identification, eligibility, and study identification; study discussion and date of informed consent; dates of visits; results of safety and efficacy parameters as required by the protocol; record of all AEs and follow-up of AEs; concomitant medication; drug receipt/dispensing/return records; study drug administration information; and date of study completion and reason for early discontinuation of study drug or withdrawal from the study, if applicable.

In addition, the author of an entry in the source documents should be identifiable.

At a minimum, the type and level of detail of source data available for a study subject should be consistent with that commonly recorded at the site as a basis for standard medical care. Specific details required as source data for the study will be reviewed with the investigator before the study and will be described in the monitoring guidelines (or other equivalent document).

The following data will be recorded directly into the eCRF and will be considered source data:

- Race
- Tobacco and alcohol use
- Health Economics data

The recording of PRO assessments (EQ-5D-5L, BPI-SF and FACT-P), as reported by the subject, will be considered source data. If the subject is unable to read or write, a designated member of the staff should be present for reading and explaining all written information and/or completing the questionnaires.

17.5. Case Report Form Completion

Case report forms are provided for each subject in electronic format.

Electronic data capture (eDC) will be used for this study. The study data will be transcribed by study personnel from the source documents onto an eCRF, and transmitted in a secure manner to the sponsor within the timeframe agreed upon between the sponsor and the site. The electronic file will be considered to be the eCRF.

All data relating to the study must be recorded in eCRFs prepared by the sponsor. Data must be entered into eCRFs in English. Designated site personnel must complete the eCRF as soon as possible after a subject visit, and the forms should be available for review at the next scheduled monitoring visit. The investigator must confirm that all data entries in the eCRFs are accurate and correct.

All subjective measurements (eg, pain scale information or other questionnaires) as reported by the subject will be completed by the same individual who recorded the initial baseline determinations whenever possible.

All eCRF entries, corrections, and alterations must be made by the investigator or other authorized study-site personnel. If necessary, queries will be generated in the eDC tool. The investigator or an authorized member of the investigational staff must adjust the eCRF (if applicable) and complete the query.

If corrections to an eCRF are needed after the initial entry into the eCRF, this can be done in 3 different ways:

- Site personnel can make corrections in the eDC tool at their own initiative or as a response to an auto query (generated by the eDC tool).
- A site manager can generate a query for resolution by the investigational staff.
- A clinical data manager can generate a query for resolution by the investigational staff.

17.6. Data Quality Assurance/Quality Control

Steps to be taken to ensure the accuracy and reliability of data include the selection of qualified investigators and appropriate study sites, review of protocol procedures with the investigator and associated personnel before the study, and periodic monitoring visits by the sponsor. Written instructions will be provided for collection, preparation, and shipment of samples.

Guidelines for eCRF completion will be provided and reviewed with study personnel before the start of the study.

The sponsor will review eCRFs for accuracy and completeness during on-site monitoring visits and after transmission to the sponsor; any discrepancies will be resolved with the investigator or designee, as appropriate. After upload of the data into the clinical study database they will be verified for accuracy and consistency with the data sources.

17.7. Record Retention

In compliance with the ICH/GCP guidelines, the investigator/institution will maintain all eCRFs and all source documents that support the data collected from each subject, as well as all study documents as specified in ICH/GCP Section 8, Essential Documents for the Conduct of a Clinical Trial, and all study documents as specified by the applicable regulatory requirement(s). The investigator/institution will take measures to prevent accidental or premature destruction of these documents.

Essential documents must be retained until at least 2 years after the last approval of a marketing application in an ICH region and until there are no pending or contemplated marketing applications in an ICH region or until at least 2 years have elapsed since the formal discontinuation of clinical development of the investigational product. These documents will be retained for a longer period if required by the applicable regulatory requirements or by an agreement with the sponsor. It is the responsibility of the sponsor to inform the investigator/institution as to when these documents no longer need to be retained.

If the responsible investigator retires, relocates, or for other reasons withdraws from the responsibility of keeping the study records, custody must be transferred to a person who will accept the responsibility. The sponsor must be notified in writing of the name and address of the new custodian. Under no circumstance shall the investigator relocate or dispose of any study documents before having obtained written approval from the sponsor.

If it becomes necessary for the sponsor or the appropriate regulatory authority to review any documentation relating to this study, the investigator must permit access to such reports.

17.8. Monitoring

The sponsor will perform on-site monitoring visits as frequently as necessary. The monitor will record dates of the visits in a study site visit log that will be kept at the site. The first post-initiation visit will be made as soon as possible after enrollment has begun. At these visits, the monitor will compare the data entered into the eCRFs with the hospital or clinic records (source documents). The nature and location of all source documents will be identified to ensure that all sources of original data required to complete the eCRF are known to the sponsor and investigational staff and are accessible for verification by the sponsor site contact. If electronic records are maintained at the investigational site, the method of verification must be discussed with the investigational staff.

Direct access to source documentation (medical records) must be allowed for the purpose of verifying that the data recorded in the eCRF are consistent with the original source data. Findings from this review of eCRFs and source documents will be discussed with the investigational staff. The sponsor expects that, during monitoring visits, the relevant investigational staff will be available, the source documentation will be accessible, and a suitable environment will be provided for review of study-related documents. The monitor will meet with the investigator on a regular basis during the study to provide feedback on the study conduct.

17.9. Study Completion/Termination

17.9.1. Study Completion

The study is considered completed with the last study assessment for the last subject participating in the study. The final data from the investigational site will be sent to the sponsor (or designee) after completion of the final subject assessment at that site, in the time frame specified in the Clinical Trial Agreement.

17.9.2. Study Termination

The sponsor reserves the right to close the investigational site or terminate the study at any time for any reason at the sole discretion of the sponsor. Investigational sites will be closed upon study completion. An investigational site is considered closed when all required documents and study supplies have been collected and a site closure visit has been performed.

The investigator may initiate site closure at any time, provided there is reasonable cause and sufficient notice is given in advance of the intended termination.

Reasons for the early closure of an investigational site by the sponsor or investigator may include but are not limited to:

- Failure of the investigator to comply with the protocol, the requirements of the IEC/IRB or local health authorities, the sponsor's procedures, or GCP guidelines
- Inadequate recruitment of subjects by the investigator
- Discontinuation of further drug development

17.10. On-Site Audits/Inspections

Representatives of the sponsor's clinical quality assurance department may visit the site at any time during or after completion of the study to conduct an audit of the study in compliance with regulatory guidelines and company policy. These audits will require access to all study records, including source documents, for inspection and comparison with the eCRFs. Subject privacy must, however, be respected. The investigator and staff are responsible for being present and available for consultation during routinely scheduled site audit visits conducted by the sponsor or its designees.

Similar auditing procedures (inspections) may also be conducted by agents of any regulatory body, either as part of a national GCP compliance program or to review the results of this study in support of a regulatory submission. The investigator should immediately notify the sponsor if they have been contacted by a regulatory agency concerning an upcoming inspection.

17.11. Use of Information and Publication

All information, including but not limited to information regarding abiraterone acetate or the sponsor's operations (eg, patent application, formulas, manufacturing processes, basic scientific data, prior clinical data, formulation information) supplied by the sponsor to the investigator and not previously published, and any data, including pharmacogenomic or exploratory biomarker

research data, generated as a result of this study, are considered confidential and remain the sole property of the sponsor. The investigator agrees to maintain this information in confidence and use this information only to accomplish this study, and will not use it for other purposes without the sponsor's prior written consent.

The investigator understands that the information developed in the study will be used by the sponsor in connection with the continued development of abiraterone acetate, and thus may be disclosed as required to other clinical investigators or regulatory agencies. To permit the information derived from the clinical studies to be used, the investigator is obligated to provide the sponsor with all data obtained in the study.

The results of the study will be reported in a Clinical Study Report generated by the sponsor and will contain eCRF data from all study sites that participated in the study. Recruitment performance or specific expertise related to the nature and the key assessment parameters of the study will be used to determine a coordinating investigator. Results of biomarker data analyses performed after the Clinical Study Report has been issued will be reported in a separate report and will not require a revision of the Clinical Study Report. Study subject identifiers will not be used in publication of results. Any work created in connection with performance of the study and contained in the data that can benefit from copyright protection (except any publication by the investigator as provided for below) shall be the property of the sponsor as author and owner of copyright in such work.

Consistent with Good Publication Practices and International Committee of Medical Journal Editors guidelines, the sponsor shall have the right to publish such primary (multicenter) data and information without approval from the investigator. The investigator has the right to publish study site-specific data after the primary data are published. If an investigator wishes to publish information from the study, a copy of the manuscript must be provided to the sponsor for review at least 60 days before submission for publication or presentation. Expedited reviews will be arranged for abstracts, poster presentations, or other materials. If requested by the sponsor in writing, the investigator will withhold such publication for up to an additional 60 days to allow for filing of a patent application. In the event that issues arise regarding scientific integrity or regulatory compliance, the sponsor will review these issues with the investigator. The sponsor will not mandate modifications to scientific content and does not have the right to suppress information. For multicenter study designs and substudy approaches, secondary results generally should not be published before the primary endpoints of a study have been published. Similarly, investigators will recognize the integrity of a multicenter study by not submitting for publication data derived from the individual study site until the combined results from the completed study have been submitted for publication, within 12 months of the availability of the final data (tables, listings, graphs), or the sponsor confirms there will be no multicenter study publication. Authorship of publications resulting from this study will be based on the guidelines on authorship, such as those described in the Uniform Requirements for Manuscripts Submitted to Biomedical Journals, which state that the named authors must have made a significant contribution to the design of the study or analysis and interpretation of the data, provided critical review of the paper, and given final approval of the final version.

Registration of Clinical Studies and Disclosure of Results

The sponsor will register and/or disclose the existence of and the results of clinical studies as required by law.

REFERENCES

1. Ambs S, Prueitt R, Yi M, et al. Genomic profiling of MicroRNA and messenger RNA reveals deregulated microRNA expression in prostate cancer. Cancer Res 2008;68:15:6162-70.

2. Arlt W. The approach to the adult with newly diagnosed adrenal insufficiency. J Clin Endocrinol Metab 2009;94:1059-67.

3. Attard G, Cooper CS, de Bono JS. Steroid hormone receptors in prostate cancer: a hard habit to break? Cancer Cell. 2009;16(6):458-62.

4. Attard G, Reid AH, Auchus RJ, et al. Clinical and biochemical consequences of CYP17A1 inhibition with abiraterone given with and without exogenous glucocorticoids in castrate men with advanced prostate cancer. J Clin Endocrinol Metab 2012;97(2):507-16.

5. Attard G, Reid AHM, A'Hern R et al. Selective inhibitor of CYP17 with abiraterone acetate is highly active in the treatment of castration-resistant prostate cancer. J Clin Onc 2009; 27 (23): 3742-3748.

6. Attard G, Reid AHM, Yap T, et al. Phase I clinical trial of selective inhibitor CYP17 abiraterone acetate, confirms that castration-resistant prostate cancer commonly remains hormone driven. J Clin Onc 2008; 26 (28): 4563-4571.

7. Auchus RJ. The genetics, pathophysiology, and management of human deficiencies of P450c17. Endocrinol Metab Clin North Am 2001;30(1):101-19.

8. Basch EM, Somerfield MR, Beer TM, et al. American Society of Clinical Oncology Endorsement of the Cancer Care Ontario Practice Guidelines on Nonhormonal Therapy for Men With Metastatic Hormone_Refractory (Castration-Resistant) Prostate Cancer. J Clin Oncol 2007; 25:5313-8.

9. Bruchovsky N, Wilson JD. The conversion of testosterone to 5-alpha-androstan-17-beta-ol-3-one by rat prostate in vivo and in vitro. J Biol Chem 1968; 243:2012-21.

10. Calabro F, Sternberg CN. Current indications for chemotherapy in prostate cancer patients. Eur Urol 2007; 51:17-26

11. Carducci M, Nelson JB, Vogelzang NJ, Mulani P. Determination of Clinically Meaningful Change (CMC) for Functional Assessment of Cancer Therapy-Prostate (FACT-P). J Clin Oncol, 2005 ASCO Annual Meeting Proceedings. Abstract 8077;23:8077.

12. Chen CD, Welsbie DS, Tran C, et al. Molecular determinants of resistance to antiandrogen therapy. Nat Med 2004; 10:33-9.

13. Cleeland CS, Nakamura Y, Mendoza TR, et al. Dimensions of the impact of cancer pain in a four country sample: New information from multidimensional scaling. Pain 1996;67:267-273.

14. Crawford ED, Eisenberger MA, McLeod DG, Spaulding JT, Benson R, Dorr FA, et al. A controlled trial of leuprolide with and without flutamide in prostatic carcinoma. N Engl J Med 1989;321:419-24.

15. de Bono JS, Logothethis CJ, Molina A, et al. Abiraterone and increased survival with metastatic prostate cancer. N Engl J Med 2011; 364(21): 1995-2005.

16. Dexamethason 0.5mg JENAPHARM® [SmPC]. Brehna, Germany: Mibe GmbH Arzneimittel; 2010.

17. Efstathiou E. et al. Cytoreduction and androgen signaling modulation by abiraterone acetate plus leuprolide acetate versus leuprolide acetate in localized high-risk prostate cancer: preliminary results of a randomized preoperative study. J Clin Oncol 2012; 30 (suppl; abstr 4556)

18. Eisenberger MA, Blumenstein BA, Crawford ED, Miller G, et al. Bilateral orchiectomy with or without flutamide for metastatic prostate cancer. N Engl J Med 1998;339:1036-42.

19. Eisenhauer EA, Therasse P, Bogaerts et al., New response evaluation criteria in solid tumours: Revised RECIST guideline (version 1.1). Eur J Cancer 2009;45:228-247.

20. EQ-5D Web site. http://www.euroqol.org/eq-5d/what-is-eq-5d.html. Accessed August 7, 2012.

21. ESMO Clinical Recommendations. Annals of Oncology: 19:Supplement 2, May, 2008

22. Esper P, Mo F, Chodak G, Sinner M, Cella D, Pienta KJ. Measuring quality of life in men with prostate cancer using the functional assessment of cancer therapy-prostate instrument. Urology 1997;50:920-8.

23. Forshew T, Murtaza M, Parkinson C, et al. Noninvasive identification and monitoring of cancer mutations by targeted deep sequencing of plasma DNA. Sci Transl Med. 2012;4(136):136ra68.

24. Fossa SD, Slee PH, Brausi M, et al. Flutamide versus prednisone in patients with prostate cancer symptomatically progressing after androgen-ablative therapy: a Phase 3 study of the European organization for research and treatment of cancer genitourinary group. J Clin Oncol 2001;19:62-71.

25. Grasso CS, Wu YM, Robinson DR, et al. The mutational landscape of lethal castration-resistant prostate cancer. Nature 2012;487(7406):239-43.

26. Gregory CW, He B, Johnson RT, et al. A mechanism for androgen receptor-mediated prostate cancer recurrence after androgen deprivation therapy. Cancer Res 2001; 61:4315-4319.

27. Gregory CW, Johnson RT, Jr, Mohler JL, et al. Androgen receptor stabilization in recurrent prostate cancer is associated with hypersensitivity to low androgen. Cancer Res 2001; 61:2892-8.

28. He L, Hannon G. MicroRAs: small RNAs with a big role in gene regulation. Nat Rev Genet 2004;5:522-31.

29. Heidenreich A, Aus G, Abbou CC, et al. Guidelines on Prostate Cancer. European Association of Urology 2008.

30. Hellerstedt BA and Pienta KJ. The current state of hormonal therapy for prostate cancer. CA Cancer J Clin 2002; 52:154-79.

31. Investigator's Brochure: JNJ-212082 (abiraterone acetate) Edition 12. Janssen R&D (26 August 2015); Addendum 1 to Edition 12 (23 Nov 2015).

32. Jacobs SC. Spread of Prostatic Cancer to Bone. Urology 1983;21(4):337-44.

33. Kamangar F, Dores G.M, Anderson WF. Patterns of cancer incidence, mortality, and prevalence across five continents: defining priorities to reduce cancer disparities in different geographic regions of the world. J Clin Oncol 2006;24:2137-50.

34. Kumar MS, Lu J, Mercer KL, Golub TR, Jacks T. Impaired microRNA processing enhances cellular transformation and tumorigenesis. Nat Genet 2007;39:673–7.

35. Labrie F, Dupont A, Belanger A, et al. New approach in the treatment of prostate cancer: complete instead of partial withdrawal of androgens. Prostate 1983; 4:579-94.

36. Li B, Shi X, Xue L, Chen A, Valicenti R, deVere White R. Effect of down-regulation of microRNA 106b on p21-mediated cell cycle arrest in response to radiation in prostate cancer cells. ASCO Abstract 53 (2010 Genitourinary Cancers Symposium).

37. Li Z, Chen Y, Cao D, et al. Glucocorticoid up-regulates transforming growth factor-{beta} (TGF-{beta}) type II receptor and enhances TGF-{beta} signaling in human prostate cancer PC-3 cells. Endocrinology 2006; 147:5259-67.

38. Lim L, Lau N, Garret-Engele P et al. Microarray analysis shows that some microRNAs downregulate large numbers of target mRNAs. Nature 2005;433:769-73.

39. Mohler JL, Gregory CW, Ford OH. III, et al. The androgen axis in recurrent prostate cancer. Clin Cancer Res 2004; 10:440-8.

40. Montgomery RB, Mostaghel EA, Vessella R, et al. Maintenance of intratumoral androgens in metastatic prostate cancer: a mechanism for castration-resistant tumor growth. Cancer Res. 2008 68(11):4447-54.

41. Morioka M, Kobayashi T, Furukawa Y, Jo Y, et al. Prostatespecific antigen levels and prognosis in patients with hormone-refractory prostate cancer treated with low-dose dexamethasone. Urol Int 2002;68:10-5.

42. Nishimura K, Nonomura N, Satoh E, et al. Potential mechanisms for the effects of dexamethasone on growth of androgen-independent prostate cancer. JNCI 2001; 93:1739-46.

43. Olmos D, Brewer D, Clark J et al. Prognostic value of blood mRNA expression signatures in castration-resistant prostate cancer: a prospective, two-stage study. Lancet Oncology 2012; 13(11):1114-24.

44. Ozen M, Creighton C, Ozdemir M, Ittmann M. Widespread deregulation of microRNA expression in human prostate cancer. Oncogene. 2008;27:1788-93.

45. Peterson RE, Imperato-McGinley J, et al. Male pseudohermaphroditism due to steroid 5-alpha-reductase deficiency. Am J Med 1977; 62:170-91.

46. Porkka K, Pfeiffer M, Waltering K, Vessella R, Tammela T, Visakorpi T. MicroRNA expression profiling in Prostate cancer. Cancer Res 2007;67:6130-5.

47. PredniSONE 2.5 mg, 5 mg tablets [package insert] Columbus, Ohio, US: Roxane Laboratories Inc; 2012.

48. Prednison acis® 5 mg tablets [SmPC]. Grunwald, Germany: acis Arzneimittel GmbH; 2011.

49. Prednisone 2.5 mg, 5 mg tablets USP [package insert]. Eatontown, NJ, US: West-Ward Pharmaceutical Corp; 1997.

50. Questionnaires: cancer specific measures, FACT-P for patients with prostate cancer. FACIT Web site. http://www.facit.org/FACITOrg/Questionnaires. Accessed August 7, 2012.

51. Ryan CJ, Smith MR, Fong L, et al. Phase I clinical trial of the CYP17 inhibitor abiraterone acetate demonstrating clinical activity in patients with castration-resistant prostate cancer who received prior ketoconazole therapy. J Clin Oncol. 2010;28(9):1481-8.

52. Ryan J et al. Interim analysis results of COU-AA-302, a randomized, phase III study of abiraterone acetate in chemotherapy-naïve patients with metastatic castration-resistant prostate cancer. J Clin Oncol 2012; 30 (suppl; abstr LBA4518).

53. Saika T, Kusaka N, Tsushima T, et al. Treatment of androgen-independent prostate cancer with dexamethasone: a prospective study in stage D2 patients. Int J Urol 2001;8:290-4.

54. Saunders FJ. Some aspects of relation of structure of steroids to their prostate-stimulating effects. Natl Cancer Inst Monogr 1963; 12:139-159.

55. Scher HI, Halabi S, Tannock I, et al. Design and end points of clinical trials for patients with progressive prostate cancer and castrate levels of testosterone: recommendations of the Prostate Cancer Clinical Trials Working Group. J Clin Oncol 2008; 26:1148-59.

56. Schmitt B, Wilt TJ, Schellhammer PF, et al. Combined androgen blockade with nonsteroidal antiandrogens for advanced prostate cancer: a systematic review. Urology 2001;57:727-32.

57. Schwarzenbach H, Hoon DS, Pantel K. Cell-free nucleic acids as biomarkers in cancer patients. Nat Rev Cancer. 2011;11(6):426-37.

58. Serlin RC, Mendoza TR, Nakamura Y, Edwards KR, Cleeland CS. When is cancer pain mild moderate or severe? Grading pain severity by its interference with function. Pain 1995;61:277-84.

59. Smith MR, Saad F, Egerdie B, et al. Sarcopenia during androgen-deprivation therapy for prostate cancer. J Clin Oncol 2012; 30(26):3271.

60. Stigliano A, Gandini O, Cerquetti L, et al. Increased metastatic lymph node 64 and CYP17 expression are associated with high stage prostate cancer. J Endocrinol 2007; 194:55-61.

61. Storlie JA, Buckner JC, Wiseman GA, Burch PA, Hartmann LC, Richardson RL. Prostate specific antigen levels and clinical response to low dose dexamethasone for hormone-refractory metastatic prostate carcinoma. Cancer 1995;76:96-100.

62. Sun T, Yang M, Wang Q, et al. Castration-Resistant Prostate cancer-related microRNAs. ASCO Abstract 150 (2010 Genitourinary Cancers Symposium).

63. Szczyrba J, Löprich E, Wach S, et al. The microRNA profile of prostate carcinoma obtained by deep sequencing. Mol Cancer Res. 2010;8(4):529-38.

64. Tannock I, Gospodarowicz M, Meakin W, Panzarella T, Stewart L, Rider W. Treatment of metastatic prostatic cancer with low-dose prednisone: evaluation of pain and quality of life as pragmatic indices of response J Clin Oncol 1989;7:590-7.

65. Tannock IF, Osoba D, Stockler MR, et al. Chemotherapy with mitoxantrone plus prednisone or prednisone alone for symptomatic hormone resistant prostate cancer: a Canadian randomized trial with palliative endpoints. J Clin Oncol 1996; 14:1756-64.

66. Taplin ME and Ho SM. Clinical review 134: The endocrinology of prostate cancer. J Clin Endocrinol Metab 2001; 86:3467-77.

67. Taplin ME et al. Effect of neoadjuvant abiraterone acetate plus leuprolide acetate on PSA, pathological complete response, and near pathological complete response in localized high-risk prostate cancer: results of a randomized phase II study. J Clin Oncol 2012; 30 (suppl; abstr 4521).

68. Tong A, Fulgham P, Jay C, et al. MicroRNA profile analysis of human prostate cancers. Cancer Gene Therapy 2009: 16;206-16.

69. Venkitaraman R, Thomas K, Huddart RA, Horwich A, Dearnaley DP, Parker CC. Efficacy of low-dose dexamethasone in castration-refractory prostate cancer. BJU Int 2008;101(4):440-3.

70. Wang L, Tang H, Thayanithy V, et al. Gene networks and microRNAs implicated in aggressive prostate cancer. Cancer Res. 2009: 69:9490-7.

71. Wilson JD. Role of dihydrotestostrone in androgen action. Prostate Suppl 1996; 6:88-92.

72. Zytiga (abiraterone acetate) [SmPC]. Beerse, Belgium. Janssen-Cilag International NV; 2016.

Attachment 1: Summary of Response Evaluation Criteria in Solid Tumors (RECIST, Version 1.1)

The following information was extracted from Section 3, Section 4, and Appendix I of the New response evaluation criteria in solid tumours: Revised RECIST guideline (version 1.1) authored by Eisenhauer et al (2009). Refer to the European Journal of Cancer article (2009;45(2):228-247) for the complete publication.[19]

3. Measurability of tumor at baseline

3.1 Definitions

At baseline, tumor lesions/lymph nodes will be categorized measurable or non-measurable as follows:

3.1.1 Measurable

Tumor lesions: Must be accurately measured in at least one dimension (longest diameter in the plane of measurement is to be recorded) with a *minimum* size of:

- 10 mm by CT scan (CT scan slice thickness no greater than 5 mm)
- 10 mm caliper measurement by clinical exam (lesions which cannot be accurately measured with calipers should be recorded as non-measurable)
- 20 mm by chest X-ray
- *Malignant lymph nodes:* To be considered pathologically enlarged and measurable, a lymph node must be ≥15 mm in short axis when assessed by CT scan (CT scan slice thickness recommended to be no greater than 5 mm). At baseline and in follow-up, only the short axis will be measured and followed. See also 'Baseline documentation of target and non-target lesions' in section 4.2 of the RECIST guideline for information on lymph node measurement.

3.1.2 Non-measurable

All other lesions, including small lesions (longest diameter <10 mm or pathological lymph nodes with ≥10 to <15 mm short axis) as well as truly non-measurable lesions. Lesions considered truly non-measurable include: leptomeningeal disease, ascites, pleural or pericardial effusion, inflammatory breast disease, lymphangitic involvement of skin or lung, and abdominal masses/abdominal organomegaly identified by physical exam that is not measurable by reproducible imaging techniques.

3.2 Specifications by methods of measurements

3.2.1 Measurement of lesions

All measurements should be recorded in metric notation, using calipers if clinically assessed. All baseline evaluations should be performed as close as possible to the treatment start and never more than 4 weeks before the beginning of the treatment.

3.2.2 Method of assessment

The same method of assessment and the same technique should be used to characterize each identified and reported lesion at baseline and during follow-up. Imaging based evaluation should always be done rather than clinical examination.

4. Tumor response evaluation

4.1 Assessment of overall tumor burden and measurable disease

To assess objective response or future progression, it is necessary to estimate the *overall tumor burden at baseline* and use this as a comparator for subsequent measurements.

4.2 Baseline documentation of 'target' and 'non-target' lesions

When more than one measurable lesion is present at baseline all lesions up to a maximum of five lesions total (and a maximum of two lesions per organ) representative of all involved organs should be identified as *target lesions* and will be recorded and measured at baseline (this means in instances where patients have only one or two organ sites involved a *maximum* of two and four lesions respectively will be recorded). For evidence to support the selection of only five target lesions, see analyses on a large prospective database in the article by Bogaerts et al. (Reference #10 in Eisenhauer publication).

Target lesions should be selected on the basis of their size (lesions with the longest diameter), be representative of all involved organs, but in addition should be those that lend themselves to *reproducible repeated measurements*. It may be the case that, on occasion, the largest lesion does not lend itself to reproducible measurement in which circumstance the next largest lesion, which can be measured reproducibly, should be selected.

Lymph nodes merit special mention since they are normal anatomical structures, which may be visible by imaging even if not involved by tumor. As noted in Section 3, pathological nodes which are defined as measurable and may be identified as target lesions must meet the criterion of a short axis of ≥15 mm by CT scan. Only the short axis of these nodes will contribute to the baseline sum. The *short* axis of the node is the diameter normally used by radiologists to judge if a node is involved by solid tumor. Nodal size is normally reported as two dimensions in the plane in which the image is obtained (for CT scan this is almost always the axial plane; for MRI the plane of acquisition may be axial, sagittal or coronal). The smaller of these measures is the short axis. For example, an abdominal node which is reported as being 20 mm•30 mm has a short axis of 20 mm and qualifies as a malignant, measurable node. In this example, 20 mm should be recorded as the node measurement (See also the example in Fig. 4 in Appendix II of the Eisenhauer reference). All other pathological nodes (those with short axis ≥10 mm but <15 mm) should be considered non-target lesions. Nodes that have a short axis <10 mm are considered non-pathological and should not be recorded or followed.

A sum of the diameters (longest for non-nodal lesions, short axis for nodal lesions) for all target lesions will be calculated and reported as the *baseline sum diameters*. If lymph nodes are to be included in the sum, then as noted above, only the short axis is added into the sum. The baseline sum diameters will be used as reference to further characterize any objective tumor regression in the measurable dimension of the disease.

All other lesions (or sites of disease) including pathological lymph nodes should be identified as *non-target lesions* and should also be recorded at baseline. Measurements are not required and these lesions should be followed as 'present', 'absent', or in rare cases 'unequivocal progression' (more details to follow). In addition, it is possible to record multiple nontarget lesions involving the same organ as a single item on the case record form (e.g. 'multiple enlarged pelvic lymph nodes' or 'multiple liver metastases').

4.3 Response criteria

This section provides the definitions of the criteria used to determine objective tumor response for target lesions.

4.3.1 Evaluation of target lesions

Complete Response (CR): Disappearance of all target lesions. Any pathological lymph nodes (whether target or non-target) must have reduction in short axis to <10 mm.

Partial Response (PR): At least a 30% decrease in the sum of diameters of target lesions, taking as reference the baseline sum diameters.

Progressive Disease: At least a 20% increase in the sum of diameters of target lesions, taking as reference the smallest sum on study (this includes the baseline sum if that is the smallest on study). In addition to the relative increase of 20%, the sum must also demonstrate an absolute increase of at least 5 mm. (Note: the appearance of one or more new lesions is also considered progression).

Stable Disease (SD): Neither sufficient shrinkage to qualify for PR nor sufficient increase to qualify for progressive disease, taking as reference the smallest sum diameters while on study.

4.3.2 Special notes on the assessment of target lesions

Lymph nodes. Lymph nodes identified as target lesions should always have the actual short axis measurement recorded (measured in the same anatomical plane as the baseline examination), even if the nodes regress to below 10 mm on study. This means that when lymph nodes are included as target lesions, the 'sum' of lesions may not be zero even if complete response criteria are met, since a normal lymph node is defined as having a short axis of <10 mm. Case report forms or other data collection methods may therefore be designed to have target nodal lesions recorded in a separate section where, in order to qualify for CR, each node must achieve a short axis <10 mm. For PR, SD and progressive disease, the actual short axis measurement of the nodes is to be included in the sum of target lesions.

Target lesions that become 'too small to measure'. While on study, all lesions (nodal and non-nodal) recorded at baseline should have their actual measurements recorded at each subsequent evaluation, even when very small (eg 2 mm). However, sometimes lesions or lymph nodes which are recorded as target lesions at baseline become so faint on CT scan that the radiologist may not feel comfortable assigning an exact measure and may report them as being 'too small to measure'. When this occurs it is important that a value be recorded on the CRF. If it is the opinion of the radiologist that the lesion has likely disappeared, the measurement should be recorded as 0 mm. If the lesion is believed to be present and is faintly seen but too small to measure, a default value of 5 mm should be assigned (*Note*: It is less likely that this rule will be used for lymph nodes since they usually have a definable size when normal and are frequently surrounded by fat such as in the retroperitoneum; however, if a lymph node is believed to be present and is faintly seen but too small to measure, a default value of 5 mm should be assigned in this circumstance as well). This default value is derived from the 5 mm CT slice thickness (but should not be changed with varying CT slice thickness). The measurement of these lesions is potentially non-reproducible; therefore providing this default value will prevent false responses or progressions based upon measurement error. To reiterate, however, if the radiologist is able to provide an actual measure, that should be recorded, even if it is below 5 mm.

Lesions that split or coalesce on treatment. When non-nodal lesions 'fragment', the longest diameters of the fragmented portions should be added together to calculate the target lesion sum. Similarly, as lesions coalesce, a plane between them may be maintained that would aid in obtaining maximal diameter measurements of each individual lesion. If the lesions have truly coalesced such that they are no longer separable, the vector of the longest diameter in this instance should be the maximal longest diameter for the 'coalesced lesion'.

4.3.3 Evaluation of non-target lesions

This section provides the definitions of the criteria used to determine the tumor response for the group of non-target lesions. While some non-target lesions may actually be measurable, they need not be measured and instead should be assessed only qualitatively at the timepoints specified in the protocol.

Complete Response (CR): Disappearance of all non-target lesions and normalization of tumor marker level. All lymph nodes must be non-pathological in size (<10 mm short axis).

Non-CR/Non-progressive disease: Persistence of one or more non-target lesion(s) and/or maintenance of tumor marker level above the normal limits.

Progressive Disease: Unequivocal progression (see comments below) of existing non-target lesions. (*Note*: the appearance of one or more new lesions is also considered progression).

4.3.4 Special notes on assessment of progression of non-target disease

The concept of progression of non-target disease requires additional explanation as follows:

When the patient also has measurable disease. In this setting, to achieve 'unequivocal progression' on the basis of the non-target disease, there must be an overall level of substantial worsening in non-target disease such that, even in presence of SD or PR in target disease, the overall tumor burden has increased sufficiently to merit discontinuation of therapy. A modest 'increase' in the size of one or more non-target lesions is usually not sufficient to quality for unequivocal progression status. The designation of overall progression solely on the basis of change in non-target disease in the face of SD or PR of target disease will therefore be extremely rare.

When the patient has only non-measurable disease. This circumstance arises in some Phase studies when it is not a criterion of study entry to have measurable disease. The same general concepts apply here as noted above, however, in this instance there is no measurable disease assessment to factor into the interpretation of an increase in non-measurable disease burden. Because worsening in non-target disease cannot be easily quantified (by definition: if all lesions are truly non-measurable) a useful test that can be applied when assessing patients for unequivocal progression is to consider if the increase in overall disease burden based on the change in non-measurable disease is comparable in magnitude to the increase that would be required to declare progressive disease for measurable disease: i.e. an increase in tumor burden representing an additional 73% increase in 'volume' (which is equivalent to a 20% increase diameter in a measurable lesion). Examples include an increase in a pleural effusion from 'trace' to 'large', an increase in lymphangitic disease from localized to widespread, or may be described in protocols as 'sufficient to require a change in therapy.' If 'unequivocal progression' is seen, the patient should be considered to have had overall progressive disease at that point. While it would be ideal to have objective criteria to apply to non-measurable disease, the very nature of that disease makes it impossible to do so, therefore the increase must be substantial.

4.3.5 New lesions

The appearance of new malignant lesions denotes disease progression; therefore, some comments on detection of new lesions are important. There are no specific criteria for the identification of new radiographic lesions; however, the finding of a new lesion should be unequivocal: i.e. not attributable to differences in scanning technique, change in imaging modality or findings thought to represent something other than tumor (for example, some 'new' bone lesions may be simply healing or flare of pre-existing lesions). This is particularly important when the patient's baseline lesions show partial or complete response. For example, necrosis of a liver lesion may be reported on a CT scan report as a 'new' cystic lesion, which it is not.

A lesion identified on a follow-up study in an anatomical location that was not scanned at baseline is considered a new lesion and will indicate disease progression. An example of this is the patient who has

visceral disease at baseline and while on study has a CT or MRI brain ordered which reveals metastases. The patient's brain metastases are considered to be evidence of progressive disease even if he/she did not have brain imaging at baseline.

If a new lesion is equivocal, for example because of its small size, continued therapy and follow-up evaluation will clarify if it represents truly new disease. If repeat scans confirm there is definitely a new lesion, then progression should be declared using the date of the initial scan.

4.4.1 Timepoint response

It is assumed that at each protocol specified timepoint, a response assessment occurs. Table 1 in this attachment provides a summary of the overall response status calculation at each timepoint for patients who have measurable disease at baseline.

When patients have non-measurable (therefore non-target) disease only, Table 2 in this attachment is to be used.

4.4.2 Missing assessments and inevaluable designation

When no imaging/measurement is done at all at a particular timepoint, the patient is not evaluable (NE) at that timepoint. If only a subset of lesion measurements are made at an assessment, usually the case is also considered NE at that timepoint, unless a convincing argument can be made that the contribution of the individual missing lesion(s) would not change the assigned timepoint response. This would be most likely to happen in the case of progressive disease. For example, if a patient had a baseline sum of 50 mm with three measured lesions and at follow-up only two lesions were assessed, but those gave a sum of 80 mm, the patient will have achieved progressive disease status, regardless of the contribution of the missing lesion.

4.4.3 Best overall response: all timepoints

The *best overall response* is determined once all the data for the patient is known.

Best response determination in studies where confirmation of complete or partial response IS NOT required: Best response in these studies is defined as the best response across all timepoints (for example, a patient who has SD at first assessment, PR at second assessment, and progressive disease on last assessment has a best overall response of PR). When SD is believed to be best response, it must also meet the protocol specified minimum time from baseline. If the minimum time is not met when SD is otherwise the best timepoint response, the patient's best response depends on the subsequent assessments. For example, a patient who has SD at first assessment, progressive disease at second and does not meet minimum duration for SD, will have a best response of progressive disease. The same patient lost to follow-up after the first SD assessment would be considered inevaluable.

Table 1 - Timepoint response: patients with Target (+/- non-target) disease

Target lesions	Non-target lesions	New lesions	Overall response
CR	CR	No	CR
CR	Non-CR/non-PD	No	PR
CR	Not evaluated	No	PR
PR	Non-PD or not all evaluated	No	PR
SD	Non-PD or not all evaluated	No	SD
Not all evaluated	Non-PD	No	NE
PD	Any	Yes or No	PD
Any	PD	Yes or No	PD
Any	Any	Yes	PD

CR = complete response; PR = partial response; SD = stable disease; PD = progressive disease; NE = not evaluable.

Table 2 - Timepoint response: patients with non-target disease only

Non-target lesions	New lesions	Overall response
CR	No	CR
Non-CR/non-PD	No	Non-CR/non-PD[a]
Not all evaluated	No	NE
Unequivocal PD	Yes or No	PD
Any	Yes	PD

CR = complete response; PD = progressive disease; NE = not evaluable.

a ‘Non-CR/non-PD’ is preferred over ‘stable disease’ for non-target disease since SD is increasingly used as endpoint for assessment of efficacy in some studies so to assign this category when no lesions can be measured is not advised.

Attachment 2: BPI-SF

1903

Date: ☐☐ / ☐☐ / ☐☐
(month) (day) (year)

Subject's Initials : ______________

Study Subject #: ☐☐☐☐

PLEASE USE BLACK INK PEN

Study Name: ______________________________

Protocol #: ______________________________

PI: ______________________________

Revision: 07/01/05

Brief Pain Inventory (Short Form)

1. Throughout our lives, most of us have had pain from time to time (such as minor headaches, sprains, and toothaches). Have you had pain other than these everyday kinds of pain today?

☐ Yes ☐ No

2. On the diagram, shade in the areas where you feel pain. Put an X on the area that hurts the most.

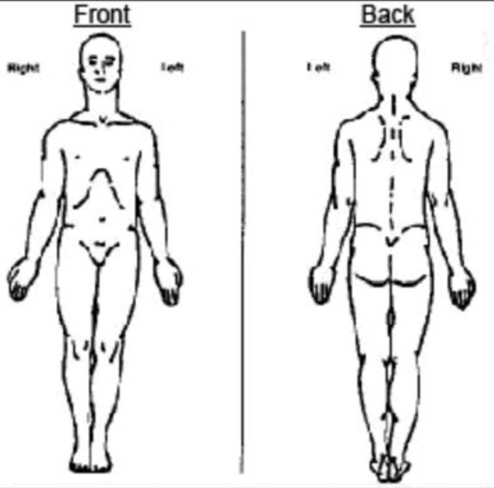

3. Please rate your pain by marking the box beside the number that best describes your pain at its worst in the last 24 hours.

☐ 0 ☐ 1 ☐ 2 ☐ 3 ☐ 4 ☐ 5 ☐ 6 ☐ 7 ☐ 8 ☐ 9 ☐ 10

No Pain — Pain As Bad As You Can Imagine

4. Please rate your pain by marking the box beside the number that best describes your pain at its least in the last 24 hours.

☐ 0 ☐ 1 ☐ 2 ☐ 3 ☐ 4 ☐ 5 ☐ 6 ☐ 7 ☐ 8 ☐ 9 ☐ 10

No Pain — Pain As Bad As You Can Imagine

5. Please rate your pain by marking the box beside the number that best describes your pain on the average.

☐ 0 ☐ 1 ☐ 2 ☐ 3 ☐ 4 ☐ 5 ☐ 6 ☐ 7 ☐ 8 ☐ 9 ☐ 10

No Pain — Pain As Bad As You Can Imagine

6. Please rate your pain by marking the box beside the number that tells how much pain you have right now.

☐ 0 ☐ 1 ☐ 2 ☐ 3 ☐ 4 ☐ 5 ☐ 6 ☐ 7 ☐ 8 ☐ 9 ☐ 10

No Pain — Pain As Bad As You Can Imagine

Page 1 of 2

Copyright 1991 Charles S. Cleeland, PhD
Pain Research Group
All rights reserved

1903

PLEASE USE BLACK INK PEN

Date: ☐☐ / ☐☐ / ☐☐
(month) (day) (year)

Subject's Initials : ______________

Study Subject #: ☐☐☐☐

Study Name: ______________________________

Protocol #: ______________________________

PI: ______________________________

Revision: 07/01/05

7. What treatments or medications are you receiving for your pain?

8. In the last 24 hours, how much relief have pain treatments or medications provided? Please mark the box below the percentage that most shows how much relief you have received.

0%	10%	20%	30%	40%	50%	60%	70%	80%	90%	100%
☐ No Relief	☐	☐	☐	☐	☐	☐	☐	☐	☐	☐ Complete Relief

9. Mark the box beside the number that describes how, during the past 24 hours, pain has interfered with your:

A. General Activity

☐ 0 Does Not Interfere ☐ 1 ☐ 2 ☐ 3 ☐ 4 ☐ 5 ☐ 6 ☐ 7 ☐ 8 ☐ 9 ☐ 10 Completely Interferes

B. Mood

☐ 0 Does Not Interfere ☐ 1 ☐ 2 ☐ 3 ☐ 4 ☐ 5 ☐ 6 ☐ 7 ☐ 8 ☐ 9 ☐ 10 Completely Interferes

C. Walking ability

☐ 0 Does Not Interfere ☐ 1 ☐ 2 ☐ 3 ☐ 4 ☐ 5 ☐ 6 ☐ 7 ☐ 8 ☐ 9 ☐ 10 Completely Interferes

D. Normal Work (includes both work outside the home and housework)

☐ 0 Does Not Interfere ☐ 1 ☐ 2 ☐ 3 ☐ 4 ☐ 5 ☐ 6 ☐ 7 ☐ 8 ☐ 9 ☐ 10 Completely Interferes

E. Relations with other people

☐ 0 Does Not Interfere ☐ 1 ☐ 2 ☐ 3 ☐ 4 ☐ 5 ☐ 6 ☐ 7 ☐ 8 ☐ 9 ☐ 10 Completely Interferes

F. Sleep

☐ 0 Does Not Interfere ☐ 1 ☐ 2 ☐ 3 ☐ 4 ☐ 5 ☐ 6 ☐ 7 ☐ 8 ☐ 9 ☐ 10 Completely Interferes

G. Enjoyment of life

☐ 0 Does Not Interfere ☐ 1 ☐ 2 ☐ 3 ☐ 4 ☐ 5 ☐ 6 ☐ 7 ☐ 8 ☐ 9 ☐ 10 Completely Interferes

Page 2 of 2

Copyright 1991 Charles S. Cleeland, PhD
Pain Research Group
All rights reserved

Attachment 3: ECOG Performance Status

Grade	ECOG
0	Fully active, able to carry on all pre-disease performance without restriction
1	Restricted in physically strenuous activity but ambulatory and able to carry out work of a light or sedentary nature, eg, light house work, office work
2	Ambulatory and capable of all selfcare but unable to carry out any work activities. Up and about more than 50% of waking hours
3	Capable of only limited selfcare, confined to bed or chair more than 50% of waking hours
4	Completely disabled. Cannot carry on any selfcare. Totally confined to bed or chair
5	Dead

As published in Oken MM, Creech RH, Tormey DC, Horton J, Davis TE, McFadden ET, Carbone PP. Toxicity And Response Criteria Of The Eastern Cooperative Oncology Group (1982). Am J Clin Oncol 5:649-655.

Eastern Cooperative Oncology Group, Robert Comis M.D., Group Chair.

Attachment 4: EQ-5D-5L (Sample UK English Version)

Under each heading, please tick the ONE box that best describes your health TODAY

MOBILITY

- I have no problems in walking about ☐
- I have slight problems in walking about ☐
- I have moderate problems in walking about ☐
- I have severe problems in walking about ☐
- I am unable to walk about ☐

SELF-CARE

- I have no problems washing or dressing myself ☐
- I have slight problems washing or dressing myself ☐
- I have moderate problems washing or dressing myself ☐
- I have severe problems washing or dressing myself ☐
- I am unable to wash or dress myself ☐

USUAL ACTIVITIES *(e.g. work, study, housework, family or leisure activities)*

- I have no problems doing my usual activities ☐
- I have slight problems doing my usual activities ☐
- I have moderate problems doing my usual activities ☐
- I have severe problems doing my usual activities ☐
- I am unable to do my usual activities ☐

PAIN / DISCOMFORT

- I have no pain or discomfort ☐
- I have slight pain or discomfort ☐
- I have moderate pain or discomfort ☐
- I have severe pain or discomfort ☐
- I have extreme pain or discomfort ☐

ANXIETY / DEPRESSION

- I am not anxious or depressed ☐
- I am slightly anxious or depressed ☐
- I am moderately anxious or depressed ☐
- I am severely anxious or depressed ☐
- I am extremely anxious or depressed ☐

SAMPLE

2

UK (English) v.2 © 2009 EuroQol Group. EQ-5D™ is a trade mark of the EuroQol Group

- We would like to know how good or bad your health is TODAY.
- This scale is numbered from 0 to 100.
- 100 means the best health you can imagine.
  0 means the worst health you can imagine.
- Mark an X on the scale to indicate how your health is TODAY.
- Now, please write the number you marked on the scale in the box below.

YOUR HEALTH TODAY =

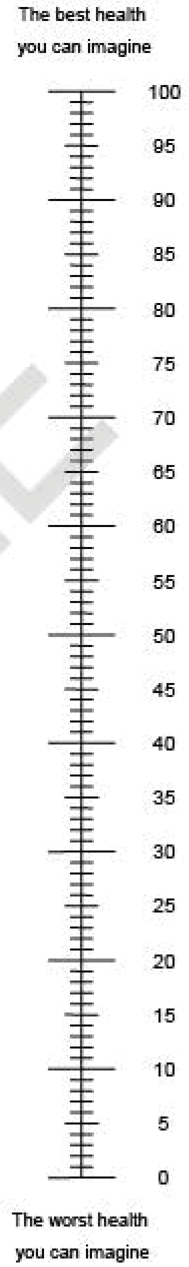

SAMPLE

3

UK (English) v.2 © 2009 EuroQol Group. EQ-5D™ is a trade mark of the EuroQol Group

Obtained from:
http://www.euroqol.org/fileadmin/user_upload/Documenten/PDF/Languages/Sample_UK__English__EQ-5D-5L.pdf (accessed 9 July 2012)

Attachment 5: FACT-P (Sample UK English Version)

Below is a list of statements that other people with your illness have said are important. **Please circle or mark one number per line to indicate your response as it applies to the past 7 days.**

	**PHYSICAL WELL-BEING**	**Not at all**	**A little bit**	**Some-what**	**Quite a bit**	**Very much**
GP1	I have a lack of energy	0	1	2	3	4
GP2	I have nausea	0	1	2	3	4
GP3	Because of my physical condition, I have trouble meeting the needs of my family	0	1	2	3	4
GP4	I have pain	0	1	2	3	4
GP5	I am bothered by side effects of treatment	0	1	2	3	4
GP6	I feel ill	0	1	2	3	4
GP7	I am forced to spend time in bed	0	1	2	3	4

	**SOCIAL/FAMILY WELL-BEING**	**Not at all**	**A little bit**	**Some-what**	**Quite a bit**	**Very much**
GS1	I feel close to my friends	0	1	2	3	4
GS2	I get emotional support from my family	0	1	2	3	4
GS3	I get support from my friends	0	1	2	3	4
GS4	My family has accepted my illness	0	1	2	3	4
GS5	I am satisfied with family communication about my illness	0	1	2	3	4
GS6	I feel close to my partner (or the person who is my main support)	0	1	2	3	4
Q1	*Regardless of your current level of sexual activity, please answer the following question. If you prefer not to answer it, please mark this box* ☐ *and go to the next section.*					
GS7	I am satisfied with my sex life	0	1	2	3	4

Please circle or mark one number per line to indicate your response as it applies to the <u>past 7 days</u>.

	**<u>EMOTIONAL WELL-BEING</u>**	**Not at all**	**A little bit**	**Some-what**	**Quite a bit**	**Very much**
GE1	I feel sad	0	1	2	3	4
GE2	I am satisfied with how I am coping with my illness	0	1	2	3	4
GE3	I am losing hope in the fight against my illness	0	1	2	3	4
GE4	I feel nervous	0	1	2	3	4
GE5	I worry about dying	0	1	2	3	4
GE6	I worry that my condition will get worse	0	1	2	3	4

	**<u>FUNCTIONAL WELL-BEING</u>**	**Not at all**	**A little bit**	**Some-what**	**Quite a bit**	**Very much**
GF1	I am able to work (include work at home)	0	1	2	3	4
GF2	My work (include work at home) is fulfilling	0	1	2	3	4
GF3	I am able to enjoy life	0	1	2	3	4
GF4	I have accepted my illness	0	1	2	3	4
GF5	I am sleeping well	0	1	2	3	4
GF6	I am enjoying the things I usually do for fun	0	1	2	3	4
GF7	I am content with the quality of my life right now	0	1	2	3	4

Please circle or mark one number per line to indicate your response as it applies to the past 7 days.

	ADDITIONAL CONCERNS	Not at all	A little bit	Some-what	Quite a bit	Very much
C2	I am losing weight	0	1	2	3	4
C6	I have a good appetite	0	1	2	3	4
P1	I have aches and pains that bother me	0	1	2	3	4
P2	I have certain parts of my body where I experience pain	0	1	2	3	4
P3	My pain keeps me from doing things I want to do	0	1	2	3	4
P4	I am satisfied with my present comfort level	0	1	2	3	4
P5	I am able to feel like a man	0	1	2	3	4
P6	I have trouble moving my bowels	0	1	2	3	4
P7	I have difficulty urinating	0	1	2	3	4
BL2	I urinate more frequently than usual	0	1	2	3	4
P8	My problems with urinating limit my activities	0	1	2	3	4
BL5	I am able to have and maintain an erection	0	1	2	3	4

Obtained from: http://www.facit.org/FACITOrg/Questionnaires (accessed 9 July 2012)

Attachment 6: Protocol Specific PCWG2 Criteria

Progressive Disease after Androgen Deprivation Eligibility Criteria:
The reference value (#1) is the last PSA measured before increases are documented, with subsequent values obtained a minimum of 1 week apart. If the PSA at time point 3 (value #3A) is greater than that at point 2, then eligibility has been met. If the PSA is not greater than point 2 (value #3B), but value #4 is, the patient is eligible assuming that other criteria are met, if values 3A or #4 are 2 ng/mL or higher.

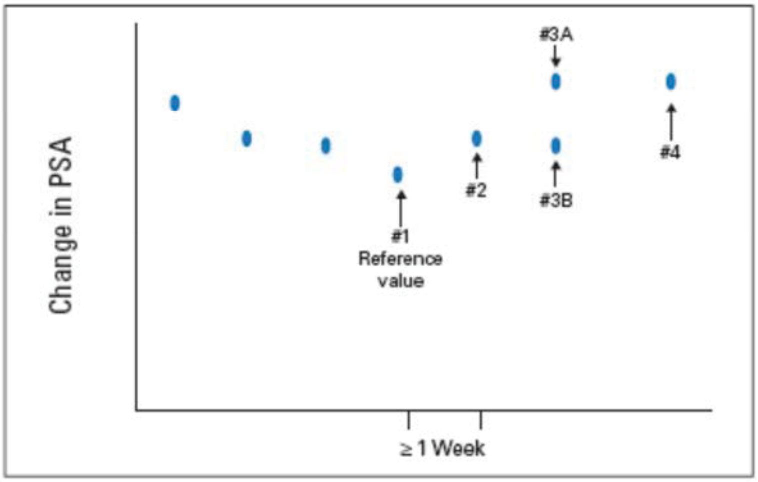

Eligibility based on PSA

Attachment 7: NCI CTCAE v4.0

Common Terminology Criteria for Adverse Events (CTCAE) and Common Toxicity Criteria (CTC) v4.0. National Cancer Institute Web site:

http://ctep.cancer.gov/protocolDevelopment/electronic_applications/ctc.htm. Accessed October 10, 2012.

LAST PAGE

Janssen Medical Affairs EMEA

Statistical Analysis Plan
Main Treatment analysis

A Randomized Phase 2 Study Evaluating Abiraterone Acetate With Different Steroid Regimens for Preventing Symptoms Associated With Mineralocorticoid Excess in Asymptomatic, Chemotherapy-Naïve and Metastatic Castration-Resistant Prostate Cancer(mCRPC) Patients

---

Protocol 212082PCR2023; Phase II

JNJ-212082 (abiraterone acetate)

Status: Approved
Date: 10 February 2017
Prepared by: Janssen EMEA
Document No.: EDMS-insert EDMS number

Compliance: The study described in this report was performed according to the principles of Good Clinical Practice (GCP).

Confidentiality Statement
The information in this document contains trade secrets and commercial information that are privileged or confidential and may not be disclosed unless such disclosure is required by applicable law or regulations. In any event, persons to whom the information is disclosed must be informed that the information is privileged or confidential and may not be further disclosed by them. These restrictions on disclosure will apply equally to all future information supplied to you that is indicated as privileged or confidential.

**Status**	☒ Draft	☐ Initial	☐ Final
**Protocol Title:**	**A Randomized Phase 2 Study Evaluating Abiraterone Acetate With Different Steroid Regimens for Preventing Symptoms Associated With Mineralocorticoid Excess in Asymptomatic, Chemotherapy-Naïve and Metastatic Castration-Resistant Prostate Cancer(mCRPC) Patients**		
**Protocol Number:**	**Protocol 212082PCR2023, Phase II**		
**Contents**	**Specification of planned analysis**		

Prepared By:

Study Statistician
Marjolein Lahaye

Name	Signature	Date

Approved By:

Study Responsible Physician
Florence Lefresne

Name	Signature	Date

Compliance: The study described in this report was performed according to the principles of Good Clinical Practice (GCP).

Confidentiality Statement

The information in this document contains trade secrets and commercial information that are privileged or confidential and may not be disclosed unless such disclosure is required by applicable law or regulations. In any event, persons to whom the information is disclosed must be informed that the information is privileged or confidential and may not be further disclosed by them. These restrictions on disclosure will apply equally to all future information supplied to you that is indicated as privileged or confidential.

TABLE OF CONTENTS

AMENDMENT HISTORY 5

ABBREVIATIONS 5

1. INTRODUCTION 7
1.1. Trial Objectives 7
1.2. Trial Design 8
1.3. Statistical Hypotheses for Trial Objectives 8
1.4. Sample Size Justification 8
1.5. Randomization and Blinding 9

2. GENERAL ANALYSIS DEFINITIONS 9
2.1. Visit Windows 9
2.2. Pooling Algorithm for Analysis Centers 10
2.3. Analysis Sets 10
2.3.1. Efficacy Analysis Set 11
2.3.2. Safety Analysis Set 11
2.4. Definition of Subgroups 11

3. INTERIM ANALYSIS AND DATA MONITORING COMMITTEE REVIEW 11

4. SUBJECT INFORMATION 11
4.1. Subjects excluded from the Intention-to-Treat population 11
4.2. Demographics and Baseline Characteristics 11
4.2.1. Demographics 11
4.2.2. Baseline Prostate Cancer Characteristics 12
4.3. Prior Cancer-Related Therapy/Surgery 12
4.4. ECOG Performance Status at Baseline 13
4.5. BPI-SF at Baseline 13
4.6. Clinical Laboratory Tests at Baseline 13
4.6.1. Hematology 13
4.6.2. Chemistry 13
4.6.3. Urinalysis-Dipstick 13
4.7. Left Ventricular Ejection 14
4.8. ECG Results 14
4.9. Medical History 14
4.10. Physical Examination 14
4.11. Disposition Information 14
4.12. Major Protocol Deviations 14
4.13. Extent of Exposure 14
4.14. Concomitant Medication 15

5. EFFICACY 15
5.1. Analysis Specifications 15
5.1.1. Level of Significance 16
5.1.2. General Imputation Methods 16
5.2. Efficacy Endpoint(s) 16
5.2.1. PSA Response Rate 16
5.2.1.1. Definition 16
5.2.1.2. Analysis Methods 16
5.2.2. Eastern Cooperative Oncology Group (ECOG) Performance Status 17
5.2.2.1. Definition 17
5.2.2.2. Analysis Methods 17
5.2.2.3. Imputation Method 17
5.2.3. Evaluation of response 17
5.2.3.1. Definition 17
5.2.3.2. Analysis Methods 17

5.2.4. Best response ... 18
5.2.4.1. Definition ... 18
5.2.4.2. Analysis method ... 18
5.2.5. Duration of response in Subjects with Measurable Disease ... 18
5.2.5.1. Definition ... 18
5.2.5.2. Analysis method ... 18
5.2.6. New bone lesions and Progressed by Bone Scan ... 18
5.2.6.1. Definition ... 18
5.2.6.2. Analysis method ... 18
5.2.7. Progression-Free Survival ... 18
5.2.7.1. Definition ... 18
5.2.7.2. Analysis method ... 19
5.2.8. Skeletal related events ... 19
5.2.8.1. Definition ... 19
5.2.8.2. Analysis method ... 19
5.2.9. Overall survival ... 19
5.2.9.1. Definition ... 19
5.2.9.2. Analysis method ... 19
5.2.10. Time to Opiate Use for Cancer-related Pain ... 19
5.2.10.1. Definition ... 19
5.2.10.2. Analysis method ... 19
5.2.11. Patient-Reported Outcomes ... 20
5.2.11.1. Brief Pain Inventory – Short form (BPI-SF) ... 20
5.2.11.1.1. Definition ... 20
5.2.11.1.2. Analysis Methods ... 20
5.2.11.1.3. Imputation Method ... 21
5.2.11.2. EQ-5D-5L ... 21
5.2.11.2.1. Definition ... 21
5.2.11.2.2. Analysis Methods ... 21
5.2.11.2.3. Imputation Method ... 22
5.2.11.3. Functional Assessment of Cancer Therapy – Prostate (FACT-P) ... 22
5.2.11.3.1. Definition ... 22
5.2.11.3.2. Analysis Methods ... 22
5.2.11.3.3. Imputation Method ... 22

6. SAFETY ... 23
6.1. Adverse Events ... 23
6.1.1. Primary endpoint ... 23
6.1.2. Secondary endpoint ... 23
6.2. Clinical Laboratory Tests ... 25
6.2.1. Plasma ACTH ... 25
6.2.2. Serum androgens ... 25
6.2.3. Androgen precursors profile ... 25
6.2.4. Chemistry Tests ... 26
6.2.5. Insulin resistance ... 26
6.2.6. Fasting serum lipids ... 26
6.3. DXA scans ... 27
6.4. Body weight ... 27
6.5. Vital signs ... 27
6.6. Biomarkers ... 28
6.7. Medical Resource Utilization ... 28

REFERENCES ... 28

ATTACHMENTS ... 29

1. LIST OF PREFERRED TERMS FOR SMQS ... 29

2. PRIMARY ANALYSIS SAP ... 49

AMENDMENT HISTORY

ABBREVIATIONS

ACTH	Adrenocorticotrophic Hormone
AE	Adverse event
ALP	Alkaline phosphatase
ALT	Alanine aminotransferase
BMI	Body Mass Index
BPI-SF	Brief Pain Inventory-Short Form
AST	Aspartate aminotransferase
CI	Confidence interval
CR	Complete Response
CRF	Case report form
CRPC	Castration-Resistant Prostate Cancer
CSR	Clinical Study Report
CT	Computed Tomography
CTCAE	Common Terminology Criteria for Adverse Events
DMC	Data Monitoring Committee
DHEA	Dehydroepiandrosterone
DHEA-S	Dehydroepiandrosterone-sulphate
DHT	Dihydrotestosterone
DBP	Diastolic blood pressure
ECG	Electrocardiogram
DXA	Dual-energy X-ray absorptiometry
ECOG	Eastern Cooperative Oncology Group
eCRF	Electronic case report form
EOE	End of extension
EOMS	End-of-main-study
EOMT	End-of-main-study-treatment
EQ-5D-(5L)	European Quality of Life-5 Dimensions, 5 Levels Questionnaire
FACT-P	Functional Assessment of Cancer Therapy - Prostate Cancer
GnRH	Gonadotrophin-releasing hormone
ICU	Intensive Care Unit
INR	International Normalized Ratio
ITT	Intent-to-Treat
IVRS	Interactive voice response system
LDH	Lactate Dehydrogenase
LOCF	Last observation carried forward
MedDRA	Medical Dictionary for Regulatory Activities
mCRPC	Metastatic Castration-Resistant Prostate Cancer
mITT	Modified Intent-to-Treat
MRI	Magnetic resonance imaging
MRU	Medical Resource Utilization
MSTP	Main Study Treatment Phase
NA	Not Applicable
NE	Not Evaluable
OS	Overall survival
PCWG2	Prostate Cancer Working Group
PD	Progression disease
PI	Principal investigator
PK	Pharmacokinetic(s)
PR	Partial Response
PSA	Prostate-specific antigen
PFS	Progression-free-survival
PT	Preferred term (MedDRA)

PT	Prothrombin Time
PTT	Partial Thromboplastin Time
QoL	Quality of Life
RBC	Red Blood Cells
RECIST	Response Evaluation Criteria In Solid Tumors
SAE	Serious adverse event
SAP	Statistical Analysis Plan
SD	Stable Disease
SD	Standard deviation
SE	Standard error
SOC	System organ class (MedDRA)
SBP	Systolic blood pressure
TEAE	Treatment emergent adverse event
TNM	Classification of Malignant Tumours
VAS	Visual Analogue Scale
WBC	White Blood Cells
WHO	World Health Organization

1. INTRODUCTION

This Statistical Analysis Plan (SAP) describes the general methodological aspects of the Main Treatment phase analysis of the Alternative Steroid study (protocol number Protocol 212082-PCR-2023). This is a randomized, open-label, parallel-arm, multicenter, phase 2 study of treatment with abiraterone acetate and 4 alternative steroid treatment strategies. The Main Study Treatment analysis will be done when all subjects have ended the Main Study Treatment Phase (MSTP) with a maximum of 39 cycles (156 weeks).

1.1. Trial Objectives

Primary objective

The primary objective of this study is to evaluate the safety of abiraterone acetate with 4 alternative steroid treatment strategies related to symptoms associated with mineralocorticoid excess toxicities (ie, hypokalemia and/or hypertension) during the first 24 weeks of treatment in asymptomatic, chemotherapy-naïve, mCRPC subjects.

Secondary Objectives

The following secondary objectives will be assessed during the entire study in asymptomatic, chemotherapy-naïve and mCRPC subjects treated with abiraterone acetate and 4 alternative steroid treatment strategies:

- To further characterize the global safety profile (including the incidence of mineralocorticoid excess toxicities [eg, hypokalemia and hypertension]
- To characterize mid-term and long-term exogenous glucocorticoid side effects.
- To characterize the clinical benefit.
- To evaluate the impact on pain and quality of life (QoL) as measured by the EQ-5D-5L, Brief Pain Inventory - short form (BPI-SF) and Functional Assessment of Cancer Therapy - Prostate Cancer (FACT-P) tools.
- To collect medical resource utilization (MRU) data that may be used in future economic modeling (the construction and reporting of the economic model will be conducted separately from this study).
- To evaluate overall survival.
- To collect data on subsequent therapies for prostate cancer (time to next therapy for prostate cancer, time to initiation of chemotherapy, treatment duration, best response) following cessation of study treatment.
- Additionally, the study includes optional biomarker analyses of circulating genetic material as a secondary exploratory objective to:

- Identify genomic aberrations in circulating plasma deoxyribonucleic acid (DNA), and evaluate expression of circulating plasma and whole blood mRNA and microRNA (miRNA) occurring pre- and on-treatment.
- Analyze circulating biomarkers as predictors of response or sensitivity to abiraterone acetate or for identifying mechanisms of resistance.

1.2. Trial Design

This is a randomized, open-label, parallel-arm, multicenter, phase 2 study of treatment with abiraterone acetate and 4 alternative steroid treatment strategies in asymptomatic, chemotherapy-naïve, mCRPC subjects.

The subject population will comprise medically or surgically castrated men aged 18 years or older with mCRPC who have shown tumor progression and are asymptomatic by prospectively defined criteria. A target of 144 subjects will be enrolled in this study, with 36 subjects planned per treatment arm.

The study will consist of a screening phase of 4 weeks followed by an open-label MSTP of a maximum of 39 cycles (156 weeks), with an extension phase for progression-free subjects (if applicable), followed by a follow-up phase. The MSTP cut-off date will be 156 weeks after the start of study treatment for the first subject participating in the study, with the EOMT performed by 16 July 2016 ± 15 days. Subjects will participate in the MSTP until the cut-off date; study treatment will be administered until radiographic disease progression, and/or unequivocal clinical progression, and/or other reasons for discontinuation.

End-of-main-study-treatment (EOMT) assessments will be performed for all subjects randomized into the MSTP, regardless of whether they complete study treatment up to the cut-off date or discontinue from the MSTP before the cut-off date. Additionally, for subjects discontinuing study treatment before the cut-off date, an end-of-main-study (EOMS) visit will be performed 4 weeks after the cessation of study medication.

Subjects will be randomized in a 1:1:1:1 ratio to receive abiraterone acetate 1,000 mg once daily with either prednisone 5 mg twice daily (Arm 1), prednisone 5 mg once daily (Arm 2), prednisone 2.5 mg twice daily (Arm 3) or dexamethasone 0.5 mg once daily (Arm 4).

1.3. Statistical Hypotheses for Trial Objectives

It will be determined whether the 4 treatment arms warrant further study based on the "no mineralocorticoid excess" rate. “No mineralocorticoid excess” is defined as experiencing (yes or no) neither of the 2 mineralocorticoid excess toxicities, ie, neither hypokalemia nor hypertension, during the first 24 weeks of treatment. The highest percentage of subjects experiencing neither hypokalemia nor hypertension during the first 24 weeks at which it is required to reject the treatment is 50% and the treatment would be worth developing further if this rate is 75%.

1.4. Sample Size Justification

The primary parameter is defined as experiencing (yes or no) neither of the 2 mineralocorticoid excess toxicities during the first 24 weeks of treatment, ie, neither hypokalemia nor hypertension. For this

study the assumption is that 75% of the subjects within each treatment arm will not experience any of the events defined in the primary parameter, ie, hypokalemia and/or hypertension, during 24 weeks of treatment. Fifty percent is defined as the highest percentage to reject the treatment. An exact binomial test with a 5% one-sided significance level will have 89% power to detect the difference between 50% and 75% when the sample size is 30. Assuming 15% of non-evaluable subjects, 36 subjects per treatment arm will be included. The sample size calculation makes clear that this study is powered to explore which arm(s) would be worth developing further, not to compare the four arms.

1.5. Randomization and Blinding

Central randomization will assign subjects in a 1:1:1:1 ratio to one of the four treatment 4 arms based on a computer-generated randomization schedule. The randomization will be balanced by using randomly permuted blocks. No stratification will be used. As this is an open-label study no procedures for maintaining the blind is needed.

2. GENERAL ANALYSIS DEFINITIONS

2.1. Visit Windows

The scheduled visits are given in the table below. All assignments to these visits are made in chronological order. When a subject ends the MSTP prematurely, the EOMT visit should be filled out. At the same time, the collected information at this endpoint visit will also be assigned to the visit which should have been taken place according to the visit schedule, with two exceptions:

1. No information will be assigned when there is already a visit in that time interval

2. Only the information will be assigned to the visit in that time interval that is normally collected on that visit according to protocol.

For example: a subject progresses or discontinues treatment at day 75 and all information on the EOMT visit has been collected. According to the time interval in the table below, information should also be assigned to visit Cycle 4 (day 84), because no data was collected for that visit. This applies only to information normally collected on this visit, so for instance, assessment on Vital Signs and ECOG should be assigned to that visit, but for BPI-SF or EQ-5D-5L it should not. All assessments done at the EOMT visit will however be assigned to the main study treatment endpoint visit.

Scheduled Visit Number[a]	Time Interval (label on output)	Time Interval[c] (Day)	Target Time Point
1.0	Screening	-28 to 0	-28
2.0	Cycle 1, day 1[b]	1	1
3.0	Cycle 1, day 15	8-22	15
4.0	Cycle 2, day 1	23-42	29
6.0	Cycle 3, day 1	43-70	57
8.0	Cycle 4, day 1	71-98	85
9.0	Cycle 5, day 1	99-126	113
10.0	Cycle 6, day 1	127-182	141
11.0	Cycle 9, day 1	183-266	225
12.0	Cycle 12, day 1	267-350	309
13.0	Cycle 15, day 1	351-434	393
14.0	Cycle 18, day 1	435-518	477
15.0	Cycle 21, day 1	519-602	561
16.0	Cycle 24, day 1	603-686	645
17.0	Cycle 27, day 1	687-770	729
18.0	Cycle 30, day 1	771-854	813
19.0	Cycle 33, day 1	855-938	897
20.0	Cycle 36, day 1	939-1022	981
21.0	Cycle 39, day 1	1023-1078	1065
22.0	EOMT	1079-	1093

a. Visit numbering used in the data entry system
b. Day 1, Cycle 1 is initiation of study treatment
c. Visits are scheduled within ± 2 days of the scheduled target day up to and including cycle 6 and ± 6 days of the scheduled target day after Cycle 6. This column only shows the time interval for the visit to which an early termination visit (EOMT) will be rescheduled when it takes place within this time interval (unless a scheduled visit already exists within this time interval).

Definition of baseline

The last visit prior to the first study treatment will be considered as the baseline visit. This can be the screening visit if assessment is not done at Cycle 1 day 1, otherwise it will be the Cycle 1 day 1 visit.

Definition of main treatment endpoint

For each parameter the last available post-baseline visit within the MSTP with non-missing data for that parameter will be considered as the main study treatment endpoint visit.

2.2. Pooling Algorithm for Analysis Centers

Pooling subsets of centers for analysis was not planned for in the protocol.

2.3. Analysis Sets

All enrolled subjects who provide their written consent for data collection and who are randomized, regardless of whether they received any study treatment, will be included in the Intention-to-treat (ITT) analysis set. This analysis set will be used for all analyses of disposition, demographic, and baseline disease characteristics.

For subjects who provide their written informed consent but are not randomized only demographic data will be summarized descriptively.

If more than 10% of the subjects have major protocol deviations, an additional per-protocol analysis may be performed.

2.3.1. Efficacy Analysis Set

The ITT analysis set will be used for all analysis on efficacy parameters. Additionally, where appropriate, the efficacy analysis may be performed including only randomized subjects who receive at least 1 dose of abiraterone acetate plus prednisone or dexamethasone and have at least 1 post-baseline efficacy assessment (modified intent-to-treat [mITT] efficacy analysis set).

2.3.2. Safety Analysis Set

The safety analysis set will include all randomized subjects who received at least one dose of any study drug (i.e. either abiraterone acetate or steroid). All safety analysis will be performed on the safety analysis set.

2.4. Definition of Subgroups

No subgroups are foreseen.

3. INTERIM ANALYSIS AND DATA MONITORING COMMITTEE REVIEW

No interim analysis will be performed. No DMC review will take place. The primary analysis which is based on the first 24 weeks of the MSTP is performed to describe the results of the primary objective and some secondary objectives. The corresponding SAP is attached to this SAP.

4. SUBJECT INFORMATION

4.1. Subjects excluded from the Intention-to-Treat population

For the subjects excluded from the ITT population, the reason for exclusion will be summarized and limited demographics will be presented, i.e.:

- Demographics (Age, Medical history)
- In- and exclusion criteria

4.2. Demographics and Baseline Characteristics

Descriptive statistics on the baseline demographic characteristics described below will be presented for the ITT analysis set and data will be summarized overall and per treatment arm. For continuous and ordinal data descriptive statistics (mean, standard deviation, median, range and 95% CI) will be provided. Categorical data will be summarized as frequency distribution.

4.2.1. Demographics

Descriptive statistics will be provided for Age, Height, Weight, BMI, Systolic/Diastolic blood pressure, Heart rate, Respiratory rate and temperature at baseline. Number of subjects with hypertension stage 1 (i.e. $140 \leq SBP < 160$ or $90 \leq DBP < 100$) or stage 2 and above (i.e. $SBP \geq 160$ or $DBP \geq 100$) at

baseline will be calculated. BMI will be calculated as (weight at baseline)/(height at screening)^2 (kg/m2). Race, both Alcohol consumption and Tobacco use (Never used, Current user, Former user), Age class (<65, 65-74, 75-84, ≥85), number of subject with hypertension stage 1 as well as with stage 2 and above and BMI class (<25, 25-30, >30) will be summarized as frequency distribution per treatment arm.

4.2.2. Baseline Prostate Cancer Characteristics

Descriptive statistics will be provided for the following baseline parameters assessed:

- Time from initial prostate cancer diagnosis to randomization (months)
- Time from start of previous radiotherapy for treatment of prostate cancer to randomization (months), overall and per type of radiotherapy (External Beam Therapy, Brachytherapy, Other)
- Time from start of previous systemic therapy as well as time from start of previous GnRH agonist and previous GnRH antagonist therapy to randomization (months)
- Time from start of previous surgery since diagnosis to randomization (months)

The following baseline parameters will be summarized as frequency distribution:

- Histopathological grade (GX, G1, G2, G3, G4, Unknown or not done)
- Primary Gleason score (1,2,3,4,5)
- Secondary Gleason score (1,2,3,4,5)
- Total Gleason score at initial diagnosis using the following classification (≤7, ≥8).
- TNM Classification at Diagnosis: Tx, T0-T4; Nx, N0, N1; Mx, M0, M1
- Stage at Diagnosis (I, II, III, IV)
- Extent of disease at baseline will be summarized as frequency distribution
    - Bone
    - Lymph node
    - Visceral disease (lung or liver)
        - Liver
        - Lung
        - Lung and liver
    - Other (including all other sites)
- Disease localization at baseline: bone only, soft tissue only, bone and soft tissue (with soft tissue defined as lymph nodes and/or liver and/or lung and/or other softs tissue)
- Target and non-target disease at baseline
    - Target only
    - Non-target only
    - Measurable disease (defined as having at least one target lesion at baseline identified per RECIST)
- Number of bone metastases at baseline 0, 1 - 4, 5 - 10, >10

4.3. Prior Cancer-Related Therapy/Surgery

The number of subjects that received prior prostate cancer radiotherapy (External Beam Therapy, Brachytherapy, Other), prior systemic therapy, i.e. prior GnRH agonist, GnRH antagonist, antiandrogen therapy (flutamide, bicalutamide, nilutamide, other antiandrogen), Bone targeted agent (biposphonate,

denosumab, Other) and other systemic therapy, prior cancer-related surgery (orchiectomy, radical prostatectomy, Other), and the number of prior antiandrogen therapies will be summarized as frequency distribution. Number of subjects that had previous surgery for the indication under study since diagnosis and type of surgery/procedure (Orchiectomy, Radical prostatectomy, Other) will be summarized as frequency distribution.

4.4. ECOG Performance Status at Baseline

The ECOG Performance Status is a grade scale with scores running from 0 to 5, with 0 denoting perfect health and 5 denoting death. The ECOG score at baseline will be summarized descriptively as ordinal data and as frequency distribution.

4.5. BPI-SF at Baseline

The BPI-SF is self-administered questionnaire developed to assess the severity of pain and the impact of pain on daily functions[1]. The BPI-SF includes 4 items measuring the intensity of pain which make up the pain intensity subscale (scored as the mean of the 4 severity items), 7 items that assess how much pain has interfered with 7 daily activities which make up the pain interference subscale (scored as the mean of the seven interference items), and an additional item on the extent of pain relief. The BPI-SF also records whether the subject has had pain other than everyday kinds of pain today (yes/no). The baseline score of the 4 items measuring the intensity of pain, the pain intensity subscale, the pain interference subscale score and the extent of pain relief will be summarized descriptively. The baseline score of 4-items measuring the intensity of pain, the extent of pain relief and number of subjects with/without pain other than everyday kinds of pain at baseline will also be summarized as frequency distribution.

4.6. Clinical Laboratory Tests at Baseline

4.6.1. Hematology

For Hemoglobin, Hematocrit, Platelet count, Prothrombin Time (PT), Partial Thromboplastin Time (PTT), International Normalized Ratio (INR), Red blood cell (RBC) count, White blood cell (WBC) count, Neutrophils, Lymphocytes, Monocytes, Eosinophils and Basophils at baseline the number and percentage of subjects with abnormal values (High, Low) will be summarized as frequency distribution.

4.6.2. Chemistry

For all chemistry lab values at baseline, Albumin, ALP, AST, ALT, Calcium, LDH, Total Bilirubin, Total Protein, C-reactive Protein, K, Na, Urea & Creatinine, Homeostatic Model Assessments, Fasting Serum Lipids, Testosterone, Plasma ACTH and PSA, descriptive statistics will be provided and the number and percentage of subjects with Low, Normal, High values will be summarized as frequency distribution.

4.6.3. Urinalysis-Dipstick

For all Urinalysis-Dipstick lab values at baseline, i.e. Urine Glucose, Urine Protein, Urine Blood Sediment, RBC, WBC, Epithelial Cells, Crystals, Casts and Bacteria, descriptive statistics will be provided and the number and percentage of subjects with Low, Normal, High values will be summarized as frequency distribution.

4.7. Left Ventricular Ejection

Left Ventricular Ejection fraction is assessed at baseline. The number and percentage of subjects with a left ventricular ejection fraction ≥ 50% and the method used will be summarized as frequency distribution.

4.8. ECG Results

At baseline an ECG has been performed. The number and percentage of subject with an abnormal ECG as well as a clinically significant abnormality will be summarized as frequency distribution. For subjects with an abnormal ECG at baseline listing of Medical History will be provided.

4.9. Medical History

The number and percentage of subjects with any abnormality will be summarized overall and per body system. Specifications of abnormalities ongoing at study entry as well as overall will be summarized per body system.

4.10. Physical Examination

The number and percentage of subjects with any abnormality will be summarized overall and per body system. Specifications of abnormalities ongoing at study entry as well as overall will be summarized per body system.

4.11. Disposition Information

The number of subjects at each visit within the MSTP will be summarized as frequency distribution.

The number and percentage of subjects that completed the end of main study visit and for those subjects that that did not complete the reason will be summarized as frequency distribution. In case the subject did not complete the main study treatment period per protocol the primary reason for the subject's discontinuation of the main study treatment will be summarized as frequency distribution. In case study treatment was discontinued prematurely for the reason of a (serious) treatment emergent adverse event the type of (S)AEs will be summarized as frequency distribution, per body class. In case study treatment was discontinued for other reasons or because of ‘Withdrawal of consent’, the corresponding specifications will be summarized as frequency distribution.

The number of subjects that entered the extension phase will be summarized as frequency distribution.

4.12. Major Protocol Deviations

The number and percentage of subjects with a major protocol deviation identified during the study will be summarized as frequency distribution, overall and per PD category (i.e. Eligibility criteria not met, Prohibited concurrent medication, Dose modification/toxicity management not followed and Other).

4.13. Extent of Exposure

Descriptive statistics for the average dose and the mode dose (i.e. most frequently used dose) will be provided and for the start dose, the mode dose and the last dose used in the MSTP the frequency

distribution will be provided for treatment with abiraterone acetate. Frequency distribution as well as descriptive statistics will be provided for the number of months and number of cycles exposed with abiraterone acetate as well as steroids. The number of subjects that received the dose of steroids as prescribed by randomization (not taking into account missed doses) will be summarized as frequency distribution. The number and percentage of subjects with a dose reduction or a dose interruption for abiraterone acetate and for steroid dose as well as the reasons for the dose modifications will be summarized as frequency distribution.

4.14. Concomitant Medication

Medications started prior to first study treatment dose and continued into the treatment period will be considered concomitant medication used at baseline. Medication used at baseline or started during the MSTP will be considered as concomitant medication used during the MSTP. The number of subjects that used any concomitant medication at baseline, the number of subjects that used any concomitant medication during the MSTP and the number of subjects that started any concomitant medication during the MSTP will be summarized overall and per type of medication (generic name in the WHO drug dictionary) within ATC class. The most frequently used as well as started medications (>=5% of the subjects) will be summarized by frequency distribution.

In addition the use of potential antihypertensive medication will be analyzed. Any medication within the class 'Cardiovascular System' except for 'Lipid Modifying Agents' will be considered potential antihypertensive medication. The number of subjects that used any antihypertensive medication at baseline, the number of subjects that used any antihypertensive medication during the MSTP and the number of subjects that started any antihypertensive medication during the MSTP will be summarized overall and per type of medication.

For the type or classes of medications listed below the number of subjects that used any of these of medications at baseline, the number of subjects that used any of these medications during the MSTP and the number of subjects that started any of these medications during the MSTP will be summarized as frequency distribution:

- Potassium
- Lipid modifying agents
- Insulin
- Treatment for non-insulin-dependent diabetes mellitus
- Bone osteoprotective agent

5. EFFICACY

5.1. Analysis Specifications

All efficacy analyses described below will be performed on the efficacy analysis set and data will be summarized overall and per treatment arm, for all scheduled visits during the MSTP, unless mentioned differently, including the main study treatment endpoint visit.

For continuous and ordinal data descriptive statistics (mean, standard deviation, median, range and 95% CI) will be provided.

Categorical data will be summarized as frequency distribution and for dichotomous data 95% CI will be added, where applicable. Shifts within treatment arms will be tested using the Wilcoxon Signed-Rank test.

Time-to-event endpoints will be analyzed using Kaplan-Meier product limit methods to estimate the survival distributions and the median time-to-event. Results will be summarized by 25th, 50th (median) and 75th percentiles with associated 2-sided 95% CIs, range and mean (SE), as well as percent of subjects with an event/censored by treatment arm and overall. A graphical presentation of the time-to-event curve will also be provided.

5.1.1. Level of Significance

All statistical tests will be interpreted at the 5% significance level (2-tailed), unless otherwise specified.

5.1.2. General Imputation Methods

Last observation carried forward (LOCF) approach will be used for the endpoint analysis. Last observation is defined as the last visit with non-missing data for the parameter analyzed. See also section 2.1.1.

5.2. Efficacy Endpoint(s)

5.2.1. PSA Response Rate

5.2.1.1. Definition

Blood samples are drawn to assess the PSA levels at baseline, cycle 2 and each visit afterwards. Based on these PSA levels two types of PSA response are defined: One as a ≥50% decline from baseline and one as a ≥90% decline from baseline. For a PSA response to be confirmed, an additional PSA measurement obtained 4 or more weeks later has to show ≥50% or ≥90% decline from baseline. Time to PSA Progression (by PCWG2 criteria) is defined as the time interval from the date of randomization to the date of the first PSA progression as defined in the PCWG2 criteria during the MSTP.

5.2.1.2. Analysis Methods

The number and percentage of subjects with PSA Response (≥50% decline) and the number and percentage of subjects with a PSA Response (≥90% decline) both confirmed and unconfirmed at each cycle will be summarized as frequency distribution and corresponding 95% CI will be added. The number of subjects with PSA response (decrease from baseline >= 50% anytime during the MSTP) and the number of subjects with PSA progression (based on the PCWG2 criteria) during the MSTP will be summarized as frequency distribution and corresponding 95% CI will be added. A waterfall plot will be provided for the maximum percent change of PSA during the MSTP compared to baseline.

Time to PSA Progression will be analyzed using Kaplan-Meier product-limit methods. Subjects who have no PSA progression at end of the MSTP will be censored at the last known date of no progression

in the MSTP. Subjects with no on-study assessment or no baseline assessment will be censored at date of randomization.

5.2.2. Eastern Cooperative Oncology Group (ECOG) Performance Status

5.2.2.1. Definition

The ECOG Performance Status is a grade scale with scores running from 0 to 5, with 0 denoting perfect health and 5 denoting death. The ECOG is assessed at each scheduled visit during the MSTP. The time to deterioration in ECOG performance score by 1 point is defined as the time interval from the date of randomization to the first date in which at least one point change (worsening) in the ECOG is observed. Confirmation of worsening of status is required.

5.2.2.2. Analysis Methods

Changes from baseline for ECOG scores will be calculated for each subject at every post-baseline scheduled visit. Descriptive statistics will be provided for the ECOG score and the corresponding changes, at each visit. The changes from baseline at all visits will be tested with the Wilcoxon-signed-rank test, and the associated p-values will be presented.

Time to deterioration in ECOG performance score by 1 point will be analyzed using Kaplan-Meier product-limit methods. Subjects who have no deterioration in ECOG during the MSTP will be censored at the last known date of no deterioration. Patients with no on-study assessment or no baseline assessment will be censored at date of randomization.

5.2.2.3. Imputation Method

No imputation for missing items will take place.

5.2.3. Evaluation of response

5.2.3.1. Definition

Target Lesion(s) Response, Non-Target Lesion(s) Response, Overall response for soft tissue and Appearance of any new lesions (Yes/No) is assessed at cycle 3, cycle 6, cycle 12, cycle 18, cycle 21 and every 3 cycles afterwards until cycle 36 and EOMT. Both Target Lesions Response and non-Target Lesion response are assessed as Complete Response (CR), Partial Response (PR), Stable Disease (SD), Progressive Disease (PD), Not Applicable (NA) or Not Evaluable (NE). Overall Response for soft tissue is assessed as CR, PR, SD, PD, Non-CR/Non-PD or NE. Objective response rate is defined as the proportion of subjects with measurable disease achieving a complete or partial response according to modified RECIST criteria.

5.2.3.2. Analysis Methods

Target lesion(s) response, non-Target Lesion(s) Response, Overall Response as well as Objective Response will be summarized as frequency distribution at each assessment visit. Appearance of any new lesion during the MSTP will be summarized as frequency distribution at each assessment visit. For subjects with new lesions listings will be provided reporting the anatomic site and the location of the lesion.

5.2.4. Best response

5.2.4.1. Definition

Best response to study therapy is assessed at end of the MSTP or end of extension (EOMS or EOE). For subjects entering the extension phase best response during the MSTP should be derived from the overall response at each visit during the MSTP using modified RECIST criteria.

5.2.4.2. Analysis method

Best response during the MSTP will be summarized as frequency distribution.

5.2.5. Duration of response in Subjects with Measurable Disease

5.2.5.1. Definition

Duration of response is defined as the time interval from the first date of a response (CR/PR, which was later confirmed) in subjects with measurable disease at baseline, according to modified RECIST criteria, to the date of progression or death due to PD.

5.2.5.2. Analysis method

Duration of response will be analyzed using Kaplan-Meier product-limit methods. Subjects who have not progressed or died due to PD at the end of the MSTP will be censored on the last date of assessment.

5.2.6. New bone lesions on bone scan confirmed by second bone scan

5.2.6.1. Definition

The number of new bone lesions compared to baseline is assessed at cycle 3, cycle 6 and all subsequent visits of the MSTP as 0, 1, 2 or more new lesions. A patient is considered to have progressed by bone scan if

- the first bone scan with ≥2 new lesions compared to baseline is observed <12 weeks from randomization and is confirmed by a second bone scan taken ≥6 weeks later showing ≥2 additional new lesions (a total of ≥4 new lesions compared to baseline) or
- the first bone scan with ≥2 new lesions compared to baseline is observed ≥12 weeks from randomization and the new lesions are verified on the next bone scan ≥6 weeks later (a total of ≥2 new lesions compared to baseline).

5.2.6.2. Analysis method

The number of new bone lesions at each visit and the number of subjects that progressed by bone scan during the MSTP will be summarized as frequency distribution.

5.2.7. Progression-Free Survival

5.2.7.1. Definition

Progression-free survival (PFS) is defined as the time from randomization to the occurrence of one of the following: radiographic progression, clinical progression or death. Radiographic Progression-Free

Survival (rPFS) is defined as the time from randomization to the occurrence of either radiographic progression or death.

5.2.7.2. Analysis method

Both PFS and rPFS will be analyzed using Kaplan-Meier product-limit methods. Subjects who did not progress during and who are still alive at end of the MSTP will be considered censored at the date of the last assessment of no disease progression.

5.2.8. Skeletal related events

5.2.8.1. Definition

Time to first skeletal event is defined as the time from randomization to the date of the first occurrence of a skeletal-related event.

5.2.8.2. Analysis method

Time till first skeletal event will be analyzed using Kaplan-Meier product-limit methods. Subjects who did not experience a skeletal-related event during the MSTP will be considered censored at the time of the discontinuation or at the time of completion of the MSTP.

5.2.9. Overall survival

5.2.9.1. Definition

Survival data is collected throughout the MSTP and during extension and follow-up. Overall survival time (OS) defined as the time from randomization to the date of death (regardless of the cause) within the MSTP will be analyzed for the MSTP but will be updated once the entire study (including extension and follow-up) is ended.

5.2.9.2. Analysis method

OS will be analyzed using Kaplan-Meier product-limit methods. Subjects who are still alive at the end of the MSTP will be considered censored at the time of the discontinuation or at the time of completion of the MSTP.

5.2.10. Time to Opiate Use for Cancer-related Pain

5.2.10.1. Definition

Time to opiate use for cancer-related pain is defined as the time interval from the date of randomization to the first date of opiate use.

5.2.10.2. Analysis method

Time to opiate use for cancer-related pain will be analyzed using Kaplan-Meier product-limit methods. Subjects who have not used opiates for cancer-related pain during the study will be censored at the end of the MSTP. Subjects with no on-study assessment or no baseline assessment will be censored at date of randomization.

5.2.11. Patient-Reported Outcomes

5.2.11.1. Brief Pain Inventory – Short form (BPI-SF)

5.2.11.1.1. Definition

The BPI-SF is a self-administered questionnaire developed to assess the severity of pain and the impact of pain on daily functions[1]. The BPI-SF includes 4 items measuring the intensity of pain at its "worst," "least," "average," and "now" (current pain) These 4 items are scored as 0=no pain and 10=pain as bad as you can imagine, which make up the pain intensity subscale. It also assesses 7 items that assess how much pain has interfered with 7 daily activities, including general activity, walking, work, mood, enjoyment of life, relations with others, and sleep. These 7 items are score as 0=no interference and 10=completely interferes, which make up the pain interference subscale. Additionally the extent of pain relief with medication received and whether the subject has had pain other than everyday kinds of pain today (yes/no) is assessed. During the MSTP, BPI-SF is assessed at baseline, at visit cycle 6, visit cycle 18 and EOMT.

Based on the BPI-SF assessment the following time-to-event parameters will be calculated:

Time to Average Pain Intensity Progression: defined as the time interval from randomization to the first date a subject experiences an increase of ≥30% from baseline in the average of BPI-SF pain intensity item scores (items 3, 4, 5, and 6) observed at 2 consecutive evaluations ≥4 weeks apart without a decrease in analgesic usage score.

Time to Average Pain Intensity Progression Using 2-Point Increase Threshold: is defined as the time interval from randomization to the first date a subject experiences an increase by 2 points from baseline in the BPI-SF average pain intensity (average of BPI-SF items 3, 4, 5, and 6) observed at 2 consecutive evaluations ≥4 weeks apart without decrease in analgesic usage score.

Time to Worst Pain Intensity Progression: is defined as the time interval from randomization to the first date a subject experiences an increase by ≥30% from baseline in the BPI-SF worst pain intensity item (item 3) observed at 2 consecutive evaluations ≥4 weeks apart without decrease in analgesic usage score.

Time to Worst Pain Intensity Progression Using 2-Point Increase Threshold: was defined as the time interval from randomization to the first date a subject experiences an increase by 2 points from baseline in the BPI-SF worst pain intensity item (item 3) observed at 2 consecutive evaluations ≥4 weeks apart without a decrease in analgesic usage score.

Time to Pain Interference Progression: is defined as the time interval from randomization to the first date a subject experiences an increase of one half the standard deviation of the baseline BPI-SF pain interference scale.

5.2.11.1.2. Analysis Methods

The pain intensity subscale is calculated as the mean of the 4 items measuring the pain intensity. The pain interference subscale is calculated as the mean of the 7 items measuring the pain interference, only if at least four of the seven items have been completed. Changes from baseline to each visit of the scores

of the 4 items measuring pain intensity, the pain intensity subscale and the pain interference subscale will be calculated. Both the scores as well as the changes of the 4 items and the 2 subscales will be summarized descriptively. The changes from baseline at each assessment visits (including main study treatment endpoint) will be tested with the Wilcoxon-signed-rank test, and the associated p-values will be presented. The extent of pain relief with medication received (0%, 10%, …, 100%) and whether the subject has had pain other than everyday kinds of pain today (yes/no) will be summarized as frequency distribution.

The following time-to-event parameters will be analyzed using Kaplan-Meier product-limit methods:

Time to Average Pain Intensity Progression
Time to Average Pain Intensity Progression Using 2-Point Increase Threshold
Time to Worst Pain Intensity Progression
Time to Worst Pain Intensity Progression Using 2-Point Increase Threshold
Time to Pain Interference Progression

Subjects who have not experienced pain intensity progression or pain interference progression during the MSTP will be censored on the last known date the subject is known to have not progressed. Subjects with no on-study assessment or no baseline assessment will be censored at date of randomization.

5.2.11.1.3. Imputation Method

No imputation for missing items will take place.

5.2.11.2. EQ-5D-5L

5.2.11.2.1. Definition

During the MSTP the EQ-5D-5L is a frequently used generic instrument to measure health-related QoL[2]. It comprises 5 dimensions, i.e. mobility, self-care, usual activities, pain/discomfort and anxiety/depression. Each dimension has 5 levels: 1=no problems, 2=slight problems, 3=moderate problems, 4=severe problems and 5=extreme problems.

The EQ-5D-5L VAS records the respondent's self-rated health on a 20 cm vertical, visual analogue scale with endpoints 100 labelled 'the best health you can imagine' and 0 labelled 'the worst health you can imagine.

With the levels of the 5 dimensions the EQ-5D-5L index value is calculated.

The EQ-5D-5L is assessed at baseline, at cycle 6, cycle 18 and EOMT.

5.2.11.2.2. Analysis Methods

The reported levels of each of the 5 dimensions will be summarized as frequency distribution for each assessment visit. Changes from baseline at each assessment visits (including main study treatment endpoint) will be calculated for the EQ VAS and the EQ-5D-5L index score. Descriptive statistics will be provided for the reported level of each of the 5 dimensions and for the EQ VAS as well as for the

corresponding changes, at each assessment visit. The changes from baseline at all visits will be tested with the Wilcoxon-signed-rank test, and the associated p-values will be presented.

5.2.11.2.3. Imputation Method

No imputation for missing items will take place.

5.2.11.3. Functional Assessment of Cancer Therapy – Prostate (FACT-P)

5.2.11.3.1. Definition

The FACT-P is a multidimensional, self-report QoL instrument specifically designed for use with prostate cancer subjects[3]. It consists of 27 core items which assess subject function in four domains: Physical, Social/Family, Emotional, and Functional well-being, which is further supplemented by 12 site specific items to assess for prostate related symptoms. Each item is rated on a 0 to 4 Likert type scale (0=Not at all,…, 4=Very much) and then combined to produce subscale scores for each domain, as well as a global QoL score. Higher scores represent better QoL.

During the MSTP the FACT-P is assessed at baseline, cycle 6, cycle 18 and EOMT.

Based on the FACT-P assessments the following time-to-event parameters will be calculated:

Time to degradation in the FACT-P (Total Score)
Time to degradation in the FACT-P Prostate Cancer Scale (PCS)
Time to degradation in the FACT-P (PWB)
Time to degradation in the FACT-P (SFWB)
Time to degradation in the FACT-P (EWB)
Time to degradation in the FACT-P (FWB)

All parameters are defined as the time interval from randomization to the first time degradation in the specific FACT-P scale is assessed.

5.2.11.3.2. Analysis Methods

The score of the each of the 4 domains, the Prostate Cancer-specific subscale and the total FACT-P are calculated. Changes from baseline at each assessment visits (including main study treatment endpoint) will be calculated. Descriptive statistics will be provided for the reported level of each of the 4 domains, the Prostate Cancer-specific subscale and the Total FACT-P as well as for the corresponding changes, at each assessment visit. The changes from baseline at all visits will be tested with the Wilcoxon-signed-rank test, and the associated p-values will be presented.

The time-to-event parameters mentioned in section 5.2.11.3.1 will be analyzed using Kaplan-Meier product-limit methods. Subjects who have not experienced any degradation in the specific FACT-P scale during the MSTP will be censored on the last assessment date during the MSTP. Subjects with no on-study assessment or no baseline assessment will be censored at date of randomization.

5.2.11.3.3. Imputation Method

Imputation methods are described in FACT-P Ver4 scoring[3].

6. SAFETY

All safety analysis described below will be performed on the safety analysis set and data will be summarized overall and per treatment arm. For continuous and ordinal data descriptive statistics (mean, standard deviation, median, range and 95% CI) will be provided. Categorical data will be summarized as frequency distribution.

6.1. Adverse Events

All adverse events (whether serious or non-serious, related or not related) following exposure to study treatment are recorded in the CRF. The verbatim terms used in the eCRF by investigators to identify AEs will be coded to System Organ Class (SOC) and preferred terms using the MedDRA® coding system (version 15.1 or higher). All reported AEs that started during the MSTP, which will include pre-existing medical conditions that have worsened since baseline, will be included in the analysis. All AEs are collected through 30 days post last dose. AEs with an onset date during treatment and AEs that have worsened since baseline will be defined as Treatment Emergent AEs (TEAEs).

All incidences of hypokalemia (NCI CTCAE grade ≥1) and of hypertension (NCI CTCAE grade ≥2) will be reported as AEs.

The severity of AEs will be graded on a scale of 1 to 5 according to the adult NCI Common Terminology Criteria for Adverse Events (CTCAE version 4.0) where higher grades indicate events of higher severity.

All AEs will have their relationship to study drug assessed by the investigator as not related, Doubtful, Possible, Probable or Very likely related. Adverse events will be categorized and summarized according to their highest relationship to study drug. Adverse events reported as Possible, Probable or Very likely related will be classified as causally related AEs.

6.1.1. Primary endpoint

The primary parameter is defined as experiencing (yes or no) neither of the 2 mineralocorticoid excess toxicities during the first 24 weeks of treatment, i.e. neither hypokalemia nor hypertension. This will be derived from treatment-emergent AE data (see SMQs in Attachment 1), which will be defined using MedDRA and graded according to the NCI CTCAE v4.0. Subjects discontinuing the study prior to week 24 of the MSTP without experiencing one of the 2 mineralocorticoid excess toxicities will not be included in the analysis of the primary parameter.

The number and percentage of subjects experiencing neither of the 2 mineralocorticoid excess toxicities during the first 24 weeks of treatment will be summarized as frequency distribution per treatment arm and the corresponding 95% CI will be displayed.

6.1.2. Secondary endpoint

For summary purposes, TEAEs will be defined as all reported events with a start date on study treatment or that increase in severity on during study treatment. The following summaries of TEAEs will be provided as incidence tables by treatment group and overall:

- the number and percentage of subject with at least one TEAE, serious TEAE (TESAE), causally related (i.e. possibly, probably or very likely related) TEAEs, Grade 3-4 TEAE, TEAE leading to Treatment discontinuation, TEAE leading to death
- frequency distribution of TEAEs (including and excluding TESAEs) and TESAEs, per body class
- most frequently mentioned (≥5%) TEAEs (including and excluding TESAEs) and TESAEs
- frequency distribution of TEAEs/TESAEs with grade 3 or 4 according to the worst grade experienced, per body class
- frequency distribution of TEAEs/TESAEs leading to treatment discontinuation, per body class
- frequency distribution of TEAEs/TESAEs leading to death, per body class
- frequency distribution of severity, relationship to study treatment, action taken regarding study treatment, concomitant therapy started and outcome of all reported TEAEs
- listing of characteristics and treatment information for subjects who died during the study
- listing of TEAEs for which subjects discontinued the MSTP
- listing of AEs started after signing informed consent but before initiation of study treatment
- listing of AEs that started within 30 days after last dose.

The number and percentage of subjects experiencing neither causally related hypertension nor causally related hypokalemia during the first 24 weeks of treatment will be summarized as frequency distribution per treatment arm and the corresponding 95% CI will be displayed.

The primary parameter will also be analyzed for subjects with and for subjects without pre-existing hypertension (according to Medical history) separately.

Adverse events of special interest, including fluid retention/edema, hypokalemia, hypertension, cardiac disorders and hepatotoxicity, were identified based on previous experience with abiraterone acetate. Other AEs of special interest are fatigue, hyperglycemia, infection, oesteoporosis and weight gain. Preferred terms were grouped according to Standardized MedDRA Queries (SMQ) that are used across abiraterone acetate studies (see attachment 1 'List of Preferred Terms for SMQs'). The following summaries of TEAEs of special interest will be provided as incidence tables by treatment group and overall.

- frequency distribution of TEAEs of special interest
- frequency distribution of TEAEs of special interest by grade
- actual time between the start of study treatment and the start of the hypertension as well as start of hypokalemia (days) will be summarized descriptively
- Graphical representation of the percent incidence of TEAEs by exposure for all-grade and for grade ≥ 3

- Graphical representation of the cumulative incidence rate of AEs (all grades) from time of first incidence of AEs

6.2. Clinical Laboratory Tests

6.2.1. Plasma ACTH

During the MSTP, plasma ACTH is assessed at baseline and cycle 3. Absolute changes from baseline will be calculated at cycle 3. Descriptive statistics will be provided for the observed values as well as for the absolute changes. Changes from baseline at cycle 3 will be tested with the Wilcoxon-signed-rank test, and the associated p-values will be presented. In addition the number of subjects who experience low, normal or high values at baseline and at cycle 3, as well across will be summarized as frequency distribution.

6.2.2. Serum androgens

During the MSTP, serum androgens such as DHEA, dihydrotestosterone, testosterone, DHEA-S and androstenedione are collected at baseline, cycle 2, cycle 3, cycle 6, cycle 12, cycle 18, cycle 24, cycle 30, cycle 36 and EOMT. Absolute changes from baseline will be calculated at each assessment visit. Descriptive statistics will be provided for the observed values as well as for the absolute changes of these tests at each assessment visit. The changes from baseline at all visits will be tested with the Wilcoxon-signed-rank test, and the associated p-values will be presented. In addition the number of subjects who experience low, normal or high values at each visit and the shifts from baseline at each post-baseline (i.e. from low-normal-high at baseline to low-normal-high at post-baseline visits) will summarized as frequency distribution.

6.2.3. Androgen precursors profile

During the MSTP, 24-hour urine assessments such as DHEA, DHEA-S, DHT, androstenedione and testosterone are assessed at baseline, cycle 3, cycle 6, cycle 12, cycle 18, cycle 24, cycle 30, cycle 36 and EOMT. Absolute changes from baseline will be calculated at each assessment visit. Descriptive statistics will be provided for the observed values as well as for the absolute changes of all the 24-hour urine assessments of mineralocorticoids and their precursor steroids at each assessment visit. The changes from baseline at all visits will be tested with the Wilcoxon-signed-rank test, and the associated p-values will be presented. In addition the number of subjects who experience low, normal or high values at each visit and the shifts from baseline at each post-baseline (i.e. from low-normal-high at baseline to low-normal-high at post-baseline visits) will summarized as frequency distribution.

Apart from the analysis of all the urinary steroids assessed, the following sum of steroids will be calculated and analyzed:

- Active androgens: Androsterone + etiocholanolone
- Androgen precursors: DHEA + 5-PD + 5-PT
- 17-hydroxyprogesterone (17OHP) Metabolites: 17HP + PT + PTONE
- Deoxycorticosterone (DOC) metabolites: THDOC + 5αTHDOC

- Corticosterone (B) metabolites: THB + 5αTHB + THA + 5αTHA
- Total glucocorticoid metabolites: THF+5αTHF + THE + α-cortol + β-cortol + α-cortolone + β-cortolone + cortisol + cortisone

Descriptive statistics will be provided for the observed values as well as for the absolute changes of these sums of steroids at each assessment visit. The changes from baseline at all visits will be tested with the Wilcoxon-signed-rank test, and the associated p-values will be presented.

6.2.4. Chemistry Tests

During the MSTP, chemistry tests such as Albumin, alkaline phosphatase, ALT (SGPT), AST (SGOT), Calcium, LDH, total protein, Total Bilirubin and C-reactive Protein are assessed at baseline, cycle 2, cycle 3, cycle 6, cycle 12, cycle 18 and EOMT. Other chemistry tests such as K, Na, Urea & Creatinine are assessed at all visits. Absolute changes from baseline will be calculated at each assessment visit. Descriptive statistics will be provided for the observed values as well as for the absolute changes of these tests at each assessment visit. The changes from baseline at all visits will be tested with the Wilcoxon-signed-rank test, and the associated p-values will be presented. In addition the number of subjects who experience low, normal or high values at each visit and the shifts from baseline at each post-baseline (i.e. from low-normal-high at baseline to low-normal-high at post-baseline visits) will summarized as frequency distribution. For all tests (except LDH, total protein, C-reactive Protein and urea) baseline toxicity grade will also be cross tabulated with worst on-treatment toxicity grade (NCI CTC AE version 4.0).

6.2.5. Insulin resistance

During the MSTP, fasting plasma glucose, fasting serum insulin and HbA1c are assessed at baseline, cycle 2, cycle 3, cycle 6, cycle 12, cycle 18 and EOMT.

HOMA-IR will be calculated using the following formula:

Fasting plasma glucose (mmol/L) * fasting serum insulin (mU/L) / 22.5

For all 4 tests, absolute changes as well as percent change from baseline will be calculated at each assessment visit. Descriptive statistics will be provided for the observed values as well as for the absolute changes of these 4 tests at each assessment visit. Changes from baseline at all visits will be tested with the Wilcoxon-signed-rank test, and the associated p-values will be presented. For fasting plasma glucose, fasting serum insulin and HbA1c the number of subjects who experience low, normal or high values at each visit and the shifts from baseline at each post-baseline (i.e. from low-normal-high at baseline to low-normal-high at post-baseline visits) will summarized as frequency distribution.

6.2.6. Fasting serum lipids

Fasting serum lipids include cholesterol, triglycerides, high-density lipoprotein and low-density lipoprotein and are assessed at baseline, cycle 2, cycle 3, cycle 6, cycle 12, cycle 18 and EOMT. For all these tests absolute changes from baseline will be calculated at each assessment visit. Descriptive statistics will be provided for the observed values as well as for the absolute changes of these tests at each assessment visit. Changes from baseline at all visits will be tested with the Wilcoxon-signed-rank

test and the associated p-values will be presented. In addition the number of subjects who experience low, normal or high values at each visit and the shifts from baseline at each post-baseline (i.e. from low-normal-high at baseline to low-normal-high at post-baseline visits) will summarized as frequency distribution.

6.3. DXA scans

During the MSTP DXA scan results and DXA scan fluid retention assessments where performed at baseline, cycle 3, cycle 6, cycle 12 and EOMT.

For BMD-Total Body, BMD-Left Hip, BMD-Lumbar Spine, BMD Arms, BMD legs, BMC-Total Body, Area Total Body, Region (% fat), Fat (g) and Lean Mass (g) for all regions (i.e. Total Body, Left arm, Right arm, Left leg, Right leg and Trunk) percent change from baseline as well as since previous visit will be calculated. Descriptive statistics will be provided for the baseline value and for the percent change of these parameters at each assessment visit. Changes at all visits will be tested with the Wilcoxon-signed-rank test and the associated p-values will be presented.

T-scores and Z-scores of Left Hip, Lumbar Spine and Total Body are assessed using the following scores: 'Less than -2.5 with presence of fracture (osteoporosis)', 'Between -1 and -2.5 (osteopenia)' and 'Higher than -1 (normal)' and will be summarized as frequency distribution at each visit assessed. Changes from baseline within these classes at each post-baseline visit will be summarized as frequency distribution.

For Waist circumference and Mid-thigh circumference percent changes from baseline will be calculated. Descriptive statistics will be provided for the observed values as well as for the percent changes of these parameters at each post-baseline visit. Information on whether there is evidence of fluid retention will be summarized as frequency distribution at each visit assessed.

6.4. Body weight

The body weight measurements are recorded at every scheduled visit during the MSTP. BMI at every scheduled visit will be calculated using the formula weight (kg)/(Height at baseline (m))^2. The change from baseline in weight and BMI will be calculated for each post-baseline scheduled visit. Descriptive statistics will be provided for the observed values and for the changes versus baseline in weigh and BMI at each assessment visit. The changes from baseline will be tested with the Wilcoxon-signed-rank test, and the associated p-values will be presented.

The number and percentage of subjects with an increase in body weight >= 5% at any assessment during the MSTP compared to baseline will be summarized as frequency distribution.

6.5. Vital signs

Vital sign measurements (i.e. blood pressure, heart rate, respirations, and body temperature) are recorded at every scheduled visit during the MSTP. Vital signs and change from baseline in vital signs will be summarized by descriptive statistics at each assessment visit.

The number of subjects with hypertension stage 1 (i.e. $140 \leq SBP < 160$ or $90 \leq DBP < 100$) or stage 2 and above (i.e. $SBP \geq 160$ or $DBP \geq 100$) at baseline will be calculated and will be cross-tabulated with the number of subjects with worst hypertension stage 1 or stage 2 and above during the MSTP.

6.6. Biomarkers

The biomarker analysis will be performed by the Janssen biomarker team. The corresponding SAP will be provided by this team and will therefore be a separate document, not included in this SAP.

6.7. Medical Resource Utilization

Whether a subject required any medical resource of any kind other those mandated in the study is assessed from the first dose of main study treatment until the last dose of main study treatment.

The following parameters assessed during the MSTP will be calculated as a 4-weekly rate and summarized descriptively:

- number and duration of overnight hospitalizations
- if Intensive Care Unit (ICU) was involved the number and length of ICU stays
- number of hospital day care ward (without overnight) visits
- number of emergency room visits
- number of oncologist visits
- number of urologist visits
- number of practitioner visits
- number of home care nurse visits
- number of study investigator visits
- number of other visits
- number of radiotherapy tests, surgical therapy tests, MRI tests, CT tests, X-Ray tests, Other tests/procedures

REFERENCES

1. BPI-SF scale:

BPI-SF.pdf BPI_UserGuide.pdf

2. EQ-5D-5L scale:

EQ-5D-5L UserGuide_v2.0_Oct(

3. FACT-P scale:

FACT-P_ENG_Final_Ver4_19Nov07.pdf FACT-P_Ver4_scoring.pdf

ATTACHMENTS

1. LIST OF PREFERRED TERMS FOR SMQS

**Hypertension (SMQ)**	
Accelerated hypertension	Aldosterone urine abnormal
Aldosterone urine increased	Angiotensin converting enzyme increased
Angiotensin I increased	Angiotensin II increased
Blood aldosterone abnormal	Blood aldosterone increased
Blood catecholamines abnormal	Blood catecholamines increased
Blood pressure abnormal	Blood pressure ambulatory abnormal
Blood pressure ambulatory increased	Blood pressure diastolic abnormal
Blood pressure diastolic increased	Blood pressure fluctuation
Blood pressure inadequately controlled	Blood pressure increased
Blood pressure management	Blood pressure orthostatic abnormal
Blood pressure orthostatic increased	Blood pressure systolic abnormal
Blood pressure systolic increased	Catecholamines urine abnormal
Catecholamines urine increased	Diastolic hypertension
Diuretic therapy	Eclampsia
Ectopic aldosterone secretion	Ectopic renin secretion
Endocrine hypertension	Epinephrine abnormal
Epinephrine increased	Essential hypertension
Gestational hypertension	Hellp syndrome
Hyperaldosteronism	Hypertension
Hypertension neonatal	Hypertensive angiopathy
Hypertensive cardiomegaly	Hypertensive cardiomyopathy
Hypertensive crisis	Hypertensive emergency
Hypertensive encephalopathy	Hypertensive heart disease
Hypertensive nephropathy	Labile blood pressure
Labile hypertension	Malignant hypertension
Malignant hypertensive heart disease	Malignant renal hypertension
Maternal hypertension affecting foetus	Mean arterial pressure increased
Metabolic syndrome	Metanephrine urine abnormal
Metanephrine urine increased	Neurogenic hypertension
Non-dipping	Norepinephrine abnormal
Norepinephrine increased	Normetanephrine urine increased
Orthostatic hypertension	Pre-eclampsia
Prehypertension	Procedural hypertension
Pseudoaldosteronism	Renal hypertension
Renal sympathetic nerve ablation	Renin abnormal
Renin increased	Renin-angiotensin system inhibition
Renovascular hypertension	Retinopathy hypertensive
Secondary aldosteronism	Secondary hypertension
Systolic hypertension	Tyramine reaction
Withdrawal hypertension	

Hypokalemia events
Blood potassium abnormal
Blood potassium
Hypokalaemia
Blood potassium decreased

Haemodynamic oedema, effusion and fluid overload (SMQ)	
Acute pulmonary oedema	Administration site oedema
Administration site swelling	Amyloid related imaging abnormalities
Application site oedema	Application site swelling
Ascites	Bone marrow oedema
Bone marrow oedema syndrome	Bone swelling
Brain oedema	Bronchial oedema
Capillary leak syndrome	Catheter site oedema
Cerebral oedema management	Cervix oedema
Compression stockings application	Cytotoxic oedema
Effusion	Elephantiasis nostras verrucosa
Extensive swelling of vaccinated limb	Fluid overload
Fluid retention	Gallbladder oedema
Gastrointestinal oedema	Generalised oedema
Gestational oedema	Gravitational oedema
Heat oedema	Hydraemia
Hydrothorax	Hypervolaemia
Hypoosmolar state	Implant site oedema
Implant site swelling	Incision site oedema
Incision site swelling	Infusion site oedema
Infusion site swelling	Injection site joint swelling
Injection site oedema	Injection site swelling
Instillation site oedema	Joint effusion
Joint swelling	Lipoedema
Local swelling	Localised oedema
Lymphoedema	Mouth swelling
Muscle oedema	Muscle swelling
Myocardial oedema	Non-cardiogenic pulmonary oedema
Oedema	Oedema due to cardiac disease
Oedema due to hepatic disease	Oedema due to renal disease
Oedema mucosal	Oedema neonatal
Oedema peripheral	Oedematous kidney
Oesophageal oedema	Pelvic fluid collection
Pericardial effusion	Perinephric effusion
Peripheral oedema neonatal	Peripheral swelling
Pleural effusion	Prevertebral soft tissue swelling of cervical space
Pulmonary oedema	Pulmonary oedema neonatal

**Haemodynamic oedema, effusion and fluid overload (SMQ)**	
Puncture site oedema	Reexpansion pulmonary oedema
Retroperitoneal effusion	Retroperitoneal oedema
Scleroedema	Skin oedema
Skin swelling	Small bowel angioedema
Spinal cord oedema	Subdural effusion
Swelling	Testicular swelling
Vasogenic cerebral oedema	Visceral oedema

Cardiac disorder events	Sub category
Acute coronary syndrome	Ischaemic heart disease (SMQ)
Acute myocardial infarction	Ischaemic heart disease (SMQ)
Angina pectoris	Ischaemic heart disease (SMQ)
Angina unstable	Ischaemic heart disease (SMQ)
Angina unstable	Ischaemic heart disease (SMQ)
Arteriogram coronary abnormal	Ischaemic heart disease (SMQ)
Arteriosclerosis coronary artery	Ischaemic heart disease (SMQ)
Arteriospasm coronary	Ischaemic heart disease (SMQ)
Blood creatine phosphokinase abnormal	Ischaemic heart disease (SMQ)
Blood creatine phosphokinase increased	Ischaemic heart disease (SMQ)
Blood creatine phosphokinase mb abnormal	Ischaemic heart disease (SMQ)
Blood creatine phosphokinase mb increased	Ischaemic heart disease (SMQ)
Cardiac stress test abnormal	Ischaemic heart disease (SMQ)
Cardiopulmonary exercise test abnormal	Ischaemic heart disease (SMQ)
Computerised tomogram coronary artery abnormal	Ischaemic heart disease (SMQ)
Coronary angioplasty	Ischaemic heart disease (SMQ)
Coronary arterial stent insertion	Ischaemic heart disease (SMQ)
Coronary artery bypass	Ischaemic heart disease (SMQ)
Coronary artery disease	Ischaemic heart disease (SMQ)
Coronary artery dissection	Ischaemic heart disease (SMQ)
Coronary artery embolism	Ischaemic heart disease (SMQ)
Coronary artery insufficiency	Ischaemic heart disease (SMQ)
Coronary artery occlusion	Ischaemic heart disease (SMQ)
Coronary artery reocclusion	Ischaemic heart disease (SMQ)
Coronary artery restenosis	Ischaemic heart disease (SMQ)
Coronary artery stenosis	Ischaemic heart disease (SMQ)
Coronary artery thrombosis	Ischaemic heart disease (SMQ)
Coronary bypass thrombosis	Ischaemic heart disease (SMQ)
Coronary endarterectomy	Ischaemic heart disease (SMQ)
Coronary no-reflow phenomenon	Ischaemic heart disease (SMQ)
Coronary ostial stenosis	Ischaemic heart disease (SMQ)
Coronary revascularisation	Ischaemic heart disease (SMQ)
Dissecting coronary artery aneurysm	Ischaemic heart disease (SMQ)
Ecg electrically inactive area	Ischaemic heart disease (SMQ)
Ecg signs of myocardial infarction	Ischaemic heart disease (SMQ)
Ecg signs of myocardial ischaemia	Ischaemic heart disease (SMQ)
Electrocardiogram q wave abnormal	Ischaemic heart disease (SMQ)
Electrocardiogram st segment abnormal	Ischaemic heart disease (SMQ)
Electrocardiogram st segment depression	Ischaemic heart disease (SMQ)
Electrocardiogram st segment elevation	Ischaemic heart disease (SMQ)
Electrocardiogram st-t segment abnormal	Ischaemic heart disease (SMQ)
Electrocardiogram st-t segment depression	Ischaemic heart disease (SMQ)
Electrocardiogram st-t segment elevation	Ischaemic heart disease (SMQ)
Electrocardiogram t wave abnormal	Ischaemic heart disease (SMQ)
Electrocardiogram t wave inversion	Ischaemic heart disease (SMQ)
Exercise electrocardiogram abnormal	Ischaemic heart disease (SMQ)

Cardiac disorder events	Sub category
Exercise test abnormal	Ischaemic heart disease (SMQ)
External counterpulsation	Ischaemic heart disease (SMQ)
Haemorrhage coronary artery	Ischaemic heart disease (SMQ)
Infarction	Ischaemic heart disease (SMQ)
Ischaemic cardiomyopathy	Ischaemic heart disease (SMQ)
Kounis syndrome	Ischaemic heart disease (SMQ)
Microvascular coronary artery disease	Ischaemic heart disease (SMQ)
Myocardial infarction	Ischaemic heart disease (SMQ)
Myocardial ischaemia	Ischaemic heart disease (SMQ)
Myocardial necrosis	Ischaemic heart disease (SMQ)
Myocardial necrosis marker increased	Ischaemic heart disease (SMQ)
Myocardial reperfusion injury	Ischaemic heart disease (SMQ)
Myocardial stunning	Ischaemic heart disease (SMQ)
Papillary muscle infarction	Ischaemic heart disease (SMQ)
Percutaneous coronary intervention	Ischaemic heart disease (SMQ)
Post procedural myocardial infarction	Ischaemic heart disease (SMQ)
Postinfarction angina	Ischaemic heart disease (SMQ)
Prinzmetal angina	Ischaemic heart disease (SMQ)
Scan myocardial perfusion abnormal	Ischaemic heart disease (SMQ)
Silent myocardial infarction	Ischaemic heart disease (SMQ)
Stress cardiomyopathy	Ischaemic heart disease (SMQ)
Stress echocardiogram abnormal	Ischaemic heart disease (SMQ)
Subclavian coronary steal syndrome	Ischaemic heart disease (SMQ)
Subendocardial ischaemia	Ischaemic heart disease (SMQ)
Troponin i increased	Ischaemic heart disease (SMQ)
Troponin increased	Ischaemic heart disease (SMQ)
Troponin t increased	Ischaemic heart disease (SMQ)
Vascular graft occlusion	Ischaemic heart disease (SMQ)
Accelerated idioventricular rhythm	Cardiac arrhythmias (SMQ)
Accessory cardiac pathway	Cardiac arrhythmias (SMQ)
Adams-stokes syndrome	Cardiac arrhythmias (SMQ)
Agonal rhythm	Cardiac arrhythmias (SMQ)
Anomalous atrioventricular excitation	Cardiac arrhythmias (SMQ)
Arrhythmia	Cardiac arrhythmias (SMQ)
Arrhythmia neonatal	Cardiac arrhythmias (SMQ)
Arrhythmia supraventricular	Cardiac arrhythmias (SMQ)
Arrhythmogenic right ventricular dysplasia	Cardiac arrhythmias (SMQ)
Atrial conduction time prolongation	Cardiac arrhythmias (SMQ)
Atrial fibrillation	Cardiac arrhythmias (SMQ)
Atrial flutter	Cardiac arrhythmias (SMQ)
Atrial parasystole	Cardiac arrhythmias (SMQ)
Atrial tachycardia	Cardiac arrhythmias (SMQ)
Atrioventricular block	Cardiac arrhythmias (SMQ)
Atrioventricular block complete	Cardiac arrhythmias (SMQ)
Atrioventricular block first degree	Cardiac arrhythmias (SMQ)
Atrioventricular block second degree	Cardiac arrhythmias (SMQ)

Cardiac disorder events	Sub category
Atrioventricular conduction time shortened	Cardiac arrhythmias (SMQ)
Atrioventricular dissociation	Cardiac arrhythmias (SMQ)
Baseline foetal heart rate variability disorder	Cardiac arrhythmias (SMQ)
Bifascicular block	Cardiac arrhythmias (SMQ)
Bradyarrhythmia	Cardiac arrhythmias (SMQ)
Bradycardia	Cardiac arrhythmias (SMQ)
Bradycardia foetal	Cardiac arrhythmias (SMQ)
Bradycardia neonatal	Cardiac arrhythmias (SMQ)
Brugada syndrome	Cardiac arrhythmias (SMQ)
Brugada syndrome	Cardiac arrhythmias (SMQ)
Bundle branch block	Cardiac arrhythmias (SMQ)
Bundle branch block bilateral	Cardiac arrhythmias (SMQ)
Bundle branch block left	Cardiac arrhythmias (SMQ)
Bundle branch block right	Cardiac arrhythmias (SMQ)
Cardiac arrest	Cardiac arrhythmias (SMQ)
Cardiac arrest neonatal	Cardiac arrhythmias (SMQ)
Cardiac death	Cardiac arrhythmias (SMQ)
Cardiac fibrillation	Cardiac arrhythmias (SMQ)
Cardiac flutter	Cardiac arrhythmias (SMQ)
Cardiac telemetry abnormal	Cardiac arrhythmias (SMQ)
Cardio-respiratory arrest	Cardiac arrhythmias (SMQ)
Cardio-respiratory arrest neonatal	Cardiac arrhythmias (SMQ)
Chronotropic incompetence	Cardiac arrhythmias (SMQ)
Conduction disorder	Cardiac arrhythmias (SMQ)
Defect conduction intraventricular	Cardiac arrhythmias (SMQ)
Ecg p wave inverted	Cardiac arrhythmias (SMQ)
Electrocardiogram abnormal	Cardiac arrhythmias (SMQ)
Electrocardiogram ambulatory abnormal	Cardiac arrhythmias (SMQ)
Electrocardiogram change	Cardiac arrhythmias (SMQ)
Electrocardiogram delta waves abnormal	Cardiac arrhythmias (SMQ)
Electrocardiogram p wave abnormal	Cardiac arrhythmias (SMQ)
Electrocardiogram pq interval prolonged	Cardiac arrhythmias (SMQ)
Electrocardiogram pq interval shortened	Cardiac arrhythmias (SMQ)
Electrocardiogram pr prolongation	Cardiac arrhythmias (SMQ)
Electrocardiogram pr shortened	Cardiac arrhythmias (SMQ)
Electrocardiogram qrs complex prolonged	Cardiac arrhythmias (SMQ)
Electrocardiogram qt prolonged	Cardiac arrhythmias (SMQ)
Electrocardiogram repolarisation abnormality	Cardiac arrhythmias (SMQ)
Electrocardiogram repolarisation abnormality	Cardiac arrhythmias (SMQ)
Electrocardiogram rr interval prolonged	Cardiac arrhythmias (SMQ)
Electrocardiogram u-wave abnormality	Cardiac arrhythmias (SMQ)
Extrasystoles	Cardiac arrhythmias (SMQ)
Foetal arrhythmia	Cardiac arrhythmias (SMQ)
Foetal heart rate acceleration abnormality	Cardiac arrhythmias (SMQ)
Foetal heart rate deceleration abnormality	Cardiac arrhythmias (SMQ)
Foetal heart rate disorder	Cardiac arrhythmias (SMQ)
Heart alternation	Cardiac arrhythmias (SMQ)

Cardiac disorder events	Sub category
Heart block congenital	Cardiac arrhythmias (SMQ)
Heart rate abnormal	Cardiac arrhythmias (SMQ)
Heart rate decreased	Cardiac arrhythmias (SMQ)
Heart rate increased	Cardiac arrhythmias (SMQ)
Heart rate irregular	Cardiac arrhythmias (SMQ)
Junctional ectopic tachycardia	Cardiac arrhythmias (SMQ)
Junctional ectopic tachycardia	Cardiac arrhythmias (SMQ)
Lenegre's disease	Cardiac arrhythmias (SMQ)
Long qt syndrome	Cardiac arrhythmias (SMQ)
Long qt syndrome congenital	Cardiac arrhythmias (SMQ)
Loss of consciousness	Cardiac arrhythmias (SMQ)
Lown-ganong-levine syndrome	Cardiac arrhythmias (SMQ)
Neonatal tachycardia	Cardiac arrhythmias (SMQ)
Nodal arrhythmia	Cardiac arrhythmias (SMQ)
Nodal rhythm	Cardiac arrhythmias (SMQ)
Nonreassuring foetal heart rate pattern	Cardiac arrhythmias (SMQ)
Pacemaker generated arrhythmia	Cardiac arrhythmias (SMQ)
Pacemaker syndrome	Cardiac arrhythmias (SMQ)
Palpitations	Cardiac arrhythmias (SMQ)
Parasystole	Cardiac arrhythmias (SMQ)
Paroxysmal arrhythmia	Cardiac arrhythmias (SMQ)
Pulseless electrical activity	Cardiac arrhythmias (SMQ)
Rebound tachycardia	Cardiac arrhythmias (SMQ)
Reperfusion arrhythmia	Cardiac arrhythmias (SMQ)
Retrograde p-waves	Cardiac arrhythmias (SMQ)
Rhythm idioventricular	Cardiac arrhythmias (SMQ)
Sick sinus syndrome	Cardiac arrhythmias (SMQ)
Sinoatrial block	Cardiac arrhythmias (SMQ)
Sinus arrest	Cardiac arrhythmias (SMQ)
Sinus arrhythmia	Cardiac arrhythmias (SMQ)
Sinus bradycardia	Cardiac arrhythmias (SMQ)
Sinus tachycardia	Cardiac arrhythmias (SMQ)
Sudden cardiac death	Cardiac arrhythmias (SMQ)
Sudden death	Cardiac arrhythmias (SMQ)
Supraventricular extrasystoles	Cardiac arrhythmias (SMQ)
Supraventricular tachyarrhythmia	Cardiac arrhythmias (SMQ)
Supraventricular tachycardia	Cardiac arrhythmias (SMQ)
Syncope	Cardiac arrhythmias (SMQ)
Tachyarrhythmia	Cardiac arrhythmias (SMQ)
Tachycardia	Cardiac arrhythmias (SMQ)
Tachycardia foetal	Cardiac arrhythmias (SMQ)
Tachycardia paroxysmal	Cardiac arrhythmias (SMQ)
Torsade de pointes	Cardiac arrhythmias (SMQ)
Trifascicular block	Cardiac arrhythmias (SMQ)
Ventricular arrhythmia	Cardiac arrhythmias (SMQ)
Ventricular asystole	Cardiac arrhythmias (SMQ)

Cardiac disorder events	Sub category
Ventricular dyssynchrony	Cardiac arrhythmias (SMQ)
Ventricular extrasystoles	Cardiac arrhythmias (SMQ)
Ventricular fibrillation	Cardiac arrhythmias (SMQ)
Ventricular flutter	Cardiac arrhythmias (SMQ)
Ventricular parasystole	Cardiac arrhythmias (SMQ)
Ventricular pre-excitation	Cardiac arrhythmias (SMQ)
Ventricular tachyarrhythmia	Cardiac arrhythmias (SMQ)
Ventricular tachycardia	Cardiac arrhythmias (SMQ)
Wandering pacemaker	Cardiac arrhythmias (SMQ)
Withdrawal arrhythmia	Cardiac arrhythmias (SMQ)
Wolff-parkinson-white syndrome	Cardiac arrhythmias (SMQ)
Wolff-parkinson-white syndrome congenital	Cardiac arrhythmias (SMQ)
Acute left ventricular failure	Cardiac failure (SMQ)
Acute pulmonary oedema	Cardiac failure (SMQ)
Acute right ventricular failure	Cardiac failure (SMQ)
Artificial heart implant	Cardiac failure (SMQ)
Atrial natriuretic peptide abnormal	Cardiac failure (SMQ)
Atrial natriuretic peptide increased	Cardiac failure (SMQ)
Brain natriuretic peptide abnormal	Cardiac failure (SMQ)
Brain natriuretic peptide increased	Cardiac failure (SMQ)
Cardiac asthma	Cardiac failure (SMQ)
Cardiac cirrhosis	Cardiac failure (SMQ)
Cardiac failure	Cardiac failure (SMQ)
Cardiac failure acute	Cardiac failure (SMQ)
Cardiac failure chronic	Cardiac failure (SMQ)
Cardiac failure congestive	Cardiac failure (SMQ)
Cardiac failure high output	Cardiac failure (SMQ)
Cardiac index decreased	Cardiac failure (SMQ)
Cardiac output decreased	Cardiac failure (SMQ)
Cardiac resynchronisation therapy	Cardiac failure (SMQ)
Cardiac ventriculogram abnormal	Cardiac failure (SMQ)
Cardiac ventriculogram left abnormal	Cardiac failure (SMQ)
Cardiac ventriculogram right abnormal	Cardiac failure (SMQ)
Cardiogenic shock	Cardiac failure (SMQ)
Cardiomegaly	Cardiac failure (SMQ)
Cardiopulmonary failure	Cardiac failure (SMQ)
Cardiorenal syndrome	Cardiac failure (SMQ)
Cardio-respiratory distress	Cardiac failure (SMQ)
Cardiothoracic ratio increased	Cardiac failure (SMQ)
Central venous pressure increased	Cardiac failure (SMQ)
Chronic left ventricular failure	Cardiac failure (SMQ)
Chronic right ventricular failure	Cardiac failure (SMQ)
Cor pulmonale	Cardiac failure (SMQ)
Cor pulmonale acute	Cardiac failure (SMQ)
Cor pulmonale chronic	Cardiac failure (SMQ)
Diastolic dysfunction	Cardiac failure (SMQ)

Cardiac disorder events	Sub category
Dilatation ventricular	Cardiac failure (SMQ)
Dyspnoea paroxysmal nocturnal	Cardiac failure (SMQ)
Ejection fraction decreased	Cardiac failure (SMQ)
Heart transplant	Cardiac failure (SMQ)
Hepatic congestion	Cardiac failure (SMQ)
Hepatic vein dilatation	Cardiac failure (SMQ)
Hepatojugular reflux	Cardiac failure (SMQ)
Jugular vein distension	Cardiac failure (SMQ)
Left ventricular dysfunction	Cardiac failure (SMQ)
Left ventricular failure	Cardiac failure (SMQ)
Low cardiac output syndrome	Cardiac failure (SMQ)
Myocardial depression	Cardiac failure (SMQ)
Neonatal cardiac failure	Cardiac failure (SMQ)
Nocturnal dyspnoea	Cardiac failure (SMQ)
N-terminal prohormone brain natriuretic peptide	Cardiac failure (SMQ)
N-terminal prohormone brain natriuretic peptide	Cardiac failure (SMQ)
Obstructive shock	Cardiac failure (SMQ)
Oedema	Cardiac failure (SMQ)
Oedema due to cardiac disease	Cardiac failure (SMQ)
Oedema neonatal	Cardiac failure (SMQ)
Oedema peripheral	Cardiac failure (SMQ)
Orthopnoea	Cardiac failure (SMQ)
Peripheral oedema neonatal	Cardiac failure (SMQ)
Pulmonary congestion	Cardiac failure (SMQ)
Pulmonary oedema	Cardiac failure (SMQ)
Pulmonary oedema neonatal	Cardiac failure (SMQ)
Right ventricular dysfunction	Cardiac failure (SMQ)
Right ventricular failure	Cardiac failure (SMQ)
Scan myocardial perfusion abnormal	Cardiac failure (SMQ)
Stroke volume decreased	Cardiac failure (SMQ)
Systolic dysfunction	Cardiac failure (SMQ)
Venous pressure increased	Cardiac failure (SMQ)
Venous pressure jugular abnormal	Cardiac failure (SMQ)
Venous pressure jugular increased	Cardiac failure (SMQ)
Ventricular assist device insertion	Cardiac failure (SMQ)
Ventricular dysfunction	Cardiac failure (SMQ)
Ventricular dyssynchrony	Cardiac failure (SMQ)
Ventricular failure	Cardiac failure (SMQ)

**Hepatic Dysfunction**	
Bilirubin excretion disorder	Cholestasis and jaundice of hepatic origin (SMQ)
Cholaemia	Cholestasis and jaundice of hepatic origin (SMQ)
Cholestasis	Cholestasis and jaundice of hepatic origin (SMQ)
Cholestatic liver injury	Cholestasis and jaundice of hepatic origin (SMQ)
Cholestatic pruritus	Cholestasis and jaundice of hepatic origin (SMQ)
Deficiency of bile secretion	Cholestasis and jaundice of hepatic origin (SMQ)
Drug-induced liver injury	Cholestasis and jaundice of hepatic origin (SMQ)
Hepatitis cholestatic	Cholestasis and jaundice of hepatic origin (SMQ)
Hyperbilirubinaemia	Cholestasis and jaundice of hepatic origin (SMQ)
Icterus index increased	Cholestasis and jaundice of hepatic origin (SMQ)
Jaundice	Cholestasis and jaundice of hepatic origin (SMQ)
Jaundice cholestatic	Cholestasis and jaundice of hepatic origin (SMQ)
Jaundice hepatocellular	Cholestasis and jaundice of hepatic origin (SMQ)
Mixed liver injury	Cholestasis and jaundice of hepatic origin (SMQ)
Ocular icterus	Cholestasis and jaundice of hepatic origin (SMQ)
Parenteral nutrition associated liver disease	Cholestasis and jaundice of hepatic origin (SMQ)
Yellow skin	Cholestasis and jaundice of hepatic origin (SMQ)
Acute graft versus host disease in liver	Drug related hepatic disorders - severe events only (SMQ)
Acute hepatic failure	Drug related hepatic disorders - severe events only (SMQ)
Acute yellow liver atrophy	Drug related hepatic disorders - severe events only (SMQ)
Allergic hepatitis	Drug related hepatic disorders - severe events only (SMQ)
Anorectal varices	Drug related hepatic disorders - severe events only (SMQ)
Anorectal varices haemorrhage	Drug related hepatic disorders - severe events only (SMQ)
Ascites	Drug related hepatic disorders - severe events only (SMQ)
Asterixis	Drug related hepatic disorders - severe events only (SMQ)
Autoimmune hepatitis	Drug related hepatic disorders - severe events only (SMQ)
Bacterascites	Drug related hepatic disorders - severe events only (SMQ)
Benign hepatic neoplasm	Drug related hepatic disorders - severe events only (SMQ)
Biliary cirrhosis	Drug related hepatic disorders - severe events only (SMQ)
Biliary cirrhosis primary	Drug related hepatic disorders - severe events only (SMQ)
Biliary fibrosis	Drug related hepatic disorders - severe events only (SMQ)
Cholestatic liver injury	Drug related hepatic disorders - severe events only (SMQ)
Chronic graft versus host disease in liver	Drug related hepatic disorders - severe events only (SMQ)
Chronic hepatic failure	Drug related hepatic disorders - severe events only (SMQ)
Chronic hepatitis	Drug related hepatic disorders - severe events only (SMQ)
Coma hepatic	Drug related hepatic disorders - severe events only (SMQ)
Cryptogenic cirrhosis	Drug related hepatic disorders - severe events only (SMQ)
Diabetic hepatopathy	Drug related hepatic disorders - severe events only (SMQ)
Drug-induced liver injury	Drug related hepatic disorders - severe events only (SMQ)
Duodenal varices	Drug related hepatic disorders - severe events only (SMQ)
Focal nodular hyperplasia	Drug related hepatic disorders - severe events only (SMQ)

Hepatic Dysfunction	
Gallbladder varices	Drug related hepatic disorders - severe events only (SMQ)
Gastric varices	Drug related hepatic disorders - severe events only (SMQ)
Gastric varices haemorrhage	Drug related hepatic disorders - severe events only (SMQ)
Graft versus host disease in liver	Drug related hepatic disorders - severe events only (SMQ)
Granulomatous liver disease	Drug related hepatic disorders - severe events only (SMQ)
Haemangioma of liver	Drug related hepatic disorders - severe events only (SMQ)
Haemorrhagic hepatic cyst	Drug related hepatic disorders - severe events only (SMQ)
Hepatectomy	Drug related hepatic disorders - severe events only (SMQ)
Hepatic adenoma	Drug related hepatic disorders - severe events only (SMQ)
Hepatic angiosarcoma	Drug related hepatic disorders - severe events only (SMQ)
Hepatic atrophy	Drug related hepatic disorders - severe events only (SMQ)
Hepatic calcification	Drug related hepatic disorders - severe events only (SMQ)
Hepatic cancer	Drug related hepatic disorders - severe events only (SMQ)
Hepatic cancer metastatic	Drug related hepatic disorders - severe events only (SMQ)
Hepatic cancer recurrent	Drug related hepatic disorders - severe events only (SMQ)
Hepatic cancer stage i	Drug related hepatic disorders - severe events only (SMQ)
Hepatic cancer stage ii	Drug related hepatic disorders - severe events only (SMQ)
Hepatic cancer stage iii	Drug related hepatic disorders - severe events only (SMQ)
Hepatic cancer stage iv	Drug related hepatic disorders - severe events only (SMQ)
Hepatic cirrhosis	Drug related hepatic disorders - severe events only (SMQ)
Hepatic cyst	Drug related hepatic disorders - severe events only (SMQ)
Hepatic cyst ruptured	Drug related hepatic disorders - severe events only (SMQ)
Hepatic encephalopathy	Drug related hepatic disorders - severe events only (SMQ)
Hepatic encephalopathy prophylaxis	Drug related hepatic disorders - severe events only (SMQ)
Hepatic failure	Drug related hepatic disorders - severe events only (SMQ)
Hepatic fibrosis	Drug related hepatic disorders - severe events only (SMQ)
Hepatic haemangioma rupture	Drug related hepatic disorders - severe events only (SMQ)
Hepatic hydrothorax	Drug related hepatic disorders - severe events only (SMQ)
Hepatic infiltration eosinophilic	Drug related hepatic disorders - severe events only (SMQ)
Hepatic lesion	Drug related hepatic disorders - severe events only (SMQ)
Hepatic necrosis	Drug related hepatic disorders - severe events only (SMQ)
Hepatic neoplasm	Drug related hepatic disorders - severe events only (SMQ)
Hepatic steatosis	Drug related hepatic disorders - severe events only (SMQ)
Hepatitis	Drug related hepatic disorders - severe events only (SMQ)
Hepatitis acute	Drug related hepatic disorders - severe events only (SMQ)
Hepatitis cholestatic	Drug related hepatic disorders - severe events only (SMQ)
Hepatitis chronic active	Drug related hepatic disorders - severe events only (SMQ)
Hepatitis chronic persistent	Drug related hepatic disorders - severe events only (SMQ)
Hepatitis fulminant	Drug related hepatic disorders - severe events only (SMQ)
Hepatitis fulminant	Drug related hepatic disorders - severe events only (SMQ)
Hepatitis toxic	Drug related hepatic disorders - severe events only (SMQ)
Hepatobiliary cancer	Drug related hepatic disorders - severe events only (SMQ)

**Hepatic Dysfunction**	
Hepatobiliary cancer in situ	Drug related hepatic disorders - severe events only (SMQ)
Hepatobiliary disease	Drug related hepatic disorders - severe events only (SMQ)
Hepatobiliary neoplasm	Drug related hepatic disorders - severe events only (SMQ)
Hepatoblastoma	Drug related hepatic disorders - severe events only (SMQ)
Hepatoblastoma recurrent	Drug related hepatic disorders - severe events only (SMQ)
Hepatocellular carcinoma	Drug related hepatic disorders - severe events only (SMQ)
Hepatocellular foamy cell syndrome	Drug related hepatic disorders - severe events only (SMQ)
Hepatocellular injury	Drug related hepatic disorders - severe events only (SMQ)
Hepatopulmonary syndrome	Drug related hepatic disorders - severe events only (SMQ)
Hepatorenal failure	Drug related hepatic disorders - severe events only (SMQ)
Hepatorenal syndrome	Drug related hepatic disorders - severe events only (SMQ)
Hepatotoxicity	Drug related hepatic disorders - severe events only (SMQ)
Intestinal varices	Drug related hepatic disorders - severe events only (SMQ)
Intrahepatic portal hepatic venous fistula	Drug related hepatic disorders - severe events only (SMQ)
Ischaemic hepatitis	Drug related hepatic disorders - severe events only (SMQ)
Liver ablation	Drug related hepatic disorders - severe events only (SMQ)
Liver and small intestine transplant	Drug related hepatic disorders - severe events only (SMQ)
Liver carcinoma ruptured	Drug related hepatic disorders - severe events only (SMQ)
Liver disorder	Drug related hepatic disorders - severe events only (SMQ)
Liver injury	Drug related hepatic disorders - severe events only (SMQ)
Liver operation	Drug related hepatic disorders - severe events only (SMQ)
Liver sarcoidosis	Drug related hepatic disorders - severe events only (SMQ)
Liver transplant	Drug related hepatic disorders - severe events only (SMQ)
Lupoid hepatic cirrhosis	Drug related hepatic disorders - severe events only (SMQ)
Lupus hepatitis	Drug related hepatic disorders - severe events only (SMQ)
Mixed hepatocellular cholangiocarcinoma	Drug related hepatic disorders - severe events only (SMQ)
Mixed liver injury	Drug related hepatic disorders - severe events only (SMQ)
Nodular regenerative hyperplasia	Drug related hepatic disorders - severe events only (SMQ)
Non-alcoholic steatohepatitis	Drug related hepatic disorders - severe events only (SMQ)
Non-alcoholic steatohepatitis	Drug related hepatic disorders - severe events only (SMQ)
Oedema due to hepatic disease	Drug related hepatic disorders - severe events only (SMQ)
Oesophageal varices haemorrhage	Drug related hepatic disorders - severe events only (SMQ)
Peripancreatic varices	Drug related hepatic disorders - severe events only (SMQ)
Peritoneovenous shunt	Drug related hepatic disorders - severe events only (SMQ)
Portal fibrosis	Drug related hepatic disorders - severe events only (SMQ)
Portal hypertension	Drug related hepatic disorders - severe events only (SMQ)
Portal hypertensive enteropathy	Drug related hepatic disorders - severe events only (SMQ)
Portal hypertensive gastropathy	Drug related hepatic disorders - severe events only (SMQ)
Portal shunt	Drug related hepatic disorders - severe events only (SMQ)
Portal tract inflammation	Drug related hepatic disorders - severe events only (SMQ)
Portal vein cavernous transformation	Drug related hepatic disorders - severe events only (SMQ)

**Hepatic Dysfunction**	
Portal vein dilatation	Drug related hepatic disorders - severe events only (SMQ)
Portopulmonary hypertension	Drug related hepatic disorders - severe events only (SMQ)
Radiation hepatitis	Drug related hepatic disorders - severe events only (SMQ)
Renal and liver transplant	Drug related hepatic disorders - severe events only (SMQ)
Retrograde portal vein flow	Drug related hepatic disorders - severe events only (SMQ)
Reye's syndrome	Drug related hepatic disorders - severe events only (SMQ)
Reynold's syndrome	Drug related hepatic disorders - severe events only (SMQ)
Small-for-size liver syndrome	Drug related hepatic disorders - severe events only (SMQ)
Spider naevus	Drug related hepatic disorders - severe events only (SMQ)
Splenic varices	Drug related hepatic disorders - severe events only (SMQ)
Splenic varices haemorrhage	Drug related hepatic disorders - severe events only (SMQ)
Stomal varices	Drug related hepatic disorders - severe events only (SMQ)
Subacute hepatic failure	Drug related hepatic disorders - severe events only (SMQ)
Varices oesophageal	Drug related hepatic disorders - severe events only (SMQ)
Varicose veins of abdominal wall	Drug related hepatic disorders - severe events only (SMQ)
5'nucleotidase increased	Liver related investigations, signs and symptoms (SMQ)
Alanine aminotransferase abnormal	Liver related investigations, signs and symptoms (SMQ)
Alanine aminotransferase increased	Liver related investigations, signs and symptoms (SMQ)
Ammonia abnormal	Liver related investigations, signs and symptoms (SMQ)
Ammonia increased	Liver related investigations, signs and symptoms (SMQ)
Ascites	Liver related investigations, signs and symptoms (SMQ)
Aspartate aminotransferase abnormal	Liver related investigations, signs and symptoms (SMQ)
Aspartate aminotransferase increased	Liver related investigations, signs and symptoms (SMQ)
Bacterascites	Liver related investigations, signs and symptoms (SMQ)
Bile output abnormal	Liver related investigations, signs and symptoms (SMQ)
Bile output decreased	Liver related investigations, signs and symptoms (SMQ)
Biliary ascites	Liver related investigations, signs and symptoms (SMQ)
Bilirubin conjugated abnormal	Liver related investigations, signs and symptoms (SMQ)
Bilirubin conjugated increased	Liver related investigations, signs and symptoms (SMQ)
Biopsy liver abnormal	Liver related investigations, signs and symptoms (SMQ)
Blood alkaline phosphatase abnormal	Liver related investigations, signs and symptoms (SMQ)
Blood alkaline phosphatase increased	Liver related investigations, signs and symptoms (SMQ)
Blood bilirubin abnormal	Liver related investigations, signs and symptoms (SMQ)
Blood bilirubin increased	Liver related investigations, signs and symptoms (SMQ)
Blood bilirubin unconjugated increased	Liver related investigations, signs and symptoms (SMQ)
Blood cholinesterase abnormal	Liver related investigations, signs and symptoms (SMQ)
Blood cholinesterase decreased	Liver related investigations, signs and symptoms (SMQ)
Bromosulphthalein test abnormal	Liver related investigations, signs and symptoms (SMQ)
Child-pugh-turcotte score increased	Liver related investigations, signs and symptoms (SMQ)
Deficiency of bile secretion	Liver related investigations, signs and symptoms (SMQ)
Foetor hepaticus	Liver related investigations, signs and symptoms (SMQ)
Galactose elimination capacity test	Liver related investigations, signs and symptoms (SMQ)

**Hepatic Dysfunction**	
abnormal	
Galactose elimination capacity test decreased	Liver related investigations, signs and symptoms (SMQ)
Gamma-glutamyltransferase abnormal	Liver related investigations, signs and symptoms (SMQ)
Gamma-glutamyltransferase increased	Liver related investigations, signs and symptoms (SMQ)
Glutamate dehydrogenase increased	Liver related investigations, signs and symptoms (SMQ)
Guanase increased	Liver related investigations, signs and symptoms (SMQ)
Haemorrhagic ascites	Liver related investigations, signs and symptoms (SMQ)
Hepaplastin abnormal	Liver related investigations, signs and symptoms (SMQ)
Hepaplastin decreased	Liver related investigations, signs and symptoms (SMQ)
Hepatic artery flow decreased	Liver related investigations, signs and symptoms (SMQ)
Hepatic congestion	Liver related investigations, signs and symptoms (SMQ)
Hepatic enzyme abnormal	Liver related investigations, signs and symptoms (SMQ)
Hepatic enzyme decreased	Liver related investigations, signs and symptoms (SMQ)
Hepatic enzyme increased	Liver related investigations, signs and symptoms (SMQ)
Hepatic fibrosis marker abnormal	Liver related investigations, signs and symptoms (SMQ)
Hepatic fibrosis marker increased	Liver related investigations, signs and symptoms (SMQ)
Hepatic function abnormal	Liver related investigations, signs and symptoms (SMQ)
Hepatic hydrothorax	Liver related investigations, signs and symptoms (SMQ)
Hepatic mass	Liver related investigations, signs and symptoms (SMQ)
Hepatic pain	Liver related investigations, signs and symptoms (SMQ)
Hepatic sequestration	Liver related investigations, signs and symptoms (SMQ)
Hepatic vascular resistance increased	Liver related investigations, signs and symptoms (SMQ)
Hepatobiliary scan abnormal	Liver related investigations, signs and symptoms (SMQ)
Hepatomegaly	Liver related investigations, signs and symptoms (SMQ)
Hepatosplenomegaly	Liver related investigations, signs and symptoms (SMQ)
Hyperammonaemia	Liver related investigations, signs and symptoms (SMQ)
Hyperbilirubinaemia	Liver related investigations, signs and symptoms (SMQ)
Hypercholia	Liver related investigations, signs and symptoms (SMQ)
Hypertransaminasaemia	Liver related investigations, signs and symptoms (SMQ)
Hypoalbuminaemia	Liver related investigations, signs and symptoms (SMQ)
Kayser-fleischer ring	Liver related investigations, signs and symptoms (SMQ)
Leucine aminopeptidase increased	Liver related investigations, signs and symptoms (SMQ)
Liver function test abnormal	Liver related investigations, signs and symptoms (SMQ)
Liver induration	Liver related investigations, signs and symptoms (SMQ)
Liver iron concentration abnormal	Liver related investigations, signs and symptoms (SMQ)
Liver iron concentration increased	Liver related investigations, signs and symptoms (SMQ)
Liver palpable subcostal	Liver related investigations, signs and symptoms (SMQ)
Liver scan abnormal	Liver related investigations, signs and symptoms (SMQ)
Liver tenderness	Liver related investigations, signs and symptoms (SMQ)
Mitochondrial aspartate aminotransferase increased	Liver related investigations, signs and symptoms (SMQ)
Molar ratio of total branched-chain	Liver related investigations, signs and symptoms (SMQ)

**Hepatic Dysfunction**	
amino acid to tyrosine	
Oedema due to hepatic disease	Liver related investigations, signs and symptoms (SMQ)
Perihepatic discomfort	Liver related investigations, signs and symptoms (SMQ)
Periportal oedema	Liver related investigations, signs and symptoms (SMQ)
Peritoneal fluid protein abnormal	Liver related investigations, signs and symptoms (SMQ)
Peritoneal fluid protein decreased	Liver related investigations, signs and symptoms (SMQ)
Peritoneal fluid protein increased	Liver related investigations, signs and symptoms (SMQ)
Pneumobilia	Liver related investigations, signs and symptoms (SMQ)
Portal vein flow decreased	Liver related investigations, signs and symptoms (SMQ)
Portal vein pressure increased	Liver related investigations, signs and symptoms (SMQ)
Retinol binding protein decreased	Liver related investigations, signs and symptoms (SMQ)
Retrograde portal vein flow	Liver related investigations, signs and symptoms (SMQ)
Total bile acids increased	Liver related investigations, signs and symptoms (SMQ)
Transaminases abnormal	Liver related investigations, signs and symptoms (SMQ)
Transaminases increased	Liver related investigations, signs and symptoms (SMQ)
Ultrasound liver abnormal	Liver related investigations, signs and symptoms (SMQ)
Urine bilirubin increased	Liver related investigations, signs and symptoms (SMQ)
Urobilinogen urine decreased	Liver related investigations, signs and symptoms (SMQ)
Urobilinogen urine increased	Liver related investigations, signs and symptoms (SMQ)
X-ray hepatobiliary abnormal	Liver related investigations, signs and symptoms (SMQ)
Abnormal faeces	Biliary system related investigations, signs and symptoms (SMQ)
Bile culture positive	Biliary system related investigations, signs and symptoms (SMQ)
Bile duct pressure increased	Biliary system related investigations, signs and symptoms (SMQ)
Bile output abnormal	Biliary system related investigations, signs and symptoms (SMQ)
Bile output decreased	Biliary system related investigations, signs and symptoms (SMQ)
Bile output increased	Biliary system related investigations, signs and symptoms (SMQ)
Biliary ascites	Biliary system related investigations, signs and symptoms (SMQ)
Bilirubin conjugated abnormal	Biliary system related investigations, signs and symptoms (SMQ)
Bilirubin conjugated increased	Biliary system related investigations, signs and symptoms (SMQ)
Bilirubin excretion disorder	Biliary system related investigations, signs and symptoms (SMQ)
Bilirubinuria	Biliary system related investigations, signs and symptoms (SMQ)
Biopsy bile duct abnormal	Biliary system related investigations, signs and symptoms (SMQ)
Blood alkaline phosphatase abnormal	Biliary system related investigations, signs and symptoms

**Hepatic Dysfunction**	
	(SMQ)
Blood alkaline phosphatase increased	Biliary system related investigations, signs and symptoms (SMQ)
Blood bilirubin abnormal	Biliary system related investigations, signs and symptoms (SMQ)
Blood bilirubin increased	Biliary system related investigations, signs and symptoms (SMQ)
Blood bilirubin unconjugated increased	Biliary system related investigations, signs and symptoms (SMQ)
Cholangiogram abnormal	Biliary system related investigations, signs and symptoms (SMQ)
Cholecystogram intravenous abnormal	Biliary system related investigations, signs and symptoms (SMQ)
Cholecystogram oral abnormal	Biliary system related investigations, signs and symptoms (SMQ)
Deficiency of bile secretion	Biliary system related investigations, signs and symptoms (SMQ)
Endoscopic retrograde cholangiopancreatography abnormal	Biliary system related investigations, signs and symptoms (SMQ)
Endoscopy biliary tract abnormal	Biliary system related investigations, signs and symptoms (SMQ)
Faeces pale	Biliary system related investigations, signs and symptoms (SMQ)
Gallbladder palpable	Biliary system related investigations, signs and symptoms (SMQ)
Hyperbilirubinaemia	Biliary system related investigations, signs and symptoms (SMQ)
Jaundice	Biliary system related investigations, signs and symptoms (SMQ)
Jaundice cholestatic	Biliary system related investigations, signs and symptoms (SMQ)
Jaundice extrahepatic obstructive	Biliary system related investigations, signs and symptoms (SMQ)
Limy bile syndrome	Biliary system related investigations, signs and symptoms (SMQ)
Ultrasound biliary tract abnormal	Biliary system related investigations, signs and symptoms (SMQ)
Urine bilirubin increased	Biliary system related investigations, signs and symptoms (SMQ)
Urobilinogen urine decreased	Biliary system related investigations, signs and symptoms (SMQ)
X-ray hepatobiliary abnormal	Biliary system related investigations, signs and symptoms (SMQ)

**Infection events**	
Arthritis infective	Bronchitis
Bronchitis bacterial	Campylobacter gastroenteritis
Candidiasis	Catheter related infection
Cellulitis	Clostridial infection
Cystitis	Ear infection
Enterococcal infection	Escherichia urinary tract infection
Eye infection	Eyelid infection
Febrile infection	Fungal infection
Fungal skin infection	Gastrointestinal infection
Gingival infection	Herpes virus infection
Herpes zoster	Infection
Localised infection	Lower respiratory tract infection
Lower respiratory tract infection viral	Lung infection
Nasopharyngitis	Oesophageal candidiasis
Oesophageal infection	Onychomycosis
Oral candidiasis	Oral fungal infection
Oral infection	Osteomyelitis
Pharyngitis	Pneumonia
Postoperative wound infection	Proteus infection
Respiratory tract infection	Rhinorrhoea
Rotavirus infection	Sepsis
Sinusitis	Skin candida
Skin infection	Staphylococcal infection
Tinea pedis	Tooth abscess
Tooth infection	Upper respiratory tract infection
Urinary tract infection	Urinary tract infection bacterial
Urosepsis	Viral infection
Viral upper respiratory tract infection	Wound infection

**Osteoporosis events**	
Acetabulum fracture	Body height abnormal
Body height below normal	Body height decreased
Bone density abnormal	Bone density decreased
Bone formation decreased	Bone formation test
Bone formation test abnormal	Bone loss
Bone marrow oedema syndrome	Bone metabolism disorder
Bone resorption test	Bone resorption test abnormal
Cervical vertebral fracture	Closed fracture manipulation
C-telopeptide	C-telopeptide increased
Deoxypyridinoline urine increased	External fixation of fracture
Femoral neck fracture	Femur fracture
Forearm fracture	Fracture
Fracture treatment	Fractured ischium
Fractured sacrum	Hip arthroplasty
Hip fracture	Hip surgery
Ilium fracture	Internal fixation of fracture
Kyphoscoliosis	Kyphosis
Lumbar vertebral fracture	Multiple fractures
N-telopeptide urine increased	Open reduction of fracture
Open reduction of spinal fracture	Osteocalcin increased
Osteopenia	Osteoporosis
Osteoporosis postmenopausal	Osteoporosis prophylaxis
Osteoporotic fracture	Pelvic fracture
Post-traumatic osteoporosis	Pubis fracture
Pyridinoline urine increased	Radius fracture
Resorption bone increased	Rib fracture
Senile osteoporosis	Spinal compression fracture
Spinal deformity	Spinal fracture
Thoracic vertebral fracture	Vertebroplasty
Wrist fracture	Wrist surgery

Hyperglycaemia/New Onset Diabetes Mellitus (Smq)	
Abnormal loss of weight	Abnormal weight gain
Acidosis	Altered state of consciousness
Blood cholesterol increased	Blood glucose abnormal
Blood glucose increased	Blood insulin abnormal
Blood insulin decreased	Blood lactic acid increased
Blood osmolarity increased	Blood triglycerides increased
Body mass index decreased	Body mass index increased
Coma	Dehydration
Depressed level of consciousness	Diabetes complicating pregnancy
Diabetes mellitus	Diabetes mellitus inadequate control
Diabetes with hyperosmolarity	Diabetic coma
Diabetic hyperglycaemic coma	Diabetic hyperosmolar coma
Diabetic ketoacidosis	Diabetic ketoacidotic hyperglycaemic coma
Fructosamine increased	Gestational diabetes
Glucose tolerance decreased	Glucose tolerance impaired
Glucose tolerance impaired in pregnancy	Glucose tolerance test abnormal
Glycosuria	Glycosuria during pregnancy
Glucose urine present	Glycosylated haemoglobin increased
Hunger	Hypercholesterolaemia
Hyperglycaemia	Hyperlactacidaemia
Hyperosmolar state	Hyperphagia
Hypertriglyceridaemia	Hypoglycaemia
Increased appetite	Increased insulin requirement
Insulin autoimmune syndrome	Insulin resistance
Insulin resistance syndrome	Insulin resistant diabetes
Insulin tolerance test abnormal	Ketoacidosis
Ketonuria	Ketosis
Lactic acidosis	Lipids increased
Loss of consciousness	Metabolic acidosis
Neonatal diabetes mellitus	Obesity
Overweight	Pancreatogenous diabetes
Polydipsia	Polyuria
Slow response to stimuli	Thirst
Unresponsive to stimuli	Weight decreased
Weight increased	Underweight
Anti-islet cell antibody positive	Blood glucose fluctuation
Metabolic syndrome	Impaired insulin secretion
Insulin-requiring type 2 diabetes mellitus	Anti-insulin antibody positive
Anti-insulin antibody increased	Impaired fasting glucose
Urine ketone body present	Anti-gad antibody positive
Hyperlipidaemia	Hyperglycaemic hyperosmolar nonketotic syndrome
Blood 1,5-anhydroglucitol decreased	Central obesity
Latent autoimmune diabetes in adults	Type 1 diabetes mellitus
Type 2 diabetes mellitus	Anti-insulin receptor antibody positive
Anti-insulin receptor antibody increased	Hypoinsulinaemia

Diabetic hepatopathy	Hyperglycaemic unconsciousness
Hyperglycaemic seizure	Anti-ia2 antibody positive
Anti-zinc transporter 8 antibody positive	Fulminant type 1 diabetes mellitus
Type 3 diabetes mellitus	Acquired lipoatrophic diabetes
Diabetic metabolic decompensation	

**Weight gain**	
Weight increased	

**Fatigue**	
Fatigue	
