# Supplementary material for: Assessment of the Safety of Glucocorticoid Regimens in Combination With Abiraterone Acetate for Metastatic Castration-Resistant Prostate Cancer: A Randomized, Open-label Phase 2 Study
Source: JAMA Oncol. 2019 Jun 27;5(8):1159–67. doi: 10.1001/jamaoncol.2019.1011 (PMC6604092; doi:10.1001/jamaoncol.2019.1011)
Supplement: Supplement 2. — eMethods eReferences eFigure 1. Patient Disposition eFigure 2. Changes from baseline to cycle 3 by treatment group for urinary corticosterone metabolites (A), the major androgen metabolite androsterone (B), and DHEA (C); and values at cycle 3 for the urinary metabolites of corticosterone (D) and cortisone (E) among patients experiencing or not experiencing clinical mineralocorticoid excess toxicity (grade ≥2 hypertension or grade ≥1 hypokalemia) in the first 24 weeks eFigure 3. Values at cycle 3 for the urinary metabolites of corticosterone (A) and cortisone (B) among patients experiencing or not experiencing clinical mineralocorticoid excess toxicity (grade ≥2 hypertension or grade ≥1 hypokalemia) in the first 24 weeks eFigure 4. Cumulative incidences for adverse events of hypokalemia or hypertension eFigure 5. Change from baseline in fasting serum insulin (A) and HOMA-IR (B), and percentage change from baseline in total lean body mass (C) and total body fat (D) at end of main study (up to 39 cycles) eFigure 6. Clinical benefit: Kaplan-Meier plot of radiographic progression-free survival during the main study for intention-to-treat population, divided by treatment group (A); and patient-reported quality of life as measured by EQ-5D-5L and FACT-P (B) eTable 1. Study investigators and centers eTable 2. Full list of urinary steroid metabolites analyzed by gas chromatography-mass spectrometry eTable 3. Baseline characteristics eTable 4. Changes from baseline to cycle 3 by treatment group for plasma ACTH and key urinary steroid metabolites eTable 5. Urinary steroid metabolites with P<0.1 for the comparison of values at cycle 3 day 1 between patients with or without clinical mineralocorticoid excess toxicity in the first 24 weeks eTable 6. Adverse events of hypokalemia by treatment group and severity eTable 7. Adverse events of special interest during the main study (up to 39 cycles) eTable 8. Changes from baseline in bone mineral density - total body and arms eTable 9. [file jamaoncol-5-1159-s002.pdf]

# Supplementary Online Content

Attard G, Merseburger AS, Arlt W, et al. Assessment of the safety of glucocorticoid regimens in combination with abiraterone acetate for metastatic castration-resistant prostate cancer: a randomized, open-label phase-2 study. Published online June 27, 2019. *JAMA Oncology*. doi:10.1001/jamaoncol.2019.1011

**eMethods**

**eReferences**

**eFigure 1**. Patient Disposition

**eFigure 2.** Changes from baseline to cycle 3 by treatment group for urinary corticosterone metabolites (A), the major androgen metabolite androsterone (B), and DHEA (C); and values at cycle 3 for the urinary metabolites of corticosterone (D) and cortisone (E) among patients experiencing or not experiencing clinical mineralocorticoid excess toxicity (grade ≥2 hypertension or grade ≥1 hypokalemia) in the first 24 weeks

**eFigure 3.** Values at cycle 3 for the urinary metabolites of corticosterone (A) and cortisone (B) among patients experiencing or not experiencing clinical mineralocorticoid excess toxicity (grade ≥2 hypertension or grade ≥1 hypokalemia) in the first 24 weeks

**eFigure 4.** Cumulative incidences for adverse events of hypokalemia or hypertension

**eFigure 5.** Change from baseline in fasting serum insulin (A) and HOMA-IR (B), and percentage change from baseline in total lean body mass (C) and total body fat (D) at end of main study (up to 39 cycles)

**eFigure 6.** Clinical benefit: Kaplan-Meier plot of radiographic progression-free survival during the main study for intention-to-treat population, divided by treatment group (A); and patient-reported quality of life as measured by EQ-5D-5L and FACT-P (B)

**eTable 1.** Study investigators and centers

© 2019 Attard G et al. *JAMA Oncology*.

**eTable 2.** Full list of urinary steroid metabolites analyzed by gas chromatography-mass spectrometry

**eTable 3.** Baseline characteristics

**eTable 4.** Changes from baseline to cycle 3 by treatment group for plasma ACTH and key urinary steroid metabolites

**eTable 5.** Urinary steroid metabolites with P<0.1 for the comparison of values at cycle 3 day 1 between patients with or without clinical mineralocorticoid excess toxicity in the first 24 weeks

**eTable 6.** Adverse events of hypokalemia by treatment group and severity

**eTable 7.** Adverse events of special interest during the main study (up to 39 cycles)

**eTable 8.** Changes from baseline in bone mineral density - total body and arms

**eTable 9.** Number of patients with any PSA response and confirmed PSA response during the main study (up to 39 cycles)

This supplementary material has been provided by the authors to give readers additional information about their work.

© 2019 Attard G et al. *JAMA Oncology*.

Gerhardt Attard, MD, PhD[1], Axel S Merseburger, MD[2], Wiebke Arlt, MD, DSc[3], Cora N Sternberg, MD[4], Susan Feyerabend, MD[5], Alfredo Berruti, MD[6], Steven Joniau, MD, PhD[7], Lajos Géczi, MD[8], Florence Lefresne, MD[9], Marjolein Lahaye, MSc[9], Florence Nave Shelby, PhD[9], Geneviève Pissart, MSc[9], Sue Chua, MBBS[10], Robert J Jones, PhD[11], Bertrand Tombal, MD, PhD[12]

**Affiliations**

[1]University College London Cancer Institute, London, UK; [2]Department of Urology, University Hospital Schleswig-Holstein, Lübeck, Germany; [3]Institute of Metabolism and Systems Research (IMSR), University of Birmingham & Centre for Endocrinology, Diabetes and Metabolism (CEDAM), Birmingham Health Partners, Birmingham, UK; [4]San Camillo and Forlanini Hospitals, Department of Medical Oncology, Rome, Italy; [5]Studienpraxis Urologie, Nürtingen, Germany; [6]Department of Medical and Surgical Specialties, Radiological Sciences and Public Health, University of Brescia, Brescia, Italy; [7]Department of Urology, University Hospitals Leuven, Leuven, Belgium; [8]National Institute of Oncology, Budapest, Hungary; [9]Janssen Research and Development, Beerse, Belgium; [10]Department of Nuclear Medicine and PET, Royal Marsden NHS Foundation Trust, London, UK; [11]Institute of Cancer Sciences, University of Glasgow, UK; [12]Institut de Recherche Clinique, Université Catholique de Louvain, Brussels, Belgium.

© 2019 Attard G et al. *JAMA Oncology*.

**eMethods**

**Additional Information on Study Procedures**

Plasma adrenocorticotropic hormone (ACTH) was collected at 4 degrees Celsius and analyzed locally. Urine was collected with a record of 24-hour urine volume on day 1 of cycle 1 (with collection starting 24 hours before initiation of treatment), day 1 of cycles 3, 6, and every 6 cycles until cycle 36, and at the end of the main study. Frozen aliquots were sent in batches to a central laboratory where urinary steroids were analyzed using gas chromatography-mass spectrometry as described previously[1] in a clinically-accredited laboratory (Alta Bioscience, Birmingham, UK). Serum insulin and glucose were measured on day 1 of cycles 1, 2, 3, 6, 12, and 18, and at the end of the main study. Dual-energy x-ray absorptiometry scans to assess bone mineral density, total body fat and lean body mass were performed up to 28 days prior to cycle 1, at cycles 3, 6, and 12, and at the end of the main study. Serum PSA was measured on day 1 of cycles 1-6 and every three cycles thereafter. Radiographic disease progression was assessed by CT and bone scans performed up to 28 days prior to cycle 1 and every 12 weeks on treatment, or earlier if clinically indicated. To assess health-related quality of life, patients completed the EuroQol five dimensions (EQ-5D-5L) questionnaire[2] and the Functional Assessment of Cancer Therapy - Prostate Cancer (FACT-P) questionnaire[3] on day 1 of cycles 1, 6, and 18 and at the end of the main study.

**Information on Urinary Steroid Analysis**

For evaluation of the effect on steroid biosynthesis, we evaluated changes between day 1 of cycles 1 and 3 of: urinary metabolites of steroids upstream of CYP17A1, deoxycorticosterone (sum of tetrahydro-11-deoxycorticosterone and 5α-tetrahydro-11-deoxycorticosterone) and corticosterone (sum of tetrahydrocorticosterone, 5α-tetrahydrocorticosterone, tetrahydro-11-dehydrocorticosterone, and 5α-tetrahydro-11-dehydrocorticosterone); and urinary metabolites of downstream steroids, namely the major androgen metabolite androsterone, the adrenal androgen precursor dehydroepiandrosterone (DHEA), and

© 2019 Attard G et al. *JAMA Oncology.*

the sum of androgen precursors (dehydroepiandrosterone, 16α-hydroxy-DHEA, pregnenetriol, and pregnenediol).

**eReferences**

1. Attard G, Reid AH, Yap TA, et al. Phase I clinical trial of a selective inhibitor of CYP17, abiraterone acetate, confirms that castration-resistant prostate cancer commonly remains hormone driven. *J Clin Oncol.* 2008;26(28):4563-4571.

2. Herdman M, Gudex C, Lloyd A, et al. Development and preliminary testing of the new five-level version of EQ-5D (EQ-5D-5L). *Qual Life Res.* 2011;20(10):1727-1736.

3. Esper P, Mo F, Chodak G, Sinner M, Cella D, Pienta KJ. Measuring quality of life in men with prostate cancer using the functional assessment of cancer therapy-prostate instrument. *Urology.* 1997;50(6):920-928.

© 2019 Attard G et al. *JAMA Oncology.*

**eFigure 1. Patient Disposition**

*Patients completing the 24 weeks of treatment or discontinuing the 24 weeks early and experiencing either hypertension (CTCAE Grade ≥2) or hypokalemia. †Includes adverse event requiring treatment with diuretics, adverse event of grade 3 or 4 hypokalemia, adverse event with sustained toxicity greater than NCI-CTCAE (v4.0) grade 1, or other adverse event. ‡Includes 1 patient whose study treatment regimen was permanently changed by investigator. §Patients who required diuretic treatment or a change in glucocorticoid dose, or who experienced grade 3 or 4 hypokalemia, could join the extension protocol at improvement to grade ≤1 and in the absence of disease progression; patients who completed the main study also could join the extension protocol.

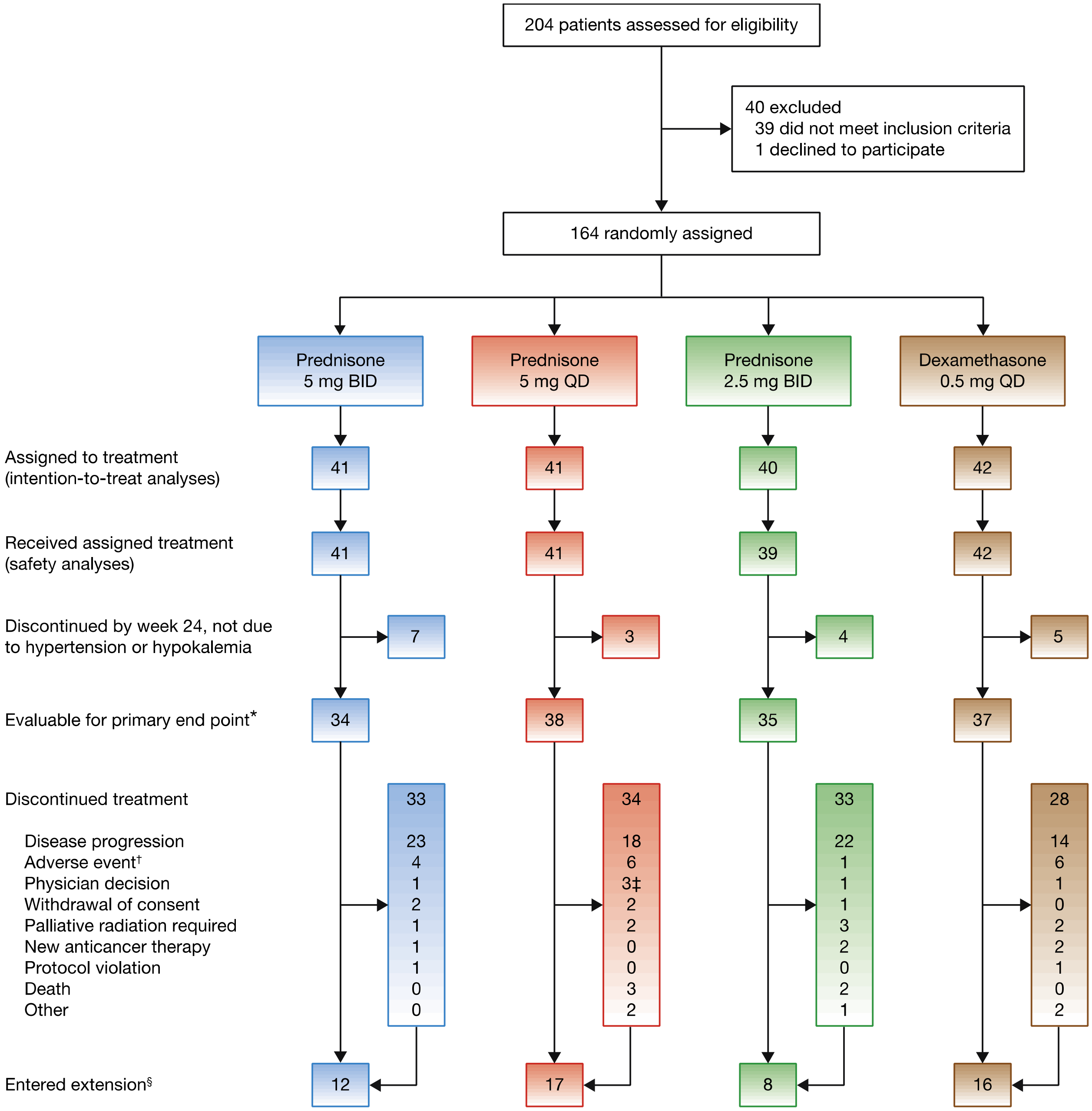


© 2019 Attard G et al. *JAMA Oncology*.

**eFigure 2. Changes from baseline to cycle 3 by treatment group for urinary corticosterone metabolites (A), the major androgen metabolite androsterone (B), and DHEA (C); and values at cycle 3 for the urinary metabolites of corticosterone (D) and cortisone (E) among patients experiencing or not experiencing clinical mineralocorticoid excess toxicity (grade ≥2 hypertension or grade ≥1 hypokalemia) in the first 24 weeks**

DHEA, dehydroepiandrosterone. Violin plots represent the four treatment regimen arms. Boxes represent median (horizontal bar), mean (X) and quartiles (Q1 and Q3), and whiskers indicate quartiles ± 1.5 × (Q3-Q1). Minimum and maximum values are the lowest and highest dots. Significant changes from baseline, by Wilcoxon Signed Rank test, are noted in panels A-C.

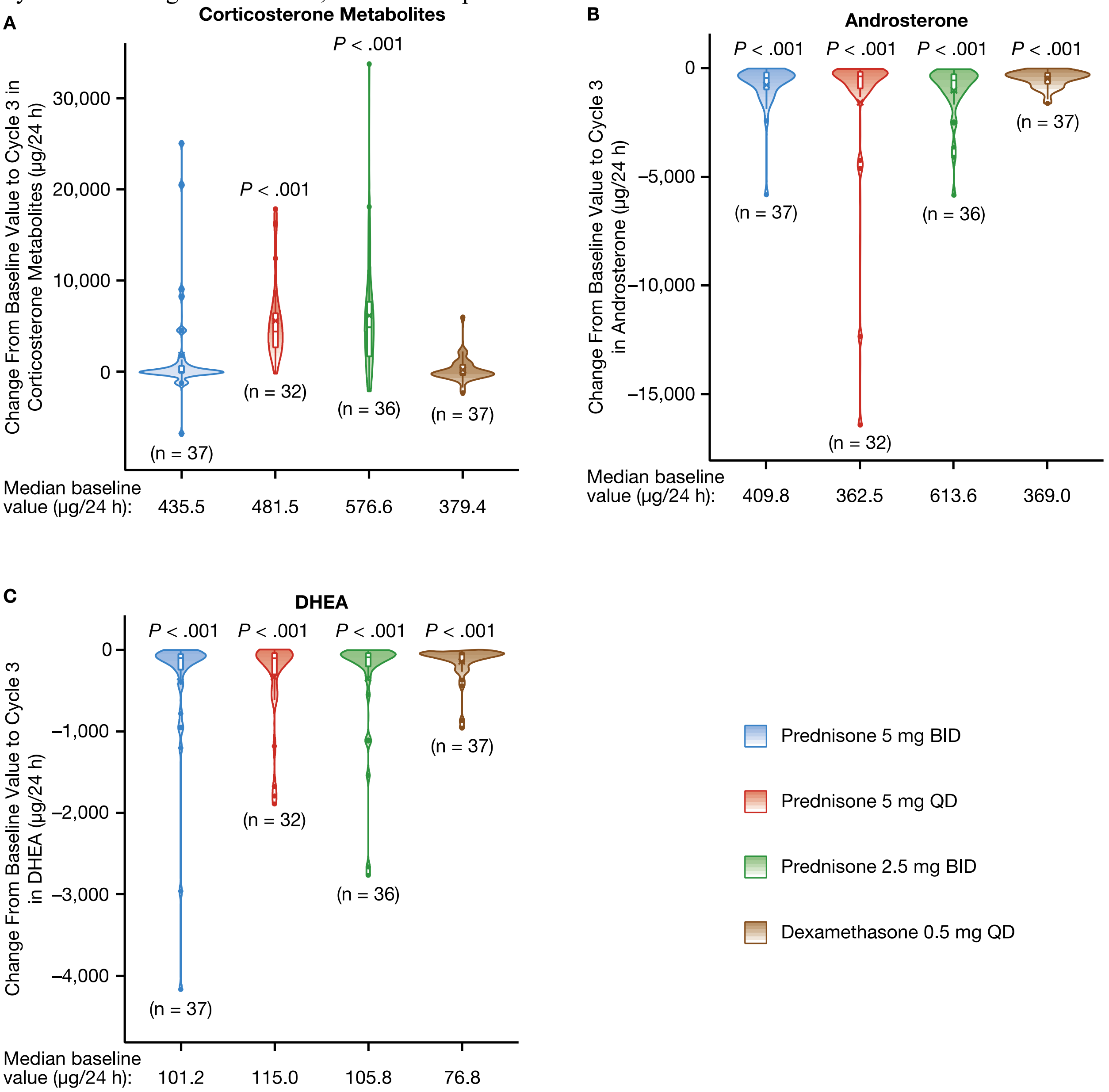


© 2019 Attard G et al. *JAMA Oncology*.

**eFigure 3. Values at cycle 3 for the urinary metabolites of corticosterone (A) and cortisone (B) among patients experiencing or not experiencing clinical mineralocorticoid excess toxicity (grade ≥2 hypertension or grade ≥1 hypokalemia) in the first 24 weeks**

Violin plots represent the four treatment regimen arms. Boxes represent median (horizontal bar), mean (X) and quartiles (Q1 and Q3), and whiskers indicate quartiles ± 1.5 × (Q3-Q1). Minimum and maximum values are the lowest and highest dots. Significant differences between patients experiencing or nor experiencing clinical mineralocorticoid toxicity, by Wilcoxon two-sample test, are noted.

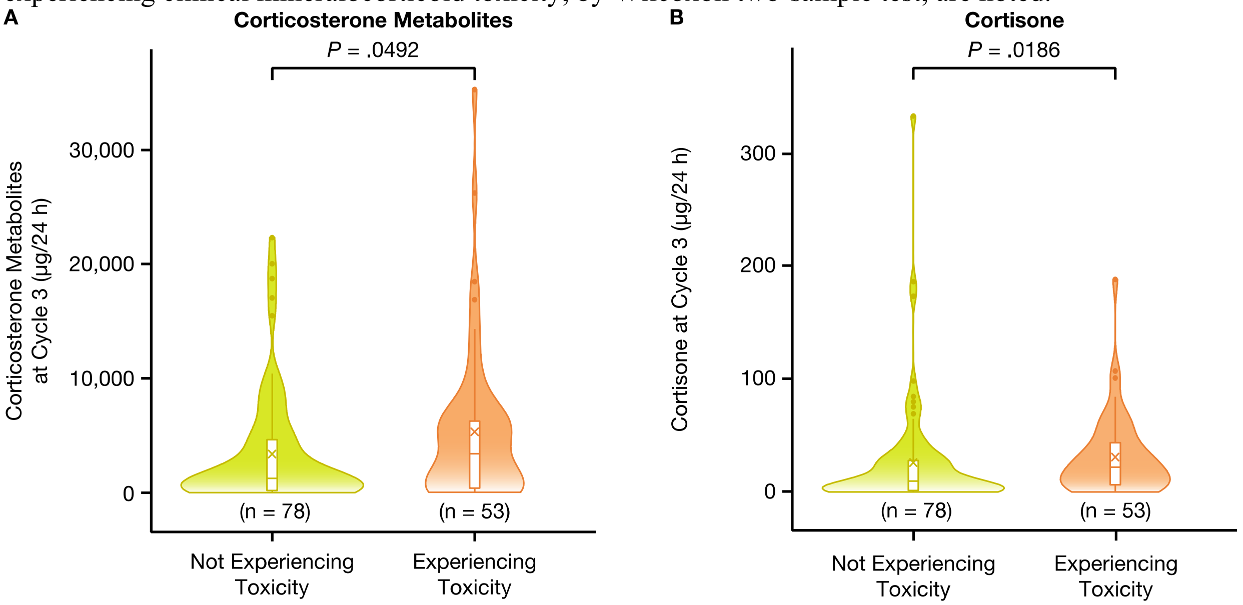


© 2019 Attard G et al. *JAMA Oncology*.

**eFigure 4. Cumulative incidences for adverse events of hypokalemia or hypertension.**
Note: this figure includes adverse events at any time during the main study treatment period; the primary study endpoint in Table 1 included only the first 24 weeks (6 cycles).

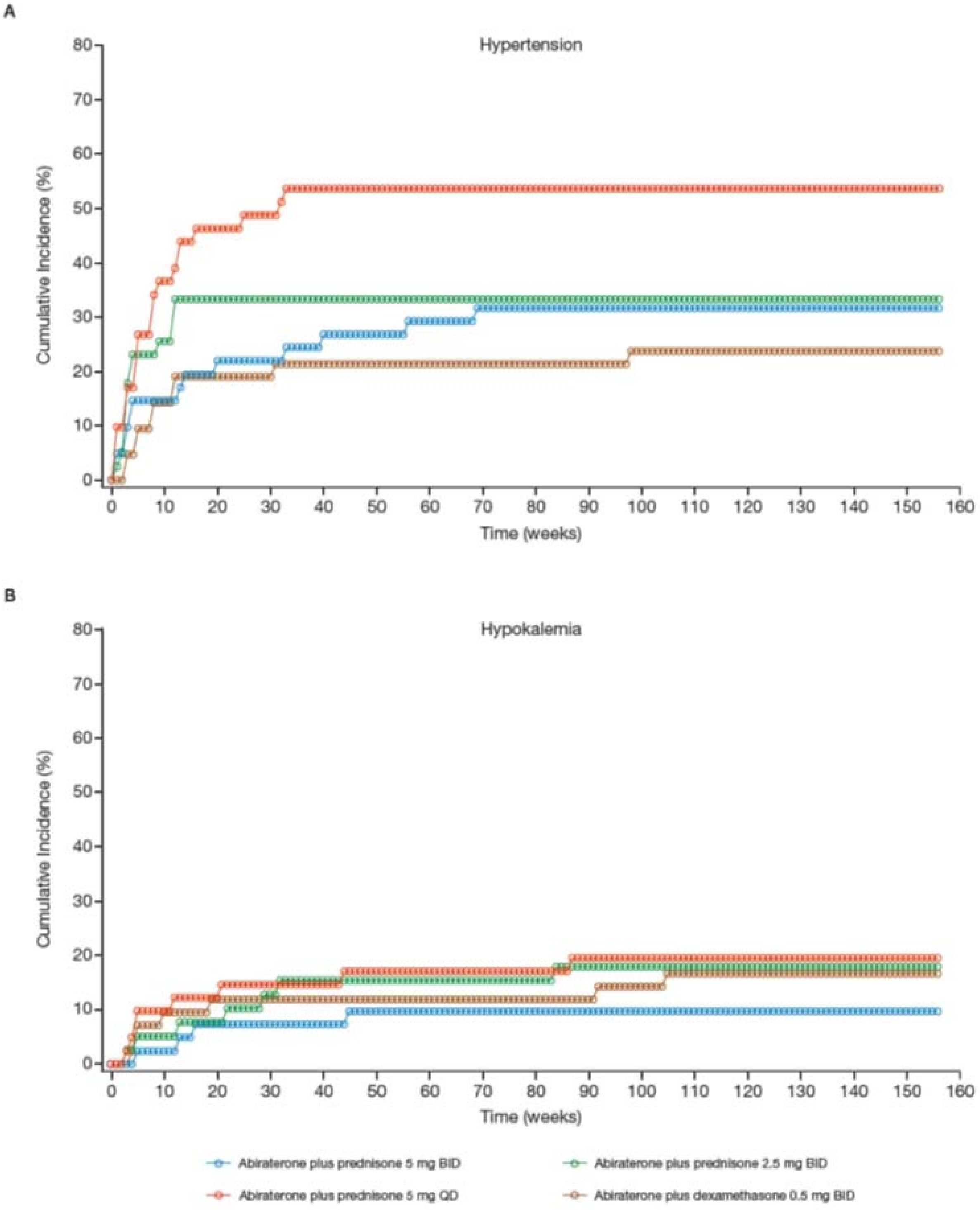


© 2019 Attard G et al. *JAMA Oncology*.

**eFigure 5. Change from baseline in fasting serum insulin (A) and HOMA-IR (B), and percentage change from baseline in total lean body mass (C) and total body fat (D) at end of main study (up to 39 cycles).**

HOMA-IR, homeostatic model assessment–insulin resistance; LOCF, last observation carried forward. Boxes represent median (horizontal bar) with 95% CIs, and whiskers indicate minimum and maximum values. Significant changes from baseline, by Wilcoxon Signed Rank test, are noted in each panel.

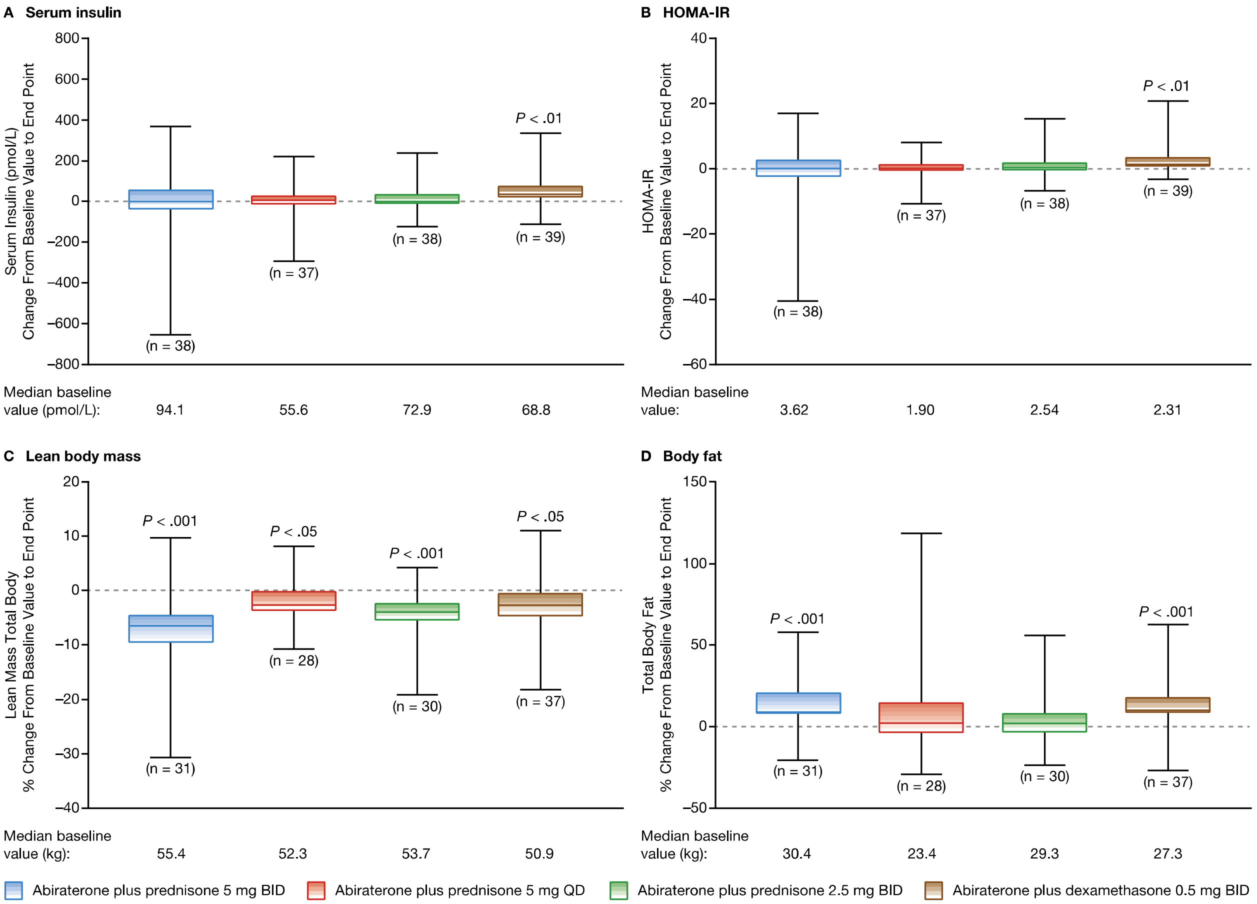


© 2019 Attard G et al. *JAMA Oncology*.

**eFigure 6. Clinical benefit: Kaplan-Meier plot of radiographic progression-free survival during the main study for intention-to-treat population, divided by treatment group (A); and patient-reported quality of life as measured by EQ-5D-5L and FACT-P (B)**

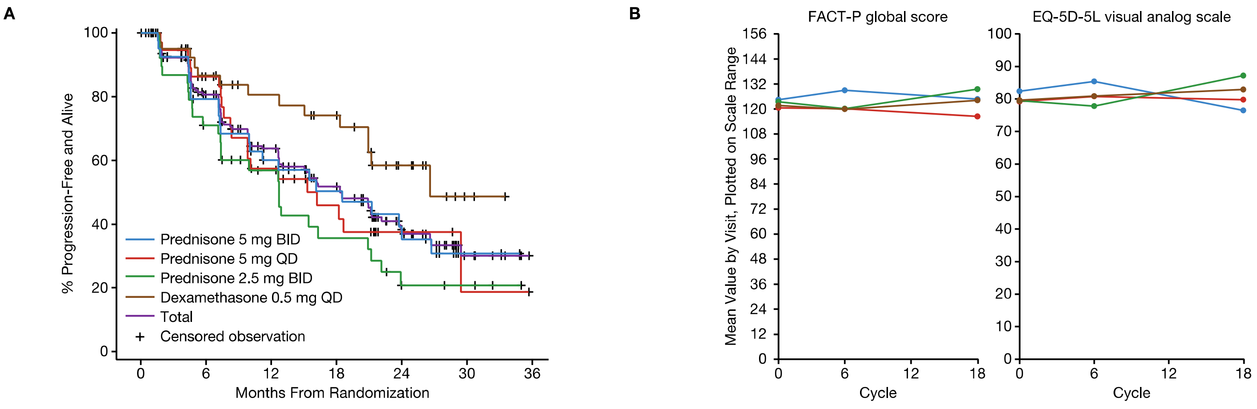


| Subjects at risk | 0 | 6 | 12 | 18 | 24 | 30 | 36 |
|---|---|---|---|---|---|---|---|
| Pred 5 mg BID | 41 | 29 | 20 | 15 | 10 | 2 | 0 |
| Pred 5 mg QD | 41 | 29 | 17 | 11 | 4 | 1 | 0 |
| Pred 2.5 mg BID | 40 | 26 | 17 | 10 | 4 | 3 | 0 |
| Dex 0.5 mg QD | 42 | 31 | 25 | 21 | 10 | 2 | 0 |
| Total | 164 | 115 | 79 | 57 | 28 | 8 | 0 |

| No. of patients with observed data, by cycle | FACT-P global score | | | EQ-5D-5L visual analog scale | | |
|---|---|---|---|---|---|---|
| Cycle | 0 | 6 | 18 | 0 | 6 | 18 |
| Pred 5 mg BID | 35 | 28 | 16 | 38 | 34 | 16 |
| Pred 5 mg QD | 38 | 30 | 13 | 37 | 31 | 12 |
| Pred 2.5 mg BID | 36 | 29 | 10 | 37 | 32 | 11 |
| Dex 0.5 mg QD | 34 | 30 | 18 | 37 | 34 | 20 |

© 2019 Attard G et al. *JAMA Oncology*.

**eTable 1. Study investigators and centers**

**Belgium:**

- Betrand Tombal, Clinique Universitaires Saint-Luc, Brussels
- Steven Joniau, Universitair Ziekenhuis Leuven, Leuven
- Patrick Werbrouck, AZ Groeninge, Kortrijk
- Peter Schatteman, Onze-Lieve-Vrouwe Ziekenhuis, Aalst
- Sylvie Rottey, Universitair Ziekenhuis Gent, Gent
- Daisy Luyten, Jessa Ziekenhuis, Hasselt

**Germany:**

- Axel Merseburger, Medizinische Hochschule Hannover Klinik für Urologie und Urologische Onkologie, Hannover
- Arnulf Stenzl, Universitätsklinikum Tübingen Klinik und Poliklinik für Urologie, Tübingen
- Susan Feyerabend, Studienpraxis Urologie, Nürtingen

**Great Britain:**

- Gerhardt Attard, Royal Marsden NHS Foundation Trust, Downs Road, Sutton
- Anjali Zarkar, Queen Elizabeth Hospital, Birmingham
- Robert Jones, Beatson West of Scotland Cancer Centre, Glasgow
- Gerhardt Attard, Royal Marsden NHS Foundation Trust, Fulham Road, London
- Michael Button, Velindre Cancer Centre, Cardiff

**Hungary:**

- Peter Tenke, Jahn Ferenc Dél-Pesti Kórház, Budapest
- Laszlo Landherr, Uzsoki Utcai Kórház, Budapest
- Lajos Hencz, BAZ Megyei Kórház és Egyetemi Oktatókórház, Miskolc
- Lajos Géczi, Országos Onkológiai Intézet, Budapest

**Italy:**

- Cora Sternberg, Ospedale San Camillo Forlanini, Roma
- Alfredo Berruti, Azienda Ospedaliera Spedali Civili Brescia, Brescia
- Gaetano Facchini, Istituto Nazionale Tumori – IRCCS – Fondazione G. Pascale, Napoli
- Enrico Cortesi, Sapienza Università di Roma, Roma

© 2019 Attard G et al. *JAMA Oncology*.

**eTable 2. Full list of urinary steroid metabolites analyzed by gas chromatography-mass spectrometry**

Androsterone
Etiocholanolone
Dehydroepiandrosterone
11-oxoetiocholanolone
17 alpha-hydroxyprogesterone
11 beta-hydroxy androsterone
11 beta-hydroxyetiocholanolone
3 alpha 5 alpha 17 alpha-hydroxyprogesterone
16-alpha hydroxydehydroepiandrosterone
Pregnanediol
Pregnanetriol
5-Pregnenediol
Tetrahydro-11-deoxy-cortisol
Tetrahydroxycorticosterone
5 alpha tetrahydrodeoxycorticosterone
Pregnanetriolone
Tetrahydrocortisone
5-Pregnen-3-beta, 17-alpha, 20-alpha-triol
Tetrahydroaldosterone
Tetrahydrocorticosterone
5 alpha tetrahydrocorticosterone
5 alpha tetrahydroaldosterone
beta-Tetrahydrocortisol
5 alpha Tetrahydrocortisol
alpha-cortolone
3 alpha, 5-beta-tetrahydro-aldosterone
beta-cortol
beta-cortolone
alpha-cortol
Cortisone
Cortisol
6-beta hydroxycortisol

© 2019 Attard G et al. *JAMA Oncology.*

**eTable 3. Baseline characteristics**

| | Abiraterone acetate with | | | |
|---|---|---|---|---|
| | Prednisone<br>5 mg BID<br>(n=41) | Prednisone<br>5 mg QD<br>(n=41) | Prednisone<br>2.5 mg BID<br>(n=40) | Dexamethasone<br>5 mg QD<br>(n=42) |
| Age (years)<br>Median (range) | 68.0 (50-88) | 69.0 (54-88) | 70.0 (67-72) | 71.0 (54-90) |
| Body-mass index (kg/m$^2$)<br>Mean (SD) | 27.8 (4.5) | 26.4 (3.3) | 28.4 (3.9) | 27.0 (3.8) |
| Gleason score at diagnosis | | | | |
| ≤ 7 | 19 (51.4%) | 9 (23.7%) | 17 (43.6%) | 21 (52.5%) |
| 8-10 | 18 (48.6%) | 29 (76.3%) | 22 (56.4%) | 19 (47.5%) |
| Missing | 4 | 3 | 1 | 2 |
| Metastasis stage at diagnosis | | | | |
| M0 | 22 (66.7%) | 11 (37.9%) | 13 (50.0%) | 26 (81.3%) |
| M1 | 11 (33.3%) | 18 (62.1%) | 13 (50.0%) | 6 (18.8%) |
| Missing | 8 | 12 | 14 | 10 |
| Time from diagnosis of prostate cancer to randomization (months) | | | | |
| Mean (SD) | 66.0 (56.1) | 69.2 (53.9) | 59.6 (38.4) | 101.4 (65.0) |
| Missing | 0 | 0 | 1 | 0 |
| Time from start of systemic therapy to randomization (months) | | | | |
| Mean (SD) | 57.9 (43.7) | 55.3 (40.5) | 48.0 (30.2) | 82.1 (61.2) |
| Missing | 0 | 0 | 0 | 0 |
| ECOG performance status at baseline | | | | |
| 0 | 37 (90.2%) | 41 (100.0%) | 34 (87.2%) | 38 (90.5%) |
| 1 | 4 (9.8%) | 0 | 5 (12.8%) | 4 (9.5%) |
| Missing | 0 | 0 | 1 | 0 |
| PSA at baseline (µg/L) | | | | |
| Median<br>(range) | 55.3<br>(1.6-792.7) | 49.3<br>(1.5-1537.0) | 50.3<br>(2.2-893.7) | 38.5<br>(0.7-712.0) |
| Missing | 0 | 0 | 2 | 0 |
| Measurable disease at baseline | | | | |
| Measurable disease* | 20 (90.9%) | 18 (60.0%) | 11 (61.1%) | 16 (64.0%) |
| Missing | 19 | 11 | 22 | 17 |
| Metastatic sites at baseline | | | | |
| Bone | 33 (80.5%) | 32 (78.0%) | 34 (87.2%) | 33 (80.5%) |
| Nodes | 19 (46.3%) | 25 (61.0%) | 16 (41.0%) | 21 (51.2%) |
| Lung† | 0 | 1 (2.4%) | 0 | 2 (4.9%) |
| Liver† | 0 | 1 (2.4%) | 0 | 1 (2.4%) |
| Missing | 0 | 0 | 1 | 1 |
| Bone lesions at baseline | | | | |
| None | 8 (20.0%) | 10 (25.0%) | 6 (15.8%) | 9 (21.4%) |
| 1-4 | 8 (20.0%) | 11 (27.5%) | 9 (23.7%) | 15 (35.7%) |

© 2019 Attard G et al. *JAMA Oncology*.

| | | | | |
|---|---|---|---|---|
| 5-10 | 9 (22.5%) | 4 (10.0%) | 8 (21.1%) | 5 (11.9%) |
| > 10 | 15 (37.5%) | 15 (37.5%) | 15 (39.5%) | 13 (31.0%) |
| Missing | 1 | 1 | 2 | 0 |
| Blood pressure at baseline‡ | | | | |
| Stage <1§ | 30 (73.2%) | 22 (53.7%) | 28 (71.8%) | 28 (66.7%) |
| Stage 1‖ | 9 (22.0%) | 18 (43.9%) | 9 (23.1%) | 13 (31.0%) |
| Stage 2 or higher¶ | 2 (4.9%) | 1 (2.4%) | 2 (5.1%) | 1 (2.4%) |
| Missing | 0 | 0 | 1 | 0 |
| Baseline medications | | | | |
| Any antihypertensive | 23 (56.1%) | 16 (39.0%) | 21 (52.5%) | 23 (54.8%) |
| Potassium supplementation | 0 | 0 | 0 | 0 |
| Any lipid-modifying agent | 14 (34.1%) | 11 (26.8%) | 10 (25.0%) | 14 (33.3%) |
| Any bisphosphonate | 2 (4.9%) | 0 | 1 (2.5%) | 2 (4.8%) |
| Denosumab | 0 | 2 (4.9%) | 2 (5.0%) | 2 (4.8%) |
| Chemistry tests, mean (SD) | | | | |
| Potassium (mmol/L) | 4.32 (0.417) | 4.26 (0.432) | 4.29 (0.359) | 4.39 (0.499) |
| Lactate dehydrogenase (U/L) | 373.8 (371.5) | 256.8 (89.7) | 328.1 (266.5) | 289.7 (171.1) |
| Alkaline phosphatase (IU/L) | 194.0 (169.8) | 167.0 (128.4) | 194.5 (137.1) | 171.3 (161.4) |
| Albumin (g/L) | 42.18 (4.60) | 41.80 (4.26) | 43.63 (3.33) | 43.00 (3.90) |
| Insulin (pmol/L) | 125.3 (128.9) | 73.2 (62.9) | 95.6 (75.4) | 72.3 (40.2) |
| HOMA-IR | 5.26 (7.30) | 2.54 (2.23) | 3.60 (3.52) | 2.61 (1.55) |

ECOG=Eastern Cooperative Oncology Group; PSA=prostate specific antigen. Data are n (%), mean (SD), or median (range). *Per RECIST guidelines v1.1, measurable disease was defined as at least one target lesion with longest diameter ≥10 mm by computed tomography scan. †The initial study protocol excluded patients with lung or liver metastases; after 61 of 164 patients were enrolled, a protocol amendment included patients with lung or liver metastases. ‡Baseline blood pressure was the lowest of 3 consecutive measurements at the baseline visit. §Systolic blood pressure <140 mmHg and diastolic blood pressure <90 mmHg. ‖Systolic blood pressure 140-159 mmHg and/or diastolic blood pressure 90-99 mmHg. ¶Systolic blood pressure ≥160 mmHg and/or diastolic blood pressure ≥100 mmHg

© 2019 Attard G et al. *JAMA Oncology.*

**eTable 4. Changes from baseline to cycle 3 by treatment group for plasma ACTH and key urinary steroid metabolites**

| | Abiraterone with | | | |
|---|---|---|---|---|
| | Prednisone<br>5 mg BID<br>(n=41) | Prednisone<br>5 mg QD<br>(n=41) | Prednisone<br>2.5 mg BID<br>(n=39) | Dexamethasone<br>0.5 mg QD<br>(n=42) |
| Plasma adrenocorticotropic hormone | | | | |
| Absolute change (pmol/L) | | | | |
| Median | -1.07 | 8.95 | 3.97 | -1.82 |
| IQR | -3.08 to 0.30 | 4.39 to 12.63 | 0.42 to 9.24 | -3.39 to -0.10 |
| *P* | .1602 | <.0001 | .0001 | .0155 |
| Percentage change (%) | | | | |
| Median | -27 | 189 | 101 | -39 |
| IQR | -71 to 26 | 115 to 338 | 6 to 194 | -63 to -4 |
| *P* | .2667 | <.0001 | <.0001 | .0527 |
| Urinary deoxycorticosterone metabolites | | | | |
| Absolute change (µg/24h) | | | | |
| Median | 11.4 | 75.9 | 100.5 | 5.1 |
| IQR | -1.9 to 60.9 | 46.7 to 176.2 | 36.9 to 158.6 | -6.2 to 20.9 |
| *P* | .0007 | <.0001 | <.0001 | .0708 |
| Percentage change (%) | | | | |
| Median | 149 | 862 | 716 | 35 |
| IQR | -15 to 324 | 351 to 1491 | 402 to 1391 | -39 to 337 |
| *P* | <.0001 | <.0001 | <.0001 | .0074 |
| Urinary corticosterone metabolites | | | | |
| Absolute change (µg/24h) | | | | |
| Median | 17.8 | 4452.6 | 4913.5 | -180.1 |
| IQR | -99.1 to 646.2 | 2610.2 to 6573.0 | 1650.6 to 7787.9 | -355.6 to 798.2 |
| *P* | .1906 | <.0001 | <.0001 | .7958 |
| Percentage change (%) | | | | |
| Median | 13 | 984 | 1016 | -42 |
| IQR | -37 to 130 | 622 to 1851 | 471 to 1846 | -79 to 135 |
| *P* | .084 | <.0001 | <.0001 | .7505 |
| Urinary androsterone | | | | |
| Absolute change (µg/24h) | | | | |
| Median | -429.2 | -367.3 | -548.0 | -364.8 |
| IQR | -961.5 to -190.7 | -922.7 to -147.9 | -1105.0 to -272.8 | -703.1 to -200.1 |
| *P* | <.0001 | <.0001 | <.0001 | <.0001 |
| Percentage change (%) | | | | |
| Median | -100 | -99 | -99 | -100 |
| IQR | -100 to -99 | -100 to -98 | -100 to -98 | -100 to -99 |
| *P* | <.0001 | <.0001 | <.0001 | <.0001 |
| Urinary dehydroepiandrosterone | | | | |
| Absolute change (µg/24h) | | | | |
| Median | -100.6 | -103.5 | -92.6 | -58.0 |
| IQR | -242.1 to -51.3 | -346.3 to -39.3 | -205.3 to -38.5 | -151.0 to -38.2 |
| *P* | <.0001 | <.0001 | <.0001 | <.0001 |
| Percentage change (%) | | | | |
| Median | -97 | -94 | -90 | -96 |

© 2019 Attard G et al. *JAMA Oncology.*

| | | | | |
|---|---|---|---|---|
| IQR | -99 to -93 | -98 to -89 | -97 to -82 | -98 to -88 |
| *P* | <.0001 | <.0001 | <.0001 | <.0001 |
| Urinary androgen precursors | | | | |
| Absolute change (µg/24h) | | | | |
| Median | -330.8 | -37.0 | -48.9 | -262.1 |
| IQR | -555.5 to -165.8 | -189.2 to 68.0 | -319.9 to 210.3 | -369.0 to -89.5 |
| *P* | <.0001 | .1823 | .3371 | <.0001 |
| Percentage change (%) | | | | |
| Median | -81 | -11 | -14 | -88 |
| IQR | -89 to -63 | -50 to 42 | -53 to 39 | -93 to -56 |
| *P* | <.0001 | .6608 | .8899 | <.0001 |

© 2019 Attard G et al. *JAMA Oncology*.

**eTable 5. Urinary steroid metabolites with *P*<0.1 for the comparison of values at cycle 3 day 1 between patients with or without clinical mineralocorticoid excess toxicity in the first 24 weeks**

| Urinary Steroid Metabolite at Cycle 3 Day 1 | *P* for Patients With vs Without Toxicity in First 24 Weeks* |
|---|---|
| 16a-hydroxy-DHEA | 0.0710 |
| 17-hydroxy-pregnanolone | 0.0575 |
| 3a, 5a-17-hydroxy-pregnanolone | 0.0204 |
| 5a-tetrahydro-11-dehydro-corticosterone | 0.0706 |
| 5a-tetrahydro-corticosterone | 0.0481 |
| Corticosterone metabolites | 0.0492 |
| Cortisol | 0.0148 |
| Cortisone | 0.0186 |
| Pregnanetriol | 0.0536 |
| Pregnanetriolone | 0.0775 |
| Pregnenetriol | 0.0710 |
| Tetrahydro-11-dehydro-corticosterone | 0.0554 |
| Tetrahydro-11-deoxycorticosterone | 0.0831 |
| Tetrahydro-corticosterone | 0.0395 |
| Tetrahydrocortisol | 0.0701 |
| Tetrahydrocortisone | 0.0287 |
| a-cortol | 0.0699 |
| a-cortolone | 0.0660 |
| ß-cortolone | 0.0671 |

**P* by Wilcoxon two-sample test with no correction for multiple testing.

© 2019 Attard G et al. *JAMA Oncology*.

**eTable 6. Adverse events of hypokalemia by treatment group and severity**

| | Abiraterone with | | | |
|---|---|---|---|---|
| | Prednisone 5 mg BID (n=41) | Prednisone 5 mg QD (n=41) | Prednisone 2.5 mg BID (n=39) | Dexamethasone 0.5 mg QD (n=42) |
| Any grade | 4 (9.8%) | 8 (19.5%) | 7 (17.9%) | 7 (16.7%) |
| Grade 1 (mild) | 2 (4.9%) | 4 (9.8%)* | 4 (10.3%) | 5 (11.9%) |
| Grade 2 (moderate) | 2 (4.9%) | 2 (4.9%)* | 3 (7.7%) | 2 (4.8%) |
| Grade 3 (severe) | 0 | 3 (7.3%) | 0 | 0 |
| Grade 4 (life-threatening) | 0 | 0 | 0 | 0 |
| Grade 5 (fatal) | 0 | 0 | 0 | 0 |

One patient in the prednisone 5 mg QD group experienced both grade 1 and grade 2 hypokalemia.

© 2019 Attard G et al. *JAMA Oncology*.

**eTable 7. Adverse events of special interest during the main study (up to 39 cycles)**

| | Abiraterone with | | | | | | | |
|---|---|---|---|---|---|---|---|---|
| | Prednisone 5 mg BID (n=41) | | Prednisone 5 mg QD (n=41) | | Prednisone 2.5 mg BID (n=39) | | Dexamethasone 0.5 mg QD (n=42) | |
| Any adverse event | 40 (97.6%) | | 37 (90.2%) | | 38 (97.4%) | | 42 (100.0%) | |
| Any grade 3-4 adverse event | 11 (26.8%) | | 17 (41.5%) | | 13 (33.3%) | | 18 (42.9%) | |
| Any serious adverse event | 9 (22.0%) | | 9 (22.0%) | | 11 (28.2%) | | 15 (35.7%) | |
| Adverse event leading to death | 1 (2.4%) | | 1 (2.4%) | | 3 (7.7%) | | 2 (4.8%) | |
| Adverse events of interest† | All grades | Grade 3-4 | All grades | Grade 3-4 | All grades | Grade 3-4 | All grades | Grade 3-4 |
| Hypertension | 12 (29.3%) | 3 (7.3%) | 22 (53.7%) | 9 (22.0%) | 13 (33.3%) | 5 (12.8%) | 9 (21.4%) | 3 (7.1%) |
| Edema peripheral | 8 (19.5%) | 0 | 4 (9.8%) | 0 | 3 (7.7%) | 0 | 8 (19.0%) | 0 |
| Hypokalemia | 4 (9.8%) | 0 | 8 (19.5%) | 3 (7.3%) | 7 (17.9%) | 0 | 7 (16.7%) | 0 |
| Weight increased | 4 (9.8%) | 1 (2.4%) | 2 (4.9%) | 0 | 1 (2.6%) | 0 | 6 (14.3%) | 0 |
| ALT increased | 3 (7.3%) | 1 (2.4%) | 2 (4.9%) | 1 (2.4%) | 3 (7.7%) | 0 | 5 (11.9%) | 1 (2.4%) |
| Blood bilirubin increased | 3 (7.3%) | 0 | 0 | 0 | 1 (2.6%) | 0 | 0 | 0 |
| Fatigue | 2 (4.9%) | 0 | 6 (14.6%) | 0 | 4 (10.3%) | 0 | 6 (14.3%) | 0 |
| Osteopenia | 2 (4.9%) | 0 | 3 (7.3%) | 0 | 0 | 0 | 1 (2.4%) | 0 |
| AST increased | 1 (2.4%) | 0 | 2 (4.9%) | 1 (2.4%) | 3 (7.7%) | 0 | 4 (9.5%) | 0 |
| Blood alkaline phosphatase increased | 1 (2.4%) | 0 | 1 (2.4%) | 1 (2.4%) | 1 (2.6%) | 0 | 3 (7.1%) | 0 |
| Osteoporosis | 1 (2.4%) | 0 | 1 (2.4%) | 0 | 1 (2.6%) | 0 | 3 (7.1%) | 0 |
| Atrial flutter | 0 | 0 | 1 (2.4%) | 1 (2.4%) | 0 | 0 | 0 | 0 |
| Left ventricular failure | 0 | 0 | 1 (2.4%) | 0 | 0 | 0 | 0 | 0 |
| Atrioventricular block first degree | 0 | 0 | 0 | 0 | 1 (2.6%) | 0 | 0 | 0 |
| Atrioventricular block second degree | 0 | 0 | 0 | 0 | 1 (2.6%) | 0 | 0 | 0 |
| Cardiac failure | 0 | 0 | 0 | 0 | 1 (2.6%) | 1 (2.6%) | 0 | 0 |

© 2019 Attard G et al. *JAMA Oncology*.

| | | | | | | | | |
|---|---|---|---|---|---|---|---|---|
| Ventricular arrhythmia | 0 | 0 | 0 | 0 | 1 (2.6%) | 0 | 0 | 0 |
| Hyperbilirubinemia | 0 | 0 | 0 | 0 | 1 (2.6%) | 0 | 0 | 0 |
| Supraventricular tachyarrhythmia | 0 | 0 | 0 | 0 | 0 | 0 | 1 (2.4%) | 1 (2.4%) |
| Hyperglycemia | 0 | 0 | 0 | 0 | 0 | 0 | 2 (4.8%) | 0 |

ALT=alanine aminotransferase; AST=aspartate aminotransferase; BID=twice daily; QD=once daily. * Events that were reported at any grade for at least 5% of patients in any group (listed in descending order for the prednisone 5 mg BID group). † Adverse event of interest based on phase 2 and phase 3 studies of abiraterone acetate plus prednisone; all reported adverse events of interest are listed in descending order for the prednisone 5 mg BID group.

© 2019 Attard G et al. *JAMA Oncology*.

**eTable 8. Changes from baseline in bone mineral density - total body and arms**

| | Abiraterone with | | | |
|---|---|---|---|---|
| | Prednisone 5 mg BID (n=41) | Prednisone 5 mg QD (n=41) | Prednisone 2.5 mg BID (n=39) | Dexamethasone 0.5 mg QD (n=42) |
| Total body | | | | |
| Baseline | | | | |
| N | 32 | 33 | 31 | 37 |
| Mean (SD) | 1.20 (0.14) | 1.14 (0.16) | 1.22 (0.17) | 1.12 (0.14) |
| Median | 1.19 | 1.13 | 1.21 | 1.09 |
| 95% CI | (1.15, 1.25) | (1.09, 1.20) | (1.15, 1.28) | (1.07, 1.17) |
| Min, Max | (1.01; 1.70) | (0.82; 1.58) | (0.95; 1.63) | (0.88; 1.49) |
| Cycle 12, day 1 – % Change from Baseline | | | | |
| N | 23 | 20 | 14 | 27 |
| Mean (SD) | -0.96 (9.19) | 0.83 (6.04) | 0.50 (3.03) | -1.46 (4.36) |
| Median | -0.09 | -0.31 | 0.20 | -0.65 |
| 95% CI | (-4.93, 3.01) | (-1.99, 3.66) | (-1.25, 2.24) | (-3.18, 0.27) |
| Min, Max | (-33.31; 11.78) | (-10.08; 15.55) | (-4.87; 6.35) | (-15.54; 5.89) |
| p-value | 0.9531 | 0.8983 | 0.5830 | 0.1677 |
| End of main study – % Change from Baseline | | | | |
| N | 32 | 33 | 31 | 37 |
| Mean (SD) | -1.23 (8.82) | 0.08 (5.66) | -0.12 (5.57) | -2.01 (4.30) |
| Median | -0.44 | 0.66 | 0.52 | -1.26 |
| 95% CI | (-4.41, 1.95) | (-1.21, 2.80) | (-2.16, 1.93) | (-3.44, -0.57) |
| Min, Max | (-33.31; 15.59) | (-10.08; 15.55) | (-21.42; 9.18) | (-16.30; 5.09) |
| p-value | 0.6184 | 0.4888 | 0.6184 | 0.0153 |
| | | | | |
| Arms | | | | |
| Baseline | | | | |
| N | 32 | 33 | 30 | 37 |
| Mean (SD) | 1.27 (0.41) | 1.24 (0.40) | 1.38 (0.35) | 1.33 (0.38) |
| Median | 1.23 | 1.21 | 1.49 | 1.41 |
| 95% CI | (1.12, 1.42 | (1.10, 1.38) | (1.25, 1.50) | (1.21, 1.46) |
| Min, Max | (0.68; 2.17) | (0.57; 1.93) | (0.68; 2.06) | (0.68; 2.22) |
| p-value | | | | |
| Cycle 12, day 1 – % Change from Baseline | | | | |
| N | 21 | 22 | 14 | 26 |
| Mean (SD) | 3.30 (19.65) | 0.49 (14.24) | -1.81 (15.57) | -2.40 (9.69) |
| Median | 0.19 | 0.34 | -0.15 | -0.85 |
| 95% CI | (-5.64, 12.24) | (-5.83, 6.81) | (-10.80, 7.18) | (-6.32, 1.51) |
| Min, Max | (-49.97; 53.73) | (-51.71; 32.19) | (-49.15; 25.43) | (-34.00; 20.85) |
| p-value | 0.5789 | 0.6486 | 0.8077 | 0.1459 |
| End of main study – % Change from Baseline | | | | |
| N | 32 | 33 | 30 | 37 |
| Mean (SD) | 0.83 (18.75) | -2.05 (14.90) | -2.96 (18.50) | -4.91 (17.09) |
| Median | 0.26 | -0.47 | -0.16 | -0.42 |

© 2019 Attard G et al. *JAMA Oncology.*

| | Abiraterone with | | | |
|---|---|---|---|---|
| | Prednisone 5 mg BID (n=41) | Prednisone 5 mg QD (n=41) | Prednisone 2.5 mg BID (n=39) | Dexamethasone 0.5 mg QD (n=42) |
| 95% CI | (-5.93, 7.59) | (-7.34, 3.23) | (-9.87, 3.95) | (-10.61, 0.79) |
| Min, Max | (-50.23; 53.73) | (-52.66; 32.19) | (-51.42; 31.60) | (-54.84; 23.53) |
| p-value | 0.7982 | 0.5695 | 0.6659 | 0.1909 |

© 2019 Attard G et al. *JAMA Oncology*.

**eTable 9. Number of patients with any PSA response and confirmed PSA response during the main study (up to 39 cycles)**

| | Abiraterone with | | | |
|---|---|---|---|---|
| | Prednisone 5 mg BID (N=41) | Prednisone 5 mg QD (N=41) | Prednisone 2.5 mg BID (N=40) | Dexamethasone 0.5 mg QD (N=42) |
| Any PSA response | | | | |
| n (%) | 26 (63.4%) | 32 (78.0%) | 24 (60.0%) | 37 (88.1%) |
| 95% CI | (48.1, 76.4) | (63.3, 88.0) | (44.6, 73.7) | (75.0, 94.8) |
| | | | | |
| Confirmed PSA response* | | | | |
| n (%) | 25 (61.0%) | 30 (73.2%) | 21 (52.5%) | 34 (81.0%) |
| 95% CI | (45.7, 74.3) | (58.1, 84.3) | (37.5, 67.1) | (66.7, 90.0) |

PSA=prostate specific antigen. *Patients with a second documented reduction of ≥50% from baseline ≥4 weeks after the first reduction.

© 2019 Attard G et al. *JAMA Oncology*.
