# Supplementary material for: Assessment of the Safety of Glucocorticoid Regimens in Combination With Abiraterone Acetate for Metastatic Castration-Resistant Prostate Cancer: A Randomized, Open-label Phase 2 Study
Source: JAMA Oncol. 2019 Jun 27;5(8):1159–67. doi: 10.1001/jamaoncol.2019.1011 (PMC6604092; doi:10.1001/jamaoncol.2019.1011)
Supplement: Supplement 3. — Data Sharing Statement [file jamaoncol-5-1159-s003.pdf]

Data Sharing Statement

Attard. Assessment of the Safety of Glucocorticoid Regimens in Combination With Abiraterone Acetate. *JAMA Oncol*. Published August 08, 2019. 10.1001/jamaoncol.2019.1011

Data
Data available: Yes
Data types: Deidentified participant data
How to access data: http://yoda.yale.edu
When available: With publication

Supporting Documents
Document types: None

Additional Information
Who can access the data: researchers whose proposed use of the data has been approved
Types of analyses: for any research purpose
Mechanisms of data availability: after approval of a proposal
Any additional restrictions: cannot be used in pursuit of litigation or for commercial interests
